# Hepamine - A Liver Disease Microarray Database, Visualization Platform and Data-Mining Resource

**DOI:** 10.1038/s41598-020-61508-y

**Published:** 2020-03-16

**Authors:** Timo Itzel, Melanie Neubauer, Matthias Ebert, Matthias Evert, Andreas Teufel

**Affiliations:** 1 Division of Hepatology, Department of Medicine II, Medical Faculty Mannheim, Heidelberg University, Mannheim, Germany; 2 Division of Bioinformatics, Department of Medicine II, Medical Faculty Mannheim, Heidelberg University, Mannheim, Germany; 3 Department of Medicine II, Medical Faculty Mannheim, Heidelberg University, Mannheim, Germany; 4 Department of Pathology, University of Regensburg, Regensburg, Germany

**Keywords:** Data mining, Liver diseases

## Abstract

Numerous gene expression profiling data on liver diseases were generated and stored in public databases. Only few were used for additional analyses by the hepatology research community. This may mostly be due to limited bioinformatics knowledge of most biomedical research personnel. In order to support an easy translation of bioinformatics data into translational hepatology research, we created Hepamine, a liver disease gene expression, visualization platform and data-mining resource. Microarray data were obtained from the NCBI GEO database. Pre-analysis of expression data was performed using R statistical software and the limma microarray analysis package from the Bioconductor repository. We generated Hepamine, a web-based repository of pre-analyzed microarray data for various liver diseases. At its initial release Hepamine contains 13 gene expression datasets, 20 microarray experiments and approximately 400 000 gene expression measurements. A self-explanatory website offers open and easy access to gene expression profiles. Results are furthermore visualized in simple three-color tables indicating differential expression. All data were linked to common functional and genetic databases particularly through the DAVID bioinformatics suite. Hepamine provides comprehensive data and easy access to hepatologic gene expression data even without in depth bioinformatics or microarray profiling experience. http://www.hepamine.de.

## Introduction

Chronic liver disease is a major health burden worldwide. In particular viral hepatitis B and C but also an increasing number of patients with non-alcoholic fatty liver disease/non-alcoholic steatohepatitis (NAFLD/NASH)^1,2^ in developed countries result in a steadily increasing number of patients with liver fibrosis, cirrhosis or hepatocellular carcinoma (HCC) as the common end stage of chronic liver disease^3^.

Recent advances in drug development for viral hepatitis will certainly be beneficial for many patients^4^. However, for patients with fatty liver disease, liver fibrosis, cirrhosis or liver cancer still only limited therapeutic option are available^5,6^. Thus, investigating the molecular cause of many chronic liver diseases but also its common end stage fibrosis, cirrhosis and HCC are still urgently needed to provide better care for these patients^3^.

Over the past two decades, gene expression profiling of common liver diseases was extensively performed. Aim of this extensive survey of the liver transcriptome was the identification of “druggable” molecular targets and signaling networks to be targeted by specifically designed molecules. Even more, several groups aimed at using gene expression data particularly with respect to liver cancer in order to predict survival of patients or recurrence of this disease after resection. These efforts recently cumulated in the definition of consensus molecular subtypes (CMS) being helpful for prognosis prediction but also as an entry to precision medicine. As a result of these extensive efforts a vast amount of transcriptomic data was deposited in public databases and made publicly available. These data provide a rich source for further, comparative and integrative OMICS analysis to better understand the molecular basis of chronic liver disease. However, in order to utilize this information, knowledge of the available database resources but also bioinformatics tools are indispensable being a major hurdle in re-utilizing these data. Biomedical researchers are generally not capable of analyzing these data themselves due to a lack of programming skills. However, bioinformaticians are rather focused on the development of novel analysis algorithms.

In order to overcome this significant gap in knowledge transfer from molecular biology and bioinformatics to translational medical and ultimately clinical research, we provide Hepamine, a liver disease microarray database, visualization platform and data-mining resource, which provides easy but still detailed access to transcriptomic data on liver disease.

## Material and Methods

Microarray data were obtained from the NCBI GEO Gene Expression Omnibus Archive of Functional Genomics Data (https://www.ncbi.nlm.nih.gov/geo)^7^. Pre-analysis of expression data was performed using R statistical software and NCBI GEO´s geo2r scripts provided for each data set (https://www.ncbi.nlm.nih.gov/geo/geo2r)^8^. For reasons of comparing results between different experiments, all analysis scripts were adjusted to apply the newest array annotations using the hgu133plus2.db, hgu133a2.db, hgu133a.db, or hugene10sttranscriptcluster.db packages provided by the Bioconductor suite (https://www.bioconductor.org)^9^ (Table. 1). Next, all spots representing a specific gene were summarized by means of calculation an average expression in order to obtain a gene centered result. For further analysis, geo2r scripts were based on the Bioconductor limma package. Expression data were stored locally in a MySQL database. The business logic for visualization and filtering of the data was implemented on a python based Django 2.0 framework.

**Table 1:** Currently available pre-analyzed data sets.

Dataset	Array Platform		Label	Disease	Comparison	
	GEO	Annotation			Probe	Control
GSE11190	GPL570	hgu133plus2.db	E-GEOD-11190 HCV	HCV	Post-interferon liver biopsy	Pre-interferon liver biopsy
GSE14323	GPL571	hgu133a2.db	E-GEOD-14323 HCC	HCC	HCC	Normal
			E-GEOD-14323 cirrhosis	Fibrosis Cirrhosis	cirrhosis	Normal
GSE17548	GPL570	hgu133plus2.db	E-GEOD-17548 HCC	HCC	Ankara HCC tissue, Izmir HCC tissue	Ankara cirrhosis tissue, Izmir cirrhosis tissue
			E-GEOD-17548 HCC subset Ankara	HCC	Ankara HCC tissue	Ankara cirrhosis tissue
			E-GEOD-17548 HCC subset Izmir	HCC	Izmir HCC tissue	Izmir cirrhosis tissue
GSE17967	GPL571	hgu133a2.db	E-GEOD-17967 HCC	HCC	HCV + cirrhosis with HCC	HCV + cirrhosis without HCC
GSE26566	GPL6104		E-GEOD-26566 CCC	CCC	Cholangiocarcinoma	Normal intrahepatic bile duct
GSE32225	GPL8432		E-GEOD-32225 CCC	CCC	Human intrahepatic cholangiocarcinoma	Human normal biliary epithelial cells
GSE32958	GPL6244	hugene10sttranscriptcluster.db	E-GEOD-32958 CCC	CCC	Intrahepatic Cholangiocarcinoma (ICC)	Non-Tumor Tissue
	GPL6244	hugene10sttranscriptcluster.db	E-GEOD-32958 FNH	FNH	Focal Nodular Hyperplasia (FNH)	Non-Tumor Tissue
GSE33814	GPL6884		E-GEOD-33814 NASH	NAFLD NASH	steatohepatitis	normal
			E-GEOD-33814 Steatosis	NAFLD NASH	steatosis	normal
GSE38941	GPL570	hgu133plus2.db	E-GEOD-38941 HBV	HBV	HBV-associated acute liver failure	normal
GSE45001	GPL14550		E-GEOD-45001 CCC	CCC	Tumoral	Non-Tumoral
GSE46960	GPL6244	hugene10sttranscriptcluster.db	E-GEOD-46960 Biliary	Biliary Atresia	Biliary Atresia	Normal control
GSE49541	GPL570	hgu133plus2.db	E-GEOD-49541 Fibrosis	Fibrosis Cirrhosis	advanced (fibrosis stage 3–4)	mild (fibrosis stage 0–1)
GSE56140	GPL18461		E-GEOD-56140 HCC	HCC	hepatocellular carcinoma	cirrhosis
			E-GEOD-56140 HCC central	HCC	tumor central	cirrhosis
			E-GEOD-56140 HCC peripheral	HCC	tumor peripherial	cirrhosis

In order to support a functional characterization of the result tables, we provide predefined functionally related gene sets, e.g. signaling pathways (Supll. Table 1). Those (KEGG) pathways were obtained from MSigDB provided by Broad Institute^10^.

WikiPathways^11^ were obtained from the collaborative WikiPathways platform at https://www.wikipathways.org/index.php/Download_Pathways.

Included data sets were selected as follows: Firstly, all data sets related to chronic liver disease were selected from the GEO public functional genomics data repository (https://www.ncbi.nlm.nih.gov/geo). Secondly, only data sets with GEO2R available scripts were selected for further processing. Thirdly, the analysis script utilized provided series matrix files. If data sets did not contain such a series matrix file, the respective data were not further processed or integrated in our database. Finally, dual-channel arrays were also excluded from these analyses as their analysis algorithm does not fit the used analysis scripts.

### Web interface

**Figure 1:**
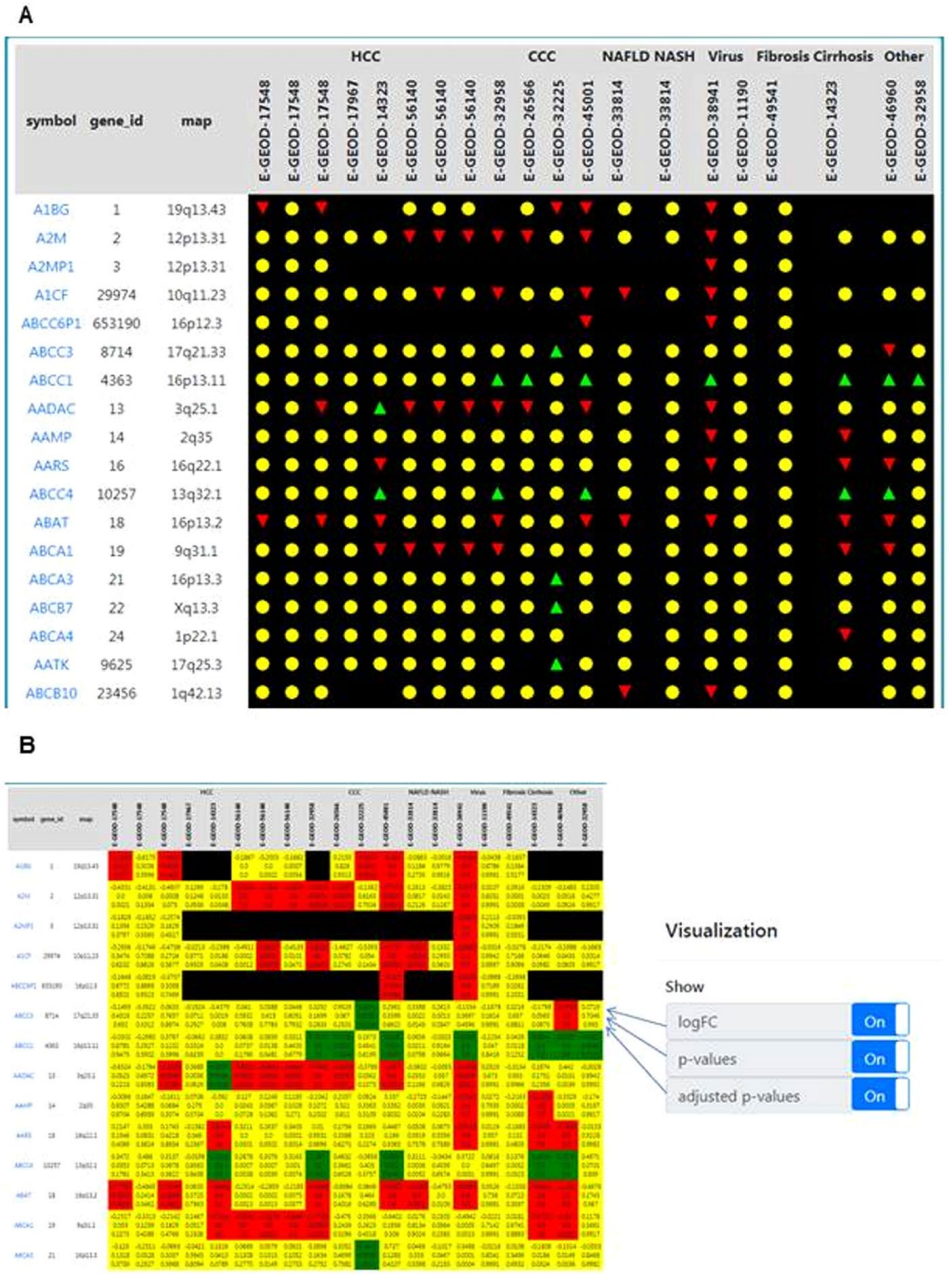
Hepamine visualization options of gene expression. (**A**) Simplified three-colour traffic light scheme:  up-regulated,  unchanged,  down-regulated (**B**) Custom view by selection from fold change, p-value and adjusted p-value.

After selecting the “Search Hepamine” option on the landing page, users will be guided to an initial data overview. This page contains all available data sets and lists all genes recognized in at least one of the available data sets. Primary data view displays gene expression as a three-way, traffic light visualization. Genes with a green arrow were upregulated, gene expression displayed as a yellow button was unchanged, and genes with a red arrow down were down regulated. This display offers an easy digestible overview of the excessive data accumulated (Fig. 1A).

However, as advanced users may be interested in more details about the differential gene expression in terms of fold-change, p-value, or adjusted p-value, Hepamine offers an alternative data view incorporating all this information for each gene in every data set. This alternative view may be selected by pressing the “Configure” button and selecting the respective features from the data filter panel on top of the page. Users may then select which features should be displayed selecting from fold change, p-value, and adjusted p-value. (Fig. 1B).

### Data selection options

**Figure 2:**
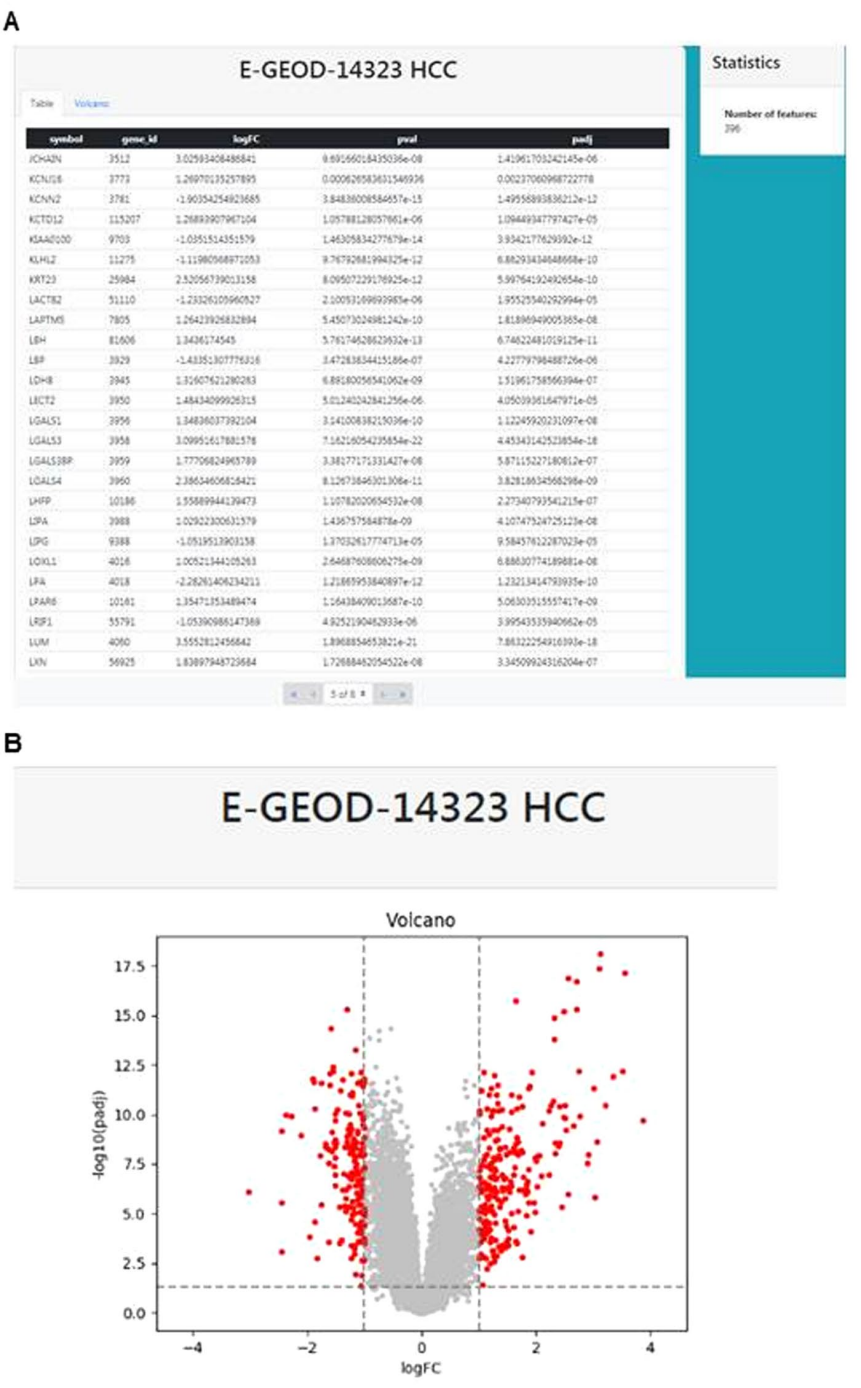
Additional data visualization options (analysis mode): (**A**) Result table, differential gene expression (**B**) Volcano plot.

As users may be interested in a particular set of genes, we offer diverse options to limit Hepamine searches to genes, gene sets, or diseases of interest. Selecting the “Search” button, users may insert a list of genes in which they are interested in and have the output results limited to only these genes. Given a total of up to 25 185 genes per analysis results pages (searching the full genome annotated genes), this option performs focused searches of our liver disease specific data. If the user is interested in the data of only one of the displayed datasets, there is a link to the analysis view. There the data are shown as table or volcano plot (Fig. 2).

Alternatively users may upload a gene list of interest via the same site. Data need to be formatted as a.txt file and contain official gene symbol abbreviations as defined by the HUGO Gene Nomenclature Committee (HGNC)^12^. Besides, we provide predefines gene sets (e.g. signaling pathways, gene ontologies) from the Kyoto Encyclopedia of Genes and Genomes (KEGG)^13^ or WikiPathways^11^ to specifically view functional related genes of interest.

Finally, selecting the “Visualize Context” option, users may limit their searches to any disease of interest: HCC, CCA, NAFLD/NASH, viral hepatitis, fibrosis/cirrhosis, or other.

### Data filtering options

Advanced users may change the selection criteria of significance within the data evaluation process. Again, the data filter bar on top of the page offers detailed selection options for fold change and adjusted p value of any preferred values.

Also, as some users may find it useful to perform more focused data searches, the top data filtering bar offers the option to select specific data sets such as HCC, CCA, viral hepatitis, NAFLD/NASH, fibrosis/cirrhosis, or other liver diseases such as biliary atresia.

### Functional data analysis

Having retrieved a list of genes and their differential expression in one or even more liver disease, a user may likely be interested in further characterizing their selected group of genes, e.g. by means of pathway analysis or gene set enrichment. Enabling such access to deeper transcriptional information was achieved by linking Hepamine to the DAVID (database for annotation, visualization and integrated discovery) bioinformatics analysis platform^14,15^. DAVID allows for detail information retrieval of gene annotation, visualization of results and integrated functional analysis. Upon selection of the “DAVID transfer” button, the gene list of interest/results are automatically transferred and DAVID results presented in a separate browser tab. Since the DAVID API only allows a fully automatic transfer of up to 400 genes, larger gene lists must be transferred in a semi-automated fashion. By activating the “DAVID transfer” button, users will have their gene list saved to the “clipboard”, obtain a detailed instruction on how to transfer their data, and be able to simply paste their selected gene list/content to the DAVID website. From thereon, users may continue using the DAVID website just as if they had used an automatic data transfer.

### Use case

As a potential use case for Hepamine we compared differential gene expression among different data sets of liver cancer (HCC) development in order to identify potential drivers of hepatocarcinogenesis being showing robust differential expression among all data sets.

**Figure 3:**
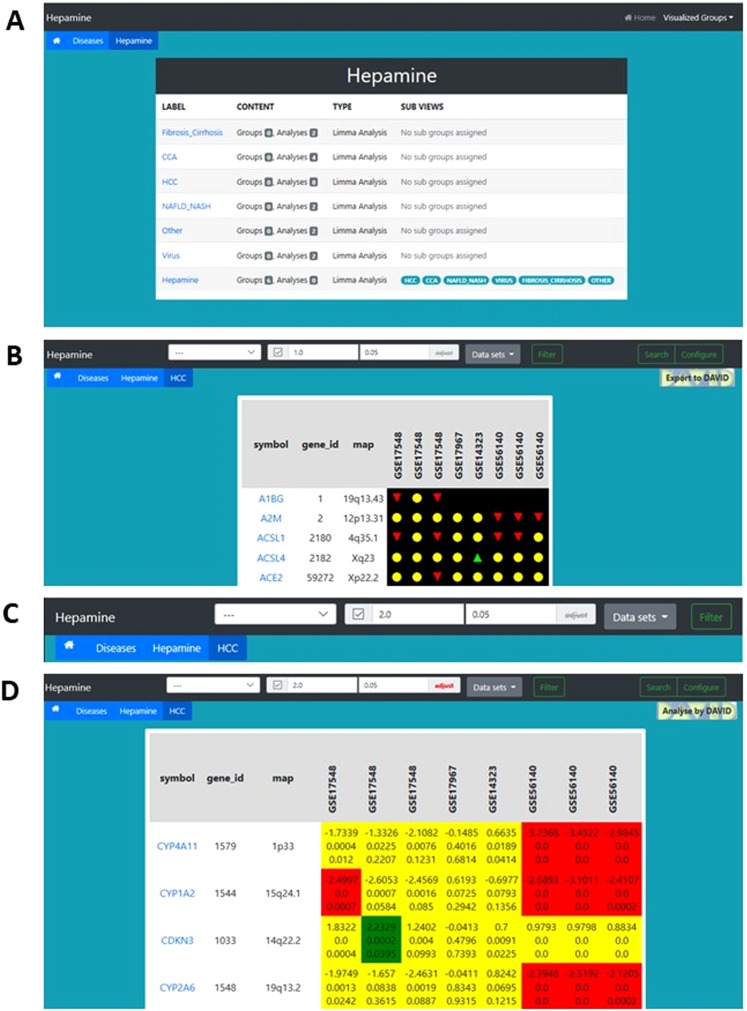
Screenshots of main analysis steps of the described use case HCC analysis. (**A**–**D**) Analysis in Hepamine.

Initial step was selection of all HCC related data sets from the “Diseases” panel. This selection resulted in a subset table of 4 available HCC datasets with a total of 8 subsets (Fig. 3A). As a default setting, gene expression profiles were depicted color coded: green arrow up meaning up regulation, red arrow down meaning down regulation, yellow circles signaling no changes in differential gene expression (Fig. 3B). The available GSE numbers of the available datasets were then used to confirm the corresponding experimental description. Switching back to the results table significance level for differential gene expression were set to 4-fold regulation (log_2_-fold 2) and a p-value of 0,05 (Fig. 3C). This search returned 223 genes being selected by these criteria.

For an even more stringed evaluation statistics adjusting for multiple testing was turned on selecting adjusted p-values “On”, at the right of the p-value field. Applying these parameters, we identified 175 genes being differentially expressed in at least one data set. By pressing the “Configure” button the visualization options become available. Selecting “logFC”, “p-values”, and “adjusted p-values” to be turned “On” resulted in a very detailed data table showing all important parameters fold-change, p-value, and adjusted p-value (Fig. 3D).

In order to get a functional assessment of these genes we automatically transferred these genes to DAVID via direct data transfer provided by Hepamine. Subsequently, these data were immediately available for further functional analysis to be selected from the DAVID Annotation summary results.

## Discussion and Conclusions

As outlined above, gene expression profiling of common liver diseases was extensively performed over the past two decades. Aim of most studies on the liver transcriptome were the identification of “druggable” molecular targets, identification of prognostic subgroups, or predictive markers to accompany the entry into an era of precision medicine. Furthermore, multiple studies for liver cancer aimed at using gene expression data particularly to predict survival of patients or recurrence of the disease after resection^16^.

However, the analysis of the large amount of publicly available data on gene expression in various liver diseases seems not feasible for biologists or physicians not familiar with bioinformatics or at least microarray analysis. Even with profound experience in microarray experiments, analysis of such data is a complex and time-consuming task. We therefore implemented a novel database resource, Hepamine, to aid fast access to key hepatological expression data sets for a broader community.

We expect the Hepamine database to be highly useful in correlating *in vitro*/*ex vivo* models of disease to *in vivo* patient data. Furthermore, comparison of gene expression in diverse liver diseases and progressive liver disease, e.g. hepatitis, liver fibrosis, and liver cirrhosis will be possible with Hepamine. Finally, as demonstrated in the supplied use case, robustness of gene expression data may be increased by comparing several data sets with the same disease. If being up- or downregulated in several data sets the genes can be much more trusted to be a true driver of disease development rather than being just a biologically not meaningful by-stander^17^

Since our database is the first of its kind and a novel tool in hepatological research, we set high value on a user friendly but also high performance usability for advanced bioinformatics analyses. To guarantee easy data access and connectivity we transformed this data base into a powerful web application. However, since requirements on such a web application may vary significantly depending on user’s needs, we offer two different data views. Traffic light visualization gives a fast and easy to digest overview on large gene expression data. It furthermore gives a quick impression on the consistency of expression data from different data sets reflecting gene expression changes of the same disease. In contrast, for users in need of a more detailed summary on the observed differential gene expressions, our data view displays all important numeric information for each gene and in every data set. This may be of interest in selecting genes with a high fold change or a strong p-value as it may be argued that expression changes of those genes have a higher probability of being robust in additional experiments to be potentially performed by the user. Those selections may furthermore be supported by means of selecting a higher stringency from the filter selection, increasing fold-change and/or p-value.

As advanced users may also want to further investigate a gene list consisting of genes being differentially expressed in a subset of liver diseases of interest we chose to automatically link our website to the DAVID bioinformatics resources consists of an integrated biological knowledgebase and analytic tools aimed at systematically extracting biological meaning from large gene/protein lists^14,15^. The DAVID web resource is also publicly available and contains approximately 68 bioinformatics enrichment tools. Among those functions are gene functional classification tools, functional annotation charts or clustering and functional annotation tables^14,15^.

DAVID supports automatic transfer of genes lists only up 400 genes. In order to at least ease transfer of larger genes lists our website supports a semi-automated fashion selecting a DAVID transfer button and subsequently copied into the working storage. Thus, users may easily paste their list into DAVID and continue using the website.

However, our web tool also has some short comings. Thus, it is obvious that microarray data are limited to genes known and annotated at the time of data production. In contrast, RNA sequencing data include additional unknown transcripts that could be later characterized and annotated. In order to ensure highest possible cooperativity among the diverse experiments performed under most likely highly different conditions, we chose at this stage only to incorporate data sets that could be evaluated using a Geo2R script provided by the GEO data repository^8^ and thus limited our selection to microarray data.

The fact that the selected experiments and respective data sets were not hybridized under the same or at least comparable conditions is also a major drawback of our data collection. However, at this point there is no large data set on liver diseases available (except for liver cancer, TCGA data) and our assembled gene expression array provides the most comprehensive overview. Given the diverse experimental modalities, users must be aware of the limited comparability of the selected data sets. Given these limitations our database is well suited for hypothesis generation – as demonstrated in the supplementary use case - but definitely not for validation of otherwise obtained hypotheses.

In summary, we implemented the Hepamine gene expression web-resource, which is easy to use but yet contains comprehensive data to close the knowledge gap from molecular biology and bioinformatics to translational medical and ultimately clinical research on liver diseases.

## Supplementary information

Supplementary information
