# Supplementary material for: Hepamine - A Liver Disease Microarray Database, Visualization Platform and Data-Mining Resource
Source: Sci Rep. 2020 Mar 16;10:4760. doi: 10.1038/s41598-020-61508-y (PMC7075926; doi:10.1038/s41598-020-61508-y)
Supplement: Supplementary file 1 — Supplementary information [file 41598_2020_61508_MOESM1_ESM.pdf]

Hepamine - A Liver Disease Microarray Database, Visualization Platform and Data-Mining Resource

Timo Itzel[1,2], Melanie Neubauer[1], Matthias Ebert[3], Matthias Evert[4], Andreas Teufel[1,2]

Supplemental Table 1
Predefined data sets available in Hepamine

Predefined gene sets in Hepamine

Data source	Pathways	Symbols
KEGG	Abc Transporters	ABCA1, ABCA2, ABCA3, ABCB7, ABCA4, ABCD1, ABCD2, ABCC6, CFTR, ABCC2, ABCC1, ABCB1, ABCB4, ABCD3, ABCD4, ABCC8, TAP1, TAP2, ABCB11, ABCC3, ABCG2, ABCG1, ABCC5, ABCB6, ABCC9, ABCC4, ABCA7, ABCA10, ABCA9, ABCA8, ABCB8, ABCB10, ABCB9, ABCA6, ABCA5, ABCA12, ABCG4, ABCG5, ABCG8, ABCC11, ABCC10, ABCC12, ABCA13, ABCB5
KEGG	Acute Myeloid Leukemia	AKT1, AKT2, ARAF, BAD, CCND1, BRAF, RUNX1, RUNX1T1, CEBPA, CHUK, EIF4EBP1, FLT3, MTOR, GRB2, HRAS, IKBKB, JUP, KIT, KRAS, MYC, NFKB1, NRAS, PIK3CA, PIK3CB, PIM1, PIK3CD, PIK3CG, PIK3R1, PIK3R2, PML, PPARD, MAPK1, MAPK3, MAP2K1, MAP2K2, RAF1, RARA, RELA, RPS6KB1, RPS6KB2, SOS1, SOS2, SPI1, STAT3, STAT5A, STAT5B, TCF7, TCF7L2, ZBTB16, PIK3R3, IKBKG, CCNA1, AKT3, PIM2, PIK3R5, LEF1, TCF7L1, LOC652346, LOC652671, LOC652799
KEGG	Adherens Junction	ACP1, ACTB, ACTG1, ACTN4, ACTN1, ACTN2, ACTN3, RHOA, CDC42, CDH1, CREBBP, CSNK2A1, CSNK2A2, CSNK2B, CTNNA1, CTNNA2, CTNNB1, CTNND1, EGFR, EP300, ERBB2, FER, FGFR1, FYN, IGF1R, INSR, LMO7, SMAD2, SMAD3, SMAD4, MET, AFDN, MAPK1, MAPK3, PTPN1, PTPN6, PTPRB, PTPRF, PTPRJ, PTPRM, NECTIN1, NECTIN2, RAC1, RAC2, RAC3, SNAI2, SNAI1, SRC, MAP3K7, TCF7, TCF7L2, TGFBR1, TGFBR2, TJP1, VCL, WAS, YES1, IQGAP1, WASF1, WASL, FARP2, WASF2, BAIAP2, SORBS1, WASF3, NECTIN3, CTNNA3, LEF1, NLK, PARD3, NECTIN4, TCF7L1, SSX2IP, LOC646821, LOC100418883

Predefined gene sets in Hepamine

**Data source**	**Pathways**	**Symbols**
KEGG	Adipocytokine Signaling Pathway	ACACB, AGRP, AKT1, AKT2, CD36, CHUK, CPT1A, CPT1B, ACSL1, ACSL3, ACSL4, MTOR, G6PC, IKBKB, IRS1, JAK2, LEP, LEPR, NFKB1, NFKBIA, NFKBIB, NFKBIE, NPY, PCK1, PCK2, POMC, PPARA, PRKAA1, PRKAA2, PRKAB1, PRKAB2, PRKAG1, PRKCQ, MAPK8, MAPK9, MAPK10, PTPN11, RELA, RXRA, RXRB, RXRG, SLC2A1, SLC2A4, STAT3, STK11, TNF, TNFRSF1A, TNFRSF1B, TRAF2, IRS4, IKBKG, IRS2, TRADD, SOCS3, ADIPOQ, AKT3, CAMKK2, PPARGC1A, ACSL6, ADIPOR1, PRKAG2, ACSL5, PRKAG3, G6PC2, ADIPOR2, CAMKK1, CPT1C
KEGG	Alanine Aspartate And Glutamate Metabolism	ABAT, ADSL, ADSS, AGXT, ASL, ASNS, ASPA, ASS1, CAD, CPS1, GAD1, GAD2, GFPT1, GLS, GLUD1, GLUD2, GLUL, GOT1, GOT2, GPT, PPAT, ALDH5A1, DDO, ALDH4A1, GFPT2, GLS2, NIT2, AGXT2, GPT2, ACY3, ADSSL1, IL4I1
KEGG	Aldosterone Regulated Sodium Reabsorption	ATP1A1, ATP1A2, ATP1A3, ATP1A4, ATP1B1, ATP1B2, ATP1B3, FXYD2, SFN, HSD11B1, HSD11B2, IGF1, INS, INSR, IRS1, KCNJ1, KRAS, NR3C2, PDPK1, PIK3CA, PIK3CB, PIK3CD, PIK3CG, PIK3R1, PIK3R2, PRKCA, PRKCB, PRKCG, MAPK1, MAPK3, SCNN1A, SCNN1B, SCNN1G, SGK1, IRS4, PIK3R3, IRS2, SLC9A3R2, NEDD4L, ATP1B4, PIK3R5, FXYD4
KEGG	Allograft Rejection	FAS, FASLG, CD28, CD80, CD86, CD40, CD40LG, GZMB, HLA-A, HLA-B, HLA-C, HLA-DMA, HLA-DMB, HLA-DOA, HLA-DOB, HLA-DPA1, HLA-DPB1, HLA-DQA1, HLA-DQA2, HLA-DQB1, HLA-DRA, HLA-DRB1, HLA-DRB3, HLA-DRB4, HLA-DRB5, HLA-E, HLA-F, HLA-G, IFNG, IL2, IL4, IL5, IL10, IL12A, IL12B, PRF1, TNF, LOC652614
KEGG	Alpha Linolenic Acid Metabolism	ACOX1, PLA2G1B, PLA2G2A, PLA2G4A, PLA2G5, ACOX3, PLA2G6, PLA2G10, JMJD7-PLA2G4B, FADS2, PLA2G2D, PLA2G2E, PLA2G3, PLA2G2F, PLA2G12A, PLA2G12B, PLA2G4E, PLA2G2C, PLA2G4B

Predefined gene sets in Hepamine

Data source	Pathways	Symbols
KEGG	Alzheimers Disease	ADAM10, APAF1, APBB1, APOE, APP, FAS, ATP2A1, ATP2A2, ATP2A3, ATP5F1A, ATP5F1B, ATP5F1C, ATP5F1D, ATP5F1E, ATP5PB, ATP5MC1, ATP5MC2, ATP5MC3, ATP5PF, ATP5PO, BAD, BID, CACNA1C, CACNA1D, CACNA1F, CACNA1S, CALM1, CALM2, CALM3, CALML3, CAPN1, CAPN2, CASP3, CASP7, CASP8, CASP9, CDK5, COX4I1, COX5B, COX6A1, COX6A2, COX6B1, COX6C, COX7A1, COX7A2, COX7B, COX7C, COX8A, CYC1, ERN1, GAPDH, GNAQ, GRIN1, GRIN2A, GRIN2B, GRIN2C, GRIN2D, GSK3B, HSD17B10, IDE, IL1B, ITPR1, ITPR2, ITPR3, LPL, LRP1, MAPT, MME, ATP6, ATP8, COX1, COX2, COX3, CYTB, NDUFA1, NDUFA2, NDUFA3, NDUFA4, NDUFA5, NDUFA6, NDUFA7, NDUFA8, NDUFA9, NDUFA10, NDUFAB1, NDUFB1, NDUFB2, NDUFB3, NDUFB4, NDUFB5, NDUFB6, NDUFB7, NDUFB8, NDUFB9, NDUFB10, NDUFC1, NDUFC2, NDUFS1, NDUFS2, NDUFS3, NDUFV1, NDUFS4, NDUFS5, NDUFS6, NDUFS8, NDUFV2, NDUFV3, NOS1, PLCB2, PLCB3, PLCB4, PPP3CA, PPP3CB, PPP3CC, PPP3R1, PPP3R2, MAPK1, MAPK3, PSEN1, PSEN2, RYR3, SDHA, SDHB, SDHC, SDHD, SNCA, ADAM17, TNF, TNFRSF1A, UQCRB, UQCRC1, UQCRC2, UQCRFS1, UQCRH, FADD, CDK5R1, NAE1, COX7A2L, COX5A, EIF2AK3, ATP5PD, UQCR11, CHP1, ATF6, PLCB1, NCSTN, BACE1, BACE2, UQCRQ, UQCR10, APH1A, CALML5, CYCS, PSENEN, NDUFA4L2, CHP2, COX4I2, COX6B2, CALML6, COX7B2, COX8C, NDUFS7, ATP5MC1P5, UQCRHL, LOC642502, LOC644310, LOC727947, COX6CP3, LOC100133737
KEGG	Aminoacyl Trna Biosynthesis	AARS, CARS, DARS, EPRS, FARSA, GARS, HARS, IARS, KARS, MARS, NARS, QARS, RARS, SARS, TARS, VARS, WARS, YARS, FARSB, WARS2, FARS2, LARS2, HARS2, PARS2, YARS2, SEPSECS, LARS, SARS2, DARS2, IARS2, RARS2, VARS2, AARS2, CARS2, NARS2, TARS2, MARS2, PSTK, MTFMT, TARSL2, EARS2

Predefined gene sets in Hepamine

Data source	Pathways	Symbols
KEGG	Amino Sugar And Nucleotide Sugar Metabolism	CHIT1, CYB5R3, GALE, GALK1, GALK2, GALT, GCK, GFPT1, GMDS, GPI, HEXA, HEXB, HK1, HK2, HK3, MPI, PGM1, PGM3, PMM1, PMM2, RENBP, UAP1, TSTA3, UGDH, UGP2, FPGT, GFPT2, GNPDA1, GNE, CHIA, GMPPB, GMPPA, AMDHD2, CYB5R1, NANS, PGM2, NAGK, CMAS, GNPNAT1, UXS1, NPL, GNPDA2, NANP, FUK
KEGG	Amyotrophic Lateral Sclerosis Als	APAF1, BAD, BAX, BCL2, BCL2L1, BID, CASP1, CASP3, CASP9, CAT, MAPK14, DAXX, GPX1, GRIA1, GRIA2, GRIN1, GRIN2A, GRIN2B, GRIN2C, GRIN2D, MAP3K5, NEFM, NEFH, NEFL, NOS1, PPP3CA, PPP3CB, PPP3CC, PPP3R1, PPP3R2, MAPK11, MAPK13, MAP2K3, MAP2K6, PRPH, RAB5A, RAC1, PRPH2, MAPK12, SLC1A2, SOD1, TNF, TNFRSF1A, TNFRSF1B, TP53, CCS, TOMM40, CHP1, CYCS, ALS2, CHP2, DERL1, TOMM40L
KEGG	Antigen Processing And Presentation	B2M, CALR, CANX, CD4, CD8A, CD8B, CD74, CREB1, CTSB, CTSL, CTSS, PDIA3, HLA-A, HLA-B, HLA-C, HLA-DMA, HLA-DMB, HLA-DOA, HLA-DOB, HLA-DPA1, HLA-DPB1, HLA-DQA1, HLA-DQA2, HLA-DQB1, HLA-DRA, HLA-DRB1, HLA-DRB3, HLA-DRB4, HLA-DRB5, HLA-E, HLA-F, HLA-G, HSPA1A, HSPA1B, HSPA1L, HSPA2, HSPA4, HSPA5, HSPA6, HSPA8, HSP90AA1, HSP90AB1, IFNA1, IFNA2, IFNA4, IFNA5, IFNA6, IFNA7, IFNA8, IFNA10, IFNA13, IFNA14, IFNA16, IFNA17, IFNA21, KIR2DL1, KIR2DL2, KIR2DL3, KIR2DL4, KIR2DS1, KIR2DS3, KIR2DS4, KIR2DS5, KIR3DL1, KIR3DL2, KLRC1, KLRC2, KLRC3, KLRD1, LTA, CIITA, NFYA, NFYB, NFYC, LGMN, PSME1, PSME2, RFX5, RFXAP, TAP1, TAP2, TAPBP, KLRC4, RFXANK, PSME3, IFI30, KIR2DL5A, KIR3DL3, LOC652614

Predefined gene sets in Hepamine

Data source	Pathways	Symbols
KEGG	Apoptosis	AKT1, AKT2, APAF1, BIRC2, BIRC3, XIAP, FAS, FASLG, ATM, BAD, BAX, BCL2, BCL2L1, BID, CAPN1, CAPN2, CASP3, CASP6, CASP7, CASP8, CASP9, CASP10, CHUK, CSF2RB, DFFA, DFFB, ENDOG, IKBKB, IL1A, IL1B, IL1R1, IL1RAP, IL3, IL3RA, IRAK1, IRAK2, MYD88, NFKB1, NFKBIA, NGF, NTRK1, PIK3CA, PIK3CB, PIK3CD, PIK3CG, PIK3R1, PIK3R2, PPP3CA, PPP3CB, PPP3CC, PPP3R1, PPP3R2, PRKACA, PRKACB, PRKACG, PRKAR1A, PRKAR1B, PRKAR2A, PRKAR2B, PRKX, RELA, TNF, TNFRSF1A, TP53, TRAF2, PIK3R3, IKBKG, TRADD, RIPK1, TNFSF10, FADD, TNFRSF10D, TNFRSF10C, TNFRSF10B, TNFRSF10A, CFLAR, MAP3K14, AIFM1, EXOG, AKT3, IRAK3, CHP1, ENDOD1, PIK3R5, IRAK4, CYCS, CHP2, LOC651610
KEGG	Arachidonic Acid Metabolism	ALOX12, ALOX5, ALOX12B, ALOX15, ALOX15B, CBR1, CBR3, CYP2B6, CYP2C19, CYP2C8, CYP2C9, CYP2C18, CYP2E1, CYP2J2, CYP4A11, EPHX2, GGT1, GGT7, GGT5, GPX1, GPX2, GPX3, GPX4, GPX5, GPX7, LTA4H, CYP4F3, LTC4S, PLA2G1B, PLA2G2A, PLA2G4A, PLA2G5, PTGDS, PTGIS, PTGS1, PTGS2, TBXAS1, PLA2G6, PLA2G10, CYP4F2, AKR1C3, JMJD7-PLA2G4B, PTGES, PLA2G2D, HPGDS, PLA2G2E, PLA2G3, PLA2G2F, PTGES2, PLA2G12A, PLA2G12B, CYP2U1, PLA2G4E, GGT6, GPX6, CYP4A22, PLA2G2C, PLA2G4B
KEGG	Arginine And Proline Metabolism	AOC1, ACY1, ALDH2, ALDH1B1, ALDH9A1, ALDH3A2, AMD1, ARG1, ARG2, ASL, ASS1, ALDH7A1, CKB, CKM, CKMT1B, CKMT2, CPS1, DAO, GAMT, GATM, GLS, GLUD1, GLUD2, GLUL, GOT1, GOT2, MAOA, MAOB, NOS1, NOS2, NOS3, OAT, ODC1, OTC, P4HA1, PRODH, PYCR1, ALDH18A1, SAT1, SMS, SRM, ALDH4A1, P4HA2, GLS2, PYCR2, LAP3, PRODH2, PYCR3, AGMAT, SAT2, AZIN2, NAGS, P4HA3, CKMT1A

Predefined gene sets in Hepamine

Data source	Pathways	Symbols
KEGG	Arrhythmogenic Right Ventricular Cardiomyopathy Arvc	ACTB, ACTG1, ACTN4, ACTN1, ACTN2, ACTN3, ATP2A2, CACNA1C, CACNA1D, CACNA1F, CACNA1S, CACNA2D1, CACNB1, CACNB2, CACNB3, CACNB4, CACNG1, CDH2, CTNNA1, CTNNA2, CTNNB1, DAG1, DES, DMD, DSC2, DSG2, DSP, EMD, GJA1, ITGA6, ITGA1, ITGA2, ITGA2B, ITGA3, ITGA4, ITGA5, ITGA7, ITGA9, ITGAV, ITGB1, ITGB3, ITGB4, ITGB5, ITGB6, ITGB7, ITGB8, JUP, LAMA2, LMNA, PKP2, RYR2, SGCA, SGCB, SGCD, SGCG, SLC8A1, TCF7, TCF7L2, ITGA10, ITGA8, CACNA2D2, CACNG3, CACNG2, ITGA11, CACNG5, CACNG4, CTNNA3, LEF1, CACNA2D3, CACNG8, CACNG7, CACNG6, TCF7L1, CACNA2D4, LOC646821, LOC100418883
KEGG	Ascorbate And Aldarate Metabolism	ALDH2, ALDH1B1, ALDH9A1, ALDH3A2, ALDH7A1, UGDH, UGT2B4, UGT2B7, UGT2B10, UGT2B15, UGT2B17, UGT2B11, UGT2A1, UGT2B28, UGT1A10, UGT1A8, UGT1A7, UGT1A6, UGT1A5, UGT1A9, UGT1A4, UGT1A1, UGT1A3, MIOX, UGT2A3
KEGG	Asthma	CD40, CD40LG, FCER1A, MS4A2, FCER1G, HLA-DMA, HLA-DMB, HLA-DOA, HLA-DOB, HLA-DPA1, HLA-DPB1, HLA-DQA1, HLA-DQA2, HLA-DQB1, HLA-DRA, HLA-DRB1, HLA-DRB3, HLA-DRB4, HLA-DRB5, IL3, IL4, IL5, IL9, IL10, IL13, PRG2, RNASE3, CCL11, TNF, EPX
KEGG	Autoimmune Thyroid Disease	FAS, FASLG, CD28, CD80, CD86, CD40, CD40LG, CGA, CTLA4, GZMB, HLA-A, HLA-B, HLA-C, HLA-DMA, HLA-DMB, HLA-DOA, HLA-DOB, HLA-DPA1, HLA-DPB1, HLA-DQA1, HLA-DQA2, HLA-DQB1, HLA-DRA, HLA-DRB1, HLA-DRB3, HLA-DRB4, HLA-DRB5, HLA-E, HLA-F, HLA-G, IFNA1, IFNA2, IFNA4, IFNA5, IFNA6, IFNA7, IFNA8, IFNA10, IFNA13, IFNA14, IFNA16, IFNA17, IFNA21, IL2, IL4, IL5, IL10, PRF1, TG, TPO, TSHB, TSHR, LOC652614

Predefined gene sets in Hepamine

Data source	Pathways	Symbols
KEGG	Axon Guidance	ABL1, RHOA, CDC42, CDK5, CFL1, CFL2, DCC, DPYSL2, EFNA1, EFNA2, EFNA3, EFNA4, EFNA5, EFNB1, EFNB2, EFNB3, EPHA2, EPHA1, EPHA3, EPHA4, EPHA5, EPHA7, EPHA8, EPHB1, EPHB2, EPHB3, EPHB4, EPHB6, FES, FYN, GNAI1, GNAI2, GNAI3, GSK3B, HRAS, ITGB1, KRAS, L1CAM, ABLIM1, LIMK1, LIMK2, MET, NCK1, NFATC1, NFATC2, NFATC3, NFATC4, NRAS, NTN3, PAK1, PAK2, PAK3, PLXNA1, PLXNA2, PLXNB1, PLXNB3, PPP3CA, PPP3CB, PPP3CC, PPP3R1, PPP3R2, MAPK1, MAPK3, PTK2, RAC1, RAC2, RAC3, RASA1, RGS3, ROBO1, ROBO2, ROCK1, CXCL12, SEMA3F, SLIT1, SLIT3, CXCR4, SEMA3B, NCK2, SEMA7A, UNC5C, NRP1, SEMA5A, SLIT2, NTN1, ROCK2, SEMA3E, SRGAP3, PLXNC1, PAK4, SEMA3A, SEMA6C, SEMA6B, SEMA4F, SEMA4D, SEMA4B, SEMA3C, NFAT5, CHP1, NTNG1, ABLIM3, ARHGEF12, SRGAP2, PLXNB2, NGEF, RND1, RHOD, SEMA5B, SEMA4C, PLXNA3, DPYSL5, SEMA3G, PAK6, PAK5, SRGAP1, SEMA6A, LRRC4C, SEMA4G, NTN4, CHP2, SEMA4A, ROBO3, SEMA6D, ABLIM2, UNC5A, UNC5D, UNC5B, SEMA3D, EPHA6
KEGG	Basal Cell Carcinoma	APC, BMP2, BMP4, CTNNB1, DVL1, DVL2, DVL3, FZD2, GLI1, GLI2, GLI3, GSK3B, PTCH1, SHH, SMO, TCF7, TCF7L2, TP53, WNT1, WNT2, WNT3, WNT5A, WNT6, WNT7A, WNT7B, WNT8A, WNT8B, WNT10B, WNT11, WNT2B, WNT9A, WNT9B, FZD5, FZD3, AXIN1, AXIN2, FZD1, FZD4, FZD6, FZD7, FZD8, FZD9, PTCH2, APC2, FZD10, STK36, LEF1, WNT16, SUFU, WNT4, HHIP, WNT10A, WNT5B, TCF7L1, WNT3A
KEGG	Basal Transcription Factors	GTF2A1, GTF2A2, GTF2B, GTF2E1, GTF2E2, GTF2F1, GTF2F2, GTF2H1, GTF2H2, GTF2H3, GTF2H4, GTF2I, TAF1, TAF2, TAF4, TAF4B, TAF5, TAF6, TAF7, TAF9, TAF10, TAF11, TAF12, TAF13, TBP, TBPL1, GTF2IRD1, TAF6L, GTF2A1L, STON1, TAF5L, TAF9B, TAF7L, TAF1L, TBPL2, LOC391764

Predefined gene sets in Hepamine

Data source	Pathways	Symbols
KEGG	Base Excision Repair	PARP1, PARP4, APEX1, FEN1, HMGB1, LIG1, LIG3, MPG, MUTYH, NTHL1, OGG1, PCNA, POLB, POLD1, POLD2, POLE, POLE2, TDG, UNG, XRCC1, MBD4, PARP2, PARP3, HMGB1P1, POLD3, SMUG1, APEX2, POLL, POLE3, NEIL3, POLE4, POLD4, NEIL1, NEIL2, HMGB1P40
KEGG	B Cell Receptor Signaling Pathway	AKT1, AKT2, BTK, CD19, CD22, CD72, CD79A, CD79B, CD81, CHUK, CR2, FCGR2B, FOS, GRB2, GSK3B, HRAS, IKBKB, INPP5D, JUN, KRAS, LYN, NFATC1, NFATC2, NFATC3, NFATC4, NFKB1, NFKBIA, NFKBIB, NFKBIE, NRAS, PIK3CA, PIK3CB, PIK3CD, PIK3CG, PIK3R1, PIK3R2, PLCG2, PPP3CA, PPP3CB, PPP3CC, PPP3R1, PPP3R2, PRKCB, MAPK1, MAPK3, MAP2K1, MAP2K2, PTPN6, RAC1, RAC2, RAC3, RAF1, RELA, SOS1, SOS2, SYK, VAV1, VAV2, PIK3R3, IKBKG, IFITM1, BCL10, AKT3, VAV3, NFAT5, MALT1, LILRB3, CHP1, PIK3R5, RASGRP3, DAPP1, BLNK, CHP2, CARD11, PIK3AP1
KEGG	Beta Alanine Metabolism	ABAT, ACADM, ALDH2, ALDH1B1, ALDH9A1, ALDH3A2, AOC2, ALDH7A1, DPYD, DPYS, ECHS1, EHHADH, GAD1, GAD2, HADHA, SMS, SRM, AOC3, MLYCD, HIBCH, UPB1, CNDP1
KEGG	Biosynthesis Of Unsaturated Fatty Acids	ACAA1, ACOX1, BAAT, HADHA, FADS1, SCD, ACOX3, HACD1, FADS2, TECR, ACOT2, ACOT7, HSD17B12, ELOVL2, YOD1, PECR, ELOVL5, ELOVL6, SCD5, ACOT4, HACD2, ACOT1
KEGG	Bladder Cancer	ARAF, CCND1, BRAF, CDH1, CDK4, CDKN1A, CDKN2A, DAPK1, DAPK3, E2F1, E2F2, E2F3, TYMP, EGF, EGFR, ERBB2, FGFR3, VEGFD, HRAS, CXCL8, KRAS, MDM2, MMP1, MMP2, MMP9, MYC, NRAS, PGF, MAPK1, MAPK3, MAP2K1, MAP2K2, RAF1, RB1, THBS1, TP53, VEGFA, VEGFB, VEGFC, RPS6KA5, RASSF1, DAPK2

Predefined gene sets in Hepamine

Data source	Pathways	Symbols
KEGG	Butanoate Metabolism	ABAT, ACADS, ACAT1, ACAT2, ALDH2, ALDH1B1, ALDH9A1, ALDH3A2, ALDH7A1, BDH1, ECHS1, EHHADH, GAD1, GAD2, HADHA, HADH, HMGCL, HMGCS1, HMGCS2, OXCT1, PDHA1, PDHA2, PDHB, ACSM3, ALDH5A1, ACSM5, BDH2, AKR1B10, OXCT2, AACS, L2HGDH, ACSM1, ACSM2A, ACSM4
KEGG	Calcium Signaling Pathway	ADCY1, ADCY2, ADCY3, ADCY7, ADCY8, ADCY9, ADORA2A, ADORA2B, ADRA1D, ADRA1B, ADRA1A, ADRB1, ADRB2, ADRB3, AGTR1, SLC25A4, SLC25A5, SLC25A6, ATP2A1, ATP2A2, ATP2A3, ATP2B1, ATP2B2, ATP2B3, ATP2B4, AVPR1A, AVPR1B, BDKRB1, BDKRB2, BST1, CACNA1A, CACNA1B, CACNA1C, CACNA1D, CACNA1E, CACNA1F, CACNA1S, CALM1, CALM2, CALM3, CALML3, CAMK4, CAMK2A, CAMK2B, CAMK2D, CAMK2G, CCKAR, CCKBR, CD38, CHRM1, CHRM2, CHRM3, CHRM5, CHRNA7, DRD1, DRD5, EDNRA, EDNRB, EGFR, ERBB2, ERBB3, ERBB4, F2R, PTK2B, GNA11, GNA15, GNAL, GNAQ, GNAS, GRIN1, GRIN2A, GRIN2C, GRIN2D, GRM1, GRM5, GRPR, HRH1, HRH2, HTR2A, HTR2B, HTR2C, HTR4, HTR5A, HTR6, HTR7, ITPKA, ITPKB, ITPR1, ITPR2, ITPR3, LHCGR, MYLK, NOS1, NOS2, NOS3, NTSR1, OXTR, P2RX1, P2RX3, P2RX4, P2RX5, P2RX7, PDE1A, PDE1C, PDE1B, PDGFRA, PDGFRB, PHKA1, PHKA2, PHKB, PHKG1, PHKG2, PLCB2, PLCB3, PLCB4, PLCD1, PLCG1, PLCG2, PLN, PPID, PPP3CA, PPP3CB, PPP3CC, PPP3R1, PPP3R2, PRKACA, PRKACB, PRKACG, PRKCA, PRKCB, PRKCG, PRKX, PTAFR, PTGER1, PTGER3, PTGFR, RYR1, RYR2, RYR3, SLC8A2, SLC8A1, SLC8A3, TACR2, TACR1, TACR3, TBXA2R, TNNC2, TNNC1, TRHR, TRPC1, VDAC1, VDAC2, VDAC3, SPHK1, CACNA1I, CACNA1H, CACNA1G, P2RX6, GNA14, CYSLTR1, CHP1, P2RX2, PLCB1, PLCE1, CALML5, LTB4R2, SPHK2, CYSLTR2, CHP2, SLC25A31, PLCD4, MYLK2, PLCZ1, MYLK3, PLCD3, CALML6, ADCY4, LOC729317

Predefined gene sets in Hepamine

Data source	Pathways	Symbols
KEGG	Cardiac Muscle Contraction	ACTC1, ATP1A1, ATP1A2, ATP1A3, ATP1A4, ATP1B1, ATP1B2, ATP1B3, FXYD2, ATP2A2, CACNA1C, CACNA1D, CACNA1F, CACNA1S, CACNA2D1, CACNB1, CACNB2, CACNB3, CACNB4, CACNG1, COX4I1, COX5B, COX6A1, COX6A2, COX6B1, COX6C, COX7A1, COX7A2, COX7B, COX7C, COX8A, CYC1, COX1, COX2, COX3, CYTB, MYH6, MYH7, MYL2, MYL3, RYR2, SLC8A1, SLC9A1, TNNC1, TNNI3, TNNT2, TPM1, TPM2, TPM3, TPM4, UQCRB, UQCRC1, UQCRC2, UQCRFS1, UQCRH, COX7A2L, CACNA2D2, COX5A, CACNG3, CACNG2, SLC9A6, UQCR11, ATP1B4, UQCRQ, CACNG5, CACNG4, UQCR10, CACNA2D3, CACNG8, CACNG7, CACNG6, COX4I2, CACNA2D4, COX6B2, COX7B2, COX8C, UQCRHL, LOC644310, LOC727947, COX6CP3

Predefined gene sets in Hepamine

Data source	Pathways	Symbols
KEGG	Cell Adhesion Molecules Cams	ALCAM, CD2, CD4, CD6, CD8A, CD8B, CD22, CD28, CD80, CD86, CD34, CD40, CD40LG, CD58, CDH1, CDH2, CDH3, CDH4, CDH5, CDH15, CNTN1, CLDN4, CLDN3, CLDN7, VCAN, CTLA4, GLG1, HLA-A, HLA-B, HLA-C, HLA-DMA, HLA-DMB, HLA-DOA, HLA-DOB, HLA-DPA1, HLA-DPB1, HLA-DQA1, HLA-DQA2, HLA-DQB1, HLA-DRA, HLA-DRB1, HLA-DRB3, HLA-DRB4, HLA-DRB5, HLA-E, HLA-F, HLA-G, ICAM1, ICAM2, ICAM3, ITGA6, ITGA4, ITGA9, ITGAL, ITGAM, ITGAV, ITGB1, ITGB2, ITGB7, ITGB8, L1CAM, MAG, CD99, MPZ, NCAM1, NCAM2, NEO1, NRCAM, CLDN11, PDCD1, PECAM1, PTPRC, PTPRF, PTPRM, PVR, NECTIN1, NECTIN2, SDC1, SDC2, SDC4, SELE, SELL, SELP, SELPLG, SIGLEC1, SPN, CNTN2, CLDN5, VCAM1, MADCAM1, CNTNAP1, ITGA8, MPZL1, CLDN10, CLDN8, CLDN6, CLDN2, CLDN1, CLDN9, NRXN3, NRXN1, NRXN2, SDC3, CD226, CLDN16, NLGN1, NFASC, ICOSLG, CLDN14, CADM1, CLDN15, NECTIN3, CNTNAP2, CLDN17, CD274, ICOS, CLDN20, F11R, CLDN18, CLDN22, NLGN3, NLGN4X, NLGN2, CADM3, JAM2, PDCD1LG2, CD276, JAM3, ESAM, CLDN23, CLDN19, NEGR1, LOC652614, OCLN

Predefined gene sets in Hepamine

Data source	Pathways	Symbols
KEGG	Cell Cycle	ABL1, ATM, ATR, CCND1, BUB1, BUB1B, CCNA2, CCNB1, CCND2, CCND3, CCNE1, CCNH, CDK1, CDC6, CDC20, CDC25A, CDC25B, CDC25C, CDC27, CDK2, CDK4, CDK6, CDK7, CDKN1A, CDKN1B, CDKN1C, CDKN2A, CDKN2B, CDKN2C, CDKN2D, CHEK1, CREBBP, GADD45A, E2F1, E2F2, E2F3, E2F4, E2F5, EP300, SFN, GSK3B, HDAC1, HDAC2, MAD2L1, SMAD2, SMAD3, SMAD4, MCM2, MCM3, MCM4, MCM5, MCM6, MCM7, MDM2, MYC, GADD45B, ORC1, ORC2, ORC4, ORC5, PCNA, PLK1, PRKDC, RAD21, RB1, RBL1, RBL2, SKP1, SKP2, TFDP1, TFDP2, TGFB1, TGFB2, TGFB3, TP53, TTK, WEE1, YWHAB, YWHAE, YWHAG, YWHAH, YWHAZ, ZBTB17, SMC1A, CDC7, CDC45, MAD1L1, CUL1, CDC14B, CDC14A, CDC23, CDC16, CCNA1, PKMYT1, SMC3, CCNB2, CCNE2, BUB3, PTTG1, ESPL1, RBX1, STAG1, ANAPC10, MAD2L2, STAG2, PTTG2, GADD45G, DBF4, YWHAQ, CHEK2, ORC6, ORC3, ANAPC13, SMC1B, ANAPC2, ANAPC4, FZR1, ANAPC5, ANAPC7, ANAPC11, ANAPC1, CCNB3, CDC26, WEE2, LOC650621, LOC651610, SKP1P2, LOC731751

Predefined gene sets in Hepamine

Data source	Pathways	Symbols
KEGG	Chemokine Signaling Pathway	ADCY1, ADCY2, ADCY3, ADCY5, ADCY6, ADCY7, ADCY8, ADCY9, GRK2, GRK3, AKT1, AKT2, RHOA, ARRB1, ARRB2, CXCR5, BRAF, CDC42, CHUK, CCR1, CCR3, CCR4, CCR5, CCR6, CCR7, CCR8, CRK, CRKL, CSK, CX3CR1, DOCK2, PTK2B, FGR, FOXO3, GNAI1, GNAI2, GNAI3, GNB1, GNB2, GNB3, GNG3, GNG4, GNG5, GNG7, GNG10, GNG11, GNGT1, GNGT2, CCR10, XCR1, CXCR3, GRK4, GRK5, GRK6, GRB2, CXCL1, CXCL2, CXCL3, GSK3A, GSK3B, HCK, HRAS, IKBKB, CXCL8, CXCR1, CXCR2, CXCL10, ITK, JAK2, JAK3, KRAS, LYN, CXCL9, NFKB1, NFKBIA, NFKBIB, NRAS, PAK1, PF4, PF4V1, PIK3CA, PIK3CB, PIK3CD, PIK3CG, PIK3R1, PIK3R2, PLCB2, PLCB3, PLCB4, PPBP, PRKACA, PRKACB, PRKACG, PRKCB, PRKCD, PRKCZ, MAPK1, MAPK3, MAP2K1, PRKX, PTK2, PXN, RAC1, RAC2, RAF1, RAP1A, RAP1B, RELA, GRK1, ROCK1, CCL1, CCL2, CCL3, CCL3L1, CCL4, CCL5, CCL7, CCL8, CCL11, CCL13, CCL14, CCL15, CCL16, CCL17, CCL18, CCL19, CCL20, CCL21, CCL22, CCL23, CCL24, CCL25, CXCL6, CXCL11, CXCL5, XCL1, CX3CL1, CXCL12, SHC1, SOS1, SOS2, STAT1, STAT2, STAT3, STAT5B, XCL2, TIAM1, VAV1, VAV2, WAS, CXCR4, PIK3R3, IKBKG, WASL, ROCK2, CXCL14, CCL4L2, BCAR1, ELMO1, AKT3, RASGRP2, CCL26, VAV3, CXCL13, CXCR6, GNB5, CCR9, CCL27, PLCB1, PIK3R5, SHC2, TIAM2, GNG13, SHC3, GNG2, GNG12, PARD3, CCL28, PREX1, CXCL16, GNB4, GNG8, GRK7, ADCY4, CCL4L1, SHC4, CCL3L3, NCF1, PPBPP1, CCR2

Predefined gene sets in Hepamine

Data source	Pathways	Symbols
KEGG	Chronic Myeloid Leukemia	ABL1, AKT1, AKT2, ARAF, BAD, CCND1, BCL2L1, BCR, BRAF, RUNX1, CBL, CBLB, CDK4, CDK6, CDKN1A, CDKN1B, CDKN2A, CHUK, CRK, CRKL, CTBP1, CTBP2, E2F1, E2F2, E2F3, MECOM, GRB2, HDAC1, HDAC2, HRAS, IKBKB, KRAS, SMAD3, SMAD4, MDM2, MYC, NFKB1, NFKBIA, NRAS, PIK3CA, PIK3CB, PIK3CD, PIK3CG, PIK3R1, PIK3R2, MAPK1, MAPK3, MAP2K1, MAP2K2, PTPN11, RAF1, RB1, RELA, SHC1, SOS1, SOS2, STAT5A, STAT5B, TGFB1, TGFB2, TGFB3, TGFBR1, TGFBR2, TP53, PIK3R3, IKBKG, GAB2, AKT3, PIK3R5, CBLC, SHC2, SHC3, SHC4
KEGG	Circadian Rhythm Mammal	ARNTL, CRY1, CRY2, CSNK1D, CSNK1E, NPAS2, PER1, BHLHE40, PER3, PER2, NR1D1, CLOCK, BHLHE41
KEGG	Citrate Cycle Tca Cycle	ACLY, ACO1, ACO2, CS, DLAT, DLD, DLST, FH, IDH1, IDH2, IDH3A, IDH3B, IDH3G, MDH1, MDH2, OGDH, PC, PCK1, PCK2, PDHA1, PDHA2, PDHB, SDHA, SDHB, SDHC, SDHD, SUCLG2, SUCLG1, SUCLA2, OGDHL, SUCLG2P2, LOC642502
KEGG	Colorectal Cancer	AKT1, AKT2, APC, BIRC5, ARAF, RHOA, BAD, BAX, CCND1, BCL2, BRAF, CASP3, CASP9, CTNNB1, DCC, FOS, GSK3B, MSH6, JUN, KRAS, SMAD2, SMAD3, SMAD4, MLH1, MSH2, MSH3, MYC, PIK3CA, PIK3CB, PIK3CD, PIK3CG, PIK3R1, PIK3R2, MAPK1, MAPK3, MAPK8, MAPK9, MAPK10, MAP2K1, RAC1, RAC2, RAC3, RAF1, RALGDS, TCF7, TCF7L2, TGFB1, TGFB2, TGFB3, TGFBR1, TGFBR2, TP53, AXIN1, AXIN2, PIK3R3, AKT3, APC2, PIK3R5, APPL1, LEF1, CYCS, TCF7L1

Predefined gene sets in Hepamine

Data source	Pathways	Symbols
KEGG	Complement And Coagulation Cascades	A2M, SERPINC1, BDKRB1, BDKRB2, CFB, SERPING1, C1QA, C1QB, C1QC, C1R, C1S, C2, C3, C3AR1, C4A, C4B, C4BPA, C4BPB, C5, C5AR1, C6, C7, C8A, C8B, C8G, C9, CD59, CPB2, CR1, CR2, CD55, CFD, F2, F2R, F3, F5, F7, F8, F9, F10, F11, F12, F13A1, F13B, FGA, FGB, FGG, SERPIND1, CFH, CFI, KLKB1, KNG1, MBL2, CD46, SERPINE1, SERPINA5, SERPINA1, PLAT, PLAU, PLAUR, PLG, SERPINF2, PROC, PROS1, MASP1, TFPI, THBD, VWF, MASP2
KEGG	Cysteine And Methionine Metabolism	AHCY, AMD1, BHMT, CBS, CDO1, CTH, DNMT1, TRDMT1, DNMT3A, DNMT3B, GOT1, GOT2, LDHA, LDHB, LDHC, MAT1A, MAT2A, MPST, MTAP, MTR, SMS, SRM, TAT, AHCYL1, SDS, AHCYL2, MAT2B, DNMT3L, APIP, ADI1, ENOPH1, LDHAL6B, LDHAL6A, IL4I1

Predefined gene sets in Hepamine

Data source	Pathways	Symbols
KEGG	Cytokine Cytokine Receptor Interaction	ACVR1, ACVR1B, ACVR2A, ACVR2B, ACVRL1, AMH, AMHR2, FAS, FASLG, TNFRSF17, CXCR5, BMP2, BMP7, BMPR1A, BMPR1B, BMPR2, CD27, TNFRSF8, TNFSF8, CD40, CD40LG, CD70, CCR1, CCR3, CCR4, CCR5, CCR6, CCR7, CCR8, CNTF, CNTFR, CSF1, CSF1R, CSF2, CSF2RA, CSF2RB, CSF3, CSF3R, CTF1, CX3CR1, EDA, EGF, EGFR, EPO, EPOR, VEGFD, FLT1, FLT3, FLT3LG, FLT4, GH1, GH2, GHR, CCR10, XCR1, CXCR3, CXCL1, CXCL2, CXCL3, HGF, IFNA1, IFNA2, IFNA4, IFNA5, IFNA6, IFNA7, IFNA8, IFNA10, IFNA13, IFNA14, IFNA16, IFNA17, IFNA21, IFNAR1, IFNAR2, IFNB1, IFNG, IFNGR1, IFNGR2, IFNW1, IL1A, IL1B, IL1R1, IL1RAP, IL2, IL2RA, IL2RB, IL2RG, IL3, IL3RA, IL4, IL4R, IL5, IL5RA, IL6, IL6R, IL6ST, IL7, IL7R, CXCL8, CXCR1, IL9, CXCR2, IL9R, IL10, IL10RA, IL10RB, IL11, IL11RA, IL12A, IL12B, IL12RB1, IL12RB2, IL13, IL13RA1, IL15, IL15RA, TNFRSF9, IL17A, IL18, INHBA, INHBB, INHBC, CXCL10, KDR, KIT, LEP, LEPR, LIF, LIFR, LTA, LTB, LTBR, MET, KITLG, CXCL9, MPL, NGFR, TNFRSF11B, OSM, PDGFA, PDGFB, PDGFRA, PDGFRB, PF4, PF4V1, PPBP, PRL, PRLR, CCL1, CCL2, CCL3, CCL3L1, CCL4, CCL5, CCL7, CCL8, CCL11, CCL13, CCL14, CCL15, CCL16, CCL17, CCL18, CCL19, CCL20, CCL21, CCL22, CCL23, CCL24, CCL25, CXCL6, CXCL11, CXCL5, XCL1, CX3CL1, CXCL12, XCL2, TGFB1, TGFB2, TGFB3, TGFBR1, TGFBR2, TNF, TNFRSF1A, TNFRSF1B, TPO, TNFSF4, TNFRSF4, VEGFA, VEGFB, VEGFC, IL1R2, CXCR4, GDF5, TNFSF11, TNFRSF25, TNFSF14, TNFSF13, TNFSF12, TNFSF10, TNFSF9, TNFRSF14, TNFRSF6B, TNFRSF18, TNFRSF11A, TNFRSF10D, TNFRSF10C, TNFRSF10B, TNFRSF10A, IL18RAP, IL18R1, TNFSF18, OSMR, CXCL14, CCL4L2, TNFSF15, CCL26, CXCL13, CXCR6, TNFSF13B, CCR9, CCL27, EDAR, IL24, TNFRSF13B, CLCF1, IL17RA, IL17B, TNFRSF21, IL19, IL20, IL21R, IL22, TNFRSF12A, IL23A, IL20RA, IL20RB, TNFRSF19, IL17RB, IL26, PDGFC, CCL28, IFNK, CXCL16, IL22RA1, IL21, EDA2R, CRLF2, IL25, PLEKHO2, INHBE, RELT, TSLP, TNFRSF13C, IL22RA2, IL23R, IFNLR1, IFNL2, IFNL3, IFNL1, IFNE, CCL4L1, CCL3L3, LOC652799, PPBPP1, CCR2

Predefined gene sets in Hepamine

Data source	Pathways	Symbols
KEGG	Cytosolic Dna Sensing Pathway	ADAR, POLR3D, CASP1, CHUK, IFNA1, IFNA2, IFNA4, IFNA5, IFNA6, IFNA7, IFNA8, IFNA10, IFNA13, IFNA14, IFNA16, IFNA17, IFNA21, IFNB1, IKBKB, IL1B, IL6, IL18, CXCL10, IRF3, IRF7, NFKB1, NFKBIA, NFKBIB, RELA, CCL4, CCL5, IKBKG, RIPK1, AIM2, POLR1C, CCL4L2, IKBKE, POLR3F, POLR3G, POLR3C, RIPK3, POLR3A, TREX1, DDX58, PYCARD, TBK1, POLR1D, POLR3K, POLR3B, MAVS, ZBP1, POLR3GL, IL33, POLR3H, TMEM173, CCL4L1
KEGG	Dilated Cardiomyopathy	ACTB, ACTC1, ACTG1, ADCY1, ADCY2, ADCY3, ADCY5, ADCY6, ADCY7, ADCY8, ADCY9, ADRB1, ATP2A2, CACNA1C, CACNA1D, CACNA1F, CACNA1S, CACNA2D1, CACNB1, CACNB2, CACNB3, CACNB4, CACNG1, DAG1, DES, DMD, EMD, GNAS, IGF1, ITGA6, ITGA1, ITGA2, ITGA2B, ITGA3, ITGA4, ITGA5, ITGA7, ITGA9, ITGAV, ITGB1, ITGB3, ITGB4, ITGB5, ITGB6, ITGB7, ITGB8, LAMA2, LMNA, MYBPC3, MYH6, MYH7, MYL2, MYL3, PLN, PRKACA, PRKACB, PRKACG, PRKX, RYR2, SGCA, SGCB, SGCD, SGCG, SLC8A1, TGFB1, TGFB2, TGFB3, TNF, TNNC1, TNNI3, TNNT2, TPM1, TPM2, TPM3, TPM4, TTN, ITGA10, ITGA8, CACNA2D2, CACNG3, CACNG2, ITGA11, CACNG5, CACNG4, CACNA2D3, CACNG8, CACNG7, CACNG6, CACNA2D4, ADCY4, LOC646821, LOC100418883
KEGG	Dna Replication	DNA2, FEN1, LIG1, MCM2, MCM3, MCM4, MCM5, MCM6, MCM7, PCNA, POLA1, POLD1, POLD2, POLE, POLE2, PRIM1, PRIM2, RFC1, RFC2, RFC3, RFC4, RFC5, RPA1, RPA2, RPA3, SSBP1, RNASEH2A, POLD3, POLA2, RPA4, POLE3, POLE4, POLD4, RNASEH2B, RNASEH2C, RNASEH1
KEGG	Dorso Ventral Axis Formation	EGFR, ETS1, ETS2, ETV6, GRB2, KRAS, NOTCH1, NOTCH2, NOTCH3, NOTCH4, MAPK1, MAPK3, MAP2K1, SOS1, SOS2, PIWIL1, ETV7, PIWIL2, FMN2, SPIRE1, CPEB1, SPIRE2, PIWIL4, PIWIL3, LOC652554

Predefined gene sets in Hepamine

Data source	Pathways	Symbols
KEGG	Drug Metabolism Cytochrome P450	ADH1A, ADH1B, ADH1C, ADH4, ADH5, ADH6, ADH7, ALDH3A1, ALDH1A3, ALDH3B1, ALDH3B2, AOX1, CYP1A2, CYP2A6, CYP2A7, CYP3A7, CYP2A13, CYP2B6, CYP2C19, CYP2C8, CYP2C9, CYP2C18, CYP2D6, CYP2E1, CYP3A4, CYP3A5, FMO1, FMO2, FMO3, FMO4, FMO5, GSTA1, GSTA2, GSTA3, GSTA4, GSTM1, GSTM2, GSTM3, GSTM4, GSTM5, GSTP1, GSTT1, GSTT2, GSTZ1, MAOA, MAOB, MGST1, MGST2, MGST3, UGT2B4, UGT2B7, UGT2B10, UGT2B15, UGT2B17, GSTO1, UGT2B11, UGT2A1, UGT2B28, UGT1A10, UGT1A8, UGT1A7, UGT1A6, UGT1A5, UGT1A9, UGT1A4, UGT1A1, UGT1A3, CYP3A43, UGT2A3, GSTO2, GSTA5, GSTK1
KEGG	Drug Metabolism Other Enzymes	NAT1, NAT2, CDA, CES1, CYP2A6, CYP2A7, CYP3A7, CYP2A13, CYP3A4, CYP3A5, DPYD, DPYS, TYMP, GUSB, HPRT1, IMPDH1, IMPDH2, ITPA, TK1, TK2, TPMT, UGT2B4, UGT2B7, UGT2B10, UGT2B15, UGT2B17, UCK2, UMPS, UPP1, XDH, CES2, GMPS, UGT2B11, UGT2A1, UPB1, UGT2B28, UGT1A10, UGT1A8, UGT1A7, UGT1A6, UGT1A5, UGT1A9, UGT1A4, UGT1A1, UGT1A3, UCKL1, CYP3A43, UGT2A3, UCK1, UPP2, CES5A
KEGG	Ecm Receptor Interaction	CD36, CD44, CD47, CHAD, COL1A1, COL1A2, COL2A1, COL3A1, COL4A1, COL4A2, COL4A4, COL4A6, COL5A1, COL5A2, COL6A1, COL6A2, COL6A3, COL11A1, COL11A2, COMP, DAG1, FN1, GP1BA, GP1BB, GP5, GP9, HMMR, HSPG2, TNC, IBSP, ITGA6, ITGA1, ITGA2, ITGA2B, ITGA3, ITGA4, ITGA5, ITGA7, ITGA9, ITGAV, ITGB1, ITGB3, ITGB4, ITGB5, ITGB6, ITGB7, ITGB8, LAMA2, LAMA3, LAMA4, LAMA5, LAMB1, LAMB2, LAMB3, LAMC1, LAMC2, RELN, SDC1, SDC2, SDC4, SPP1, THBS1, THBS2, THBS3, THBS4, TNR, TNXB, VTN, VWF, ITGA10, ITGA8, SDC3, SV2B, SV2A, LAMC3, LAMB4, ITGA11, SV2C, COL5A3, GP6, TNN, COL6A6, LAMA1, AGRN

Predefined gene sets in Hepamine

Data source	Pathways	Symbols
KEGG	Endocytosis	ADRB1, ADRB2, ADRB3, GRK2, GRK3, AP2A1, AP2A2, AP2B1, ARF6, ARRB1, ARRB2, CBL, CBLB, CDC42, AP2M1, AP2S1, CLTA, CLTB, CLTC, CCR5, CSF1R, DAB2, DNM1, DNM2, EGF, EGFR, EPS15, ERBB3, ERBB4, F2R, FGFR3, FGFR2, FGFR4, FLT1, GRK4, GRK5, GRK6, HLA-A, HLA-B, HLA-C, HLA-E, HLA-F, HLA-G, HRAS, HSPA1A, HSPA1B, HSPA1L, HSPA2, HSPA6, HSPA8, IGF1R, IL2RA, IL2RB, IL2RG, CXCR1, CXCR2, KDR, KIT, LDLR, MDM2, MET, NEDD4, NTRK1, PDGFRA, PLD1, PLD2, PRKCI, PRKCZ, PSD, RAB4A, RAB5A, RAB5B, RAB5C, RET, GRK1, SH3GL1, SH3GL2, SH3GL3, SRC, TFRC, TRAF6, TSG101, CXCR4, STAM, CLTCL1, PIP5K1A, PIP5K1B, PIP4K2B, EEA1, RAB11A, ASAP2, USP8, RABEP1, HGS, RAB11B, VPS4B, RAB11FIP3, ACAP1, GIT2, DNAJC6, IQSEC1, PDCD6IP, DNM1L, RNF41, STAM2, STAMBP, EHD1, RAB31, WWP1, SNF8, VPS45, RAB11FIP2, EPN2, IQSEC2, NEDD4L, PSD3, PIP5K1C, ACAP2, PSD4, CBLC, CHMP2B, DNM3, RAB11FIP5, LDLRAP1, ARFGAP3, VPS4A, CHMP2A, GIT1, CHMP4A, EPN1, SH3KBP1, EHD4, EHD3, EHD2, ASAP1, PARD6A, VPS36, SH3GLB1, VPS28, CHMP5, VTA1, CHMP3, EPN3, VPS37C, ASAP3, ARFGAP1, PARD3, SH3GLB2, CHMP1B, SMURF1, RAB22A, SMAP1, RBSN, ARAP3, SMAP2, SMURF2, CHMP6, VPS37B, RAB11FIP1, RUFY1, ITCH, PSD2, VPS25, ARFGAP2, RAB11FIP4, PARD6G, PARD6B, MVB12B, CHMP4C, MVB12A, ACAP3, ARAP2, ARAP1, AGAP2, AGAP1, CHMP4B, GRK7, VPS37A, VPS37D, PIKFYVE, IQSEC3, LOC652614, LOC652799
KEGG	Endometrial Cancer	AKT1, AKT2, APC, ARAF, BAD, CCND1, BRAF, CASP9, CDH1, CTNNA1, CTNNA2, CTNNB1, EGF, EGFR, ELK1, ERBB2, FOXO3, GRB2, GSK3B, HRAS, ILK, KRAS, MLH1, MYC, NRAS, PDPK1, PIK3CA, PIK3CB, PIK3CD, PIK3CG, PIK3R1, PIK3R2, MAPK1, MAPK3, MAP2K1, MAP2K2, PTEN, RAF1, SOS1, SOS2, TCF7, TCF7L2, TP53, AXIN1, AXIN2, PIK3R3, AKT3, APC2, PIK3R5, CTNNA3, LEF1, TCF7L1

Predefined gene sets in Hepamine

Data source	Pathways	Symbols
KEGG	Epithelial Cell Signaling In Helicobacter Pylori Infection	ADAM10, ATP6V1A, ATP6V1B1, ATP6V1B2, ATP6V0C, ATP6V1C1, ATP6V1E1, ATP6V0B, ATP6V1G2, ATP6V0A1, ATP6AP1, CASP3, CDC42, CHUK, MAPK14, CSK, HBEGF, EGFR, CXCL1, IKBKB, CXCL8, CXCR1, CXCR2, JUN, LYN, MET, NFKB1, NFKBIA, PAK1, PLCG1, PLCG2, MAPK8, MAPK11, MAPK9, MAPK10, MAPK13, PTPN11, PTPRZ1, RAC1, RELA, MAPK12, CCL5, MAP2K4, SRC, ADAM17, TJP1, IKBKG, ATP6V0E1, MAP3K14, ATP6V0D1, ATP6V1F, ATP6V1G1, TCIRG1, NOD1, ATP6V0A2, GIT1, ATP6V0A4, F11R, ATP6V1D, ATP6V1H, JAM2, JAM3, ATP6V1E2, ATP6V1G3, IGSF5, ATP6V0E2, ATP6V0D2, ATP6V1C2
KEGG	Erbb Signaling Pathway	ABL1, ABL2, AKT1, AKT2, ARAF, AREG, BAD, BRAF, BTC, CAMK2A, CAMK2B, CAMK2D, CAMK2G, CBL, CBLB, CDKN1A, CDKN1B, CRK, CRKL, HBEGF, EGF, EGFR, EIF4EBP1, ELK1, ERBB2, ERBB3, ERBB4, EREG, MTOR, GAB1, GRB2, GSK3B, NRG1, HRAS, JUN, KRAS, MYC, NCK1, NRAS, PAK1, PAK2, PAK3, PIK3CA, PIK3CB, PIK3CD, PIK3CG, PIK3R1, PIK3R2, PLCG1, PLCG2, PRKCA, PRKCB, PRKCG, MAPK1, MAPK3, MAPK8, MAPK9, MAPK10, MAP2K1, MAP2K2, MAP2K7, PTK2, RAF1, RPS6KB1, RPS6KB2, MAP2K4, SHC1, SOS1, SOS2, SRC, STAT5A, STAT5B, TGFA, NCK2, PIK3R3, NRG2, AKT3, PAK4, NRG3, PIK3R5, CBLC, SHC2, SHC3, PAK6, PAK5, NRG4, SHC4
KEGG	Ether Lipid Metabolism	PAFAH1B1, PAFAH1B2, PAFAH1B3, PAFAH2, ENPP2, PLA2G1B, PLA2G2A, PLA2G4A, PLA2G5, PLD1, PLD2, PLA2G7, PLA2G6, PLA2G10, AGPS, PLPP1, PLPP2, PLPP3, JMJD7-PLA2G4B, PLA2G2D, PLA2G2E, PLA2G3, LPCAT2, CHPT1, PLA2G2F, LPCAT1, PLA2G12A, PLA2G12B, PLA2G4E, ENPP6, LPCAT4, PLA2G2C, PLA2G4B

Predefined gene sets in Hepamine

**Data source**	**Pathways**	**Symbols**
KEGG	Fatty Acid Metabolism	ACAA1, ACADL, ACADM, ACADS, ACADSB, ACADVL, ACAT1, ACAT2, ACOX1, ADH1A, ADH1B, ADH1C, ADH4, ADH5, ADH6, ADH7, ALDH2, ALDH1B1, ALDH9A1, ALDH3A2, ALDH7A1, CPT1A, CPT1B, CPT2, CYP4A11, ECI1, ECHS1, EHHADH, ACSL1, ACSL3, ACSL4, GCDH, HADHA, HADHB, HADH, ACOX3, ACAA2, ECI2, ACSL6, ACSL5, CPT1C, CYP4A22
KEGG	Fc Epsilon Ri Signaling Pathway	AKT1, AKT2, BTK, MAPK14, CSF2, FCER1A, MS4A2, FCER1G, FYN, GRB2, HRAS, IL3, IL4, IL5, IL13, INPP5D, KRAS, LCP2, LYN, NRAS, PDPK1, PIK3CA, PIK3CB, PIK3CD, PIK3CG, PIK3R1, PIK3R2, PLA2G1B, PLA2G2A, PLA2G4A, PLA2G5, PLCG1, PLCG2, PRKCA, PRKCB, PRKCD, PRKCE, MAPK1, MAPK3, MAPK8, MAPK11, MAPK9, MAPK10, MAPK13, MAP2K1, MAP2K2, MAP2K3, MAP2K6, MAP2K7, RAC1, RAC2, RAC3, RAF1, MAPK12, MAP2K4, SOS1, SOS2, SYK, TNF, VAV1, VAV2, PLA2G6, PLA2G10, PIK3R3, JMJD7-PLA2G4B, GAB2, AKT3, VAV3, PIK3R5, PLA2G2D, LAT, PLA2G2E, PLA2G3, PLA2G2F, PLA2G12A, PLA2G12B, PLA2G4E, PLA2G2C, PLA2G4B
KEGG	Fc Gamma R Mediated Phagocytosis	AKT1, AKT2, AMPH, ARF6, CDC42, CFL1, CFL2, CRK, CRKL, DNM1, DNM2, DOCK2, FCGR1A, FCGR2A, FCGR2B, FCGR3A, GSN, HCK, INPP5D, LIMK1, LIMK2, LYN, MARCKS, MYO10, PAK1, PIK3CA, PIK3CB, PIK3CD, PIK3CG, PIK3R1, PIK3R2, PLA2G4A, PLCG1, PLCG2, PLD1, PLD2, PRKCA, PRKCB, PRKCD, PRKCE, PRKCG, MAPK1, MAPK3, MAP2K1, PTPRC, RAC1, RAC2, RAF1, RPS6KB1, RPS6KB2, SYK, VASP, VAV1, VAV2, WAS, PIP5K1A, PIP5K1B, PIP4K2B, PLA2G6, PIK3R3, PLPP1, PLPP2, PLPP3, ASAP2, SPHK1, WASF1, WASL, FCGR2C, GAB2, AKT3, DNM1L, ARPC5, ARPC4, ARPC3, ARPC1B, ARPC2, WASF2, VAV3, ARPC1A, WASF3, PIP5K1C, PIK3R5, DNM3, LAT, ASAP1, ASAP3, SPHK2, MARCKSL1, ARPC5L, SCIN, PLA2G4E, PIKFYVE, PLA2G4F, PLA2G4D, NCF1, LOC653888, PLA2G4B

Predefined gene sets in Hepamine

Data source	Pathways	Symbols
KEGG	Focal Adhesion	ACTB, ACTG1, ACTN4, ACTN1, ACTN2, ACTN3, AKT1, AKT2, BIRC2, BIRC3, XIAP, RHOA, ARHGAP5, BAD, CCND1, BCL2, BRAF, CAPN2, CAV1, CAV2, CAV3, CCND2, CCND3, CDC42, CHAD, COL1A1, COL1A2, COL2A1, COL3A1, COL4A1, COL4A2, COL4A4, COL4A6, COL5A1, COL5A2, COL6A1, COL6A2, COL6A3, COL11A1, COL11A2, COMP, CRK, CRKL, CTNNB1, DIAPH1, DOCK1, EGF, EGFR, ELK1, ERBB2, VEGFD, FLNA, FLNB, FLNC, FLT1, FLT4, FN1, FYN, GRB2, RAPGEF1, ARHGAP35, GSK3B, HGF, HRAS, TNC, IBSP, IGF1, IGF1R, ILK, ITGA6, ITGA1, ITGA2, ITGA2B, ITGA3, ITGA4, ITGA5, ITGA7, ITGA9, ITGAV, ITGB1, ITGB3, ITGB4, ITGB5, ITGB6, ITGB7, ITGB8, JUN, KDR, LAMA2, LAMA3, LAMA4, LAMA5, LAMB1, LAMB2, LAMB3, LAMC1, LAMC2, MET, MYL2, MYL5, MYLK, PPP1R12A, PAK1, PAK2, PAK3, PDGFA, PDGFB, PDGFRA, PDGFRB, PDPK1, PGF, PIK3CA, PIK3CB, PIK3CD, PIK3CG, PIK3R1, PIK3R2, PPP1CA, PPP1CB, PPP1CC, PRKCA, PRKCB, PRKCG, MAPK1, MAPK3, MAPK8, MAPK9, MAPK10, MAP2K1, RELN, PTEN, PTK2, PXN, RAC1, RAC2, RAC3, RAF1, RAP1A, RAP1B, RASGRF1, ROCK1, SHC1, SOS1, SOS2, SPP1, SRC, THBS1, THBS2, THBS3, THBS4, TLN1, TNR, TNXB, VASP, VAV1, VAV2, VCL, VEGFA, VEGFB, VEGFC, VTN, VWF, ZYX, PIK3R3, ITGA10, ITGA8, ROCK2, BCAR1, AKT3, PAK4, LAMC3, MYL9, VAV3, MYL12A, LAMB4, ITGA11, PIP5K1C, PIK3R5, SHC2, PARVB, MYLPF, COL5A3, SHC3, PARVA, PDGFC, PAK6, PAK5, MYL7, TNN, PARVG, PDGFD, TLN2, MYLK2, MYLK3, MYL10, MYL12B, COL6A6, LAMA1, SHC4, LOC646821, LOC100418883
KEGG	Folate Biosynthesis	ALPI, ALPL, ALPP, ALPG, DHFR, FPGS, GCH1, PTS, QDPR, SPR, GGH
KEGG	Fructose And Mannose Metabolism	ALDOA, ALDOB, ALDOC, AKR1B1, FBP1, GMDS, HK1, HK2, HK3, KHK, MPI, PFKFB1, PFKFB2, PFKFB3, PFKFB4, PFKL, PFKM, PFKP, PMM1, PMM2, SORD, TPI1, TSTA3, MTMR1, FBP2, FPGT, MTMR2, MTMR6, MTMR7, PHPT1, GMPPB, GMPPA, AKR1B10, FUK

Predefined gene sets in Hepamine

**Data source**	**Pathways**	**Symbols**
KEGG	Galactose Metabolism	AKR1B1, G6PC, GAA, GALE, GALK1, GALK2, GALT, GANC, GCK, B4GALT1, GLA, GLB1, HK1, HK2, HK3, LALBA, LCT, PFKL, PFKM, PFKP, PGM1, UGP2, B4GALT2, MGAM, PGM2, G6PC2
KEGG	Gap Junction	ADCY1, ADCY2, ADCY3, ADCY5, ADCY6, ADCY7, ADCY8, ADCY9, ADRB1, CDK1, CSNK1D, DRD1, DRD2, LPAR1, EGF, EGFR, GJA1, GNA11, GNAI1, GNAI2, GNAI3, GNAQ, GNAS, GRB2, GRM1, GRM5, GUCY1A2, GUCY1A1, GUCY1B1, HRAS, HTR2A, HTR2B, HTR2C, ITPR1, ITPR2, ITPR3, KRAS, NRAS, PDGFA, PDGFB, PDGFRA, PDGFRB, PLCB2, PLCB3, PLCB4, PRKACA, PRKACB, PRKACG, PRKCA, PRKCB, PRKCG, PRKG1, PRKG2, MAPK1, MAPK3, MAPK7, MAP2K1, MAP2K2, MAP2K5, PRKX, RAF1, SOS1, SOS2, SRC, TJP1, TUBA4A, TUBA3C, TUBB2A, TUBA1A, TUBA1B, TUBB3, TUBB4A, TUBB4B, MAP3K2, PLCB1, TUBA8, PDGFC, TUBB7P, GJD2, TUBAL3, PDGFD, TUBB1, TUBB6, TUBA1C, TUBA3E, TUBA3D, ADCY4, TUBB, TUBB8, TUBB2B
KEGG	Glioma	AKT1, AKT2, ARAF, CCND1, BRAF, CALM1, CALM2, CALM3, CALML3, CAMK2A, CAMK2B, CAMK2D, CAMK2G, CDK4, CDK6, CDKN1A, CDKN2A, E2F1, E2F2, E2F3, EGF, EGFR, MTOR, GRB2, HRAS, IGF1, IGF1R, KRAS, MDM2, NRAS, PDGFA, PDGFB, PDGFRA, PDGFRB, PIK3CA, PIK3CB, PIK3CD, PIK3CG, PIK3R1, PIK3R2, PLCG1, PLCG2, PRKCA, PRKCB, PRKCG, MAPK1, MAPK3, MAP2K1, MAP2K2, PTEN, RAF1, RB1, SHC1, SOS1, SOS2, TGFA, TP53, PIK3R3, AKT3, PIK3R5, SHC2, CALML5, SHC3, CALML6, SHC4

Predefined gene sets in Hepamine

Data source	Pathways	Symbols
KEGG	Glutathione Metabolism	ANPEP, G6PD, GGT1, GGT7, GGT5, GCLC, GCLM, GPX1, GPX2, GPX3, GPX4, GPX5, GPX7, GSR, GSS, GSTA1, GSTA2, GSTA3, GSTA4, GSTM1, GSTM2, GSTM3, GSTM4, GSTM5, GSTP1, GSTT1, GSTT2, GSTZ1, IDH1, IDH2, MGST1, MGST2, MGST3, ODC1, PGD, RRM1, RRM2, SMS, SRM, GSTO1, OPLAH, RRM2B, LAP3, TXNDC12, GGCT, GSTO2, GGT6, GSTA5, GPX6, GSTK1
KEGG	Glycerolipid Metabolism	ALDH2, ALDH1B1, ALDH9A1, ALDH3A2, AKR1B1, ALDH7A1, CEL, DGKA, DGKB, DGKG, DGKQ, GK, GK2, GLA, LIPC, LPL, PNLIP, PNLIPRP1, PNLIPRP2, LIPF, DGKZ, DGKE, DGKD, PLPP1, PLPP2, PLPP3, DGAT1, DGKI, LIPG, AKR1A1, AGPAT1, AGPAT2, MGLL, TKFC, AGK, AGPAT3, AGPAT4, GPAM, PNPLA3, DGAT2, GPAT3, MBOAT2, GLYCTK, GPAT4, GPAT2, MBOAT1, AWAT2, DGKH, LCLAT1
KEGG	Glycerophospholipid Metabolism	ACHE, CDS1, CHAT, CHKA, CHKB, DGKA, DGKB, DGKG, DGKQ, GPD1, GPD2, LCAT, PCYT1A, PLA2G1B, PLA2G2A, PLA2G4A, PLA2G5, PLD1, PLD2, PCYT2, TAZ, PLA2G6, PLA2G10, GNPAT, DGKZ, DGKE, DGKD, PLPP1, PLPP2, PLPP3, JMJD7-PLA2G4B, CDS2, DGKI, PCYT1B, PGS1, PTDSS1, LPGAT1, LPCAT3, PEMT, CDIPT, LYPLA1, AGPAT1, AGPAT2, LYPLA2, GPD1L, PLA2G15, PISD, PLA2G2D, PLA2G2E, PLA2G3, CRLS1, LPCAT2, ETNK2, ETNK1, AGPAT3, AGPAT4, ADPRM, CHPT1, GPAM, PLA2G2F, MBOAT7, LPCAT1, PTDSS2, PLA2G12A, PLA2G12B, GPAT3, PLA2G4E, MBOAT2, GPAT4, GPAT2, MBOAT1, DGKH, PHOSPHO1, LCLAT1, LPCAT4, PLA2G2C, PLA2G4B
KEGG	Glycine Serine And Threonine Metabolism	AGXT, ALAS1, ALAS2, AMT, AOC2, BHMT, CBS, CTH, DAO, DLD, SARDH, GAMT, GATM, GLDC, MAOA, MAOB, PSPH, SHMT1, SHMT2, AOC3, SDS, GCAT, PHGDH, GNMT, DMGDH, PSAT1, PIPOX, CHDH, SRR, AGXT2, GLYCTK

Predefined gene sets in Hepamine

Data source	Pathways	Symbols
KEGG	Glycolysis Gluconeogenesis	ADH1A, ADH1B, ADH1C, ADH4, ADH5, ADH6, ADH7, ALDH2, ALDH3A1, ALDH1B1, ALDH1A3, ALDH3B1, ALDH3B2, ALDH9A1, ALDH3A2, ALDOA, ALDOB, ALDOC, ALDH7A1, BPGM, DLAT, DLD, ENO1, ENO2, ENO3, FBP1, G6PC, GAPDH, GCK, GPI, HK1, HK2, HK3, LDHA, LDHB, LDHC, PCK1, PCK2, PDHA1, PDHA2, PDHB, PFKL, PFKM, PFKP, PGAM1, PGAM2, PGK1, PGK2, PGM1, PKLR, PKM, TPI1, FBP2, AKR1A1, PGM2, ACSS2, G6PC2, ACSS1, LDHAL6B, GALM, LDHAL6A, PGAM4
KEGG	Glycosaminoglycan Biosynthesis Chondroitin Sulfate	CHST3, UST, B4GALT7, CHSY1, B3GAT3, B3GAT1, DSE, CHST11, CHST15, CHPF2, CSGALNACT2, CHST12, CSGALNACT1, CHST7, XYLT1, XYLT2, CHPF, CHST14, B3GALT6, B3GAT2, CHST13, CHSY3
KEGG	Glycosaminoglycan Biosynthesis Heparan Sulfate	EXT1, EXT2, EXTL1, EXTL2, EXTL3, NDST1, NDST2, NDST3, HS6ST1, HS2ST1, HS3ST3B1, HS3ST3A1, HS3ST2, HS3ST1, B4GALT7, GLCE, B3GAT3, B3GAT1, XYLT1, XYLT2, NDST4, HS6ST2, B3GALT6, B3GAT2, HS3ST5, HS6ST3
KEGG	Glycosaminoglycan Biosynthesis Keratan Sulfate	FUT8, B4GALT1, CHST6, ST3GAL1, ST3GAL2, ST3GAL3, CHST1, B4GALT4, B4GALT3, B4GALT2, CHST2, CHST4, B3GNT2, B4GAT1, B3GNT7
KEGG	Glycosaminoglycan Degradation	ARSB, GALNS, GLB1, GNS, GUSB, HEXA, HEXB, HYAL1, IDS, IDUA, NAGLU, SGSH, SPAM1, HYAL3, HYAL2, HS3ST3B1, HS3ST3A1, HPSE, HYAL4, HPSE2, HGSNAT
KEGG	Glycosphingolipid Biosynthesis Ganglio Series	B4GALNT1, GLB1, HEXA, HEXB, ST3GAL1, ST3GAL2, ST8SIA1, B3GALT4, ST3GAL5, SLC33A1, ST6GALNAC4, ST8SIA5, ST6GALNAC6, ST6GALNAC5, ST6GALNAC3
KEGG	Glycosphingolipid Biosynthesis Globo Series	FUT1, FUT2, GLA, HEXA, HEXB, NAGA, ST3GAL1, ST3GAL2, ST8SIA1, B3GALNT1, B3GALT5, FUT9, GBGT1, A4GALT

Predefined gene sets in Hepamine

Data source	Pathways	Symbols
KEGG	Glycosphingolipid Biosynthesis Lacto And Neolacto Series	ABO, FUT1, FUT2, FUT3, FUT4, FUT5, FUT6, FUT7, GCNT2, B4GALT1, ST3GAL4, ST3GAL3, ST8SIA1, B4GALT4, B4GALT3, B4GALT2, B3GALT2, B3GALT1, B3GALT5, B3GNT3, ST3GAL6, B3GNT2, FUT9, B4GAT1, B3GNT4, B3GNT5
KEGG	Glycosylphosphatidylinositol Gpi Anchor Biosynthesis	GPLD1, PIGA, PIGC, PIGF, PIGH, GPAA1, DPM2, PIGQ, PIGL, PIGB, PIGK, PIGN, PIGP, PIGT, PIGG, PIGX, PIGV, PGAP1, PIGZ, PIGO, PIGY, PIGM, PIGS, PIGU, PIGW
KEGG	Glyoxylate And Dicarboxylate Metabolism	ACO1, ACO2, CS, MDH1, MDH2, MTHFD1, GRHPR, MTHFD2, MTHFD1L, HAO2, HAO1, HYI, AFMID, GLYCTK, PGP, MTHFD2L
KEGG	Gnrh Signaling Pathway	ADCY1, ADCY2, ADCY3, ADCY5, ADCY6, ADCY7, ADCY8, ADCY9, ATF4, CACNA1C, CACNA1D, CACNA1F, CACNA1S, CALM1, CALM2, CALM3, CALML3, CAMK2A, CAMK2B, CAMK2D, CAMK2G, CDC42, CGA, MAPK14, HBEGF, EGFR, ELK1, PTK2B, FSHB, GNA11, GNAQ, GNAS, GNRH1, GNRH2, GNRHR, GRB2, HRAS, ITPR1, ITPR2, ITPR3, JUN, KRAS, LHB, MAP3K1, MAP3K3, MAP3K4, MMP2, MMP14, NRAS, PLA2G1B, PLA2G2A, PLA2G4A, PLA2G5, PLCB2, PLCB3, PLCB4, PLD1, PLD2, PRKACA, PRKACB, PRKACG, PRKCA, PRKCB, PRKCD, MAPK1, MAPK3, MAPK7, MAPK8, MAPK11, MAPK9, MAPK10, MAPK13, MAP2K1, MAP2K2, MAP2K3, MAP2K6, MAP2K7, PRKX, RAF1, MAPK12, MAP2K4, SOS1, SOS2, SRC, PLA2G6, PLA2G10, JMJD7-PLA2G4B, MAP3K2, PLCB1, PLA2G2D, PLA2G2E, PLA2G3, CALML5, PLA2G2F, PLA2G12A, PLA2G12B, PLA2G4E, CALML6, ADCY4, PLA2G2C, PLA2G4B

Predefined gene sets in Hepamine

Data source	Pathways	Symbols
KEGG	Graft Versus Host Disease	FAS, FASLG, CD28, CD80, CD86, GZMB, HLA-A, HLA-B, HLA-C, HLA-DMA, HLA-DMB, HLA-DOA, HLA-DOB, HLA-DPA1, HLA-DPB1, HLA-DQA1, HLA-DQA2, HLA-DQB1, HLA-DRA, HLA-DRB1, HLA-DRB3, HLA-DRB4, HLA-DRB5, HLA-E, HLA-F, HLA-G, IFNG, IL1A, IL1B, IL2, IL6, KIR2DL1, KIR2DL2, KIR2DL3, KIR3DL1, KIR3DL2, KLRC1, KLRD1, PRF1, TNF, KIR2DL5A, LOC652614
KEGG	Hedgehog Signaling Pathway	BMP2, BMP4, BMP5, BMP6, BMP7, BMP8B, CSNK1A1, CSNK1D, CSNK1E, CSNK1G2, CSNK1G3, GAS1, GLI1, GLI2, GLI3, GSK3B, IHH, LRP2, PRKACA, PRKACB, PRKACG, PRKX, PTCH1, SHH, SMO, WNT1, WNT2, WNT3, WNT5A, WNT6, WNT7A, WNT7B, WNT8A, WNT8B, WNT10B, WNT11, WNT2B, WNT9A, WNT9B, ZIC2, PTCH2, BTRC, FBXW11, STK36, DHH, WNT16, SUFU, RAB23, CSNK1G1, WNT4, HHIP, WNT10A, WNT5B, WNT3A, CSNK1A1L, BMP8A
KEGG	Hematopoietic Cell Lineage	ANPEP, CD1A, CD1B, CD1C, CD1D, CD1E, CD2, CD3D, CD3E, CD3G, CD4, CD5, CD7, CD8A, CD8B, CD9, CD14, CD19, MS4A1, CD22, CD33, CD34, CD36, CD37, CD38, CD44, CD59, CR1, CR2, CSF1, CSF1R, CSF2, CSF2RA, CSF3, CSF3R, CD55, DNTT, EPO, EPOR, FCER2, FCGR1A, FLT3, FLT3LG, GP1BA, GP1BB, GP5, GP9, GYPA, HLA-DRA, HLA-DRB1, HLA-DRB3, HLA-DRB4, HLA-DRB5, IL1A, IL1B, IL1R1, IL2RA, IL3, IL3RA, IL4, IL4R, IL5, IL5RA, IL6, IL6R, IL7, IL7R, IL9R, IL11, IL11RA, ITGA6, ITGA1, ITGA2, ITGA2B, ITGA3, ITGA4, ITGA5, ITGAM, ITGB3, KIT, KITLG, MME, TFRC, THPO, TNF, TPO, IL1R2, LOC652799
KEGG	Histidine Metabolism	AOC1, ALDH2, ALDH3A1, ALDH1B1, ALDH1A3, ALDH3B1, ALDH3B2, ALDH9A1, ALDH3A2, ASPA, ALDH7A1, DDC, HAL, HDC, HNMT, MAOA, MAOB, LCMT2, FTCD, HEMK1, LCMT1, METTL2B, TRMT11, CNDP1, ACY3, BUD23, UROC1, METTL6, AMDHD1

Predefined gene sets in Hepamine

Data source	Pathways	Symbols
KEGG	Homologous Recombination	BLM, BRCA2, MRE11, NBN, POLD1, POLD2, RAD51, RAD51C, RAD51B, RAD51D, RAD52, RPA1, RPA2, RPA3, SSBP1, TOP3A, XRCC2, XRCC3, SEM1, RAD54L, TOP3B, RAD50, POLD3, RAD54B, RPA4, POLD4, MUS81, EME1
KEGG	Huntingtons Disease	AP2A1, AP2A2, AP2B1, SLC25A4, SLC25A5, SLC25A6, APAF1, ATP5F1A, ATP5F1B, ATP5F1C, ATP5F1D, ATP5F1E, ATP5PB, ATP5MC1, ATP5MC2, ATP5MC3, ATP5PF, ATP5PO, BAX, BDNF, CASP3, CASP8, CASP9, AP2M1, AP2S1, CLTA, CLTB, CLTC, COX4I1, COX5B, COX6A1, COX6A2, COX6B1, COX6C, COX7A1, COX7A2, COX7B, COX7C, COX8A, CREB1, CREBBP, CYC1, DCTN1, DLG4, EP300, GNAQ, GPX1, GRIN1, GRIN2B, GRM5, HTT, HDAC1, HDAC2, HIP1, ITPR1, ATP6, ATP8, COX1, COX2, COX3, CYTB, NDUFA1, NDUFA2, NDUFA3, NDUFA4, NDUFA5, NDUFA6, NDUFA7, NDUFA8, NDUFA9, NDUFA10, NDUFAB1, NDUFB1, NDUFB2, NDUFB3, NDUFB4, NDUFB5, NDUFB6, NDUFB7, NDUFB8, NDUFB9, NDUFB10, NDUFC1, NDUFC2, NDUFS1, NDUFS2, NDUFS3, NDUFV1, NDUFS4, NDUFS5, NDUFS6, NDUFS8, NDUFV2, NDUFV3, NRF1, PLCB2, PLCB3, PLCB4, POLR2A, POLR2B, POLR2C, POLR2D, POLR2E, POLR2F, POLR2G, POLR2H, POLR2I, POLR2J, POLR2K, POLR2L, PPARG, PPID, REST, SDHA, SDHB, SDHC, SDHD, SOD1, SOD2, SP1, TAF4, TAF4B, TBP, TFAM, TGM2, TP53, UCP1, UQCRB, UQCRC1, UQCRC2, UQCRFS1, UQCRH, VDAC1, VDAC2, VDAC3, DNALI1, CLTCL1, HAP1, COX7A2L, COX5A, TBPL1, CREB5, DNAL4, ATP5PD, CREB3, DCTN2, PPARGC1A, UQCR11, RCOR1, PLCB1, SIN3A, DNAH1, DNAI1, UQCRQ, BBC3, UQCR10, DCTN4, CYCS, IFT57, DNAH3, NDUFA4L2, DNAI2, CREB3L2, SLC25A31, DNAL1, CREB3L3, COX4I2, CREB3L1, COX6B2, DNAH2, CREB3L4, COX7B2, POLR2J2, COX8C, NDUFS7, TBPL2, ATP5MC1P5, UQCRHL, POLR2J3, LOC642502, LOC644310, LOC727947, LOC729317, COX6CP3, LOC100133737

Predefined gene sets in Hepamine

**Data source**	**Pathways**	**Symbols**
KEGG	Hypertrophic Cardiomyopathy Hcm	ACTB, ACTC1, ACTG1, ATP2A2, CACNA1C, CACNA1D, CACNA1F, CACNA1S, CACNA2D1, CACNB1, CACNB2, CACNB3, CACNB4, CACNG1, DAG1, ACE, DES, DMD, EMD, IGF1, IL6, ITGA6, ITGA1, ITGA2, ITGA2B, ITGA3, ITGA4, ITGA5, ITGA7, ITGA9, ITGAV, ITGB1, ITGB3, ITGB4, ITGB5, ITGB6, ITGB7, ITGB8, LAMA2, LMNA, MYBPC3, MYH6, MYH7, MYL2, MYL3, PRKAA1, PRKAA2, PRKAB1, PRKAB2, PRKAG1, RYR2, SGCA, SGCB, SGCD, SGCG, SLC8A1, TGFB1, TGFB2, TGFB3, TNF, TNNC1, TNNI3, TNNT2, TPM1, TPM2, TPM3, TPM4, TTN, ITGA10, ITGA8, CACNA2D2, CACNG3, CACNG2, ITGA11, CACNG5, CACNG4, PRKAG2, PRKAG3, CACNA2D3, CACNG8, CACNG7, CACNG6, CACNA2D4, LOC646821, LOC100418883
KEGG	Inositol Phosphate Metabolism	IMPA1, IMPA2, INPP1, INPP4A, INPP5A, INPP5B, INPPL1, ITPK1, ITPKA, ITPKB, ALDH6A1, OCRL, PIK3C2A, PIK3C2B, PIK3C2G, PIK3C3, PIK3CA, PIK3CB, PIK3CD, PIK3CG, PI4KA, PI4KB, PIP4K2A, PLCB2, PLCB3, PLCB4, PLCD1, PLCG1, PLCG2, PTEN, TPI1, PIP5K1A, PIP5K1B, PIP4K2B, INPP4B, SYNJ1, SYNJ2, MINPP1, CDIPT, PLCB1, PIP5K1C, INPP5J, PLCE1, ISYNA1, INPP5K, MIOX, INPP5E, IPPK, PIP4K2C, PLCD4, PLCZ1, PLCD3, PIKFYVE, IPMK

Predefined gene sets in Hepamine

Data source	Pathways	Symbols
KEGG	Insulin Signaling Pathway	ACACA, ACACB, AKT1, AKT2, ARAF, BAD, BRAF, CALM1, CALM2, CALM3, CALML3, CBL, CBLB, CRK, CRKL, EIF4E, EIF4EBP1, ELK1, FASN, FBP1, FOXO1, FLOT2, MTOR, G6PC, GCK, MKNK2, GRB2, RAPGEF1, GSK3B, GYS1, GYS2, HK1, HK2, HK3, HRAS, IKBKB, INS, INPP5D, INSR, IRS1, KRAS, LIPE, NRAS, PCK1, PCK2, PDE3A, PDE3B, PDPK1, PHKA1, PHKA2, PHKB, PHKG1, PHKG2, PIK3CA, PIK3CB, PIK3CD, PIK3CG, PIK3R1, PIK3R2, PKLR, PPP1CA, PPP1CB, PPP1CC, PPP1R3A, PPP1R3C, PPP1R3D, PRKAA1, PRKAA2, PRKAB1, PRKAB2, PRKACA, PRKACB, PRKACG, PRKAG1, PRKAR1A, PRKAR1B, PRKAR2A, PRKAR2B, PRKCI, PRKCZ, MAPK1, MAPK3, MAPK8, MAPK9, MAPK10, MAP2K1, MAP2K2, PRKX, PTPN1, PTPRF, PYGB, PYGL, PYGM, RAF1, RHEB, RPS6, RPS6KB1, RPS6KB2, SHC1, SLC2A4, SOS1, SOS2, SREBF1, TSC1, TSC2, IRS4, PIK3R3, MKNK1, SOCS1, IRS2, FBP2, SOCS2, SOCS3, TRIP10, EIF4E2, AKT3, FLOT1, SORBS1, SH2B2, PPARGC1A, EXOC7, RHOQ, PIK3R5, CBLC, SHC2, PRKAG2, INPP5K, CALML5, SHC3, PRKAG3, RPTOR, G6PC2, PPP1R3B, SOCS4, CALML6, EIF4E1B, SHC4
KEGG	Intestinal Immune Network For Iga Production	TNFRSF17, CD28, CD80, CD86, CD40, CD40LG, CCR10, HLA-DMA, HLA-DMB, HLA-DOA, HLA-DOB, HLA-DPA1, HLA-DPB1, HLA-DQA1, HLA-DQA2, HLA-DQB1, HLA-DRA, HLA-DRB1, HLA-DRB3, HLA-DRB4, HLA-DRB5, IL2, IL4, IL5, IL6, IL10, IL15, IL15RA, ITGA4, ITGB7, LTBR, PIGR, CCL25, CXCL12, TGFB1, CXCR4, MADCAM1, TNFSF13, MAP3K14, TNFSF13B, CCR9, CCL27, ICOSLG, TNFRSF13B, ICOS, CCL28, AICDA, TNFRSF13C

Predefined gene sets in Hepamine

Data source	Pathways	Symbols
KEGG	Jak Stat Signaling Pathway	AKT1, AKT2, CCND1, BCL2L1, CBL, CBLB, CCND2, CCND3, CISH, CNTF, CNTFR, CREBBP, CSF2, CSF2RA, CSF2RB, CSF3, CSF3R, CSH1, CTF1, EP300, EPO, EPOR, GH1, GH2, GHR, GRB2, IFNA1, IFNA2, IFNA4, IFNA5, IFNA6, IFNA7, IFNA8, IFNA10, IFNA13, IFNA14, IFNA16, IFNA17, IFNA21, IFNAR1, IFNAR2, IFNB1, IFNG, IFNGR1, IFNGR2, IFNW1, IL2, IL2RA, IL2RB, IL2RG, IL3, IL3RA, IL4, IL4R, IL5, IL5RA, IL6, IL6R, IL6ST, IL7, IL7R, IL9, IL9R, IL10, IL10RA, IL10RB, IL11, IL11RA, IL12A, IL12B, IL12RB1, IL12RB2, IL13, IL13RA1, IL13RA2, IL15, IL15RA, JAK1, JAK2, JAK3, LEP, LEPR, LIF, LIFR, MPL, MYC, OSM, PIK3CA, PIK3CB, PIM1, PIK3CD, PIK3CG, PIK3R1, PIK3R2, PRL, PRLR, PTPN6, PTPN11, SOS1, SOS2, STAT1, STAT2, STAT3, STAT4, STAT5A, STAT5B, STAT6, TPO, TYK2, STAM, PIK3R3, PIAS1, SOCS1, SOCS2, SOCS3, PIAS2, OSMR, SOCS5, AKT3, SPRY3, SPRY1, SPRY2, STAM2, IRF9, PIAS3, IL24, CLCF1, PIK3R5, CBLC, IL19, SOCS7, IL20, IL21R, IL22, IL23A, PIAS4, IL20RA, IL20RB, IL26, IFNK, IL22RA1, IL21, CRLF2, SPRY4, TSLP, IL22RA2, SOCS4, IL23R, SPRED1, IFNLR1, SPRED2, IFNL2, IFNL3, IFNL1, IFNE
KEGG	Leishmania Infection	C3, CR1, MAPK14, CYBA, ELK1, FCGR1A, FCGR2A, FCGR3A, FCGR3B, FOS, HLA-DMA, HLA-DMB, HLA-DOA, HLA-DOB, HLA-DPA1, HLA-DPB1, HLA-DQA1, HLA-DQA2, HLA-DQB1, HLA-DRA, HLA-DRB1, HLA-DRB3, HLA-DRB4, HLA-DRB5, IFNG, IFNGR1, IFNGR2, IL1A, IL1B, IL4, IL10, IL12A, IL12B, IRAK1, ITGA4, ITGAM, ITGB1, ITGB2, JAK1, JAK2, JUN, MYD88, NCF2, NCF4, NFKB1, NFKBIA, NFKBIB, NOS2, PRKCB, MAPK1, MAPK3, MAPK11, MAPK13, PTGS2, PTPN6, RELA, MAPK12, STAT1, MAP3K7, TGFB1, TGFB2, TGFB3, TLR2, TLR4, TNF, TRAF6, FCGR2C, TAB1, TAB2, IRAK4, MARCKSL1, NCF1

Predefined gene sets in Hepamine

Data source	Pathways	Symbols
KEGG	Leukocyte Transendothelial Migration	ACTB, ACTG1, ACTN4, ACTN1, ACTN2, ACTN3, RHOA, ARHGAP5, RHOH, CDC42, CDH5, CLDN4, CLDN3, CLDN7, MAPK14, CTNNA1, CTNNA2, CTNNB1, CTNND1, CYBA, CYBB, PTK2B, GNAI1, GNAI2, GNAI3, ARHGAP35, ICAM1, ITGA4, ITGAL, ITGAM, ITGB1, ITGB2, ITK, CD99, AFDN, MMP2, MMP9, MSN, MYL2, MYL5, NCF2, NCF4, CLDN11, PECAM1, PIK3CA, PIK3CB, PIK3CD, PIK3CG, PIK3R1, PIK3R2, PLCG1, PLCG2, PRKCA, PRKCB, PRKCG, MAPK11, MAPK13, PTK2, PTPN11, PXN, RAC1, RAC2, RAP1A, RAP1B, ROCK1, MAPK12, CXCL12, SIPA1, THY1, CLDN5, TXK, VASP, VAV1, VAV2, VCAM1, VCL, EZR, CXCR4, PIK3R3, CLDN10, CLDN8, CLDN6, CLDN2, CLDN1, CLDN9, ROCK2, BCAR1, MYL9, RAPGEF3, VAV3, MYL12A, CLDN16, RAPGEF4, PIK3R5, CLDN14, CLDN15, CLDN17, NOX1, CTNNA3, MYLPF, CLDN20, NOX3, F11R, CLDN18, CLDN22, JAM2, MYL7, RASSF5, JAM3, ESAM, MYL10, MYL12B, CLDN23, CLDN19, LOC646821, NCF1, LOC100418883, OCLN
KEGG	Limonene And Pinene Degradation	ALDH2, ALDH1B1, ALDH9A1, ALDH3A2, ALDH7A1, ECHS1, EHHADH, HADHA, NAA80, YOD1
KEGG	Linoleic Acid Metabolism	ALOX15, CYP1A2, CYP3A7, CYP2C19, CYP2C8, CYP2C9, CYP2C18, CYP2E1, CYP2J2, CYP3A4, CYP3A5, PLA2G1B, PLA2G2A, PLA2G4A, PLA2G5, PLA2G6, PLA2G10, JMJD7-PLA2G4B, PLA2G2D, PLA2G2E, PLA2G3, AKR1B10, PLA2G2F, CYP3A43, PLA2G12A, PLA2G12B, PLA2G4E, PLA2G2C, PLA2G4B

Predefined gene sets in Hepamine

Data source	Pathways	Symbols
KEGG	Long Term Depression	ARAF, BRAF, CACNA1A, CRH, CRHR1, GNA11, GNA12, GNAI1, GNAI2, GNAI3, GNAO1, GNAQ, GNAS, GNAZ, GRIA1, GRIA2, GRIA3, GRID2, GRM1, GRM5, GUCY1A2, GUCY1A1, GUCY1B1, HRAS, IGF1, IGF1R, ITPR1, ITPR2, ITPR3, KRAS, LYN, NOS1, NRAS, PLA2G1B, PLA2G2A, PLA2G4A, PLA2G5, PLCB2, PLCB3, PLCB4, PPP2CA, PPP2CB, PPP2R1A, PPP2R1B, PRKCA, PRKCB, PRKCG, PRKG1, PRKG2, MAPK1, MAPK3, MAP2K1, MAP2K2, RAF1, RYR1, PLA2G6, PLA2G10, JMJD7-PLA2G4B, GNA13, PPP1R17, PLCB1, PLA2G2D, PLA2G2E, PLA2G3, PLA2G2F, PLA2G12A, PLA2G12B, PLA2G4E, PLA2G2C, PLA2G4B
KEGG	Long Term Potentiation	ADCY1, ADCY8, ARAF, ATF4, BRAF, CACNA1C, CALM1, CALM2, CALM3, CALML3, CAMK4, CAMK2A, CAMK2B, CAMK2D, CAMK2G, CREBBP, EP300, GNAQ, GRIA1, GRIA2, GRIN1, GRIN2A, GRIN2B, GRIN2C, GRIN2D, GRM1, GRM5, HRAS, ITPR1, ITPR2, ITPR3, KRAS, PPP1R12A, NRAS, PLCB2, PLCB3, PLCB4, PPP1CA, PPP1CB, PPP1CC, PPP1R1A, PPP3CA, PPP3CB, PPP3CC, PPP3R1, PPP3R2, PRKACA, PRKACB, PRKACG, PRKCA, PRKCB, PRKCG, MAPK1, MAPK3, MAP2K1, MAP2K2, PRKX, RAF1, RAP1A, RAP1B, RPS6KA1, RPS6KA2, RPS6KA3, RAPGEF3, CHP1, PLCB1, RPS6KA6, CALML5, CHP2, CALML6
KEGG	Lysine Degradation	ACAT1, ACAT2, ALDH2, ALDH1B1, ALDH9A1, ALDH3A2, ALDH7A1, DLST, ECHS1, EHHADH, GCDH, HADHA, HADH, OGDH, PLOD1, PLOD2, SETMAR, SUV39H1, NSD2, BBOX1, PLOD3, SETD1A, SETDB1, AASS, EHMT2, SETD1B, SETD2, KMT5B, AADAT, PIPOX, NSD3, TMLHE, OGDHL, ASH1L, AASDHPPT, NSD1, SUV39H2, EHMT1, SETD7, SETDB2, DOT1L, KMT5C, AASDH, KMT5A

Predefined gene sets in Hepamine

Data source	Pathways	Symbols
KEGG	Lysosome	ABCA2, ACP2, ACP5, AP1B1, AP1G1, AGA, ARSA, ARSB, ASAH1, ATP6V0C, ATP6V0B, ATP6V0A1, ATP6AP1, SCARB2, CD63, CD68, CTSC, AP1S1, AP3S1, TPP1, CLN3, CLN5, CLTA, CLTB, CLTC, CTNS, CTSB, CTSD, CTSE, CTSG, CTSH, CTSK, CTSL, CTSV, CTSO, CTSS, CTSW, CTSZ, DNASE2, FUCA1, GAA, GALC, GALNS, GBA, GLA, GLB1, GM2A, GNS, GUSB, HEXA, HEXB, HYAL1, IDS, IDUA, IGF2R, LAMP1, LAMP2, LIPA, M6PR, MAN2B1, MANBA, NAGA, NAGLU, NEU1, NPC1, SLC11A2, CTSA, PPT1, LGMN, PSAP, SORT1, SGSH, SLC11A1, SMPD1, LAPTM5, AP3B2, CLTCL1, AP3B1, CTSF, CD164, AP1S2, AP1M1, AP3D1, ATP6V0D1, AP4M1, PPT2, NAPSA, ENTPD4, LAPTM4A, AP1M2, AP3S2, TCIRG1, NPC2, AP4B1, AP3M2, AP4S1, ARSG, GGA2, GGA3, AP4E1, ABCB9, ATP6V0A2, PLA2G15, GGA1, SLC17A5, AP3M1, LAMP3, ATP6V0A4, NAGPA, ATP6V1H, LAPTM4B, MCOLN1, DNASE2B, GNPTAB, GNPTG, AP1S3, HGSNAT, ATP6V0D2, MFSD8, SUMF1, PSAPL1

Predefined gene sets in Hepamine

Data source	Pathways	Symbols
KEGG	Mapk Signaling Pathway	AKT1, AKT2, FAS, FASLG, ARRB1, ARRB2, ATF4, BDNF, BRAF, CACNA1A, CACNA1B, CACNA1C, CACNA1D, CACNA1E, CACNA1F, CACNA1S, CACNA2D1, CACNB1, CACNB2, CACNB3, CACNB4, CACNG1, CASP3, CD14, CDC25B, CDC42, CHUK, MAP3K8, ATF2, CRK, CRKL, MAPK14, DAXX, GADD45A, DDIT3, DUSP1, DUSP2, DUSP3, DUSP4, DUSP5, DUSP6, DUSP7, DUSP8, DUSP9, EGF, EGFR, ELK1, ELK4, MECOM, FGF1, FGF2, FGF3, FGF4, FGF5, FGF6, FGF7, FGF8, FGF9, FGF10, FGF11, FGF12, FGF13, FGF14, FGFR1, FGFR3, FGFR2, FGFR4, FLNA, FLNB, FLNC, FOS, GNA12, MKNK2, GRB2, NR4A1, HRAS, HSPA1A, HSPA1B, HSPA1L, HSPA2, HSPA6, HSPA8, HSPB1, IKBKB, IL1A, IL1B, IL1R1, JUN, JUND, KRAS, STMN1, MAPT, MAX, MEF2C, MAP3K1, MAP3K3, MAP3K4, MAP3K5, MAP3K11, MOS, MYC, GADD45B, NF1, NFATC2, NFATC4, NFKB1, NFKB2, NGF, NRAS, NTF3, NTF4, NTRK1, NTRK2, PAK1, PAK2, PDGFA, PDGFB, PDGFRA, PDGFRB, PLA2G1B, PLA2G2A, PLA2G4A, PLA2G5, PPM1A, PPM1B, PPP3CA, PPP3CB, PPP3CC, PPP3R1, PPP3R2, PPP5C, PRKACA, PRKACB, PRKACG, PRKCA, PRKCB, PRKCG, MAPK1, MAPK3, MAPK7, MAPK8, MAPK11, MAPK9, MAPK10, MAPK13, MAP2K1, MAP2K2, MAP2K3, MAP2K5, MAP2K6, MAP2K7, PRKX, PTPN7, PTPRR, MAP4K2, RAC1, RAC2, RAC3, RAF1, RAP1A, RAP1B, RASA1, RASA2, RASGRF1, RASGRF2, RELA, RELB, RPS6KA1, RPS6KA2, RPS6KA3, RRAS, MAPK12, MAP2K4, SOS1, SOS2, SRF, STK3, STK4, MAP3K7, TGFB1, TGFB2, TGFB3, TGFBR1, TGFBR2, TNF, TNFRSF1A, TP53, TRAF2, TRAF6, MAP3K12, IL1R2, MAPKAPK3, FGF23, PLA2G6, PLA2G10, MAP4K3, IKBKG, MAPKAPK5, MKNK1, LAMTOR3, JMJD7-PLA2G4B, FGF18, FGF17, FGF16, CACNA1I, CACNA1H, CACNA1G, RPS6KA4, MAP3K14, MAP3K6, MAP3K13, RPS6KA5, CACNA2D2, MAPKAPK2, TAOK2, MAP4K4, MAPK8IP1, RAPGEF2, FGF19, AKT3, RASGRP1, RASGRP2, CACNG3, CACNG2, TAB1, MAP3K2, GADD45G, DUSP14, MAP4K1, DUSP10, CHP1, RRAS2, MRAS, TAB2, MAPK8IP3, MAPK8IP2, RASGRP3, PLA2G2D, FGF20, FGF21, FGF22, CACNG5, CACNG4, RPS6KA6, PLA2G2E, PLA2G3, ECSIT, TAOK3, NLK, MAP3K20, CACNA2D3

Predefined gene sets in Hepamine

Data source	Pathways	Symbols
KEGG	Maturity Onset Diabetes Of The Young	NR5A2, GCK, HHEX, MNX1, FOXA2, FOXA3, HNF4A, HNF4G, ONECUT1, HES1, IAPP, INS, PDX1, NEUROD1, NKX2-2, NKX6-1, PAX4, PAX6, PKLR, SLC2A2, HNF1A, HNF1B, NEUROG3, BHLHA15, MAFA
KEGG	Melanogenesis	ADCY1, ADCY2, ADCY3, ADCY5, ADCY6, ADCY7, ADCY8, ADCY9, ASIP, CALM1, CALM2, CALM3, CALML3, CAMK2A, CAMK2B, CAMK2D, CAMK2G, CREB1, CREBBP, CTNNB1, DCT, DVL1, DVL2, DVL3, EDN1, EDNRB, EP300, FZD2, GNAI1, GNAI2, GNAI3, GNAO1, GNAQ, GNAS, GSK3B, HRAS, KIT, KRAS, MC1R, KITLG, MITF, NRAS, PLCB2, PLCB3, PLCB4, POMC, PRKACA, PRKACB, PRKACG, PRKCA, PRKCB, PRKCG, MAPK1, MAPK3, MAP2K1, MAP2K2, PRKX, RAF1, TCF7, TCF7L2, TYR, TYRP1, WNT1, WNT2, WNT3, WNT5A, WNT6, WNT7A, WNT7B, WNT8A, WNT8B, WNT10B, WNT11, WNT2B, WNT9A, WNT9B, FZD5, FZD3, FZD1, FZD4, FZD6, FZD7, FZD8, FZD9, CREB3, FZD10, PLCB1, LEF1, WNT16, CALML5, WNT4, CREB3L2, WNT10A, WNT5B, TCF7L1, CREB3L3, WNT3A, CREB3L1, CREB3L4, CALML6, ADCY4, LOC652799
KEGG	Melanoma	AKT1, AKT2, ARAF, BAD, CCND1, BRAF, CDH1, CDK4, CDK6, CDKN1A, CDKN2A, E2F1, E2F2, E2F3, EGF, EGFR, FGF1, FGF2, FGF3, FGF4, FGF5, FGF6, FGF7, FGF8, FGF9, FGF10, FGF11, FGF12, FGF13, FGF14, FGFR1, HGF, HRAS, IGF1, IGF1R, KRAS, MDM2, MET, MITF, NRAS, PDGFA, PDGFB, PDGFRA, PDGFRB, PIK3CA, PIK3CB, PIK3CD, PIK3CG, PIK3R1, PIK3R2, MAPK1, MAPK3, MAP2K1, MAP2K2, PTEN, RAF1, RB1, TP53, FGF23, PIK3R3, FGF18, FGF17, FGF16, FGF19, AKT3, PIK3R5, FGF20, FGF21, FGF22, PDGFC, PDGFD

Predefined gene sets in Hepamine

**Data source**	**Pathways**	**Symbols**
KEGG	Metabolism Of Xenobiotics By Cytochrome P450	ADH1A, ADH1B, ADH1C, ADH4, ADH5, ADH6, ADH7, ALDH3A1, ALDH1A3, ALDH3B1, ALDH3B2, AKR1C4, CYP1A1, CYP1A2, CYP1B1, CYP3A7, CYP2B6, CYP2C19, CYP2C8, CYP2C9, CYP2C18, CYP2E1, CYP2F1, CYP3A4, CYP3A5, AKR1C1, AKR1C2, EPHX1, GSTA1, GSTA2, GSTA3, GSTA4, GSTM1, GSTM2, GSTM3, GSTM4, GSTM5, GSTP1, GSTT1, GSTT2, GSTZ1, MGST1, MGST2, MGST3, UGT2B4, UGT2B7, UGT2B10, UGT2B15, UGT2B17, AKR1C3, GSTO1, UGT2B11, UGT2A1, DHDH, CYP2S1, UGT2B28, UGT1A10, UGT1A8, UGT1A7, UGT1A6, UGT1A5, UGT1A9, UGT1A4, UGT1A1, UGT1A3, CYP3A43, UGT2A3, GSTO2, GSTA5, GSTK1
KEGG	Mismatch Repair	MSH6, LIG1, MLH1, MSH2, MSH3, PCNA, PMS2, POLD1, POLD2, RFC1, RFC2, RFC3, RFC4, RFC5, RPA1, RPA2, RPA3, SSBP1, EXO1, POLD3, MLH3, RPA4, POLD4
KEGG	Mtor Signaling Pathway	AKT1, AKT2, BRAF, EIF4B, EIF4E, EIF4EBP1, VEGFD, MTOR, HIF1A, IGF1, INS, PDPK1, PGF, PIK3CA, PIK3CB, PIK3CD, PIK3CG, PIK3R1, PIK3R2, PRKAA1, PRKAA2, MAPK1, MAPK3, RHEB, RPS6, RPS6KA1, RPS6KA2, RPS6KA3, RPS6KB1, RPS6KB2, STK11, TSC1, TSC2, VEGFA, VEGFB, VEGFC, ULK1, PIK3R3, EIF4E2, ULK2, AKT3, PIK3R5, ULK3, RPS6KA6, CAB39, DDIT4, RPTOR, MLST8, CAB39L, STRADA, RICTOR, EIF4E1B

Predefined gene sets in Hepamine

Data source	Pathways	Symbols
KEGG	Natural Killer Cell Mediated Cytotoxicity	FAS, FASLG, ARAF, BID, BRAF, CASP3, CD247, CD48, CSF2, PTK2B, FCER1G, FCGR3A, FCGR3B, FYN, GRB2, GZMB, HLA-A, HLA-B, HLA-C, HLA-E, HLA-G, HRAS, ICAM1, ICAM2, IFNA1, IFNA2, IFNA4, IFNA5, IFNA6, IFNA7, IFNA8, IFNA10, IFNA13, IFNA14, IFNA16, IFNA17, IFNA21, IFNAR1, IFNAR2, IFNB1, IFNG, IFNGR1, IFNGR2, ITGAL, ITGB2, KIR2DL1, KIR2DL2, KIR2DL3, KIR2DL4, KIR2DS1, KIR2DS3, KIR2DS4, KIR2DS5, KIR3DL1, KIR3DL2, KLRC1, KLRC2, KLRC3, KLRD1, KRAS, LCK, LCP2, SH2D1A, MICB, NFATC1, NFATC2, NFATC3, NFATC4, NRAS, PAK1, PIK3CA, PIK3CB, PIK3CD, PIK3CG, PIK3R1, PIK3R2, PLCG1, PLCG2, PPP3CA, PPP3CB, PPP3CC, PPP3R1, PPP3R2, PRF1, PRKCA, PRKCB, PRKCG, MAPK1, MAPK3, MAP2K1, MAP2K2, PTPN6, PTPN11, RAC1, RAC2, RAC3, RAF1, SH3BP2, SHC1, SOS1, SOS2, SYK, TNF, TYROBP, VAV1, VAV2, ZAP70, PIK3R3, TNFSF10, TNFRSF10D, TNFRSF10C, TNFRSF10B, TNFRSF10A, NCR2, NCR1, VAV3, NFAT5, HCST, CHP1, KLRK1, PIK3R5, SHC2, LAT, CD244, SHC3, KIR2DL5A, CHP2, ULBP3, ULBP2, ULBP1, SH2D1B, RAET1E, RAET1L, NCR3, RAET1G, SHC4, MICA

Predefined gene sets in Hepamine

Data source	Pathways	Symbols
KEGG	Neuroactive Ligand Receptor Interaction	ADCYAP1R1, ADORA1, ADORA2A, ADORA2B, ADORA3, ADRA1D, ADRA1B, ADRA1A, ADRA2A, ADRA2B, ADRA2C, ADRB1, ADRB2, ADRB3, AGTR1, AGTR2, APLNR, AVPR1A, AVPR1B, AVPR2, BDKRB1, BDKRB2, BRS3, TSPO, C3AR1, C5AR1, CALCR, CCKAR, CCKBR, CGA, CHRM1, CHRM2, CHRM3, CHRM4, CHRM5, CHRNA1, CHRNA2, CHRNA3, CHRNA4, CHRNA5, CHRNA7, CHRNB1, CHRNB2, CHRNB3, CHRNB4, CHRND, CHRNE, CHRNG, LTB4R, CNR1, CNR2, CRHR1, CRHR2, CSH1, CTSG, DRD1, DRD2, DRD3, DRD4, DRD5, S1PR1, LPAR1, S1PR3, EDNRA, EDNRB, F2, F2R, F2RL1, F2RL2, FPR1, FPR2, FPR3, FSHB, FSHR, GABBR1, GABRA1, GABRA2, GABRA3, GABRA4, GABRA5, GABRA6, GABRB1, GABRB2, GABRB3, GABRD, GABRE, GABRG1, GABRG2, GABRG3, GABRP, GABRR1, GABRR2, GALR1, GCGR, GH1, GH2, GHR, GHRHR, GHSR, GIPR, GLP1R, GLRA1, GLRA2, GLRB, GNRHR, NPBWR1, NPBWR2, PRLHR, UTS2R, LPAR4, MCHR1, GPR35, MLNR, GRIA1, GRIA2, GRIA3, GRIA4, GRID1, GRID2, GRIK1, GRIK2, GRIK3, GRIK4, GRIK5, GRIN1, GRIN2A, GRIN2B, GRIN2C, GRIN2D, NR3C1, GRM1, GRM2, GRM3, GRM4, GRM5, GRM6, GRM7, GRM8, GRPR, GZMA, HCRTR1, HCRTR2, HRH1, HRH2, HTR1A, HTR1B, HTR1D, HTR1E, HTR1F, HTR2A, HTR2B, HTR2C, HTR4, HTR5A, HTR6, HTR7, LEP, LEPR, LHB, LHCGR, MAS1, MC1R, MC2R, MC3R, MC4R, MC5R, MTNR1A, MTNR1B, NMBR, NPY1R, NPY2R, NPY5R, NTSR1, OPRD1, OPRK1, OPRL1, OPRM1, OXTR, P2RX1, P2RX3, P2RX4, P2RX5, P2RX7, P2RY1, P2RY2, P2RY4, P2RY6, P2RY11, PLG, NPY4R, PRL, PRLR, PRSS1, PRSS2, PRSS3, PTAFR, PTGDR, PTGER1, PTGER2, PTGER3, PTGER4, PTGFR, PTGIR, PTH1R, PTH2R, SCTR, SSTR1, SSTR2, SSTR3, SSTR4, SSTR5, TACR2, TACR1, TACR3, TBXA2R, THRA, THRB, TRHR, TSHB, TSHR, VIPR1, VIPR2, TRPV1, GLRA3, GALR3, S1PR4, GALR2, CHRNA6, F2RL3, TAAR5, P2RX6, LPAR2, GPR50, TAAR2, S1PR2, GLP2R, GABBR2, P2RY14, LPAR6, CALCRL, NMUR1, CYSLTR1, NPFFR2, GPR83, HRH3, P2RX2, LPAR3, NTSR2, P2RY10, S1PR5, P2RY13, CHRNA9, GABRQ, PARD3, LTB4R2, NMUR2, CHRNA10, CYSLTR2, HRH4, RXFP1, NPFFR1, TAAR8, MCHR2, KISS1R, GRIN3A, GRIN3B, RXFP2, TAAR9, TAAR1

Predefined gene sets in Hepamine

Data source	Pathways	Symbols
KEGG	Neurotrophin Signaling Pathway	ABL1, AKT1, AKT2, FASLG, RHOA, ARHGDIA, ARHGDIB, ATF4, BAD, BAX, BCL2, BDNF, BRAF, CALM1, CALM2, CALM3, CALML3, CAMK4, CAMK2A, CAMK2B, CAMK2D, CAMK2G, CDC42, CRK, CRKL, MAPK14, CSK, FOXO3, GAB1, GRB2, RAPGEF1, GSK3B, HRAS, IKBKB, IRAK1, IRAK2, IRS1, JUN, KRAS, MAP3K1, MAP3K3, MAP3K5, NFKB1, NFKBIA, NFKBIB, NFKBIE, NGF, NGFR, NRAS, NTF3, NTF4, NTRK1, NTRK2, NTRK3, PDPK1, PIK3CA, PIK3CB, PIK3CD, PIK3CG, PIK3R1, PIK3R2, PLCG1, PLCG2, PRKCD, MAPK1, MAPK3, MAPK7, MAPK8, MAPK11, MAPK9, MAPK10, MAPK13, MAP2K1, MAP2K2, MAP2K5, MAP2K7, PSEN1, PTPN11, RAC1, RAF1, RAP1A, RAP1B, RELA, RPS6KA1, RPS6KA2, RPS6KA3, SORT1, MAPK12, SHC1, SOS1, SOS2, TP53, TP73, TRAF6, YWHAB, YWHAE, YWHAG, YWHAH, YWHAZ, IRS4, PIK3R3, IRS2, RIPK2, RPS6KA4, RPS6KA5, MAPKAPK2, MAGED1, AKT3, SH2B3, SH2B2, ZNF274, FRS2, YWHAQ, PRDM4, IRAK3, PIK3R5, SHC2, SH2B1, BEX3, RPS6KA6, IRAK4, CALML5, SHC3, KIDINS220, CALML6, SHC4
KEGG	N Glycan Biosynthesis	DAD1, DDOST, DPAGT1, FUT8, B4GALT1, STT3A, MAN1A1, MAN2A2, MAN2A1, MGAT1, MGAT2, MGAT3, MGAT5, RPN1, RPN2, ST6GAL1, MOGS, TUSC3, B4GALT3, B4GALT2, DPM1, DPM2, ALG3, MAN1A2, MAN1B1, MGAT4B, MGAT4A, GANAB, ALG5, ALG6, DPM3, ALG1, MAN1C1, DOLPP1, ALG8, ALG12, ALG9, ALG13, ALG10, ALG2, RFT1, ALG10B, MGAT5B, ALG14, STT3B, ALG11
KEGG	Nicotinate And Nicotinamide Metabolism	AOX1, BST1, CD38, NNMT, PNP, NT5E, ENPP1, ENPP3, NAMPT, NT5C2, NMNAT2, QPRT, NNT, NT5C, NT5C3A, NMRK1, NADSYN1, NT5M, NMNAT1, NADK, NUDT12, NT5C1A, NT5C1B, NMNAT3
KEGG	Nitrogen Metabolism	AMT, ASNS, CA1, CA2, CA3, CA4, CA5A, CA6, CA7, CA8, CA9, CA12, CPS1, CTH, GLS, GLUD1, GLUD2, GLUL, HAL, CA5B, CA14, GLS2, CA13

Predefined gene sets in Hepamine

Data source	Pathways	Symbols
KEGG	Nod Like Receptor Signaling Pathway	BIRC2, BIRC3, XIAP, CASP1, CASP5, CASP8, CHUK, MAPK14, CXCL1, CXCL2, HSP90AA1, HSP90AB1, IKBKB, IL1B, IL6, CXCL8, IL18, MEFV, NAIP, NFKB1, NFKBIA, NFKBIB, MAPK1, MAPK3, MAPK8, MAPK11, MAPK9, MAPK10, MAPK13, RELA, MAPK12, CCL2, CCL5, CCL7, CCL8, CCL11, CCL13, MAP3K7, TNF, TNFAIP3, HSP90B1, TRAF6, TRIP6, IKBKG, RIPK2, PSTPIP1, NOD1, TAB1, SUGT1, NLRP1, CARD8, TAB2, PYCARD, ERBIN, NLRC4, CARD18, NOD2, CARD9, CARD6, NLRP3, TAB3, PYDC1
KEGG	Non Homologous End Joining	DNTT, FEN1, XRCC6, LIG4, MRE11, PRKDC, XRCC4, XRCC5, RAD50, POLL, POLM, DCLRE1C, NHEJ1, LOC731751
KEGG	Non Small Cell Lung Cancer	AKT1, AKT2, ARAF, BAD, CCND1, BRAF, CASP9, CDK4, CDK6, CDKN2A, E2F1, E2F2, E2F3, EGF, EGFR, ERBB2, FHIT, FOXO3, GRB2, HRAS, KRAS, NRAS, PDPK1, PIK3CA, PIK3CB, PIK3CD, PIK3CG, PIK3R1, PIK3R2, PLCG1, PLCG2, PRKCA, PRKCB, PRKCG, MAPK1, MAPK3, MAP2K1, MAP2K2, RAF1, RARB, RB1, RXRA, RXRB, RXRG, SOS1, SOS2, STK4, TGFA, TP53, PIK3R3, AKT3, RASSF1, PIK3R5, RASSF5
KEGG	Notch Signaling Pathway	JAG1, CREBBP, CTBP1, CTBP2, DTX1, DVL1, DVL2, DVL3, EP300, KAT2A, HDAC1, HDAC2, HES1, RBPJ, JAG2, LFNG, MFNG, NOTCH1, NOTCH2, NOTCH3, NOTCH4, PSEN1, PSEN2, RFNG, ADAM17, NUMB, KAT2B, NUMBL, CIR1, NCOR2, MAML1, DLL3, RBPJL, SNW1, DTX4, NCSTN, DLL1, APH1A, DLL4, MAML3, PSENEN, MAML2, DTX2, DTX3L, PTCRA, DTX3, HES5
KEGG	Nucleotide Excision Repair	CCNH, CDK7, CETN2, ERCC8, DDB1, DDB2, ERCC1, ERCC2, ERCC3, ERCC4, ERCC5, ERCC6, GTF2H1, GTF2H2, GTF2H3, GTF2H4, LIG1, MNAT1, PCNA, POLD1, POLD2, POLE, POLE2, RAD23A, RAD23B, RFC1, RFC2, RFC3, RFC4, RFC5, RPA1, RPA2, RPA3, XPA, XPC, CUL4B, CUL4A, RBX1, POLD3, RPA4, POLE3, POLE4, POLD4, GTF2H5

Predefined gene sets in Hepamine

Data source	Pathways	Symbols
KEGG	O Glycan Biosynthesis	GALNT1, GALNT2, GALNT3, GCNT1, ST3GAL1, ST3GAL2, GALNT4, GCNT3, B4GALT5, GALNT6, GALNT5, GALNT8, C1GALT1C1, GALNT9, GCNT4, GALNT7, GALNT10, ST6GALNAC1, C1GALT1, GALNT16, GALNT11, GALNT17, GALNT14, GALNT12, GALNT13, GALNT15, GALNTL5, B3GNT6, GALNT18, GALNTL6

**Data source**	**Pathways**	**Symbols**
KEGG	Olfactory Transduction	ADCY3, GRK3, ARRB2, CALM1, CALM2, CALM3, CALML3, CAMK2A, CAMK2B, CAMK2D, CAMK2G, CLCA1, CNGB1, CNGA3, CNGA4, GNAL, GUCA1A, GUCA1B, GUCY2D, OR1D2, OR1F1, OR2C1, OR3A1, OR3A2, PDC, PDE1C, PRKACA, PRKACB, PRKACG, PRKG1, PRKG2, PRKX, OR2H2, OR1A1, OR1D5, OR1E1, OR1E2, OR1G1, OR3A3, OR6A2, GUCA1C, CLCA2, OR5I1, CLCA4, OR52A1, OR1C1, OR1A2, OR2F1, OR2B6, OR1J4, OR2M4, OR2L2, OR2K2, OR7A17, OR5L2, OR5K1, OR10J1, OR8G2P, OR8B8, OR8G1, OR10A3, OR12D2, OR11A1, OR10H3, OR10G3, OR10G2, OR10H2, OR10H1, OR7E24, OR7C2, OR7A5, OR7C1, OR4F4, OR4F3, OR4E2, OR4D1, OR2W1, OR2T1, OR2J2, OR2H1, OR1L3, OR1L1, OR1J2, CALML5, OR2S2, OR13A1, OR5H6, OR5H2, OR4K5, OR51G1, OR51B4, OR51B2, OR52N1, OR4F5, OR2A4, OR4K1, OR5AC2, OR11H1, OR4F17, OR4K15, OR8J3, OR51G2, OR51E2, OR4P4, OR4C15, OR4A5, OR4A16, OR4A15, OR2AE1, OR4F16, OR6N2, OR6K2, OR2G3, OR2G2, OR2C3, OR5V1, OR2B2, OR12D3, OR52E2, OR52J3, OR51L1, OR51A7, OR51S1, OR51F2, OR52R1, OR4C46, OR4X2, OR4B1, OR52M1, OR52K2, OR5P2, OR5P3, OR8I2, OR2D3, OR2D2, OR52W1, OR56A4, OR56A1, OR10P1, OR10AD1, OR10A7, OR4K14, OR4L1, OR11H6, OR4D2, OR7D4, OR7G1, OR1M1, OR1I1, OR10H4, OR2M5, OR2M3, OR2T12, OR14C36, OR2T34, OR2T10, OR2T4, OR2T11, OR10J5, OR2B11, OR10T2, OR10X1, OR10Z1, OR6K6, OR6N1, OR9A4, OR2Y1, OR9A2, OR2A14, OR6B1, OR2F2, OR13C5, OR13C8, OR13C3, OR13C4, OR13F1, OR1L8, OR1N2, OR1N1, OR52B4, OR52I2, OR51E1, OR10A5, OR2AG1, OR6B3, OR1Q1, OR7D2, CALML6, OR56B4, OR8U1, OR4C16, OR4C11, OR4S2, OR4C6, OR5D14, OR5L1, OR5D18, OR5AS1, OR8K5, OR5T2, OR8H1, OR8K3, OR8J1, OR5R1, OR5M3, OR5M8, OR5M11, OR5AR1, OR8B12, OR8G5, OR10G8, OR10G9, OR10S1, OR6T1, OR4D5, OR6Q1, OR9I1, OR9Q1, OR9Q2, OR1S2, OR1S1, OR10Q1, OR5B17, OR5B21, OR5A2, OR5A1, OR4D6, OR4D11, OR6C74, OR6C3, OR2T6, OR1L4, OR52B2, OR4C3, OR4S1, OR51F1, OR51B5, OR10AG1, OR5J2, OR4C13, OR4C12, OR51V1, OR8D1, OR8D2, OR8B4, OR9G4, OR10A4, OR6C6,

Predefined gene sets in Hepamine

Data source	Pathways	Symbols
KEGG	One Carbon Pool By Folate	AMT, ATIC, DHFR, GART, MTHFD1, MTHFR, MTR, SHMT1, SHMT2, TYMS, MTHFS, MTHFD2, ALDH1L1, FTCD, MTHFD1L, MTFMT, MTHFD2L
KEGG	Oocyte Meiosis	ADCY1, ADCY2, ADCY3, ADCY5, ADCY6, ADCY7, ADCY8, ADCY9, AR, BUB1, CALM1, CALM2, CALM3, CALML3, CAMK2A, CAMK2B, CAMK2D, CAMK2G, CCNB1, CCNE1, CDK1, CDC20, CDC25C, CDC27, CDK2, IGF1, IGF1R, INS, ITPR1, ITPR2, ITPR3, MAD2L1, MOS, PGR, PLK1, PPP1CA, PPP1CB, PPP1CC, PPP2CA, PPP2CB, PPP2R1A, PPP2R1B, PPP2R5A, PPP2R5B, PPP2R5C, PPP2R5D, PPP2R5E, PPP3CA, PPP3CB, PPP3CC, PPP3R1, PPP3R2, PRKACA, PRKACB, PRKACG, MAPK1, MAPK3, MAP2K1, PRKX, RPS6KA1, RPS6KA2, RPS6KA3, MAPK12, SKP1, AURKA, YWHAB, YWHAE, YWHAG, YWHAH, YWHAZ, SMC1A, CUL1, CDC23, CDC16, BTRC, PKMYT1, SMC3, CCNB2, CCNE2, PTTG1, ESPL1, SLK, RBX1, REC8, ANAPC10, MAD2L2, STAG3, PTTG2, YWHAQ, CHP1, FBXW11, ANAPC13, FBXO5, SMC1B, RPS6KA6, ANAPC2, ANAPC4, ANAPC5, ANAPC7, ANAPC11, CALML5, CHP2, CPEB1, ANAPC1, PLCZ1, SGO1, CALML6, ADCY4, SPDYA, CDC26, FBXO43, SPDYC, LOC650621, SKP1P2
KEGG	Other Glycan Degradation	AGA, FUCA1, FUCA2, GBA, GLB1, HEXA, HEXB, MAN2C1, MAN2B1, MANBA, NEU1, NEU2, NEU3, MAN2B2, ENGASE, NEU4

Predefined gene sets in Hepamine

Data source	Pathways	Symbols
KEGG	Oxidative Phosphorylation	ATP12A, ATP4A, ATP4B, ATP5F1A, ATP5F1B, ATP5F1C, ATP5F1D, ATP5F1E, ATP5PB, ATP5MC1, ATP5MC2, ATP5MC3, ATP5ME, ATP5PF, ATP6V1A, ATP6V1B1, ATP6V1B2, ATP6V0C, ATP6V1C1, ATP6V1E1, ATP6V0B, ATP6V1G2, ATP6V0A1, ATP6AP1, ATP5PO, COX4I1, COX5B, COX6A1, COX6A2, COX6B1, COX6C, COX7A1, COX7A2, COX7B, COX7C, COX8A, COX10, COX11, COX15, CYC1, ATP6, ATP8, COX1, COX2, COX3, CYTB, ND1, ND2, ND3, ND4, ND4L, ND5, ND6, NDUFA1, NDUFA2, NDUFA3, NDUFA4, NDUFA5, NDUFA6, NDUFA7, NDUFA8, NDUFA9, NDUFA10, NDUFAB1, NDUFB1, NDUFB2, NDUFB3, NDUFB4, NDUFB5, NDUFB6, NDUFB7, NDUFB8, NDUFB9, NDUFB10, NDUFC1, NDUFC2, NDUFS1, NDUFS2, NDUFS3, NDUFV1, NDUFS4, NDUFS5, NDUFS6, NDUFS8, NDUFV2, NDUFV3, PPA1, SDHA, SDHB, SDHC, SDHD, UQCRB, UQCRC1, UQCRC2, UQCRFS1, UQCRH, ATP6V0E1, ATP6V0D1, COX7A2L, ATP6V1F, COX5A, ATP6V1G1, ATP5MF, COX17, TCIRG1, ATP5PD, ATP5MG, UQCR11, ATP6V0A2, PPA2, UQCRQ, UQCR10, ATP6V0A4, ATP6V1D, ATP6V1H, NDUFA4L2, LHPP, COX4I2, ATP6V1E2, COX6B2, NDUFA11, ATP6V1G3, ATP6V0E2, COX7B2, ATP6V0D2, ATP6V1C2, COX8C, NDUFS7, ATP5MC1P5, UQCRHL, LOC642502, LOC644310, LOC727947, COX6CP3, LOC100133737
KEGG	P53 Signaling Pathway	APAF1, FAS, ATM, ATR, ADGRB1, BAX, CCND1, BID, CASP3, CASP8, CASP9, CCNB1, CCND2, CCND3, CCNE1, CCNG1, CCNG2, CDK1, CDK2, CDK4, CDK6, CDKN1A, CDKN2A, CHEK1, DDB2, GADD45A, SFN, IGF1, IGFBP3, CD82, MDM2, MDM4, GADD45B, SERPINE1, SERPINB5, PMAIP1, PTEN, RRM2, SIAH1, THBS1, TP53, TP73, TSC2, PPM1D, TNFRSF10B, CCNB2, CCNE2, EI24, TP53I3, GADD45G, CHEK2, RCHY1, BBC3, SESN1, RRM2B, SHISA5, GTSE1, CYCS, STEAP3, PIDD1, RPRM, TP53AIP1, PERP, COP1, ZMAT3, SESN2, CCNB3, SESN3, LOC651610

Predefined gene sets in Hepamine

Data source	Pathways	Symbols
KEGG	Pancreatic Cancer	AKT1, AKT2, ARAF, BAD, CCND1, BCL2L1, BRAF, BRCA2, CASP9, CDC42, CDK4, CDK6, CDKN2A, CHUK, E2F1, E2F2, E2F3, EGF, EGFR, ERBB2, VEGFD, IKBKB, JAK1, KRAS, SMAD2, SMAD3, SMAD4, NFKB1, PGF, PIK3CA, PIK3CB, PIK3CD, PIK3CG, PIK3R1, PIK3R2, PLD1, MAPK1, MAPK3, MAPK8, MAPK9, MAPK10, MAP2K1, RAC1, RAC2, RAC3, RAD51, RAF1, RALA, RALB, RALGDS, RB1, RELA, STAT1, STAT3, TGFA, TGFB1, TGFB2, TGFB3, TGFBR1, TGFBR2, TP53, VEGFA, VEGFB, VEGFC, PIK3R3, IKBKG, ARHGEF6, AKT3, RALBP1, PIK3R5
KEGG	Pantothenate And Coa Biosynthesis	BCAT1, BCAT2, DPYD, DPYS, ENPP1, ENPP3, VNN2, VNN1, UPB1, PANK1, PANK4, PPCDC, PANK3, PPCS, PANK2, COASY
KEGG	Parkinsons Disease	SLC25A4, SLC25A5, SLC25A6, APAF1, ATP5F1A, ATP5F1B, ATP5F1C, ATP5F1D, ATP5F1E, ATP5PB, ATP5MC1, ATP5MC2, ATP5MC3, ATP5PF, ATP5PO, CASP3, CASP9, COX4I1, COX5B, COX6A1, COX6A2, COX6B1, COX6C, COX7A1, COX7A2, COX7B, COX7C, COX8A, CYC1, GPR37, ATP6, ATP8, COX1, COX2, COX3, CYTB, ND1, ND2, ND3, ND4, ND4L, ND5, ND6, NDUFA1, NDUFA2, NDUFA3, NDUFA4, NDUFA5, NDUFA6, NDUFA7, NDUFA8, NDUFA9, NDUFA10, NDUFAB1, NDUFB1, NDUFB2, NDUFB3, NDUFB4, NDUFB5, NDUFB6, NDUFB7, NDUFB8, NDUFB9, NDUFB10, NDUFC1, NDUFC2, NDUFS1, NDUFS2, NDUFS3, NDUFV1, NDUFS4, NDUFS5, NDUFS6, NDUFS8, NDUFV2, NDUFV3, PRKN, SEPT5, PPID, SDHA, SDHB, SDHC, SDHD, SLC6A3, SLC18A1, SLC18A2, SNCA, TH, UBB, UBA1, UBA7, UBE2G1, UBE2G2, UBE2L3, UCHL1, UQCRB, UQCRC1, UQCRC2, UQCRFS1, UQCRH, VDAC1, VDAC2, VDAC3, COX7A2L, UBE2L6, COX5A, SNCAIP, ATP5PD, UQCR11, PARK7, UQCRQ, HTRA2, UQCR10, UBE2J1, CYCS, NDUFA4L2, PINK1, SLC25A31, COX4I2, UBE2J2, LRRK2, COX6B2, COX7B2, COX8C, NDUFS7, ATP5MC1P5, UQCRHL, LOC642502, LOC644310, LOC727947, LOC729317, COX6CP3, LOC100133737

Predefined gene sets in Hepamine

**Data source**	**Pathways**	**Symbols**
KEGG	Pathogenic Escherichia Coli Infection	ABL1, ACTB, ACTG1, RHOA, CD14, CDC42, CDH1, CTNNB1, CTTN, FYN, HCLS1, ITGB1, KRT18, NCK1, NCL, PRKCA, ROCK1, TLR4, TLR5, TUBA4A, TUBA3C, TUBB2A, EZR, WAS, YWHAZ, TUBA1A, NCK2, WASL, CLDN1, ARHGEF2, ROCK2, ARPC5, ARPC4, ARPC3, ARPC1B, ARPC2, TUBA1B, TUBB3, TUBB4A, TUBB4B, ARPC1A, YWHAQ, LY96, TUBA8, TUBB7P, TUBAL3, TUBB1, ARPC5L, TUBB6, TUBA1C, TUBA3E, TUBA3D, TUBB, TUBB8, TUBB2B, LOC646821, LOC653888, LOC100418883, OCLN

Predefined gene sets in Hepamine

Data source	Pathways	Symbols
KEGG	Pathways In Cancer	ABL1, AKT1, AKT2, APC, BIRC2, BIRC3, XIAP, BIRC5, KLK3, FAS, FASLG, AR, ARAF, RHOA, ARNT, BAD, BAX, CCND1, BCL2, BCL2L1, BCR, BID, BMP2, BMP4, BRAF, BRCA2, CASP3, CASP8, CASP9, RUNX1, RUNX1T1, CBL, CBLB, CCNE1, CDC42, CDH1, CDK2, CDK4, CDK6, CDKN1A, CDKN1B, CDKN2A, CDKN2B, CEBPA, CHUK, CKS1B, COL4A1, COL4A2, COL4A4, COL4A6, CREBBP, CRK, CRKL, CSF1R, CSF2RA, CSF3R, CTBP1, CTBP2, CTNNA1, CTNNA2, CTNNB1, DAPK1, DAPK3, DCC, DVL1, DVL2, DVL3, E2F1, E2F2, E2F3, EGF, EGFR, EP300, EPAS1, ERBB2, ETS1, MECOM, FGF1, FGF2, FGF3, FGF4, FGF5, FGF6, FGF7, FGF8, FGF9, FGF10, FGF11, FGF12, FGF13, FGF14, FGFR1, FGFR3, FGFR2, FH, VEGFD, FOXO1, FLT3, FLT3LG, FN1, FOS, MTOR, FZD2, GLI1, GLI2, GLI3, GRB2, GSK3B, GSTP1, MSH6, HDAC1, HDAC2, HGF, HIF1A, HRAS, HSP90AA1, HSP90AB1, IGF1, IGF1R, IKBKB, IL6, CXCL8, ITGA6, ITGA2, ITGA2B, ITGA3, ITGAV, ITGB1, JAK1, JUN, JUP, KIT, KRAS, LAMA2, LAMA3, LAMA4, LAMA5, LAMB1, LAMB2, LAMB3, LAMC1, LAMC2, SMAD2, SMAD3, SMAD4, MAX, MDM2, MET, KITLG, MITF, MLH1, MMP1, MMP2, MMP9, MSH2, MSH3, MYC, NFKB1, NFKB2, NFKBIA, NKX3-1, NOS2, NRAS, NTRK1, PDGFA, PDGFB, PDGFRA, PDGFRB, PGF, PIK3CA, PIK3CB, PIK3CD, PIK3CG, PIK3R1, PIK3R2, PLCG1, PLCG2, PLD1, PML, PPARD, PPARG, PRKCA, PRKCB, PRKCG, MAPK1, MAPK3, MAPK8, MAPK9, MAPK10, MAP2K1, MAP2K2, PTCH1, PTEN, PTGS2, PTK2, RAC1, RAC2, RAC3, RAD51, RAF1, RALA, RALB, RALGDS, RARA, RARB, RB1, RELA, RET, RXRA, RXRB, RXRG, SHH, SKP2, SLC2A1, SMO, SOS1, SOS2, SPI1, STAT1, STAT3, STAT5A, STAT5B, STK4, ELOC, ELOB, TCF7, TCF7L2, TGFA, TGFB1, TGFB2, TGFB3, TGFBR1, TGFBR2, TP53, TPM3, TPR, HSP90B1, TRAF1, TRAF2, TRAF3, TRAF5, TRAF6, VEGFA, VEGFB, VEGFC, VHL, WNT1, WNT2, WNT3, WNT5A, WNT6, WNT7A, WNT7B, WNT8A, WNT8B, WNT10B, WNT11, WNT2B, WNT9A, WNT9B, ZBTB16, PAX8, FZD5, FZD3, CCDC6, NCOA4, FGF23, AXIN1, AXIN2, FZD1, FZD4, FZD6, FZD7, FZD8, FZD9, CUL2, PIK3R3, IKBKG, PIAS1, PTCH2, FADD, FGF18, FGF17, FGF16, CCNA1, PIAS2, CCNE2, TRAF4, ARNT2, FGF19, RBX1 [illegible]

Predefined gene sets in Hepamine

Data source	Pathways	Symbols
KEGG	Pentose And Glucuronate Interconversions	AKR1B1, GUSB, RPE, UGDH, UGP2, UGT2B4, UGT2B7, UGT2B10, UGT2B15, UGT2B17, XYLB, UGT2B11, UGT2A1, DHDH, CRYL1, DCXR, UGT2B28, UGT1A10, UGT1A8, UGT1A7, UGT1A6, UGT1A5, UGT1A9, UGT1A4, UGT1A1, UGT1A3, UGT2A3, RPEL1
KEGG	Pentose Phosphate Pathway	ALDOA, ALDOB, ALDOC, FBP1, G6PD, GPI, PFKL, PFKM, PFKP, PGD, PGM1, PRPS1, PRPS2, RPE, TALDO1, TKT, TKTL1, FBP2, H6PD, RPIA, PGLS, DERA, PGM2, RBKS, TKTL2, PRPS1L1, RPEL1
KEGG	Peroxisome	ACAA1, ACOX1, AGXT, ABCD1, ABCD2, BAAT, CAT, CRAT, DAO, ECH1, EHHADH, EPHX2, ACSL1, ACSL3, ACSL4, HMGCL, HSD17B4, IDH1, IDH2, MPV17, MVK, NOS2, PRDX1, PEX1, PEX6, PEX7, PEX10, PEX12, PEX13, PEX14, PHYH, PEX19, ABCD3, ABCD4, PXMP2, PEX2, PEX5, SCP2, SOD1, SOD2, XDH, ACOX2, ACOX3, GNPAT, PEX3, DDO, AGPS, PEX11B, PEX11A, PEX16, ACOT8, ECI2, SLC25A17, PMVK, DHRS4, SLC27A2, PXMP4, ACSL6, MLYCD, AMACR, PRDX5, HACL1, DECR2, HAO2, PIPOX, ACSL5, HAO1, CROT, PEX26, FAR2, PECR, NUDT12, FAR1, PEX11G, PAOX, MPV17L, GSTK1, NUDT19
KEGG	Phenylalanine Metabolism	ALDH3A1, ALDH1A3, ALDH3B1, ALDH3B2, AOC2, DDC, GOT1, GOT2, HPD, MAOA, MAOB, MIF, PAH, TAT, AOC3, PRDX6, NAA80, IL4I1

Predefined gene sets in Hepamine

**Data source**	**Pathways**	**Symbols**
KEGG	Phosphatidylinositol Signaling System	CALM1, CALM2, CALM3, CALML3, CDS1, DGKA, DGKB, DGKG, DGKQ, IMPA1, IMPA2, INPP1, INPP4A, INPP5A, INPP5B, INPP5D, INPPL1, ITPK1, ITPKA, ITPKB, ITPR1, ITPR2, ITPR3, OCRL, PIK3C2A, PIK3C2B, PIK3C2G, PIK3C3, PIK3CA, PIK3CB, PIK3CD, PIK3CG, PIK3R1, PIK3R2, PI4KA, PI4KB, PIP4K2A, PLCB2, PLCB3, PLCB4, PLCD1, PLCG1, PLCG2, PRKCA, PRKCB, PRKCG, PTEN, PIP5K1A, PIP5K1B, PIP4K2B, PIK3R3, DGKZ, DGKE, DGKD, CDS2, INPP4B, SYNJ1, SYNJ2, DGKI, CDIPT, PLCB1, PIP5K1C, PIK3R5, INPP5J, PLCE1, INPP5K, CALML5, INPP5E, IPPK, PIP4K2C, PLCD4, PLCZ1, PLCD3, DGKH, CALML6, PIKFYVE
KEGG	Porphyrin And Chlorophyll Metabolism	ALAD, ALAS1, ALAS2, BLVRA, BLVRB, COX10, COX15, CP, CPOX, EPRS, FECH, FTH1, GUSB, HCCS, HMBS, HMOX1, HMOX2, PPOX, UGT2B4, UGT2B7, UGT2B10, UGT2B15, UGT2B17, UROD, UROS, UGT2B11, UGT2A1, UGT2B28, UGT1A10, UGT1A8, UGT1A7, UGT1A6, UGT1A5, UGT1A9, UGT1A4, UGT1A1, UGT1A3, UGT2A3, FTMT, EARS2, MMAB
KEGG	Ppar Signaling Pathway	ACAA1, ACADL, ACADM, ACOX1, APOA1, APOA2, APOC3, AQP7, CD36, CPT1A, CPT1B, CPT2, CYP4A11, CYP7A1, CYP8B1, CYP27A1, DBI, EHHADH, FABP4, FABP1, FABP2, FABP3, FABP5, FABP6, FABP7, ACSL1, ACSL3, ACSL4, GK, GK2, HMGCS2, ILK, LPL, ME1, MMP1, OLR1, PCK1, PCK2, PDPK1, PLIN1, PLTP, PPARA, PPARD, PPARG, RXRA, RXRB, RXRG, SCD, SCP2, UBC, UCP1, ACOX2, ACOX3, ADIPOQ, FADS2, NR1H3, SORBS1, SLC27A5, SLC27A4, SLC27A2, ACSL6, SLC27A6, ANGPTL4, ACSL5, SCD5, APOA5, CPT1C, CYP4A22, SLC27A1
KEGG	Primary Bile Acid Biosynthesis	BAAT, AKR1C4, CYP7A1, CYP8B1, CYP27A1, HSD17B4, SCP2, AKR1D1, ACOX2, CH25H, CYP7B1, CYP46A1, SLC27A5, AMACR, CYP39A1, HSD3B7

Predefined gene sets in Hepamine

**Data source**	**Pathways**	**Symbols**
KEGG	Primary Immunodeficiency	ADA, AIRE, BTK, CD3D, CD3E, CD4, CD8A, CD8B, CD19, CD40, CD40LG, CD79A, IGLL1, IL2RG, IL7R, JAK3, LCK, CIITA, PTPRC, RAG1, RAG2, RFX5, RFXAP, TAP1, TAP2, UNG, ZAP70, IKBKG, RFXANK, TNFRSF13B, BLNK, ICOS, AICDA, DCLRE1C, TNFRSF13C
KEGG	Prion Diseases	BAX, C1QA, C1QB, C1QC, C5, C6, C7, C8A, C8B, C8G, C9, EGR1, ELK1, FYN, HSPA1A, HSPA5, IL1A, IL1B, IL6, LAMC1, NCAM1, NCAM2, NOTCH1, PRKACA, PRKACB, PRKACG, MAPK1, MAPK3, MAP2K1, MAP2K2, PRKX, PRNP, CCL5, SOD1, STIP1
KEGG	Progesterone Mediated Oocyte Maturation	ADCY1, ADCY2, ADCY3, ADCY5, ADCY6, ADCY7, ADCY8, ADCY9, AKT1, AKT2, ARAF, BRAF, BUB1, CCNA2, CCNB1, CDK1, CDC25A, CDC25B, CDC25C, CDC27, CDK2, MAPK14, GNAI1, GNAI2, GNAI3, HSP90AA1, HSP90AB1, IGF1, IGF1R, INS, KRAS, MAD2L1, MOS, PDE3A, PDE3B, PGR, PIK3CA, PIK3CB, PIK3CD, PIK3CG, PIK3R1, PIK3R2, PLK1, PRKACA, PRKACB, PRKACG, MAPK1, MAPK3, MAPK8, MAPK11, MAPK9, MAPK10, MAPK13, MAP2K1, PRKX, RAF1, RPS6KA1, RPS6KA2, RPS6KA3, MAPK12, PIK3R3, CDC23, CDC16, CCNA1, PKMYT1, CCNB2, AKT3, ANAPC10, MAD2L2, PIK3R5, ANAPC13, RPS6KA6, ANAPC2, ANAPC4, FZR1, ANAPC5, ANAPC7, ANAPC11, CPEB1, ANAPC1, CCNB3, ADCY4, SPDYA, CDC26, SPDYC, LOC650621
KEGG	Propanoate Metabolism	ABAT, ACACA, ACACB, ACADM, ACAT1, ACAT2, ALDH2, ALDH1B1, ALDH9A1, ALDH3A2, ALDH7A1, ECHS1, EHHADH, HADHA, LDHA, LDHB, LDHC, ALDH6A1, MMUT, PCCA, PCCB, SUCLG2, SUCLG1, SUCLA2, MLYCD, HIBCH, ACSS2, ACSS3, ACSS1, MCEE, LDHAL6B, LDHAL6A, SUCLG2P2

Predefined gene sets in Hepamine

Data source	Pathways	Symbols
KEGG	Prostate Cancer	AKT1, AKT2, KLK3, AR, ARAF, ATF4, BAD, CCND1, BCL2, BRAF, CASP9, CCNE1, CDK2, CDKN1A, CDKN1B, CHUK, CREB1, CREBBP, CTNNB1, E2F1, E2F2, E2F3, EGF, EGFR, EP300, ERBB2, FGFR1, FGFR2, FOXO1, MTOR, GRB2, GSK3B, GSTP1, HRAS, HSP90AA1, HSP90AB1, IGF1, IGF1R, IKBKB, INS, INSRR, KRAS, MDM2, NFKB1, NFKBIA, NKX3-1, NRAS, PDGFA, PDGFB, PDGFRA, PDGFRB, PDPK1, PIK3CA, PIK3CB, PIK3CD, PIK3CG, PIK3R1, PIK3R2, MAPK1, MAPK3, MAP2K1, MAP2K2, PTEN, RAF1, RB1, RELA, SOS1, SOS2, SRD5A2, TCF7, TCF7L2, TGFA, TP53, HSP90B1, PIK3R3, IKBKG, CCNE2, CREB5, AKT3, CREB3, PIK3R5, LEF1, PDGFC, CREB3L2, PDGFD, TCF7L1, CREB3L3, CREB3L1, CREB3L4
KEGG	Proteasome	IFNG, PSMA1, PSMA2, PSMA3, PSMA4, PSMA5, PSMA6, PSMA7, PSMB1, PSMB2, PSMB3, PSMB4, PSMB5, PSMB6, PSMB7, PSMB8, PSMB9, PSMB10, PSMC1, PSMC2, PSMC3, PSMC4, PSMC5, PSMC6, PSMD1, PSMD2, PSMD3, PSMD4, PSMD7, PSMD8, PSMD11, PSMD12, PSMD13, PSME1, PSME2, SEM1, PSMF1, PSMD6, PSME3, PSMD14, PSME4, POMP, PSMA6P4, PSMB11, PSMA8, PSMC1P4, LOC652826, LOC100132108
KEGG	Protein Export	HSPA5, OXA1L, SRP9, SRP14, SRP19, SRP54, SRP68, SRP72, SRPRA, SEC62, SPCS2, SEC61B, SEC63, SEC11A, SEC61G, SPCS1, SEC61A1, SEC61A2, SRPRB, SPCS3, IMMP2L, SEC11C, IMMP1L, SRP9P1
KEGG	Proximal Tubule Bicarbonate Reclamation	AQP1, ATP1A1, ATP1A2, ATP1A3, ATP1A4, ATP1B1, ATP1B2, ATP1B3, FXYD2, CA2, CA4, SLC25A10, GLS, GLUD1, GLUD2, MDH1, PCK1, PCK2, SLC9A3, SLC4A4, SLC38A3, ATP1B4, GLS2

Predefined gene sets in Hepamine

Data source	Pathways	Symbols
KEGG	Purine Metabolism	ADA, ADCY1, ADCY2, ADCY3, ADCY5, ADCY6, ADCY7, ADCY8, ADCY9, ADK, ADSL, ADSS, AK1, AK2, AK4, AMPD1, AMPD2, AMPD3, NUDT2, APRT, ATIC, POLR3D, ENTPD1, ENTPD2, ENTPD6, ENTPD3, ENTPD5, DCK, DGUOK, FHIT, GART, GMPR, GUCY1A2, GUCY1A1, GUCY1B1, GUCY2C, GUCY2F, GUK1, GUCY2D, HPRT1, IMPDH1, IMPDH2, ITPA, NME1, NME2, NME3, NME4, PNP, NPR1, NPR2, NT5E, PDE1A, PDE1C, PDE2A, PDE3A, PDE3B, PDE4A, PDE4B, PDE4C, PDE4D, PDE6A, PDE6C, PDE6D, PDE6G, PDE6H, PDE7A, PDE8A, PDE9A, PDE1B, PDE6B, ENPP1, ENPP3, PFAS, PKLR, PKM, POLA1, POLD1, POLD2, POLE, POLE2, POLR2A, POLR2B, POLR2C, POLR2D, POLR2E, POLR2F, POLR2G, POLR2H, POLR2I, POLR2J, POLR2K, POLR2L, PPAT, PRIM1, PRIM2, PRPS1, PRPS2, RRM1, RRM2, XDH, NME5, PDE8B, PDE5A, GMPS, PAPSS2, PAPSS1, POLR1C, ENTPD4, GDA, NME6, PAICS, POLR3F, POLR3G, POLR3C, POLD3, PDE10A, POLR3A, NUDT5, NT5C2, POLA2, POLR1A, AK5, PDE7B, NME7, NT5C, ZNRD1, RRM2B, PDE11A, POLR1D, NT5C3A, GMPR2, POLR3K, NUDT9, POLE3, POLR3B, ADCY10, ALLC, POLE4, NT5M, ADPRM, POLD4, PRUNE1, POLR1E, POLR1B, POLR3GL, NT5C1A, PNPT1, NT5C1B, AK7, ADSSL1, CANT1, POLR3H, ADCY4, PRPS1L1, POLR2J2, ENTPD8, POLR2J3, URAD, NME1-NME2

Predefined gene sets in Hepamine

Data source	Pathways	Symbols
KEGG	Pyrimidine Metabolism	NUDT2, POLR3D, CAD, ENTPD1, ENTPD6, ENTPD3, ENTPD5, CDA, CTPS1, DCK, DCTD, DHODH, DPYD, DPYS, DTYMK, DUT, TYMP, ITPA, NME1, NME2, NME3, NME4, PNP, NT5E, POLA1, POLD1, POLD2, POLE, POLE2, POLR2A, POLR2B, POLR2C, POLR2D, POLR2E, POLR2F, POLR2G, POLR2H, POLR2I, POLR2J, POLR2K, POLR2L, PRIM1, PRIM2, RRM1, RRM2, TK1, TK2, TXNRD1, TYMS, UCK2, UMPS, UPP1, NME5, POLR1C, ENTPD4, NME6, TXNRD2, POLR3F, POLR3G, POLR3C, POLD3, POLR3A, NT5C2, POLA2, POLR1A, NME7, NT5C, ZNRD1, RRM2B, AK3, POLR1D, NT5C3A, CMPK1, POLR3K, UPB1, POLE3, UCKL1, POLR3B, CTPS2, POLE4, NT5M, POLD4, POLR1E, UCK1, POLR1B, POLR3GL, NT5C1A, PNPT1, NT5C1B, CANT1, CMPK2, UPRT, UPP2, POLR3H, POLR2J2, ENTPD8, POLR2J3, NME1-NME2
KEGG	Pyruvate Metabolism	ACACA, ACACB, ACAT1, ACAT2, ACYP1, ACYP2, ALDH2, ALDH1B1, ALDH9A1, ALDH3A2, AKR1B1, ALDH7A1, DLAT, DLD, GLO1, HAGH, LDHA, LDHB, LDHC, MDH1, MDH2, ME1, ME2, PC, PCK1, PCK2, PDHA1, PDHA2, PDHB, PKLR, PKM, GRHPR, ME3, ACSS2, HAGHL, ACSS1, LDHAL6B, ACOT12, LDHAL6A, LDHD

Predefined gene sets in Hepamine

Data source	Pathways	Symbols
KEGG	Regulation Of Actin Cytoskeleton	ACTB, ACTG1, ACTN4, ACTN1, ACTN2, ACTN3, APC, ARAF, RHOA, BDKRB1, BDKRB2, BRAF, CD14, CDC42, CFL1, CFL2, CHRM1, CHRM2, CHRM3, CHRM4, CHRM5, CRK, CRKL, CSK, DIAPH1, DIAPH2, DOCK1, EGF, EGFR, F2, F2R, FGD1, FGF1, FGF2, FGF3, FGF4, FGF5, FGF6, FGF7, FGF8, FGF9, FGF10, FGF11, FGF12, FGF13, FGF14, FGFR1, FGFR3, FGFR2, FGFR4, FN1, GNA12, ARHGAP35, GSN, NCKAP1L, HRAS, INS, INSRR, ITGA6, ITGA1, ITGA2, ITGA2B, ITGA3, ITGA4, ITGA5, ITGA7, ITGA9, ITGAD, ITGAE, ITGAL, ITGAM, ITGAV, ITGAX, ITGB1, ITGB2, ITGB3, ITGB4, ITGB5, ITGB6, ITGB7, ITGB8, KRAS, LIMK1, LIMK2, MOS, MSN, MYH9, MYH10, MYL2, MYL5, MYLK, PPP1R12A, NRAS, PAK1, PAK2, PAK3, PDGFA, PDGFB, PDGFRA, PDGFRB, PFN1, PFN2, PIK3CA, PIK3CB, PIK3CD, PIK3CG, PIK3R1, PIK3R2, PIP4K2A, PPP1CA, PPP1CB, PPP1CC, MAPK1, MAPK3, MAP2K1, MAP2K2, PTK2, PXN, RAC1, RAC2, RAC3, RAF1, RDX, ROCK1, RRAS, SLC9A1, SOS1, SOS2, TIAM1, TMSB4X, TMSB4XP8, VAV1, VAV2, VCL, EZR, WAS, FGF23, PIP5K1A, PIP5K1B, PIP4K2B, PIK3R3, ITGA10, ITGA8, FGF18, FGF17, FGF16, IQGAP1, ARHGEF7, WASF1, WASL, TMSB4Y, ARHGEF1, ARHGEF6, ROCK2, BCAR1, FGF19, ARPC5, ARPC4, ARPC3, ARPC1B, ARPC2, ABI2, WASF2, APC2, PAK4, MYL9, VAV3, BAIAP2, ARPC1A, MYL12A, GNA13, NCKAP1, IQGAP2, RRAS2, ITGA11, MRAS, CYFIP1, ARHGEF12, PIP5K1C, PIK3R5, TIAM2, FGF20, FGF21, CYFIP2, FGF22, GIT1, MYLPF, ARHGEF4, SSH1, SSH3, ENAH, BRK1, GNG12, PDGFC, PAK6, PAK5, MYL7, MYH14, PIP4K2C, PDGFD, DIAPH3, ARPC5L, MYLK2, SSH2, SCIN, FGD3, MYLK3, MYL10, MYL12B, IQGAP3, PIKFYVE, PFN3, PFN4, LOC646821, LOC653888, LOC100418883

Predefined gene sets in Hepamine

Data source	Pathways	Symbols
KEGG	Regulation Of Autophagy	IFNA1, IFNA2, IFNA4, IFNA5, IFNA6, IFNA7, IFNA8, IFNA10, IFNA13, IFNA14, IFNA16, IFNA17, IFNA21, IFNG, INS, PIK3C3, PRKAA1, PRKAA2, ULK1, BECN1, ATG12, ATG5, ULK2, ATG7, GABARAP, GABARAPL2, ATG4B, GABARAPL1, ULK3, PIK3R4, ATG3, ATG4C, ATG4D, ATG4A, BECN2
KEGG	Renal Cell Carcinoma	AKT1, AKT2, ARAF, ARNT, BRAF, CDC42, CREBBP, CRK, CRKL, EP300, EPAS1, ETS1, FH, VEGFD, GAB1, GRB2, RAPGEF1, HGF, HIF1A, HRAS, JUN, KRAS, MET, NRAS, PAK1, PAK2, PAK3, PDGFB, PGF, PIK3CA, PIK3CB, PIK3CD, PIK3CG, PIK3R1, PIK3R2, MAPK1, MAPK3, MAP2K1, MAP2K2, PTPN11, RAC1, RAF1, RAP1A, RAP1B, SLC2A1, SOS1, SOS2, ELOC, ELOB, TGFA, TGFB1, TGFB2, TGFB3, VEGFA, VEGFB, VEGFC, VHL, CUL2, PIK3R3, ARNT2, RBX1, AKT3, PAK4, PIK3R5, EGLN1, PAK6, PAK5, EGLN2, EGLN3, FLCN
KEGG	Renin Angiotensin System	AGT, AGTR1, AGTR2, ANPEP, CMA1, CPA3, CTSG, ACE, ENPEP, LNPEP, MAS1, MME, CTSA, REN, THOP1, NLN, ACE2
KEGG	Retinol Metabolism	ADH1A, ADH1B, ADH1C, ADH4, ADH5, ADH6, ADH7, ALDH1A1, CYP1A1, CYP1A2, CYP2A6, CYP2A7, CYP3A7, CYP2A13, CYP2B6, CYP2C19, CYP2C8, CYP2C9, CYP2C18, CYP3A4, CYP3A5, CYP4A11, CYP26A1, RDH5, RPE65, UGT2B4, UGT2B7, UGT2B10, UGT2B15, UGT2B17, PNPLA4, RDH16, DGAT1, ALDH1A2, LRAT, DHRS3, DHRS9, UGT2B11, DHRS4, UGT2A1, RDH8, RDH11, BCO1, UGT2B28, UGT1A10, UGT1A8, UGT1A7, UGT1A6, UGT1A5, UGT1A9, UGT1A4, UGT1A1, UGT1A3, RETSAT, CYP26B1, CYP3A43, UGT2A3, DGAT2, RDH12, RDH10, AWAT2, CYP4A22, DHRS4L2, CYP26C1
KEGG	Riboflavin Metabolism	ACP1, ACP2, ACP5, ACPP, ENPP1, ENPP3, TYR, MTMR1, MTMR2, MTMR6, MTMR7, PHPT1, ACP6, RFK, FLAD1, ACP4

Predefined gene sets in Hepamine

Data source	Pathways	Symbols
KEGG	Ribosome	FAU, RPSA, RPL10A, RPL3, RPL3L, RPL4, RPL5, RPL6, RPL7, RPL7A, RPL8, RPL9, RPL10, RPL11, RPL12, RPL13, RPL15, RPL17, RPL18, RPL18A, RPL19, RPL21, RPL22, RPL23A, RPL24, RPL26, RPL27, RPL30, RPL27A, RPL28, RPL29, RPL31, RPL32, RPL34, RPL35A, RPL36AL, RPL37, RPL37A, RPL38, RPL39, RPL41, RPL36A, RPLP0, RPLP1, RPLP2, RPS2, RPS3, RPS3A, RPS4X, RPS4Y1, RPS5, RPS6, RPS7, RPS8, RPS9, RPS10, RPS11, RPS12, RPS13, RPS15, RPS15A, RPS16, RPS17, RPS18, RPS19, RPS20, RPS21, RPS23, RPS24, RPS25, RPS26, RPS27, RPS27A, RPS28, RPS29, UBA52, RPL14, RPL23, RPL35, RPL13A, RPL36, MRPL13, RPS27L, RPL26L1, RSL24D1, RPL10L, RPL22L1, RSL24D1P11
KEGG	Rig I Like Receptor Signaling Pathway	CASP8, CASP10, CHUK, MAPK14, CYLD, DDX3X, IFNA1, IFNA2, IFNA4, IFNA5, IFNA6, IFNA7, IFNA8, IFNA10, IFNA13, IFNA14, IFNA16, IFNA17, IFNA21, IFNB1, IFNW1, IKBKB, CXCL8, IL12A, IL12B, CXCL10, IRF3, IRF7, MAP3K1, NFKB1, NFKBIA, NFKBIB, PIN1, MAPK8, MAPK11, MAPK9, MAPK10, MAPK13, RELA, MAPK12, MAP3K7, TNF, TRAF2, TRAF3, TRAF6, TRIM25, IKBKG, DDX3Y, TRADD, RIPK1, FADD, ATG12, ATG5, ISG15, IKBKE, TBKBP1, TANK, DDX58, TKFC, TBK1, RNF125, OTUD5, IFNK, MAVS, IFIH1, AZI2, DHX58, NLRX1, SIKE1, IFNE, TMEM173
KEGG	Rna Degradation	DDX6, ENO1, ENO2, ENO3, HSPA9, HSPD1, CNOT2, CNOT3, CNOT4, PARN, EXOSC9, EXOSC10, SKIV2L, CNOT9, CNOT8, TTC37, MPHOSPH6, C1D, PAPOLA, TENT4A, LSM6, EXOSC8, XRN2, DIS3, EXOSC7, CNOT1, EXOSC2, MTREX, EDC4, LSM5, LSM4, CNOT10, LSM1, LSM3, DCPS, CNOT7, EXOSC3, EXOSC1, LSM7, LSM8, XRN1, EXOSC4, DCP1A, PAPOLB, EXOSC5, CNOT6, LSM2, PAPOLG, EDC3, WDR61, ZCCHC7, PNPT1, EXOSC6, DCP2, DCP1B, PATL1, CNOT6L, C1DP3, C1DP2

Predefined gene sets in Hepamine

Data source	Pathways	Symbols
KEGG	Rna Polymerase	POLR3D, POLR2A, POLR2B, POLR2C, POLR2D, POLR2E, POLR2F, POLR2G, POLR2H, POLR2I, POLR2J, POLR2K, POLR2L, POLR1C, POLR3F, POLR3G, POLR3C, POLR3A, POLR1A, ZNRD1, POLR1D, POLR3K, POLR3B, POLR1E, POLR1B, POLR3GL, POLR3H, POLR2J2, POLR2J3
KEGG	Selenoamino Acid Metabolism	AHCY, CBS, CTH, GGT1, GGT7, GGT5, MARS, MAT1A, MAT2A, PAPSS2, PAPSS1, LCMT2, AHCYL1, SEPHS2, SEPHS1, AHCYL2, MAT2B, HEMK1, LCMT1, SCLY, METTL2B, TRMT11, MARS2, BUD23, GGT6, METTL6
KEGG	Small Cell Lung Cancer	AKT1, AKT2, APAF1, BIRC2, BIRC3, XIAP, CCND1, BCL2, BCL2L1, CASP9, CCNE1, CDK2, CDK4, CDK6, CDKN1B, CDKN2B, CHUK, CKS1B, COL4A1, COL4A2, COL4A4, COL4A6, E2F1, E2F2, E2F3, FHIT, FN1, IKBKB, ITGA6, ITGA2, ITGA2B, ITGA3, ITGAV, ITGB1, LAMA2, LAMA3, LAMA4, LAMA5, LAMB1, LAMB2, LAMB3, LAMC1, LAMC2, MAX, MYC, NFKB1, NFKBIA, NOS2, PIK3CA, PIK3CB, PIK3CD, PIK3CG, PIK3R1, PIK3R2, PTEN, PTGS2, PTK2, RARB, RB1, RELA, RXRA, RXRB, RXRG, SKP2, TP53, TRAF1, TRAF2, TRAF3, TRAF5, TRAF6, PIK3R3, IKBKG, PIAS1, PIAS2, CCNE2, TRAF4, AKT3, LAMC3, PIAS3, LAMB4, PIK3R5, PIAS4, CYCS, LAMA1
KEGG	Snare Interactions In Vesicular Transport	BNIP1, STX2, SNAP25, STX1A, STX3, STX4, STX5, VAMP1, VAMP2, VAMP7, STX7, VAMP8, VAMP4, STX16, STX11, STX10, SNAP23, VAMP3, SNAP29, STX8, GOSR1, SEC22B, GOSR2, STX6, BET1, VTI1B, YKT6, VAMP5, STX12, BET1L, STX18, STX17, USE1, STX1B, SNAP47, VTI1A, TSNARE1, STX19
KEGG	Sphingolipid Metabolism	ARSA, ASAH1, KDSR, GALC, GBA, GLA, GLB1, NEU1, NEU2, SMPD1, SMPD2, UGCG, UGT8, DEGS1, PLPP1, PLPP2, PLPP3, SPHK1, SGPL1, B4GALT6, GAL3ST1, SPTLC2, SPTLC1, NEU3, ACER3, SMPD3, SMPD4, ASAH2, SPHK2, CERK, SGPP1, DEGS2, ACER1, NEU4, SGPP2, SGMS2, SGMS1, ENPP7, ACER2, ASAH2C

Predefined gene sets in Hepamine

**Data source**	**Pathways**	**Symbols**
KEGG	Spliceosome	CDC5L, DDX5, DHX8, DHX15, HNRNPA1, HNRNPC, HNRNPK, HNRNPU, HSPA1A, HSPA1B, HSPA1L, HSPA2, HSPA6, HSPA8, MAGOH, HNRNPM, NCBP1, SNU13, PCBP1, PLRG1, SRSF1, SRSF2, SRSF3, SRSF4, SRSF5, SRSF6, SRSF7, TRA2B, SNRNP70, SNRPA, SNRPA1, SNRPB, SNRPB2, SNRPC, SNRPD1, SNRPD2, SNRPD3, SNRPE, SNRPF, SNRPG, U2AF1, DDX39B, SF3A2, DHX16, PRPF18, SRSF9, BUD31, SART1, PRPF4, PRPF3, EFTUD2, SNRNP40, DDX23, AQR, EIF4A3, DHX38, DDX46, RBM8A, THOC1, PQBP1, ALYREF, SF3B4, SMNDC1, BCAS2, SF3A1, PPIE, PPIH, CHERP, SLU7, PRPF8, USP39, SRSF10, TXNL4A, TCERG1, SRSF8, SF3A3, SF3B2, SNRNP27, LSM6, DDX42, U2AF2, PUF60, NCBP2, SNW1, ACIN1, SNRNP200, U2SURP, SF3B3, SF3B1, LSM5, PRPF6, PRPF40B, LSM4, SYF2, PRPF31, LSM3, RBMX, PRPF19, TRA2A, CRNKL1, CDC40, CWC15, SF3B6, PPIL1, LSM7, LSM8, WBP11, MAGOHB, PRPF38B, PRPF40A, RBM22, CTNNBL1, XAB2, THOC2, ISY1, LSM2, RBM25, SF3B5, THOC3, PHF5A, PRPF38A, RBM17, HNRNPA1P60, HNRNPA1L2, CCDC12, ZMAT2, HNRNPA3, LOC653884
KEGG	Starch And Sucrose Metabolism	AGL, AMY1A, AMY1B, AMY1C, AMY2A, AMY2B, G6PC, GAA, GANC, GBE1, GCK, GPI, GUSB, GYS1, GYS2, HK1, HK2, HK3, ENPP1, ENPP3, PGM1, PYGB, PYGL, PYGM, SI, UGDH, UGP2, UGT2B4, UGT2B7, UGT2B10, UGT2B15, UGT2B17, MGAM, UGT2B11, UGT2A1, TREH, UGT2B28, UGT1A10, UGT1A8, UGT1A7, UGT1A6, UGT1A5, UGT1A9, UGT1A4, UGT1A1, UGT1A3, PGM2, GBA3, G6PC2, UGT2A3, UXS1, PGM2L1
KEGG	Steroid Biosynthesis	CEL, CYP27B1, CYP51A1, DHCR7, DHCR24, FDFT1, LIPA, LSS, MSMO1, SC5D, SOAT1, SQLE, TM7SF2, SOAT2, EBP, NSDHL, HSD17B7

Predefined gene sets in Hepamine

**Data source**	**Pathways**	**Symbols**
KEGG	Steroid Hormone Biosynthesis	STS, AKR1C4, COMT, CYP1A1, CYP1B1, CYP3A7, CYP3A4, CYP3A5, CYP7A1, CYP11A1, CYP11B1, CYP11B2, CYP17A1, CYP19A1, CYP21A2, AKR1C1, AKR1C2, HSD3B1, HSD3B2, HSD11B1, HSD11B2, HSD17B1, HSD17B3, HSD17B2, SRD5A1, SRD5A2, AKR1D1, SULT1E1, SULT2B1, UGT2B4, UGT2B7, UGT2B10, UGT2B15, UGT2B17, HSD17B8, HSD17B6, AKR1C3, CYP7B1, UGT2B11, UGT2A1, HSD17B12, HSD17B7, UGT2B28, UGT1A10, UGT1A8, UGT1A7, UGT1A6, UGT1A5, UGT1A9, UGT1A4, UGT1A1, UGT1A3, CYP3A43, SRD5A3, UGT2A3
KEGG	Sulfur Metabolism	SULT1E1, SULT1A2, SULT1A1, SULT1A3, SULT2B1, SUOX, PAPSS2, PAPSS1, BPNT1, CHST11, CHST12, CHST13, SULT1A4

Predefined gene sets in Hepamine

**Data source**	**Pathways**	**Symbols**
KEGG	Systemic Lupus Erythematosus	ACTN4, ACTN1, ACTN2, ACTN3, C1QA, C1QB, C1QC, C1R, C1S, C2, C3, C4A, C4B, C5, C6, C7, C8A, C8B, C8G, C9, CD28, CD80, CD86, CD40, CD40LG, CTSG, ELANE, FCGR1A, FCGR2A, FCGR2B, FCGR3A, FCGR3B, GRIN2A, GRIN2B, HIST1H2AE, HIST1H2AD, H2AFX, H2AFZ, HIST1H2BD, HIST1H2BB, H3F3A, H3F3B, HLA-DMA, HLA-DMB, HLA-DOA, HLA-DOB, HLA-DPA1, HLA-DPB1, HLA-DQA1, HLA-DQA2, HLA-DQB1, HLA-DRA, HLA-DRB1, HLA-DRB3, HLA-DRB4, HLA-DRB5, IFNG, IL10, SNRPB, SNRPD1, SNRPD3, TRIM21, TROVE2, SSB, TNF, HIST3H3, HIST1H4I, HIST1H2AI, HIST1H2AK, HIST1H2AJ, HIST1H2AL, HIST1H2AC, HIST1H2AB, HIST1H2AM, HIST2H2AA3, HIST2H2AC, HIST1H2BG, HIST1H2BL, HIST1H2BN, HIST1H2BM, HIST1H2BF, HIST1H2BE, HIST1H2BH, HIST1H2BI, HIST1H2BC, HIST1H2BO, HIST2H2BE, HIST1H3A, HIST1H3D, HIST1H3C, HIST1H3E, HIST1H3I, HIST1H3G, HIST1H3J, HIST1H3H, HIST1H3B, HIST1H4A, HIST1H4D, HIST1H4F, HIST1H4K, HIST1H4J, HIST1H4C, HIST1H4H, HIST1H4B, HIST1H4E, HIST1H4L, HIST1H4G, HIST2H4A, HIST1H3F, HIST1H2AG, HIST1H2BJ, FCGR2C, H2AFY, H2AFY2, H2AFJ, H2AFB3, HIST1H2AH, HIST1H2BK, HIST3H2A, H2AFV, HIST4H4, HIST2H3C, HIST3H2BB, H2BFWT, HIST1H2AA, HIST1H2BA, H2BFM, HIST2H2AB, HIST2H3A, H3.X, H3.Y, H3F3C, HIST2H2BF, H2AFB2, H2AFB1, HIST2H4B, LOC644950, HIST2H3D, HIST2H2AA4, HIST1H2APS6
KEGG	Taste Transduction	ASIC2, ADCY6, ADCY8, CACNA1A, CACNA1B, GNAS, GNB1, GNB3, GNG3, GRM4, ITPR3, KCNB1, PDE1A, PLCB2, PRKACA, PRKACB, PRKACG, PRKX, TAS2R38, SCNN1A, SCNN1B, SCNN1G, TRPM5, TAS2R3, TAS2R4, TAS2R16, TAS2R1, TAS2R9, TAS2R8, TAS2R7, TAS2R13, TAS2R10, TAS2R14, GNG13, TAS2R5, TAS1R2, TAS1R1, TAS1R3, ADCY4, TAS2R39, TAS2R40, TAS2R41, TAS2R43, TAS2R31, TAS2R45, TAS2R46, TAS2R19, TAS2R20, TAS2R50, TAS2R60, GNAT3, TAS2R42

Predefined gene sets in Hepamine

Data source	Pathways	Symbols
KEGG	Taurine And Hypotaurine Metabolism	BAAT, CDO1, GAD1, GAD2, GGT1, GGT7, GGT5, CSAD, ADO, GGT6
KEGG	T Cell Receptor Signaling Pathway	AKT1, AKT2, RHOA, CBL, CBLB, CD3D, CD3E, CD3G, CD247, CD4, CD8A, CD8B, CD28, CD40LG, CDC42, CDK4, CHUK, MAP3K8, MAPK14, CSF2, CTLA4, DLG1, FOS, FYN, GRB2, GSK3B, HRAS, IFNG, IKBKB, IL2, IL4, IL5, IL10, ITK, JUN, KRAS, LCK, LCP2, NCK1, NFATC1, NFATC2, NFATC3, NFATC4, NFKB1, NFKBIA, NFKBIB, NFKBIE, NRAS, PAK1, PAK2, PAK3, PDCD1, PDPK1, PIK3CA, PIK3CB, PIK3CD, PIK3CG, PIK3R1, PIK3R2, PLCG1, PPP3CA, PPP3CB, PPP3CC, PPP3R1, PPP3R2, PRKCQ, MAPK1, MAPK3, MAPK11, MAPK9, MAPK13, MAP2K1, MAP2K2, MAP2K7, PTPN6, PTPRC, RAF1, RELA, MAPK12, SOS1, SOS2, MAP3K7, TEC, TNF, VAV1, VAV2, ZAP70, NCK2, PIK3R3, IKBKG, BCL10, MAP3K14, GRAP2, AKT3, RASGRP1, PAK4, VAV3, NFAT5, MALT1, CHP1, PIK3R5, CBLC, LAT, ICOS, PAK6, PAK5, CHP2, CARD11
KEGG	Terpenoid Backbone Biosynthesis	ACAT1, ACAT2, FDPS, HMGCR, HMGCS1, HMGCS2, IDI1, MVD, MVK, GGPS1, PMVK, PDSS1, PDSS2, DHDDS, IDI2
KEGG	Tgf Beta Signaling Pathway	ACVR1, ACVR2A, ACVR2B, ACVRL1, AMH, AMHR2, RHOA, BMP2, BMP4, BMP5, BMP6, BMP7, BMP8B, BMPR1A, BMPR1B, BMPR2, CDKN2B, COMP, CREBBP, DCN, E2F4, E2F5, EP300, ID1, ID2, ID3, ID4, IFNG, INHBA, INHBB, INHBC, LTBP1, SMAD1, SMAD2, SMAD3, SMAD4, SMAD5, SMAD6, SMAD7, SMAD9, MYC, NODAL, PITX2, PPP2CA, PPP2CB, PPP2R1A, PPP2R1B, MAPK1, MAPK3, RBL1, RBL2, ROCK1, RPS6KB1, RPS6KB2, SKP1, SP1, TFDP1, TGFB1, TGFB2, TGFB3, LEFTY2, TGFBR1, TGFBR2, THBS1, THBS2, THBS3, THBS4, TNF, GDF5, CUL1, CHRD, NOG, ZFYVE9, ROCK2, ZFYVE16, RBX1, FST, LEFTY1, SMURF1, SMURF2, INHBE, ACVR1C, GDF7, BMP8A, GDF6, SKP1P2

Predefined gene sets in Hepamine

Data source	Pathways	Symbols
KEGG	Thyroid Cancer	CCND1, BRAF, CDH1, CTNNB1, HRAS, KRAS, MYC, NRAS, NTRK1, PPARG, MAPK1, MAPK3, MAP2K1, MAP2K2, RET, RXRA, RXRB, RXRG, TCF7, TCF7L2, TP53, TPM3, TPR, PAX8, CCDC6, NCOA4, TFG, LEF1, TCF7L1
KEGG	Tight Junction	ACTB, ACTG1, ACTN4, ACTN1, ACTN2, ACTN3, AKT1, AKT2, RHOA, CDC42, CDK4, CLDN4, CLDN3, CLDN7, CSNK2A1, CSNK2A2, CSNK2B, CTNNA1, CTNNA2, CTNNB1, CTTN, EPB41, EPB41L1, EPB41L2, GNAI1, GNAI2, GNAI3, HCLS1, HRAS, KRAS, LLGL2, LLGL1, AFDN, MYH1, MYH2, MYH3, MYH4, MYH6, MYH7, MYH8, MYH9, MYH10, MYH11, MYL2, MYL5, NRAS, CLDN11, PPP2CA, PPP2CB, PPP2R1A, PPP2R1B, PPP2R2A, PPP2R2B, PPP2R2C, PRKCA, PRKCB, PRKCD, PRKCE, PRKCG, PRKCH, PRKCI, PRKCQ, PRKCZ, PTEN, RAB3B, RAB13, RRAS, SPTAN1, SRC, TJP1, CLDN5, YES1, SYMPK, YBX3, CASK, MYH13, MPDZ, CLDN10, CLDN8, CLDN6, CLDN2, CLDN1, CLDN9, VAPA, MAGI1, TJP2, MAGI2, AKT3, PATJ, MYL9, MYL12A, CLDN16, EXOC3, RRAS2, MRAS, MYH15, EPB41L3, CLDN14, CLDN15, CLDN17, TJP3, CTNNA3, MYLPF, CLDN20, F11R, PARD6A, CLDN18, MAP3K20, CLDN22, PPP2R2D, ASH1L, PARD3, CGN, MYH7B, JAM2, MYL7, EXOC4, MPP5, MYH14, JAM3, PARD6G, PARD6B, CRB3, MYL10, TJAP1, MYL12B, CLDN23, CLDN19, IGSF5, AMOTL1, MAGI3, LOC646821, LOC100418883, OCLN

Predefined gene sets in Hepamine

Data source	Pathways	Symbols
KEGG	Toll Like Receptor Signaling Pathway	AKT1, AKT2, CASP8, CD14, CD80, CD86, CD40, CHUK, MAP3K8, MAPK14, CTSK, FOS, IFNA1, IFNA2, IFNA4, IFNA5, IFNA6, IFNA7, IFNA8, IFNA10, IFNA13, IFNA14, IFNA16, IFNA17, IFNA21, IFNAR1, IFNAR2, IFNB1, IKBKB, IL1B, IL6, CXCL8, IL12A, IL12B, CXCL10, IRAK1, IRF3, IRF5, IRF7, JUN, LBP, CXCL9, MYD88, NFKB1, NFKBIA, PIK3CA, PIK3CB, PIK3CD, PIK3CG, PIK3R1, PIK3R2, MAPK1, MAPK3, MAPK8, MAPK11, MAPK9, MAPK10, MAPK13, MAP2K1, MAP2K2, MAP2K3, MAP2K6, MAP2K7, RAC1, RELA, MAPK12, CCL3, CCL4, CCL5, CXCL11, MAP2K4, SPP1, STAT1, MAP3K7, TLR1, TLR2, TLR3, TLR4, TLR5, TNF, TRAF3, TRAF6, PIK3R3, IKBKG, RIPK1, FADD, IKBKE, AKT3, TLR6, TAB1, TAB2, PIK3R5, LY96, TBK1, IRAK4, TLR7, TLR8, TLR9, TOLLIP, TIRAP, TICAM1, TICAM2
KEGG	Tryptophan Metabolism	AANAT, AOC1, ACAT1, ACAT2, ALDH2, ALDH1B1, ALDH9A1, ALDH3A2, AOX1, ASMT, ALDH7A1, CAT, CYP1A1, CYP1A2, CYP1B1, DDC, ECHS1, EHHADH, GCDH, HADHA, HADH, IDO1, MAOA, MAOB, OGDH, TDO2, TPH1, WARS, KMO, KYNU, WARS2, INMT, HAAO, AADAT, OGDHL, TPH2, AFMID, ACMSD, IDO2, IL4I1
KEGG	Type I Diabetes Mellitus	FAS, FASLG, CD28, CD80, CD86, CPE, GAD1, GAD2, GZMB, HLA-A, HLA-B, HLA-C, HLA-DMA, HLA-DMB, HLA-DOA, HLA-DOB, HLA-DPA1, HLA-DPB1, HLA-DQA1, HLA-DQA2, HLA-DQB1, HLA-DRA, HLA-DRB1, HLA-DRB3, HLA-DRB4, HLA-DRB5, HLA-E, HLA-F, HLA-G, HSPD1, ICA1, IFNG, IL1A, IL1B, IL2, IL12A, IL12B, INS, LTA, PRF1, PTPRN, PTPRN2, TNF, LOC652614
KEGG	Type Ii Diabetes Mellitus	CACNA1A, CACNA1B, CACNA1C, CACNA1D, CACNA1E, MTOR, GCK, HK1, HK2, HK3, IKBKB, INS, INSR, PDX1, IRS1, KCNJ11, PIK3CA, PIK3CB, PIK3CD, PIK3CG, PIK3R1, PIK3R2, PKLR, PKM, PRKCD, PRKCE, PRKCZ, MAPK1, MAPK3, MAPK8, MAPK9, MAPK10, SLC2A2, SLC2A4, ABCC8, TNF, IRS4, PIK3R3, SOCS1, IRS2, SOCS2, CACNA1G, SOCS3, ADIPOQ, PIK3R5, SOCS4, MAFA

Predefined gene sets in Hepamine

**Data source**	**Pathways**	**Symbols**
KEGG	Tyrosine Metabolism	ADH1A, ADH1B, ADH1C, ADH4, ADH5, ADH6, ADH7, ALDH3A1, ALDH1A3, ALDH3B1, ALDH3B2, AOC2, AOX1, COMT, DBH, DCT, DDC, FAH, GOT1, GOT2, GSTZ1, HGD, HPD, MAOA, MAOB, MIF, PNMT, TAT, TH, TPO, TYR, TYRP1, AOC3, LCMT2, NAA80, HEMK1, LCMT1, METTL2B, TRMT11, BUD23, METTL6, IL4I1
KEGG	Ubiquitin Mediated Proteolysis	AIRE, BIRC2, BIRC3, XIAP, BRCA1, CBL, CBLB, CDC20, CDC27, CDC34, ERCC8, DDB1, DDB2, UBE2K, MDM2, MAP3K1, MID1, TRIM37, NEDD4, PRKN, PML, SIAH1, SKP1, SKP2, ELOC, ELOB, TRAF6, UBA1, UBA7, UBE2A, UBE2B, UBE2D1, UBE2D2, UBE2D3, UBE2E1, UBE2E2, UBE2G1, UBE2G2, UBE2H, UBE2I, UBE2L3, UBE2N, UBE3A, VHL, CUL5, CUL4B, CUL4A, CUL3, CUL2, CUL1, PIAS1, SOCS1, CDC23, CDC16, HERC3, HERC2, HERC1, BTRC, SOCS3, UBA3, UBE2M, PIAS2, UBE2L6, TRIP12, UBE4A, RNF7, UBE3C, KEAP1, CUL7, RBX1, UBA2, SAE1, HUWE1, STUB1, UBE4B, ANAPC10, PIAS3, UBE2E3, WWP1, WWP2, UBE2C, UBOX5, TRIM32, RHOBTB2, FBXW11, MGRN1, NEDD4L, CBLC, PPIL2, ANAPC13, RCHY1, HERC4, FBXO2, FBXW8, FBXO4, UBE2S, PRPF19, ANAPC2, ANAPC4, FZR1, UBR5, ANAPC5, ANAPC7, UBE2J1, ANAPC11, PIAS4, UBE2D4, UBE2R2, DET1, FANCL, UBA6, UBE2W, FBXW7, UBE2Q1, KLHL9, SMURF1, BIRC6, UBE2O, COP1, ANAPC1, SMURF2, UBE2Z, ITCH, SYVN1, UBE3B, KLHL13, UBE2Q2, UBE2J2, UBE2QL1, UBE2F, UBE2U, CDC26, NHLRC1, UBE2NL, LOC650621, LOC652346, LOC652671, SKP1P2
KEGG	Valine Leucine And Isoleucine Biosynthesis	BCAT1, BCAT2, IARS, PDHA1, PDHA2, PDHB, VARS, LARS2, LARS, IARS2, VARS2

Predefined gene sets in Hepamine

Data source	Pathways	Symbols
KEGG	Valine Leucine And Isoleucine Degradation	ABAT, ACAA1, ACADM, ACADS, ACADSB, ACAT1, ACAT2, ALDH2, ALDH1B1, ALDH9A1, ALDH3A2, AOX1, ALDH7A1, AUH, BCAT1, BCAT2, BCKDHA, BCKDHB, DBT, DLD, ECHS1, EHHADH, HSD17B10, HADHA, HADHB, HADH, HMGCL, HMGCS1, HMGCS2, IVD, ALDH6A1, MMUT, OXCT1, PCCA, PCCB, ACAA2, HIBADH, HIBCH, ACAD8, MCCC1, OXCT2, MCCC2, MCEE, IL4I1
KEGG	Vascular Smooth Muscle Contraction	ACTA2, ACTG2, ADCY1, ADCY2, ADCY3, ADCY5, ADCY6, ADCY7, ADCY8, ADCY9, ADORA2A, ADORA2B, ADRA1D, ADRA1B, ADRA1A, AGTR1, ARAF, RHOA, AVPR1A, AVPR1B, BRAF, CACNA1C, CACNA1D, CACNA1F, CACNA1S, CALD1, CALM1, CALM2, CALM3, CALML3, CYP4A11, EDNRA, GNA11, GNA12, GNAQ, GNAS, GUCY1A2, GUCY1A1, GUCY1B1, ITPR1, ITPR2, ITPR3, KCNMA1, KCNMB1, MYH11, MYL6, MYLK, PPP1R12A, PPP1R12B, NPR1, NPR2, PLA2G1B, PLA2G2A, PLA2G4A, PLA2G5, PLCB2, PLCB3, PLCB4, PPP1CA, PPP1CB, PPP1CC, PRKACA, PRKACB, PRKACG, PRKCA, PRKCB, PRKCD, PRKCE, PRKCG, PRKCH, PRKCQ, PRKG1, MAPK1, MAPK3, MAP2K1, MAP2K2, PRKX, PTGIR, RAF1, ROCK1, PLA2G6, PLA2G10, JMJD7-PLA2G4B, ARHGEF1, ROCK2, ARHGEF11, CALCRL, KCNMB2, RAMP2, RAMP1, RAMP3, MRVI1, MYL9, GNA13, PLCB1, ARHGEF12, PLA2G2D, KCNMB3, KCNMB4, PLA2G2E, PLA2G3, CALML5, PLA2G2F, PLA2G12A, PLA2G12B, MYLK2, MYLK3, PPP1R14A, PLA2G4E, MYL6B, CALML6, ADCY4, CYP4A22, PLA2G2C, PLA2G4B

Predefined gene sets in Hepamine

Data source	Pathways	Symbols
KEGG	Vasopressin Regulated Water Reabsorption	ADCY3, ADCY6, ADCY9, AQP2, AQP3, AQP4, ARHGDIA, ARHGDIB, AVP, AVPR2, CREB1, DCTN1, DYNC1H1, DYNC1I1, DYNC1I2, DYNC1LI2, GNAS, NSF, PRKACA, PRKACB, PRKACG, PRKX, RAB5A, RAB5B, RAB5C, STX4, VAMP2, DYNLL1, RAB11A, RAB11B, CREB5, CREB3, DCTN2, DCTN6, DYNC1LI1, DCTN4, DYNC2LI1, CREB3L2, DYNC2H1, DCTN5, CREB3L3, CREB3L1, DYNLL2, CREB3L4
KEGG	Vegf Signaling Pathway	AKT1, AKT2, BAD, CASP9, CDC42, MAPK14, HRAS, HSPB1, KDR, KRAS, NFATC1, NFATC2, NFATC3, NFATC4, NOS3, NRAS, PIK3CA, PIK3CB, PIK3CD, PIK3CG, PIK3R1, PIK3R2, PLA2G1B, PLA2G2A, PLA2G4A, PLA2G5, PLCG1, PLCG2, PPP3CA, PPP3CB, PPP3CC, PPP3R1, PPP3R2, PRKCA, PRKCB, PRKCG, MAPK1, MAPK3, MAPK11, MAPK13, MAP2K1, MAP2K2, PTGS2, PTK2, PXN, RAC1, RAC2, RAC3, RAF1, MAPK12, SRC, VEGFA, MAPKAPK3, PLA2G6, PLA2G10, PIK3R3, JMJD7-PLA2G4B, SPHK1, SH2D2A, MAPKAPK2, AKT3, NFAT5, CHP1, PIK3R5, SHC2, PLA2G2D, PLA2G2E, PLA2G3, SPHK2, CHP2, PLA2G2F, PLA2G12A, PLA2G12B, PLA2G4E, PLA2G2C, PLA2G4B
KEGG	Vibrio Cholerae Infection	ACTB, ACTG1, ADCY3, ADCY9, ARF1, ATP6V1A, ATP6V1B1, ATP6V1B2, ATP6V0C, ATP6V1C1, ATP6V1E1, ATP6V0B, ATP6V1G2, ATP6V0A1, ATP6AP1, CFTR, GNAS, KCNQ1, MUC2, PLCG1, PLCG2, PRKACA, PRKACB, PRKACG, PRKCA, PRKCB, PRKCG, PRKX, SLC12A2, TJP1, ATP6V0E1, ATP6V0D1, ATP6V1F, TJP2, ATP6V1G1, PDIA4, TCIRG1, KDELR1, SEC61B, KDELR2, KDELR3, SEC61G, ATP6V0A2, SEC61A1, ERO1A, ATP6V0A4, ATP6V1D, ATP6V1H, SEC61A2, ATP6V1E2, ATP6V1G3, ATP6V0E2, ATP6V0D2, ATP6V1C2, LOC646821, LOC100418883

Predefined gene sets in Hepamine

Data source	Pathways	Symbols
KEGG	Viral Myocarditis	ABL1, ABL2, ACTB, ACTG1, CCND1, BID, CASP3, CASP8, CASP9, CAV1, CD28, CD80, CD86, CD40, CD40LG, CXADR, CD55, DAG1, DMD, EIF4G1, EIF4G2, FYN, HLA-A, HLA-B, HLA-C, HLA-DMA, HLA-DMB, HLA-DOA, HLA-DOB, HLA-DPA1, HLA-DPB1, HLA-DQA1, HLA-DQA2, HLA-DQB1, HLA-DRA, HLA-DRB1, HLA-DRB3, HLA-DRB4, HLA-DRB5, HLA-E, HLA-F, HLA-G, ICAM1, ITGAL, ITGB2, LAMA2, MYH1, MYH2, MYH3, MYH4, MYH6, MYH7, MYH8, MYH9, MYH10, MYH11, PRF1, RAC1, RAC2, RAC3, SGCA, SGCB, SGCD, SGCG, EIF4G3, MYH13, MYH15, CYCS, MYH7B, MYH14, LOC646821, LOC652614, LOC100418883
KEGG	Wnt Signaling Pathway	APC, RHOA, CCND1, CAMK2A, CAMK2B, CAMK2D, CAMK2G, CCND2, CCND3, CREBBP, CSNK1A1, CSNK1E, CSNK2A1, CSNK2A2, CSNK2B, CTBP1, CTBP2, CTNNB1, DVL1, DVL2, DVL3, EP300, FZD2, GSK3B, JUN, LRP6, LRP5, SMAD2, SMAD3, SMAD4, MMP7, MYC, NFATC1, NFATC2, NFATC3, NFATC4, PLCB2, PLCB3, PLCB4, PPARD, PPP2CA, PPP2CB, PPP2R1A, PPP2R1B, PPP2R5A, PPP2R5B, PPP2R5C, PPP2R5D, PPP2R5E, PPP3CA, PPP3CB, PPP3CC, PPP3R1, PPP3R2, PRKACA, PRKACB, PRKACG, PRKCA, PRKCB, PRKCG, MAPK8, MAPK9, MAPK10, PRKX, PSEN1, RAC1, RAC2, RAC3, ROCK1, SFRP1, SFRP2, SFRP4, SFRP5, SIAH1, SKP1, MAP3K7, TBL1X, TCF7, TCF7L2, TP53, WNT1, WNT2, WNT3, WNT5A, WNT6, WNT7A, WNT7B, WNT8A, WNT8B, WNT10B, WNT11, WNT2B, WNT9A, WNT9B, FZD5, FZD3, FOSL1, AXIN1, AXIN2, FZD1, FZD4, FZD6, FZD7, FZD8, FZD9, CUL1, RUVBL1, BTRC, CER1, ROCK2, RBX1, FRAT1, APC2, NFAT5, WIF1, FZD10, CHP1, DKK1, DAAM1, PLCB1, FBXW11, FRAT2, DAAM2, CACYBP, DKK4, DKK2, LEF1, WNT16, NLK, WNT4, CTNNBIP1, VANGL2, CHD8, SENP2, CHP2, SOX17, PORCN, TBL1XR1, CXXC4, WNT10A, WNT5B, VANGL1, TCF7L1, NKD1, NKD2, WNT3A, TBL1Y, CSNK1A1L, PRICKLE1, PRICKLE2, SKP1P2

Predefined gene sets in Hepamine

Data source	Pathways	Symbols
WIKIPATHWAYS	4-hydroxytamoxifen, Dexamethasone, and Retinoic Acids Regulation of p27 Expression	AKT1, EIF4E, EIF4EBP1, MTOR, MKNK2, MAP3K5, MAP3K11, PRKAA1, MAPK1, MAPK3, MAP2K1, MAP2K2, MAP2K3, MAP2K6, RAF1, TSC1, MKNK1, PBK
WIKIPATHWAYS	ACE Inhibitor Pathway	AGT, AGTR1, AGTR2, BDKRB1, BDKRB2, CMA1, CTSG, CYP11B2, ACE, KNG1, MAS1, NR3C2, NOS3, REN, TGFB1, ATP6AP2, ACE2
WIKIPATHWAYS	Acetylcholine Synthesis	ACHE, CHAT, CHKA, PCYT1A, PDHA1, PDHA2, PEMT
WIKIPATHWAYS	Acrylamide Biotransformation and Exposure Biomarkers	CYP2E1
WIKIPATHWAYS	Adipogenesis	PLIN2, AGT, AHR, FAS, ASIP, BMP1, BMP2, BMP3, BMP4, KLF5, CDKN1A, CEBPA, CEBPB, CEBPD, CNTFR, KLF6, CREB1, CTNNB1, CYP26A1, GADD45A, DDIT3, CFD, DVL1, E2F1, E2F4, EBF1, EGR2, EPAS1, FOXC2, FOXO1, FRZB, GATA2, GATA3, GATA4, GDF10, GH1, NR3C1, GTF3A, HIF1A, HMGA1, ID3, IGF1, IL6, IL6ST, INS, IRS1, LEP, LIF, LIFR, LIPE, LMNA, LPL, SMAD3, MBNL1, MEF2A, BORCS8-MEF2B, MEF2C, MEF2D, MIF, GADD45B, NDN, OSM, SERPINE1, PCK1, PCK2, PLIN1, PPARA, PPARD, PPARG, PRLR, PTGIS, RARA, RB1, RBL1, RBL2, RORA, RXRA, RXRG, SCD, SFRP4, SLC2A4, SP1, SPOCK1, SREBF1, STAT1, STAT2, STAT3, STAT5A, STAT5B, STAT6, HNF1A, NR2F1, TGFB1, TNF, TWIST1, UCP1, WNT1, WNT10B, NRIP1, FZD1, IRS4, KLF7, NCOA1, SOCS1, IRS2, DLK1, SOCS3, ADIPOQ, NCOR1, NCOR2, LPIN2, NR1H3, NAMPT, ZMPSTE24, NCOA2, AGPAT2, CELF1, PPARGC1A, LPIN1, WWTR1, BSCL2, KLF15, CISD1, CYP26B1, RETN, TRIB3, LPIN3, PNPLA3, WNT5B, MIXL1, MIR6808
WIKIPATHWAYS	Aflatoxin B1 metabolism	CYP1A2, CYP2A13, CYP3A4, EPHX1, GSTM1, AKR7A2, AKR7A3

Predefined gene sets in Hepamine

Data source	Pathways	Symbols
WIKIPATHWAYS	AGE/RAGE pathway	AGER, AKT1, ALPL, RHOA, CASP3, CASP8, CASP9, CDC42, CHUK, ATF2, MAPK14, DDOST, DIAPH1, EGFR, FOXO1, HIF1A, IKBKB, INHBB, INS, INSR, IRS1, JAK2, JUN, LGALS3, SMAD2, SMAD3, FOXO4, MMP2, MMP7, MMP9, MMP13, MMP14, MSN, MSR1, MYD88, NFKB1, NFKBIA, NOS2, NOS3, PLA2G4A, PRKCA, PRKCB, PRKCD, PRKCZ, MAPK1, MAPK3, MAPK8, MAPK9, MAP2K1, RAC1, RAF1, RELA, ROCK1, SHC1, SOD1, SP1, SRC, STAT1, STAT3, STAT5A, STAT5B, EZR, IRAK4, CYCS, TIRAP, NCF1
WIKIPATHWAYS	Alanine and aspartate metabolism	ABAT, AGXT, ASL, ASPA, ASS1, DARS, GAD1, GAD2, GOT1, GOT2, GPT, PC
WIKIPATHWAYS	Allograft Rejection	AGTR1, FAS, FASLG, CXCR5, C1QA, C1QB, C1QC, C2, C3, C4A, C4B, C5, C6, C7, C8A, C8B, C9, CASP3, CASP7, CASP8, CASP9, CD28, CD80, CD86, CD40, CD40LG, COL5A1, CSNK2A2, CTLA4, CD55, GABPA, GDNF, GZMB, HARS, HLA-A, HLA-B, HLA-C, HLA-DMA, HLA-DMB, HLA-DOA, HLA-DOB, HLA-DPA1, HLA-DPB1, HLA-DQA1, HLA-DQA2, HLA-DQB1, HLA-DRA, HLA-DRB1, HLA-DRB5, HLA-E, HLA-F, HLA-G, IFNG, IL1A, IL1B, IL2, IL2RA, IL4, IL5, CXCL8, IL10, IL12A, IL12B, IL13, IL17A, CXCL9, PDGFRA, ABCB1, PRF1, PRKCZ, CCL19, CCL21, CXCL11, CXCL12, STAT1, TGFB1, TNF, VEGFA, VIM, TUBA1B, CXCL13, GNLY, BHMT2, IL22, FOXP3, PECR, IL21, LRRK2, MICA, LOC105369230
WIKIPATHWAYS	Alpha 6 Beta 4 signaling pathway	AKT1, RHOA, MAPK14, EIF4EBP1, MTOR, GAB1, GRB2, HRAS, ITGA6, IRS1, ITGB4, LAMA2, LAMA3, LAMA5, LAMB1, LAMB2, LAMB3, LAMC1, LAMC2, PIK3R1, PIK3R2, PRKCA, PRKCD, MAPK1, MAPK3, PTK2, PTPN11, RAC1, SHC1, SOS1, SRC, IRS2, LAMA1, MIR4260, MIR4758

Predefined gene sets in Hepamine

Data source	Pathways	Symbols
WIKIPATHWAYS	Alzheimers Disease	ADAM10, APAF1, APBB1, APOE, APP, FAS, ATP2A1, ATP2A2, ATP2A3, BAD, BID, CACNA1C, CACNA1D, CACNA1F, CACNA1S, CALM1, CALM2, CALM3, CALML3, CAPN1, CAPN2, CASP3, CASP7, CASP8, CASP9, CDK5, ERN1, GAPDH, GNAQ, GRIN1, GRIN2A, GRIN2B, GRIN2C, GRIN2D, GSK3B, HSD17B10, IDE, IL1B, ITPR1, ITPR2, ITPR3, LPL, LRP1, MAPT, MME, NOS1, PLCB2, PLCB3, PLCB4, PPP3CA, PPP3CB, PPP3CC, PPP3R1, PPP3R2, MAPK1, MAPK3, PSEN1, PSEN2, RYR3, SNCA, ADAM17, TNF, TNFRSF1A, TP53, FADD, CDK5R1, NAE1, EIF2AK3, CHP1, ATF6, PLCB1, NCSTN, BACE1, APH1A, CALML5, CYCS, CHPF2, PSENEN, CHP2, VMP1, ATP5MD, LINC00599, MIR9-3HG, MIR101-1, MIR10A, MIR124-1, MIR124-2, MIR124-3, MIR125A, MIR125B1, MIR127, MIR129-1, MIR129-2, MIR132, MIR134, MIR135A1, MIR135A2, MIR136, MIR138-1, MIR138-2, MIR139, MIR181A2, MIR181B1, MIR181B2, MIR182, MIR184, MIR199B, MIR21, MIR181A1, MIR218-1, MIR218-2, MIR219A2, MIR29A, MIR29B1, MIR30C2, MIR34B, MIR34C, MIR9-1, MIR9-2, MIR9-3, MIR95, MIR323A, MIR326, MIR375, MIR377, MIR381, MIR433, MIR431, MIR329-1, MIR329-2, MIR410, MIR488, MIR495, MIR181D, LINC00461, MIR33B, MIR598, MIR671, MIR769, MIR873, MIR708, MIR874, MIR887, MIR760, MIR1307, MIR1285-2, MIR3200, MIR3176, CASP12
WIKIPATHWAYS	Amino acid conjugation of benzoic acid	GLYAT, ACSS2, GLYATL1, GLYATL2, LOC100287413

Predefined gene sets in Hepamine

Data source	Pathways	Symbols
WIKIPATHWAYS	Amino Acid metabolism	ACAA1, ACADM, ACLY, ACO2, ADH1C, ADH4, ADH5, ADH7, ALDH1A1, ARG1, ARG2, ASNS, ASS1, ALDH7A1, AUH, BCAT1, BHMT, CAD, CBS, CPS1, CS, CTH, DBH, DDC, DLD, DLST, EHHADH, EPRS, FAH, FH, GCLM, GLS, GLUD1, GLUL, GOT1, GOT2, GSR, GSS, HADH, HAL, HDC, HMGCL, HMGCS2, HNMT, IARS, IDH1, LDHA, MAOA, MDH1, MDH2, MPST, MMUT, OAT, ODC1, OGDH, OTC, PC, PCK1, PDHA1, PDK4, PKM, PNMT, PYCR1, ALDH18A1, RARS, SDHA, SMS, SRM, TAT, TDO2, TH, TPH1, TPO, VARS, WARS, PDHX, AOC3, SUCLG1, P4HA2, FARSB, FTCD, SDS, HIBADH, LARS2, HIBCH, MCCC1, G6PC2, ACSS1, GPT2, MARS2, PPM1L
WIKIPATHWAYS	AMP-activated Protein Kinase (AMPK) Signaling	ACACA, ACACB, ADRA1B, ADRA1A, AKT1, AKT2, CCNA2, CCNB1, CDKN1A, CPT1A, CPT1B, EEF2, EIF4EBP1, ELAVL1, FASN, MTOR, GYS1, GYS2, HMGCR, HNF4A, INSR, LEP, LEPR, LIPE, BORCS8-MEF2B, PFKFB3, PIK3C3, PIK3CA, PIK3CB, PIK3CD, PIK3CG, PIK3R1, PIK3R2, PRKAA1, PRKAA2, PRKAB1, PRKAB2, PRKACB, PRKACG, PRKAG1, RPS6KB1, RPS6KB2, SLC2A4, SREBF1, STK11, TP53, TSC1, TSC2, PIK3R3, CCNA1, ADIPOQ, CAMKK2, PLCB1, EEF2K, ADIPOR1, PRKAG2, CAB39, PRKAG3, STRADB, SLC2A4RG, RPTOR, ADIPOR2, CAMKK1, STRADA, CPT1C, PPARGC1B, CRTC2, INS-IGF2, LOC101930123
WIKIPATHWAYS	Amplification and Expansion of Oncogenic Pathways as Metastatic Traits	JAG1, EPAS1, TNC, NOTCH1, PIK3CG, SRC, TCF7, TCF7L2, VCAM1, VEGFA, VHL, WNT2, CXCR4, CYTIP, POSTN, LEF1, TCF7L1
WIKIPATHWAYS	Amyotrophic lateral sclerosis (ALS)	APAF1, BAD, BAX, BCL2, BCL2L1, BID, CASP1, CASP3, CASP9, CAT, MAPK14, CST3, DAXX, GPX1, GRIA1, MAP3K5, NEFM, NEFH, NEFL, NOS1, PPP3CA, PPP3CB, PPP3CC, MAP2K2, MAP2K6, PRPH, RAB5A, RAC1, SLC1A2, SOD1, TNF, TNFRSF1A, TP53, CCS, TOMM40, ALS2, DERL1, CASP12

Predefined gene sets in Hepamine

Data source	Pathways	Symbols
WIKIPATHWAYS	Androgen receptor signaling pathway	AES, AKT1, KLK3, AR, RHOA, RHOB, BAG1, CCND1, BRCA1, CALR, CAV1, RUNX2, CCNE1, CDC42, CDKN1A, CREB1, CREBBP, CTNNB1, DAXX, EGFR, EP300, ETV5, FHL2, FKBP4, FOXO1, FLNA, GSK3B, HDAC1, JUN, LIMK2, SMAD3, SMAD4, MDM2, PRDX1, PIK3R1, PIK3R2, PTEN, PTK2, RAC1, RAD9A, RAN, RB1, RELA, RLN1, RNF4, RNF6, ROCK1, SMARCE1, SP1, SRC, STAT3, TGFB1I1, TGIF1, NR2C2, UBE2I, UBE3A, SUMO1, NCOA4, NCOA3, NR0B2, PIAS1, PLPP1, NCOA1, KAT2B, PIAS2, ROCK2, RNF14, NCOR1, NCOR2, STUB1, RACK1, PIAS3, CARM1, NCOA2, KAT5, DSTN, KAT7, PARK7, KDM1A, SIRT1, PATZ1, ZNF318, SIN3A, PSMC3IP, PIAS4, PAK6, ZMIZ1, EFCAB6, BMF, MIR1281, BUB1B-PAK6
WIKIPATHWAYS	Angiogenesis	AKT1, ANGPT1, ARNT, CREBBP, MAPK14, FGF2, FGFR2, FLT1, HIF1A, KDR, SMAD1, MMP9, NOS3, PDGFB, PDGFRA, PIK3CA, PLCG1, MAPK1, PTK2, SRC, TEK, TIMP2, TIMP3, VEGFA

Predefined gene sets in Hepamine

Data source	Pathways	Symbols
WIKIPATHWAYS	Angiopoietin Like Protein 8 Regulatory Pathway	AKT1, AKT2, CBL, CBLB, MAP3K8, CRK, MAPK14, CYP2B6, CYP3A4, CYP7A1, DIO2, EIF4E, EIF4EBP1, FASN, FBP1, FOXO1, FOXO3, FLOT2, MTOR, G6PC, RAPGEF1, GSK3A, GSK3B, GYS1, HRAS, INS, INSR, IRS1, LPL, MAP3K1, MAP3K3, MAP3K4, MAP3K5, MAP3K9, MAP3K10, MAP3K11, PCK1, PDPK1, PIK3C2A, PIK3C2G, PIK3C3, PIK3CA, PIK3CB, PIK3CD, PIK3CG, PIK3R1, PIK3R2, PRKAA1, PRKAA2, PRKAB1, PRKAB2, PRKAG1, MAPK1, MAPK3, MAPK4, MAPK6, MAPK7, MAPK8, MAPK11, MAPK9, MAPK10, MAPK13, MAP2K1, MAP2K2, MAP2K3, MAP2K5, MAP2K6, MAP2K7, PTPN1, MAP4K2, RAF1, RHEB, RPS6KA1, RPS6KA2, RPS6KA3, RPS6KB1, RPS6KB2, RXRA, MAPK12, SCD, MAP2K4, SHC1, SLC2A1, SLC2A4, SLC16A2, SOS1, SOS2, SREBF1, SREBF2, MAP3K7, THRA, THRB, TSC1, TSC2, MAP3K12, IRS4, MAP4K3, PIK3R3, IRS2, RPS6KA4, MAP3K14, MAP3K6, MAP3K13, RPS6KA5, TRIP10, MAP4K4, NR1H3, FLOT1, CAP1, MAP3K2, MAP4K5, MAP4K1, EXOC7, RHOQ, CBLC, SHC2, RPS6KA6, PIK3R4, MINK1, MLXIPL, PRKAG2, SHC3, PRKAG3, SLCO1C1, ANGPTL8, RPTOR, MLST8, ABCG5, ABCG8, MAPKAP1, SESN3, RICTOR
WIKIPATHWAYS	ApoE and miR-146 in inflammation and atherosclerosis	APOE, IRAK1, NFKB2, RELA, SPI1, TLR2, TLR4, TRAF4, MIR718
WIKIPATHWAYS	Apoptosis	AKT1, APAF1, BIRC2, BIRC3, XIAP, BIRC5, FAS, FASLG, BAD, BAK1, BAX, BCL2, BCL2L1, BCL2L2, BID, BNIP3L, BOK, CASP1, CASP2, CASP3, CASP4, CASP6, CASP7, CASP8, CASP9, CASP10, CDKN2A, CHUK, DFFA, DFFB, GZMB, HELLS, IGF1, IGF1R, IGF2, IKBKB, IRF1, IRF2, IRF3, IRF4, IRF5, IRF6, IRF7, JUN, LTA, MCL1, MDM2, MAP3K1, MYC, NFKB1, NFKBIA, NFKBIB, NFKBIE, PIK3R1, PMAIP1, PRF1, MAPK10, RELA, MAP2K4, TNF, TNFRSF1A, TNFRSF1B, TP53, TP73, TRAF1, TRAF2, TRAF3, IKBKG, TP63, TRADD, TNFRSF25, RIPK1, CRADD, HRK, TNFSF10, FADD, TNFRSF10B, CFLAR, SCAF11, BCL2L11, BBC3, TNFRSF21, CYCS, DIABLO, MIR3191, MIR7846

Predefined gene sets in Hepamine

Data source	Pathways	Symbols
WIKIPATHWAYS	Apoptosis Modulation and Signaling	APAF1, BIRC2, BIRC3, XIAP, BIRC5, FAS, FASLG, BAD, BAK1, BAX, BCL2, BCL2A1, BCL2L1, BCL2L2, BID, BIK, BLK, BNIP3, BOK, CAPNS1, CASP1, CASP2, CASP3, CASP4, CASP6, CASP7, CASP8, CASP9, CASP10, CDKN2A, DAXX, DFFA, DFFB, ENDOG, FOS, HSPA1A, IKBKB, IL1R1, IRAK1, JUN, MCL1, MAP3K5, MYD88, NAIP, NFKB1, NFKBIA, TNFRSF11B, PMAIP1, SEPT4, PRKD1, MAPK3, MAPK8, PTPN13, TNFRSF1A, TNFRSF1B, TP53, TRAF3, TRAF6, IL1R2, MADD, PEA15, TRADD, TNFRSF25, RIPK1, CRADD, HRK, TNFSF10, TNFRSF6B, FADD, TNFRSF10D, TNFRSF10C, TNFRSF10B, TNFRSF10A, CFLAR, MAP3K14, AIFM1, BAG3, BCL2L10, BCL2L11, BBC3, HTRA2, JPT1, PTRH2, CYCS, TOLLIP, PIDD1, DIABLO, BIRC6, BIRC7, AIFM2, BMF, MIR718, MIR3191, MIR7846
WIKIPATHWAYS	Apoptosis Modulation by HSP70	APAF1, FAS, FASLG, BID, CASP2, CASP3, CASP6, CASP7, CASP8, CASP9, HSPA1A, MAP3K1, NFKB1, MAPK10, TNFRSF1A, RIPK1, FADD, AIFM1, CYCS
WIKIPATHWAYS	Apoptosis-related network due to altered Notch3 in ovarian cancer	ABL1, AKT1, ANXA5, BIRC5, APOE, APP, BCL3, CASP7, CDKN1A, CDKN1B, CTNNA1, NQO1, ERBB3, ERN1, ETS1, F2R, PTK2B, GCLC, HDAC1, HELLS, NRG1, HSPA5, HSPB1, HSPD1, IL7R, JUND, SMAD7, NFKB1, PAK2, PKN1, MAPK1, PTK2, RPS6KB1, THBS1, TNF, TRAF1, VIM, CUL5, AXIN1, CUL1, YBX3, RIPK2, TNFRSF10B, IER3, SQSTM1, SOCS3, RNF7, NET1, VAV3, SERBP1, BEX3, TNFRSF21, CARD14, MIR8085
WIKIPATHWAYS	Arachidonate Epoxygenase / Epoxide Hydrolase	COX8A, CYP2C8, CYP2C9, CYP2J2, EPHX2, GSTP1, COX5A
WIKIPATHWAYS	Aripiprazole Metabolic Pathway	CYP2D6, CYP3A43

Predefined gene sets in Hepamine

Data source	Pathways	Symbols
WIKIPATHWAYS	Arrhythmogenic Right Ventricular Cardiomyopathy	ACTB, ACTG1, ACTN4, ACTN1, ACTN2, ACTN3, ATP2A2, CACNA1C, CACNA1D, CACNA1F, CACNA1S, CACNA2D1, CACNB1, CACNB2, CACNB3, CACNB4, CACNG1, CDH2, CTNNA1, CTNNA2, CTNNB1, DAG1, DES, DMD, DSC2, DSG2, DSP, EMD, GJA1, ITGA6, ITGA1, ITGA2, ITGA2B, ITGA3, ITGA4, ITGA5, ITGA7, ITGA9, ITGAV, ITGB1, ITGB3, ITGB4, ITGB5, ITGB6, ITGB7, ITGB8, JUP, LAMA2, LMNA, PKP2, RYR2, SGCA, SGCB, SGCD, SGCG, SLC8A1, TCF7, TCF7L2, ITGA10, ITGA8, CACNA2D2, CACNG3, CACNG2, ITGA11, CACNG5, CACNG4, CTNNA3, LEF1, CACNA2D3, CACNG8, CACNG7, CACNG6, TCF7L1, CACNA2D4, MIR935, LINC02478
WIKIPATHWAYS	Arylamine metabolism	NAT2, CYP1A2, SULT1A2, SULT1A1, UGT1A9, UGT1A4
WIKIPATHWAYS	Aryl Hydrocarbon Receptor	AHR, ARNT, CD36, CDK2, CDKN1A, CDKN1B, KLF6, CYP1A1, CYP1A2, CYP1B1, NQO1, E2F1, EGFR, EP300, ESR1, GCLC, HRAS, HSP90AA1, KRAS, LPL, MYC, NF1, NFE2L2, NFKB1, NRAS, PLAGL1, MAPK1, MAP2K1, PTGS2, RAF1, RB1, RELA, RET, CCL1, SRC, TNF, VEGFA, NRIP1, AIP, NCOR2, CDC37, FGF21, HPGDS, AHRR, PSRC1, NCOA7, MIR1181, MIR1281
WIKIPATHWAYS	Aryl Hydrocarbon Receptor Pathway	AHR, ALDH3A1, ARNT, BAX, CDKN1B, CYP1A1, CYP1A2, CYP1B1, NQO1, EGFR, EP300, GSTA2, HES1, HSP90AA1, IFNG, IGFBP1, IL1B, IL2, IL12A, IL12B, JUN, JUNB, JUND, MGST1, NFE2L2, SERPINB2, SRC, TGFB1, TNF, SLC7A5, NCOA1, AIP, CAP2, PTGES3, CDC37, CES3, MYOF, IL17B, POLK, UGT1A7, UGT1A6, UGT1A9, UGT1A4, UGT1A1, UGT1A3, AHRR, MIR1181, MIR1281

Predefined gene sets in Hepamine

Data source	Pathways	Symbols
WIKIPATHWAYS	Association Between Physico-Chemical Features and Toxicity Associated Pathways	ACTA1, AKT1, APC, RHOA, CBL, CDKN1A, CDKN1B, CTNNB1, DVL1, EGFR, ELK1, ERBB2, ERBB4, FN1, FZD2, GPX1, GRB2, GRIA1, GSK3B, JUN, MYC, MYL1, MYLK, NCK1, NEFM, NEFH, NEFL, NOS1, PAK1, PFN1, PFN2, PIK3CG, PLCG1, PPP2CA, MAPK1, MAPK8, MAP2K1, PTK2, RAF1, MAP2K4, SHC1, SOD1, SOS1, SRC, STAT5A, TGFA, TMSB4X, WNT11, FZD5, FZD3, AXIN1, FZD1, FZD4, FZD6, FZD7, FZD8, FZD9, CAMK1, ROCK2, ACTR3, ACTR2, FZD10, DAAM1, WNT16, PFN3, PFN4, MIR4683, MIR6808
WIKIPATHWAYS	ATM Signaling Network in Development and Disease	ATM, ATR, BUB1, CDK1, CDK5, CHEK1, ATF2, MTOR, G6PD, H2AFX, HMGN1, HSPB1, LBR, MAP3K5, MRE11, NBN, NFKB1, PTPA, PPP5C, PRKAA1, PRKDC, RASGRF1, RBBP8, STK11, TP53BP1, TSC2, SMC1A, PPM1D, IKBKG, CDK5R1, RNF8, AURKB, MDC1, HDAC4, RNF40, RAD50, TRIM28, KAT5, CHEK2, ATMIN, RIF1, RNF20, DCLRE1C, CEP63, RNF168, MIR4741
WIKIPATHWAYS	ATM Signaling Pathway	ABL1, ATM, BID, BRCA1, CASP2, CCNB1, CCNE1, CDK1, CDC25A, CDC25C, CDK2, CDKN1A, CHEK1, CREB1, ATF2, GADD45A, FANCD2, H2AFX, JUN, MDM2, MDM4, MRE11, NBN, NFKBIA, MAPK9, RAD9A, RAD51, TP53, TP53BP1, TP73, AP3B2, SMC1A, IKBKG, RIPK1, CRADD, MDC1, TLK1, RAD50, CHEK2, PIDD1
WIKIPATHWAYS	ATR Signaling	ATR, CHEK1, HUS1, RAD1, RAD9A, RPA1, TOPBP1, ATRIP, RAD9B

Predefined gene sets in Hepamine

Data source	Pathways	Symbols
WIKIPATHWAYS	B Cell Receptor Signaling Pathway	AKT1, BCL6, BLK, BRAF, BTK, CAMK2A, CBL, CD19, CD22, CD79A, CD79B, CD81, CDC42, CHUK, CR2, CREB1, ATF2, CRK, CRKL, MAPK14, E2F3, ELK1, ETS1, FOXO1, FYN, GAB1, GRB2, RAPGEF1, GSK3A, GSK3B, GTF2I, HCLS1, HRAS, IKBKB, ILF2, INPP5D, IRF4, JUN, LCK, LYN, MAX, MEF2C, MEF2D, MYC, NCK1, NFATC2, NFATC3, NFKB1, NFKBIA, PDPK1, PIK3CG, PIK3R1, PIK3R2, PLCG1, PLCG2, PRKCB, PRKCD, MAPK1, MAPK4, MAPK8, MAPK9, MAP2K1, MAP2K2, MAP2K6, PTPN6, PTPN11, PTPRC, RAC1, RAC2, RAF1, REL, RELA, RPS6KA1, SH3BP2, SHC1, SOS1, SYK, MAP3K7, TEC, VAV1, VAV2, LAT2, PIP5K1A, PIP5K1B, KLF11, IKBKG, BCL10, GAB2, MALT1, MAP4K1, PIP5K1C, RASGRP3, PTPN18, DAPP1, BLNK, CARD11, PIK3AP1, MIR5196
WIKIPATHWAYS	BDNF-TrkB Signaling	ADCY1, AKT1, BDNF, BRAF, CREB1, DLG4, EIF4EBP1, MTOR, GAB1, GRB2, GRIN1, HRAS, KRAS, NRAS, NTRK2, PIK3CG, PLCG1, MAPK1, MAP2K1, RHEB, RPS6KA1, RPS6KB1, SHC1, SOS1, TRPC3, TRPC6, TSC1, TSC2, MKNK1, HOMER1, GAB2, ARC, EEF2K, LOC101930123
WIKIPATHWAYS	Benzene metabolism	CYP2E1, NQO1, EPHX1, GSTM1, MPO, DHDH
WIKIPATHWAYS	Benzo(a)pyrene metabolism	AKR1C4, CYP1A1, CYP1B1, CYP3A4, AKR1C1, AKR1C2, EPHX1, AKR1C3, AKR1A1
WIKIPATHWAYS	Biogenic Amine Synthesis	AANAT, ACHE, ASMT, CHAT, COMT, DBH, DDC, GAD1, GAD2, HDC, MAOA, PAH, PNMT, TH, TPH1, MIR4761
WIKIPATHWAYS	Bladder Cancer	ARAF, CCND1, BRAF, CDH1, CDK4, CDKN1A, CDKN2A, DAPK1, DAPK3, HBEGF, E2F1, TYMP, EGF, EGFR, ERBB2, FGFR3, HRAS, CXCL8, KRAS, MDM2, MMP1, MMP2, MMP9, MYC, NRAS, PIK3R1, PIK3R2, MAPK1, MAP2K1, MAP2K2, RAF1, RB1, SRC, THBS1, TP53, UPK3A, VEGFA, RPS6KA5, RASSF1, DAPK2, MIR637

Predefined gene sets in Hepamine

Data source	Pathways	Symbols
WIKIPATHWAYS	Blood Clotting Cascade	F2, F3, F5, F7, F8, F9, F10, F11, F12, F13B, FGA, FGB, FGG, KLK1, KLKB1, SERPINE1, SERPINB2, PLAT, PLAU, PLG, SERPINF2, VWF, F8A1
WIKIPATHWAYS	BMP2-WNT4-FOXO1 Pathway in Human Primary Endometrial Stromal Cell Differentiation	BMP2, CTNNB1, DCN, FOXO1, SMAD1, SMAD5, SMAD9, SFRP1, SST, LEFTY2, BCL2L11, DKK1, WNT4
WIKIPATHWAYS	BMP Signaling Pathway in Eyelid Development	BMP4, EGFR, FGF10, FGFR2, FOXC1, FOXC2, INHBB, JUN, SMAD1, SMAD4, SMAD5, MAP3K1, NOTCH1, PITX2, MAPK3, MAPK9, SFRP1, SHH, TGFA, DKK2
WIKIPATHWAYS	Bone Morphogenic Protein (BMP) Signalling and Regulation	BMP2, BMPR1A, BMPR1B, BMPR2, RUNX2, SMAD1, SMAD4, SMAD6, NOG, TOB1, TOB2, SMURF1
WIKIPATHWAYS	Brain-Derived Neurotrophic Factor (BDNF) signaling pathway	ACACB, AKT1, ALPL, APC, RHOG, BAD, BDNF, BMP2, CAMK4, CAMK2A, CASP3, CDC42, CDH2, CDK5, CFL1, CHUK, CNR1, CREB1, MAPK14, CSNK2A1, CTNNB1, DLG1, DOCK3, DPYSL2, EEF2, EGR1, EGR2, EIF2S1, EIF4E, EIF4EBP1, ELK1, PTK2B, FOXO3, FOS, MTOR, FYN, GABRB3, GRB2, GRIA1, GRIA2, GRIA3, GRIN1, GRIN2B, GSK3B, HRAS, IKBKB, IRS1, JAK2, JUN, KCNA3, KCNN2, MARCKS, MAPT, MEF2A, MEF2C, MAP3K1, NCAM1, NCF2, NCK1, NFATC4, NFKB1, NFKBIA, NGF, NGFR, YBX1, NSF, NTF3, NTRK1, NTRK2, NTRK3, PDPK1, PIK3R1, PIK3R2, PLCG1, PPP2CA, PRKAA1, PRKAA2, PRKCD, MAPK1, MAPK3, MAPK7, MAPK8, MAPK9, MAPK10, MAP2K1, MAP2K2, MAP2K5, PTPN11, PTPRF, RAB3A, RAC1, RAF1, RAP1A, RASGRF1, RELA, RPS6, RPS6KA1, RPS6KA3, RPS6KB1, SORT1, SHC1, SPP1, SRC, STAT1, STAT3, STAT5A, STAT5B, CDKL5, SYN1, ADAM17, TIAM1, TRAF6, TSC2, VAV2, NCK2, IKBKG, CAMK1, IRS2, KSR1, CDK5R1, SQSTM1, EIF2S2, RPS6KA5, BCL2L11, RANBP9, RACK1, VAV3, SH2B2, IGF2BP1, MAP3K2, FRS3, FRS2, CYFIP1, CRTC1, GRIP1, SHC2, SH2B1, SHC3, DOK5, KIDINS220, LINGO1, SIRPA, SHC4, NCF1

Predefined gene sets in Hepamine

Data source	Pathways	Symbols
WIKIPATHWAYS	Breast cancer pathway	PARP1, AKT1, AKT2, APC, ARAF, ATM, ATR, BAK1, BAX, CCND1, BRCA1, BRAF, BRCA2, CDK4, CDK6, CDKN1A, CETN3, CSNK1A1, CSNK2A1, CSNK2A2, CSNK2B, CTNNB1, DDB2, GADD45A, DVL1, DVL2, DVL3, E2F1, E2F2, E2F3, EGF, EGFR, ERBB2, ESR1, ESR2, FGF1, FGF2, FGF3, FGF4, FGF5, FGF6, FGF7, FGF8, FGF9, FGF10, FGFR1, FLT4, FOS, MTOR, FZD2, GRB2, GSK3B, HRAS, HES1, IGF1, IGF1R, JAG2, JUN, KIT, KRAS, LRP6, LRP5, MRE11, MYC, GADD45B, NBN, NFKB2, NOTCH1, NOTCH2, NOTCH3, NOTCH4, NRAS, PGR, PIK3CA, PIK3CD, PIK3R1, PIK3R2, MAPK1, MAPK3, MAP2K1, MAP2K2, PTEN, RAD51, RAF1, RB1, RPS6KB1, RPS6KB2, SFRP4, SHC1, SKP1, SOS1, SOS2, SP1, TCF7, TCF7L2, TP53, WNT1, WNT2, WNT3, WNT5A, WNT6, WNT7A, WNT7B, WNT10B, WNT11, WNT2B, FZD5, FZD3, FGF23, NCOA3, AXIN1, AXIN2, FZD1, FZD6, FZD7, FZD8, FZD9, PIK3R3, TNFSF11, NCOA1, FGF18, FGF17, FGF16, FGF19, AKT3, FRAT1, RAD50, APC2, DLL3, GADD45G, FZD10, FRAT2, HEY1, HEY2, SHC2, FGF20, FGF21, HEYL, FGF22, DLL1, LEF1, WNT16, POLK, SHC3, WNT4, DLL4, WNT10A, WNT5B, TCF7L1, WNT3A, CSNK1A1L, CSNK2A3, HES5, SHC4, MIR4683, LOC101929777, MIR6808
WIKIPATHWAYS	Butyrate-induced histone acetylation	ACLY, AKT1
WIKIPATHWAYS	Caffeine and Theobromine metabolism	NAT2, CYP1A2, CYP2A6, XDH

Predefined gene sets in Hepamine

Data source	Pathways	Symbols
WIKIPATHWAYS	Calcium Regulation in the Cardiac Cell	ADCY1, ADCY2, ADCY3, ADCY5, ADCY6, ADCY7, ADCY8, ADCY9, ADRA1D, ADRA1B, ADRA1A, ADRB1, ADRB2, ADRB3, ANXA6, ARRB1, ARRB2, ATP1A4, ATP1B1, ATP1B2, ATP1B3, FXYD2, ATP2A2, ATP2A3, ATP2B1, ATP2B2, ATP2B3, CACNA1A, CACNA1B, CACNA1C, CACNA1D, CACNA1E, CACNA1S, CACNB1, CACNB3, CALM1, CALM2, CALM3, CALR, CAMK4, CAMK2A, CAMK2B, CAMK2D, CAMK2G, CASQ1, CASQ2, CHRM1, CHRM2, CHRM3, CHRM4, CHRM5, FKBP1A, GJA1, GJA3, GJA4, GJA5, GJA8, GJB1, GJB2, GJB3, GJB5, GNA11, GNAI1, GNAI2, GNAI3, GNAO1, GNAQ, GNAS, GNAZ, GNB1, GNB2, GNB3, GNG3, GNG4, GNG5, GNG7, GNG11, GNGT1, SFN, GRK4, GRK5, GRK6, ITPR1, ITPR2, ITPR3, KCNB1, KCNJ3, KCNJ5, PLCB3, PLN, PRKACA, PRKACB, PKIA, PKIB, PRKAR1A, PRKAR1B, PRKAR2A, PRKAR2B, PRKCA, PRKCB, PRKCD, PRKCE, PRKCG, PRKCH, PRKD1, PRKCQ, PRKCZ, RGS1, RGS2, RGS3, RGS4, RGS7, RGS10, RGS16, RYR1, RYR2, RYR3, SLC8A1, SLC8A3, YWHAB, YWHAE, YWHAG, YWHAH, YWHAZ, RGS5, CAMK1, RGS20, RGS11, RGS9, RGS6, GJC1, RGS19, RGS14, GNB5, GJB6, YWHAQ, PKIG, RGS17, GNG13, GNG2, GNG12, GJC2, GJD2, GNB4, RGS18, GJA9, GNG8, GJB4, ADCY4, MIR197, MIR6869
WIKIPATHWAYS	Caloric restriction and aging	AKT1, MTOR, IGF1, PRKAB2, TP53, NAMPT, PPARGC1A, SIRT1
WIKIPATHWAYS	Cannabinoid receptor signaling	ADCY1, ADCY7, ADORA2A, AHR, DAGLA, CNR1, CNR2, MAPK14, CYP1A1, CYP2C19, CYP2C9, CYP3A4, FAAH, PRKACA, PRKACB, PRKACG, PRKAR1A, PRKAR1B, PRKAR2B, MAPK1, MAPK3, MAPK8, MAPK11, MAPK9, MAPK10, MAPK13, MAPK12, DAGLB, NAPEPLD
WIKIPATHWAYS	Canonical and Non-canonical Notch signaling	ADAM10, JAG1, CHUK, HES1, RBPJ, JAG2, MFAP1, MFAP2, NOTCH1, NOTCH2, NOTCH3, NOTCH4, PSEN1, PSEN2, RRAS, DLK1, MAML1, DLL3, HEY1, CNTN6, DLL1, LEF1, DLL4, MAML3, DLK2, MAML2, DNER

Predefined gene sets in Hepamine

Data source	Pathways	Symbols
WIKIPATHWAYS	Canonical and Non-Canonical TGF-B signaling	BMP1, BMPR1A, BMPR2, MAPK14, LOX, LOXL1, LOXL2, SMAD2, SMAD3, SMAD4, MAPK8, MAPK9, TGFB1, TGFBR1, TGFBR2, GREM1, LOXL4
WIKIPATHWAYS	Cardiac Hypertrophic Response	AKT1, AKT2, CALM1, CALM2, CAMK2D, CDK7, CDK9, CHUK, MAPK14, EGF, FGF2, FGFR2, MTOR, GSK3B, GUCA1A, NRG1, IGF1, IKBKB, MEF2A, MAP3K1, NFATC2, NFKB1, NPPA, PDPK1, PLA2G2A, PPP3CA, PRKCA, PRKD1, PRKG1, MAPK1, MAPK3, MAPK7, MAPK8, MAP2K1, MAP2K2, MAP2K3, MAP2K5, MAP2K6, MAP2K7, RAC1, RAF1, MAP2K4, MAP3K7, TGFB1, TGFBR1, TNF, TNFRSF1A, IKBKG, MAP3K14, IKBKE, HDAC9, HDAC4, HDAC5, MAP4K1, HDAC7, MIR2861, MIR3960
WIKIPATHWAYS	Cardiac Progenitor Differentiation	ACTC1, ANPEP, BMP1, BMP4, MAPK14, NKX2-5, FGF2, GATA4, GSK3B, NRG1, FOXA2, IGF1, IGF2, INHBA, INS, ISL1, KDR, KIT, MEF2C, MYH6, MYL2, NCAM1, NODAL, NOTCH1, ROR2, PAX6, PDGFRA, POU5F1, SCN5A, SOX1, SOX2, TBXT, TBX5, TGFB1, THY1, TNNI3, TNNT2, CXCR4, NOG, DKK1, IRX4, MESP1, TBX20, SOX17, LIN28A, NANOG, MIXL1, WNT3A, MYLK3, ZFP42, SIRPA, MESP2, LIN28B
WIKIPATHWAYS	Catalytic cycle of mammalian Flavin-containing MonoOxygenases (FMOs)	FMO1, FMO2, FMO3, FMO4, FMO5

Predefined gene sets in Hepamine

Data source	Pathways	Symbols
WIKIPATHWAYS	Cell Cycle	ABL1, ATM, ATR, CCND1, BUB1, CCNA2, CCNB1, CCND2, CCND3, CCNE1, CCNH, CDK1, CDC6, CDC20, CDC25A, CDC25B, CDC25C, CDC27, CDK2, CDK4, CDK6, CDK7, CDKN1A, CDKN1B, CDKN1C, CDKN2A, CDKN2B, CDKN2C, CDKN2D, CHEK1, GADD45A, E2F1, E2F2, E2F3, E2F4, E2F5, EP300, SFN, GSK3B, HDAC1, HDAC2, SMAD2, SMAD3, SMAD4, MCM2, MCM3, MCM4, MCM5, MCM6, MCM7, MDM2, MYC, GADD45B, ORC1, ORC2, ORC4, ORC5, PCNA, PLK1, PRKDC, RAD21, RB1, RBL1, RBL2, SKP1, SKP2, TFDP1, TFDP2, TGFB1, TGFB2, TGFB3, TP53, TTK, WEE1, YWHAB, YWHAE, YWHAG, YWHAH, YWHAZ, ZBTB17, SMC1A, CDC7, CDC45, MAD1L1, CUL1, CDC14B, CDC14A, CDC23, CDC16, CCNA1, PKMYT1, SMC3, CCNB2, CCNE2, BUB3, PTTG1, ESPL1, RBX1, STAG1, ANAPC10, MAD2L2, STAG2, PTTG2, GADD45G, DBF4, YWHAQ, CHEK2, ORC6, ORC3, ANAPC13, SMC1B, ANAPC2, ANAPC4, FZR1, ANAPC5, ANAPC7, ANAPC11, ANAPC1, CCNB3, WEE2, MIR1281, MIR3610
WIKIPATHWAYS	Cell Differentiation - Index	TLX1, TLX2, MEF2A, MEF2C, MEF2D, MYOD1, SRF, STAT3, LEFTY2, HDAC5, LEFTY1, TLX3, MIR1-1, MIR128-1, MIR150, MIR16-1, MIR16-2, MIR181A2, MIR181B1, MIR181B2, MIR181C, MIR199A1, MIR199A2, MIR203A, MIR181A1, MIR221, MIR222, MIR302A, MIR9-1, MIR133B, MIR302C, MIR302D, MIR146B, MIR181D, MEF2B, MIR302E, MIR3074, MIR3591
WIKIPATHWAYS	Cell Differentiation - Index expanded	RUNX2, EZH2, TLX1, TLX2, ID2, MEF2A, MEF2C, MEF2D, MYOD1, PAX7, SOX2, SRF, STAT3, LEFTY2, KLF4, HDAC5, LEFTY1, TLX3, MIR1-1, MIR128-1, MIR145, MIR150, MIR16-1, MIR16-2, MIR181A2, MIR181B1, MIR181B2, MIR181C, MIR199A1, MIR199A2, MIR203A, MIR206, MIR181A1, MIR221, MIR222, MIR26A1, MIR26A2, MIR302A, MIR9-1, MIR133B, MIR302C, MIR302D, MIR146B, MIR181D, MEF2B, MIR302E, MIR3074, MIR3591

Predefined gene sets in Hepamine

Data source	Pathways	Symbols
WIKIPATHWAYS	Cell-type Dependent Selectivity of CCK2R Signaling	DAGLA, CCK, CCKBR, CD38, CNR1, GNAI1, GNAQ, ITPR1, RYR1, RYR2, RYR3, TRPC1, PLCB1
WIKIPATHWAYS	Chemokine signaling pathway	ADCY1, ADCY2, ADCY3, ADCY5, ADCY6, ADCY7, ADCY8, ADCY9, GRK2, AKT1, AKT2, ARRB1, ARRB2, CXCR5, BRAF, CDC42, CHUK, CCR1, CCR3, CCR4, CCR6, CCR7, CCR8, CRK, CRKL, CSK, CX3CR1, DOCK2, PTK2B, FGR, FOXO3, GNAI1, GNAI2, GNAI3, GNB1, GNB2, GNB3, GNG3, GNG4, GNG5, GNG7, GNG10, GNG11, GNGT1, GNGT2, CCR10, XCR1, CXCR3, GRK4, GRK5, GRK6, GRB2, CXCL3, GSK3B, HCK, HRAS, IKBKB, CXCR2, CXCL10, ITK, JAK2, JAK3, KRAS, LYN, CXCL9, NFKB1, NFKBIA, NFKBIB, NRAS, PAK1, PF4, PIK3CA, PIK3CB, PIK3CD, PIK3CG, PIK3R1, PIK3R2, PLCB2, PLCB3, PLCB4, PPBP, PRKACB, PRKACG, PRKCB, PRKCD, PRKCZ, MAPK1, MAPK3, MAP2K1, PRKX, PTK2, PXN, RAC1, RAC2, RAF1, RAP1A, RAP1B, RELA, ROCK1, CCL1, CCL3, CCL4, CCL5, CCL7, CCL11, CCL15, CCL17, CCL19, CCL20, CCL21, CCL22, CCL24, CCL25, CXCL11, CXCL5, XCL1, CX3CL1, CXCL12, SHC1, SOS1, SOS2, STAT1, STAT2, STAT3, STAT5B, TIAM1, VAV1, VAV2, WAS, CXCR4, PIK3R3, IKBKG, WASL, ROCK2, CXCL14, BCAR1, ELMO1, AKT3, RASGRP2, CCL26, VAV3, CXCL13, CXCR6, GNB5, CCR9, CCL27, PLCB1, PIK3R5, SHC2, TIAM2, GNG13, SHC3, GNG2, GNG12, PARD3, CCL28, PREX1, CXCL16, GNB4, GNG8, ADCY4, SHC4, MIR197, NCF1, CCR2
WIKIPATHWAYS	Cholesterol Biosynthesis	CYP51A1, DHCR7, FDFT1, FDPS, HMGCR, HMGCS1, IDI1, LSS, MVD, MVK, MSMO1, SC5D, SQLE, PMVK, NSDHL

Predefined gene sets in Hepamine

Data source	Pathways	Symbols
WIKIPATHWAYS	Chromosomal and microsatellite instability in colorectal cancer	AKT1, AKT2, APC, BIRC5, ARAF, RHOA, BAD, BAK1, BAX, CCND1, BCL2, BRAF, CASP3, CASP9, CDKN1A, CSNK1A1, CTNNB1, DCC, DDB2, GADD45A, FOS, GSK3B, MSH6, JUN, KRAS, SMAD2, SMAD3, SMAD4, MLH1, MSH2, MSH3, MYC, GADD45B, PMAIP1, MAPK1, MAPK3, MAPK8, MAPK9, MAPK10, MAP2K1, PTGS2, RAC1, RAC2, RAC3, RAF1, RALA, RALB, RALGDS, REL, TCF7, TCF7L2, TGFB1, TGFB2, TGFB3, TGFBR1, TGFBR2, TP53, AXIN1, AXIN2, NTN1, AKT3, BCL2L11, APC2, GADD45G, APPL1, BBC3, TBK1, LEF1, POLK, CYCS, EXOC2, TCF7L1, CSNK1A1L, MIR3191

Predefined gene sets in Hepamine

Data source	Pathways	Symbols
WIKIPATHWAYS	Circadian rythm related genes	AANAT, ADA, ADCY1, ADORA1, ADORA2A, AGRP, AHCY, AHR, FAS, ARNT, ARNTL, ZFHX3, ATF4, AVP, KLF9, CDK4, CHRM1, CHRNB2, CLDN4, CPT1A, CREB1, CREM, CRH, CRX, CRY1, CRY2, CSNK1D, CSNK1E, CST3, DBP, DDC, DDX5, DHX9, DRD1, DRD2, DRD3, DRD4, DYRK1A, EGR1, EGR3, EP300, NR2F6, EZH2, F7, GFPT1, GHRH, GNA11, GNAQ, UTS2R, GSK3B, HCRTR1, HCRTR2, HDAC1, HDAC2, HNRNPD, HNRNPU, HTR7, ID2, ID3, ID4, IL6, JUN, JUND, KCND2, KCNMA1, LEP, MC3R, KMT2A, MTNR1A, MTNR1B, MTTP, NAGLU, NFIL3, NGFR, NONO, NOS2, NPAS2, NPY2R, NTRK1, NTRK3, OPRL1, SERPINE1, PAX4, PER1, PML, PPARA, PPARG, PPP1CA, PPP1CB, PPP1CC, PRF1, PRKAA1, PRKAA2, PRKCG, PRKDC, PRKG2, MAPK8, MAPK9, MAPK10, PROX1, PTEN, PTGDS, KDM5A, RBM4, RELB, RORA, RORB, RORC, RPS27A, SFPQ, SFTPC, SIX3, SKP1, SLC6A4, SLC9A3, SREBF1, STAR, SUV39H1, HNF1B, TH, KLF10, NKX2-1, TOP1, TOP2A, TP53, TPH1, TYMS, UBA52, UBC, UBE3A, NRIP1, SOX14, CUL1, OGT, BHLHE40, TNFRSF11A, HDAC3, PER3, PER2, TIMELESS, BTRC, USP2, MTA1, ADIPOQ, HOMER1, ROCK2, MAGED1, NR1D1, CLOCK, CARTPT, NCOR1, ARNT2, HS3ST2, THRAP3, NR1D2, NR1H3, NAMPT, PRMT5, NCOA2, MYBBP1A, RAI1, PPARGC1A, UTS2, ATF5, NLGN1, SETX, PHLPP1, FBXW11, CRTC1, SIRT1, OPN3, NOCT, SIN3A, FBXL3, HEBP1, GHRL, MAGEL2, PSPC1, LGR4, METTL3, ARNTL2, CCAR2, PROK2, BHLHE41, SUV39H2, RBM4B, PROK1, CIPC, KCNH7, OPN4, CAVIN3, BTBD9, TPH2, PROKR2, NMS, PASD1, CIART, SIK1, ATOH7, NPS, MIR1281, MIR3064, MIR5047, MIR4751, MIR5572, MIR6883, TPTEP2-CSNK1E
WIKIPATHWAYS	Cocaine metabolism	BCHE, CES1, CYP3A4, CES2
WIKIPATHWAYS	Codeine and Morphine Metabolism	ABCC2, CYP2C8, CYP2D6, CYP3A4, ABCB1, UGT2B4, UGT2B7, ABCC3, SLCO1B1, UGT1A10, UGT1A8, UGT1A6, UGT1A9, UGT1A1, UGT1A3
WIKIPATHWAYS	Colchicine Metabolic Pathway	CYP3A4

Predefined gene sets in Hepamine

**Data source**	**Pathways**	**Symbols**
WIKIPATHWAYS	Common Pathways Underlying Drug Addiction	ACTB, ACTG1, ACTG2, ADCY1, ADCY8, ARAF, CALM1, CALM2, CAMK4, CAMK2A, CREB1, DRD1, DRD2, DRD4, GJB1, GNAI1, GNAS, GRIA1, GRIA2, GRIA3, GRIA4, GRIN1, GRIN2A, GRM1, GRM5, PPP1CA, PPP1CB, PPP1CC, PPP1R1A, PRKACA, PRKACB, PRKACG, PRKCA, PRKCB, PRKCG, MAPK1, MAPK3, MAP2K1, MAP2K2, RAF1, RAP1A, RAP1B
WIKIPATHWAYS	Complement Activation	CFB, C1QA, C1QB, C1QC, C1R, C1S, C2, C3, C4A, C4B, C5, C6, C7, C8A, C8B, C8G, C9, CD55, CFD, CFP, MASP1, MASP2
WIKIPATHWAYS	Complement and Coagulation Cascades	SERPINC1, BDKRB1, CFB, SERPING1, C1QA, C1QB, C1QC, C1R, C1S, C2, C3, C3AR1, C4B, C5AR1, C6, C7, C8G, C9, CLU, CLTC, CPB2, CR1, CR2, CD55, CFD, F2, F2R, F3, F5, F7, F8, F9, F10, F12, F13B, FGB, SERPIND1, CFH, CFI, KLKB1, KNG1, LMAN1, CD46, SERPINE1, SERPINA5, SERPINA1, PLAT, PLAU, PLAUR, PLG, SERPINF2, PROC, PROS1, MASP1, TFPI, THBD, VWF, MASP2, MIR6843
WIKIPATHWAYS	Composition of Lipid Particles	APOA1, APOA2, APOB, APOC3, APOE, CETP, LCAT, LDLR, LPL, MIR6886
WIKIPATHWAYS	Constitutive Androstane Receptor Pathway	ALAS1, ABCC2, CYP2A6, CYP2B6, CYP2C19, CYP2C9, CYP3A4, CYP3A5, CYP4A11, EHHADH, FOXO1, GSTA2, HSP90AA1, ABCB1, PTPA, RXRA, SP1, SULT1A1, SULT2A1, DNAJC7, SMC1A, NCOA1, ABCC3, NR1I3, NCOA2, PPARGC1A, NCOA6, UGT1A6, UGT1A9, UGT1A4, UGT1A1, UGT1A3
WIKIPATHWAYS	Copper homeostasis	ADAM10, AKT1, APC, XIAP, APP, ATOX1, ATP7A, ATP7B, CCND1, CASP3, SLC31A1, SLC31A2, COX11, FOXO1, FOXO3, GSK3B, JUN, MAPT, MDM2, MT1A, MT1B, MT1E, MT1F, MT1G, MT1H, MT1JP, MT1L, MT1X, MT2A, MT3, MTF1, SLC11A2, PIK3CA, PRNP, PTEN, SCO1, SOD1, SOD3, SP1, ADAM17, TP53, ADAM9, CCS, SCO2, COX17, MTF2, BACE1, STEAP1, XAF1, STEAP3, STEAP4, MT4, COMMD1, STEAP2

Predefined gene sets in Hepamine

Data source	Pathways	Symbols
WIKIPATHWAYS	Cori Cycle	G6PD, GAPDH, GPI, GPT, HK1, INS, LDHA, PFKP, PGAM1, PGK1, PGK2, SLC2A1, SLC2A2, SLC2A4, TALDO1, TPI1
WIKIPATHWAYS	Corticotropin-releasing hormone signaling pathway	ACACA, PARP1, GRK3, AKT1, RHOA, ARRB1, ARRB2, BCL2, BRAF, CAMK2A, CASP3, CASP9, CREB1, CRH, CRHBP, CRHR1, CRHR2, MAPK14, CTNNB1, CYP11A1, CYP11B1, CYP21A2, ECE1, ELK1, ERN1, FOS, FOSB, FOSL2, GJA1, GNA11, GNAI1, GNAI2, GNAO1, GNAQ, GNAS, GNAZ, GNB1, GNB2, GNB3, GRK6, GSK3B, NR4A1, HSD3B1, HSD3B2, HSP90AA1, IL2, CXCL8, IL18, IVL, JUNB, JUND, JUP, KRT1, KRT14, MAP3K5, NFKB1, NFKBIA, NOS3, NR4A2, PLCG1, PLCG2, POMC, PRKAA2, PRKCA, PRKCB, PRKCD, PRKCI, PRKCQ, MAPK1, MAPK3, MAPK8, MAPK9, MAP2K1, PTK2, RAF1, RAP1B, RELA, SP1, STAR, SULT2A1, TCF4, TFAP2A, TGFB1, TLR4, FOSL1, CACNA1H, TBX19, TRIM28, RAPGEF3, NCOA2, GNB5, CASP12, LINC02210-CRHR1
WIKIPATHWAYS	Cytokines and Inflammatory Response	CD4, CSF1, CSF2, CSF3, CXCL1, CXCL2, HLA-DRA, HLA-DRB1, IFNB1, IFNG, IL1A, IL1B, IL2, IL3, IL4, IL5, IL6, IL7, IL10, IL11, IL12B, IL13, IL15, PDGFA, TGFB1, TNF, LOC105369230
WIKIPATHWAYS	Cytoplasmic Ribosomal Proteins	FAU, RPSA, RPL10A, RPL3, RPL3L, RPL4, RPL5, RPL6, RPL7, RPL7A, RPL8, RPL9, RPL10, RPL11, RPL12, RPL13, RPL15, RPL17, RPL18, RPL18A, RPL19, RPL21, RPL22, RPL23A, RPL24, RPL26, RPL27, RPL30, RPL27A, RPL28, RPL29, RPL31, RPL32, RPL34, RPL35A, RPL37, RPL37A, RPL38, RPL39, RPL41, RPL36A, RPLP0, RPLP1, RPLP2, RPS2, RPS3, RPS3A, RPS4X, RPS4Y1, RPS5, RPS6, RPS6KA1, RPS6KA2, RPS6KA3, RPS6KB1, RPS6KB2, RPS7, RPS8, RPS9, RPS10, RPS11, RPS12, RPS13, RPS14, RPS15, RPS15A, RPS16, RPS17, RPS18, RPS19, RPS20, RPS21, RPS23, RPS24, RPS25, RPS26, RPS27, RPS27A, RPS28, RPS29, UBA52, RPL14, RPL23, MRPL19, RPL35, RPL13A, RPL36, RPS6KA6, LOC101929876, MIR6805, MIR6850

Predefined gene sets in Hepamine

Data source	Pathways	Symbols
WIKIPATHWAYS	Cytosine methylation	DNMT1, IDH1, IDH2, MECP2, TDG, MBD3, TET2, TET1, TET3
WIKIPATHWAYS	DDX1 as a regulatory component of the Drosha microprocessor	ATM, DDX1, MRE11, NBN, RAD50, DROSHA, DGCR8, MIR1306, MIR3618
WIKIPATHWAYS	Degradation pathway of sphingolipids, including diseases	ARSA, GLA, GLB1, GM2A, HEXA, NEU1, NEU2, NEU3, NEU4
WIKIPATHWAYS	Deregulation of Rab and Rab Effector Genes in Bladder Cancer	RAB27A, RAB27B, MADD, RPH3AL, GCC2, RPH3A, EXPH5, MYRIP, SYTL2, MLPH, TBC1D10A, SYTL1, SYTL3, SYTL4, SYTL5, UNC13D
WIKIPATHWAYS	Development and heterogeneity of the ILC family	AHR, AREG, GATA3, GFI1, ID2, IFNG, IL1B, IL4, IL5, IL6, IL7, IL9, IL12A, IL12B, IL13, IL15, IL17A, IL18, NFIL3, RORA, HNF1A, TNF, ZBTB16, EOMES, TOX, TBX21, IL22, IL23A, IL25, BCL11B, TSLP, IL33
WIKIPATHWAYS	Development of pulmonary dendritic cells and macrophage subsets	RUNX2, CSF1, CSF2, FLT3LG, IRF8, ID2, IRF4, SPI1, STAT3, TCF4, TPO, IKZF1, BATF3
WIKIPATHWAYS	Diclofenac Metabolic Pathway	CYP2B6, CYP2C19, CYP2C8, CYP2C18
WIKIPATHWAYS	Differentiation of white and brown adipocyte	BMP2, BMP4, BMP7, CEBPA, CEBPB, CEBPG, CIDEA, HOXC8, HOXC9, LEP, SMAD1, SMAD5, SMAD9, PPARG, ZIC1, ADIPOQ, MPZL2, PPARGC1A, ZNF423, HSPB7, PLAC8, SLC7A10, PRDM16, PPARGC1B, EBF3
WIKIPATHWAYS	Differentiation Pathway	ALK, BMP4, CNTF, CSF1, CSF1R, EGF, EPO, FGF1, FGF2, FGF4, FGF8, FGF10, FLT3LG, HGF, IGF1, IL3, IL6, IL6R, CXCR1, IL11, INHBA, INS, KIT, KITLG, NODAL, NOTCH1, NT5E, NTF4, PDGFA, PDGFB, SHH, TF, TGFB1, TGFB3, TPO, VEGFA, WNT1, WNT2, WNT5A, WNT7B, WNT2B, GDF5, TNFSF11, NOG, FST, LEFTY1, DKK1, WNT3A
WIKIPATHWAYS	Disorders of Folate Metabolism and Transport	ATIC, DHFR, FOLR1, GART, MTHFD1, MTHFR, MTR, SHMT2, TYMS, MTHFS, ALDH1L1, FTCD, SLC46A1
WIKIPATHWAYS	Disorders of the Krebs cycle	DLST, FH, PDHX, SUCLG2, SUCLG1, SUCLA2, DHTKD1

Predefined gene sets in Hepamine

Data source	Pathways	Symbols
WIKIPATHWAYS	DNA Damage Response	ABL1, APAF1, FAS, ATM, ATR, BAX, CCND1, BID, BRCA1, CASP3, CASP8, CASP9, CCNB1, CCND2, CCND3, CCNE1, CDK1, CDC25A, CDC25C, CDK2, CDK4, CDK5, CDK6, CDKN1A, CDKN1B, CHEK1, CREB1, DDB2, GADD45A, E2F1, FANCD2, SFN, H2AFX, MDM2, MRE11, MYC, GADD45B, NBN, PMAIP1, PML, PRKDC, RAD1, RAD9A, RAD17, RAD51, RAD52, RB1, RFC1, RPA2, TP53, SMC1A, TNFRSF10B, CCNB2, CCNE2, TLK1, RAD50, GADD45G, TLK2, CHEK2, BBC3, SESN1, RRM2B, CYCS, PIDD1, TP53AIP1, ATRIP, CCNB3, HUS1B, MIR3191
WIKIPATHWAYS	DNA Damage Response (only ATM dependent)	ABL1, AKT1, AKT2, APC, FASLG, RHOA, ATM, BAD, BAK1, BAX, CCND1, BCL2, BCL6, BIK, CAT, CCND2, CCND3, CCNG2, CDC42, CDKN1A, CDKN1B, CDKN2A, CTNNB1, DVL1, DVL2, DVL3, ERBB2, FOXO3, G6PC, GRB2, GSK3B, HMGB1, HRAS, INSR, IRS1, JUN, KRAS, LDLR, SMAD3, SMAD4, MDM2, MAP3K1, MAP3K4, MYC, NFKB1, NFKB2, NRAS, PCK2, PDK1, PIK3C2A, PIK3C2B, PIK3C2G, PIK3C3, PIK3CA, PIK3CB, PIK3CD, PIK3CG, PIK3R1, PIK3R2, PLAU, PMAIP1, PPP2R5C, PPP2R5E, MAPK1, MAPK8, MAPK9, MAPK10, PTEN, RAC1, RAC2, RAC3, RBL2, SCP2, SHC1, SOD2, SOS1, SOS2, MAP3K7, TCF7, TCF7L2, TGFB1, TP53, TP73, WNT1, WNT2, WNT3, WNT5A, WNT6, WNT7A, WNT7B, WNT10B, WNT11, WNT2B, FOSL1, AXIN1, PIK3R3, AKT3, BCL2L11, FRAT1, PIK3R5, BBC3, PIK3R4, LEF1, WNT16, WNT4, WNT10A, WNT5B, TCF7L1, WNT3A, MLKL, SOD2-OT1, MIR3191, LOC101929777, MIR6886, MIR6808

Predefined gene sets in Hepamine

Data source	Pathways	Symbols
WIKIPATHWAYS	DNA IR-damage and cellular response via ATR	PARP1, ATM, ATR, BARD1, BCL6, BRCA1, BRCA2, CDK1, CDC25C, CDK2, CHEK1, CLK2, E2F1, FANCA, FANCD2, FEN1, FOXM1, H2AFX, HUS1, MCM2, MDM2, MLH1, MRE11, MSH2, NBN, PCNA, PLK1, PML, POLB, PRKDC, RAD1, RAD9A, RAD17, RAD51, RAD52, RBBP8, RECQL, UPF1, RPA1, RPA2, SMARCC2, SP1, TOP3A, TP53, TP53BP1, UBE2D3, USP1, WRN, XPA, XRCC5, SEM1, SMC1A, CDC45, PPM1D, IKBKG, HERC2, EXO1, RECQL5, EEF1E1, MDC1, DCLRE1A, RAD50, TRIM28, TOPBP1, CEP164, SMARCAL1, UIMC1, RFWD3, FANCI, TDP1, CLSPN, BRCC3, MCPH1, PALB2, RMI1, BRIP1, ATRIP, ABRAXAS1, POLN, C7orf76, LOC100506136, MIR4741
WIKIPATHWAYS	DNA IR-Double Strand Breaks (DSBs) and cellular response via ATM	ABL1, ACTL6A, PARP1, APAF1, ATM, ATR, BAK1, BAX, BID, BLM, BRCA1, BRCA2, CASP3, CASP9, CDC25C, CDK5, CDKN2A, CHEK1, ATF2, E2F1, FANCD2, H2AFX, HSF1, MDM2, MRE11, NBN, PCNA, PRKDC, RAD9A, RAD17, RAD51, RAD52, UPF1, STK3, TERF2, TP53, TP53BP1, TP73, TRAF6, SMC1A, RNF8, LATS1, SMC3, EXO1, MDC1, RAD50, TRIM28, YAP1, KAT5, RASSF1, CHEK2, RIF1, DCLRE1C, NABP2, MCPH1
WIKIPATHWAYS	DNA Replication	CDC6, CDK2, MCM2, MCM3, MCM4, MCM5, MCM6, MCM7, ORC1, ORC2, ORC4, ORC5, PCNA, POLA1, POLD1, POLD2, POLE, POLE2, PRIM1, PRIM2, RFC1, RFC2, RFC3, RFC4, RFC5, RPA1, RPA2, RPA3, UBA52, UBC, CDC7, CDC45, POLD3, DBF4, ORC6, ORC3, POLA2, RPA4, GMNN, MCM10, POLD4, CDT1
WIKIPATHWAYS	Dopamine metabolism	COMT, DDC, NQO1, MAOA, MAOB, PPP2CA, PPP2CB, PRKACA, PRKACB, PRKACG, SOD1, TH, TYR, MIR4761
WIKIPATHWAYS	Dopaminergic Neurogenesis	ALDH1A1, ASCL1, CDKN1C, DDC, EN1, EN2, FGF8, GBX2, GLI1, GLI2, FOXA2, LMX1A, LMX1B, MSX1, NEUROD1, NKX2-2, NKX6-1, NR4A2, OTX2, PITX3, RET, SHH, SLC6A3, SLC18A2, SOX2, STAT3, TGFB1, TH, WNT1, NEUROG2

Predefined gene sets in Hepamine

Data source	Pathways	Symbols
WIKIPATHWAYS	Drug Induction of Bile Acid Pathway	BAAT, ABCC2, CYP3A4, CYP7A1, ABCB1, SLC10A1, SULT2A1, VDR, ABCB11, ABCC3, NR1I2, NR1I3, NR1H4, ABCC4, SLCO1B1, SLC51B, SLC51A
WIKIPATHWAYS	Dual hijack model of Vif in HIV infection	RUNX1, CBFB, ELOC, ELOB, UBB, CUL5, RBX1, APOBEC3G, LOC100506403
WIKIPATHWAYS	EBV LMP1 signaling	CHUK, HSP90AA1, IFNB1, IKBKB, CXCL8, IRAK1, MAP3K3, NFKB1, NFKB2, NFKBIA, MAPK1, MAPK8, RELA, CCL5, CCL20, MAP3K7, TNF, TRAF1, TRAF6, IKBKG, TRADD, MAP3K14, PDLIM7, MIR718
WIKIPATHWAYS	Ectoderm Differentiation	AES, ARHGDIG, ASTN1, BMP4, BMPR1A, CDH6, CDH8, CTBP1, CTNNA2, CTNNB1, CTNND2, DMD, EDA, CELSR2, FGFR2, FHL2, FOXL1, FYN, GATA6, GLB1, GLI3, HMGB2, FOXA2, JUP, KIFC3, KRT6A, LHX1, LY6E, SMAD4, MECP2, MYC, NF2, NFATC1, ROR2, NUMA1, TNFRSF11B, SIX6, CLDN11, PAX3, PAX6, PDE7A, PGM1, SERPINB6, PIM1, PI4KA, PLXNA2, PODXL, POU2F2, PPARD, PTPN13, PTPRB, RIT1, RRBP1, CCL2, SDCBP, SHH, SKIL, SNCA, SOX2, STC1, ELOVL4, TBL1X, TCF3, TFAP2A, TFAP2C, NR2F2, TRPM2, UBTF, WNT1, MZF1, ZBTB16, FZD5, ST8SIA4, FZD4, FZD8, HIST1H2BH, OGT, PPFIBP2, STX16, HESX1, SOCS2, TSC22D1, LDB2, GREB1, PAN2, MAFB, HDAC6, SPRY2, ABCC4, CAP2, GAS2L1, BAZ1A, NLGN1, PHF8, ARHGEF9, VAX2, TSKU, TOX3, SGSM3, SCHIP1, CDON, ZBTB7B, ZNF219, PRKAG2, TRIM33, NLK, RAB8B, KCNK10, WDR44, AHI1, BCAS3, BCOR, ELOVL2, MKS1, ARHGAP15, ANKS1B, C1GALT1, RGMA, SMURF1, MYORG, GRAMD1B, ZBTB2, EDA2R, ARHGAP10, NARS2, ZFHX4, CCDC130, TCF7L1, HDAC10, BOC, SORCS1, RHPN1, TTC14, JAKMIP1, CLVS1, ARX, PLCXD3, MIR15B, MIR34C, CCDC88C, MIR361, MIR1469, MIR4683, LOC101928583
WIKIPATHWAYS	EDA Signalling in Hair Follicle Development	BMP1, EDA, GLI1, LTB, PTCH1, RELB, SHH, WNT3, EDAR, DKK1, SOSTDC1, DKK4, EDARADD, LOC101929777
WIKIPATHWAYS	Effects of Nitric Oxide	AOX1, HBA1, MB, COX1, NOS1, NOS2, NOS3, XDH

Predefined gene sets in Hepamine

Data source	Pathways	Symbols
WIKIPATHWAYS	EGF/EGFR Signaling Pathway	ABL1, AP2A1, AP2B1, AKT1, ARF6, ATF1, BRAF, CAMK2A, CAV1, CAV2, CBL, CBLB, CDC42, CFL1, AP2M1, AP2S1, CREB1, CRK, CRKL, MAPK14, CSK, DNM1, E2F1, EGF, EGFR, EIF4EBP1, ELK1, ELK4, EPS8, EPS15, ERBB2, PTK2B, FOXO1, FOS, FOSB, MTOR, GAB1, GJA1, GRB2, GRB10, HRAS, INPP5D, INPPL1, JAK1, JAK2, JUN, JUND, KRAS, STMN1, LIMK2, MEF2A, MEF2C, MEF2D, MAP3K1, MAP3K3, MAP3K4, FOXO4, COX2, MYBL2, NCK1, NEDD4, NEDD8, NOS3, PEBP1, PAK1, PCNA, PDPK1, PIK3C2B, PIK3R1, PIK3R2, PLCG1, PLD1, PLD2, PLSCR1, PRKCA, PRKCB, PRKCD, PRKCI, PRKCZ, MAPK1, MAPK4, MAPK7, MAPK8, MAPK9, MAP2K1, MAP2K2, MAP2K5, PTEN, PTK2, PTK6, PTPN11, PTPN12, PTPRR, RAB5A, RAC1, RAF1, RALA, RALB, RALGDS, RAP1A, RASA1, ROCK1, RPS6KA1, RPS6KA2, RPS6KA3, RPS6KB1, ATXN2, SH3GL2, SH3GL3, SHC1, SOS1, SOS2, SP1, SRC, STAT1, STAT3, STAT5A, STAT5B, AURKA, STXBP1, TWIST1, VAV1, VAV2, PXDN, STAM, NCOA3, NCK2, IQGAP1, SYNJ1, DOK2, SH2D2A, USP8, ARHGEF1, HGS, REPS2, RPS6KA5, BCAR1, RIN1, USP6NL, GAB2, IQSEC1, ABI1, TNK2, SPRY2, STAM2, PIAS3, VAV3, STAMBP, MAP3K2, RALBP1, MAP4K1, CBLC, EPN1, SH3KBP1, ASAP1, NDUFA13, PLCE1, ERRFI1, EPS15L1, ITCH, PTPN5, RICTOR, MIR3917, LOC100505585
WIKIPATHWAYS	Eicosanoid Synthesis	ALOX12, ALOX5, ALOX5AP, ALOX15, ALOX15B, DPEP1, GGT1, LTA4H, LTC4S, PLA2G2A, PLA2G4A, PLA2G5, PTGDS, PTGIS, PTGS1, PTGS2, TBXAS1, PLA2G6, PTGES, PNPLA8, PTGES2, PNPLA3, GGTLC2, GGTLC1, GGT2, PLA2G4B, LOC102724197
WIKIPATHWAYS	eIF5A regulation in response to inhibition of the nuclear export system	EIF5A, XPO1, IGF2BP1, XPO4

Predefined gene sets in Hepamine

Data source	Pathways	Symbols
WIKIPATHWAYS	Electron Transport Chain (OXPHOS system in mitochondria)	SLC25A4, SLC25A5, SLC25A6, ATP5F1A, ATP5F1B, ATP5F1C, ATP5F1D, ATP5F1E, ATP5PB, ATP5MC1, ATP5MC2, ATP5MC3, ATP5ME, ATP5PF, ATP5PO, COX4I1, COX5B, COX6A1, COX6A2, COX6B1, COX6C, COX7A1, COX7A2, COX7B, COX7C, COX8A, COX11, COX15, ATP6, ATP8, COX1, COX2, COX3, CYTB, ND1, ND2, ND3, ND4, ND4L, ND5, ND6, NDUFA1, NDUFA2, NDUFA3, NDUFA4, NDUFA5, NDUFA6, NDUFA7, NDUFA8, NDUFA9, NDUFA10, NDUFAB1, NDUFB1, NDUFB2, NDUFB3, NDUFB4, NDUFB5, NDUFB6, NDUFB7, NDUFB8, NDUFB9, NDUFB10, NDUFC1, NDUFC2, NDUFS1, NDUFS2, NDUFS3, NDUFV1, NDUFS4, NDUFS5, NDUFS6, NDUFS8, NDUFV2, NDUFV3, SCO1, SDHA, SDHB, SDHC, SDHD, SURF1, UCP1, UCP2, UCP3, UQCRB, UQCRC1, UQCRC2, UQCRFS1, UQCRH, SLC25A14, COX7A2L, COX5A, SLC25A27, ATP5MF, COX17, ATP5PD, ATP5MG, UQCR11, UQCRQ, DMAC2L, UQCR10, NDUFA12, ATP5IF1, NDUFS7, MIR3607, MIR4691, MIR7113
WIKIPATHWAYS	Endochondral Ossification	ACAN, AKT1, ALPL, NKX3-2, BMP6, BMP7, BMPR1A, CALM1, CALM2, RUNX2, RUNX3, SERPINH1, CDKN1C, COL2A1, COL10A1, CST5, CTSV, FGF2, FGFR1, FGFR3, FRZB, GH1, GHR, GLI3, HMGCS1, IGF1, IGF1R, IGF2, IHH, MEF2C, MGP, MMP9, MMP13, DDR2, ENPP1, PLAT, PLAU, PRKACA, PTCH1, PTH, PTHLH, PTH1R, SOX5, SOX9, SPP1, STAT1, STAT5B, TGFB1, TGFB2, THRA, TIMP3, VEGFA, IFT88, FGF18, ADAMTS4, ADAMTS1, HDAC4, ADAMTS5, KIF3A, CHST11, CAB39, SLC38A2, SOX6, SCIN, MIR6073

Predefined gene sets in Hepamine

Data source	Pathways	Symbols
WIKIPATHWAYS	Endoderm Differentiation	ACACA, APC, APP, BMP7, BMPR1A, C1QBP, FOXN3, CTBP2, CTNNB1, DAB2, DNMT3B, DUSP2, DUSP4, DUSP5, ELAVL1, ELK4, EXT1, EZH2, BPTF, FOXO1, GATA4, GATA6, GLI2, NR3C1, HHEX, FOXA1, FOXA2, ONECUT1, HOXA1, HOXC11, HPRT1, JARID2, LAMC1, LHX1, SMAD2, SMAD3, SMAD4, NME1, NODAL, NOTCH1, OTX2, PAX3, PAX9, PBX1, PBX3, POU5F1, MAP2K3, PTHLH, RARG, RGS10, SFRP1, SIAH2, SOX2, SP4, STAT1, TAF4B, TAF5, TCF4, HNF1B, TCF7, TGFB1, NKX2-1, WNT3, WNT8A, ZIC3, ZBTB17, EOMES, CUL4B, PIAS1, FOXH1, BTAF1, NOG, CER1, CDYL, GDF3, CTR9, KDM4A, TOX, LRPPRC, CEBPZ, VAV3, MAD2L2, ASCC3, SOX21, WDHD1, CEP250, MTF2, DKK1, PLCH1, RTF1, DIP2A, WWC1, CRTC1, ZNF281, DDAH1, RAB38, ELP4, PABPC1, SESN1, AHDC1, TOX3, SCHIP1, TBX21, LEF1, UBR5, SFMBT1, NLK, PAF1, MBTD1, NCAPG2, TRERF1, CAND1, EMSY, ATP8B2, EPB41L5, TNRC6C, ZNF462, BCORL1, PRDM14, SOX17, RFX7, NABP2, CDC73, PARP8, ZFHX4, NANOG, GRHL2, NAA15, TET1, TCF7L1, SOX7, MIXL1, PHF6, TRIM5, ZIC5, WDFY2, AEBP2, LEO1, TRIM71, TCEAL2, SLC2A12, MIR141, MIR373, MIR375, LOC101929777, MIR7705
WIKIPATHWAYS	Endometrial cancer	AKT1, AKT2, APC, ARAF, BAD, BAK1, BAX, CCND1, BRAF, CASP9, CDH1, CDKN1A, CTNNA1, CTNNA2, CTNNB1, DDB2, GADD45A, EGF, EGFR, ELK1, ERBB2, FGF1, FGF2, FGFR1, FGFR3, FGFR2, FOXO3, FOS, GRB2, GSK3B, HRAS, ILK, KRAS, MYC, GADD45B, NRAS, PDPK1, PIK3CA, PIK3CB, PIK3CD, PIK3R1, PIK3R2, MAPK1, MAPK3, MAP2K1, MAP2K2, PTEN, RAF1, SOS1, SOS2, TCF7, TCF7L2, TP53, AXIN1, AXIN2, PIK3R3, AKT3, APC2, GADD45G, CTNNA3, LEF1, POLK, TCF7L1

Predefined gene sets in Hepamine

Data source	Pathways	Symbols
WIKIPATHWAYS	Endothelin Pathways	ADRA1A, ADRB1, ATP2A2, CAD, CALCA, CALM1, CALM2, CNN1, ECE1, EDN1, EDNRA, EDNRB, GNA15, GNAI1, GNAS, GUCY1B2, COX2, MYL1, MYLK, NOS3, NPY, NPY1R, PRKCA, MAPK1, MAP2K1, PTGIR, RAF1, CALCRL, RAMP1, GNB5, PLCB1, GNG13, ADCY10, RIIAD1
WIKIPATHWAYS	Energy Metabolism	CAMK4, CAMK2G, CREB1, ATF2, MAPK14, EP300, ESRRA, FOXO1, FOXO3, GABPA, GSK3B, HDAC1, PRMT1, MEF2A, MEF2C, MEF2D, NRF1, PPARA, PPARD, PPARG, MED1, PPP3CA, PPP3CB, PPP3CC, PPP3R1, PPP3R2, PRKAA1, PRKAA2, PRKAB1, PRKAB2, PRKAG1, RXRA, TFAM, UCP2, UCP3, NCOA1, MYBBP1A, PPARGC1A, PPRC1, SIRT3, SIRT1, TFB1M, PRKAG2, PRKAG3, TFB2M, PPARGC1B, MEF2B, MIR1281

Predefined gene sets in Hepamine

Data source	Pathways	Symbols
WIKIPATHWAYS	Epithelial to mesenchymal transition in colorectal cancer	JAG1, AKT1, AKT2, CDH1, CDH2, COL4A1, COL4A2, COL4A3, COL4A4, COL4A5, COL4A6, CLDN4, CLDN3, CLDN7, MAPK14, CTNNB1, DSP, EZH2, FOXC2, FOXM1, FN1, FZD2, GRB2, GSK3B, HIF1A, HRAS, ID1, ID2, RBPJ, ITGA5, JAG2, JUP, KRAS, LRP6, LRP5, SMAD2, SMAD3, SMAD4, MEF2D, MMP2, MMP9, MMP15, NOTCH1, NOTCH2, NOTCH3, NOTCH4, CLDN11, PAK1, PIK3CA, PIK3CB, PIK3CD, PIK3R1, PIK3R2, PKD1, PKP1, PKP2, MAPK1, MAPK3, MAPK8, MAPK11, MAPK13, MAP2K1, MAP2K2, MAP2K3, MAP2K6, PROX1, RAF1, RBBP4, MAPK12, MAP2K4, SHC1, SNAI2, SNAI1, SOS1, SOS2, SPARC, ZEB1, TGFB1, TGFB2, TGFB3, TGFBR1, TGFBR2, TJP1, CLDN5, TP53, NR2C2, TRAF6, TWIST1, VTN, WNT1, WNT2, WNT3, WNT5A, WNT6, WNT7A, WNT7B, WNT8A, WNT8B, WNT10B, WNT11, WNT2B, WNT9A, WNT9B, FZD5, FZD3, TUSC3, FZD1, FZD4, FZD6, FZD7, FZD8, FZD9, PIK3R3, EED, DLK1, NRP2, CDKL2, CLDN12, CLDN10, CLDN8, CLDN6, CLDN2, CLDN1, CLDN9, GDF15, ZEB2, AKT3, DLL3, CLDN16, STRAP, FZD10, SUZ12, CLDN14, CLDN15, CLDN17, LATS2, DLL1, CLDN20, CLDN18, WNT16, CLDN22, WNT4, DLL4, EIF5A2, TMPRSS4, CTDSP1, MPP5, NUBPL, WNT10A, WNT5B, WNT3A, CRB3, FOXQ1, FMNL2, TWIST2, CLDN23, CLDN19, CLDN24, OCLN, MIR4683, LOC101928583, LOC101929777, MIR6511B1, MIR6755
WIKIPATHWAYS	EPO Receptor Signaling	AKT1, CISH, EPO, EPOR, GRB2, IRS1, JAK2, PDK1, PIK3CG, MAPK1, MAPK3, MAP2K1, MAP2K2, PTPRC, RAF1, RASA1, SHC1, SOS1, SRC, STAT1, STAT3, STAT5A, STAT5B, SOCS1, IRS2, PTPRU

Predefined gene sets in Hepamine

Data source	Pathways	Symbols
WIKIPATHWAYS	ErbB Signaling Pathway	ABL1, ARAF, AREG, BAD, CCND1, BTC, CAMK2A, CDKN1A, CDKN1B, CRK, HBEGF, EGF, EGFR, EIF4EBP1, ELK1, ERBB2, ERBB3, ERBB4, EREG, FOXO1, MTOR, GAB1, GRB2, GSK3B, NRG1, HRAS, JUN, KRAS, MDM2, MYC, NCK1, PDK1, PLCG1, PRKCA, MAPK1, MAPK8, MAP2K1, MAP2K7, PTK2, RPS6KB1, SOS1, SRC, STAT5A, TGFA, TP53, NRG2, AKT3, BCL2L11, PAK4, NRG3, PIK3R5, CBLC, SHC2, NRG4
WIKIPATHWAYS	ERK Pathway in Huntington's Disease	BDNF, CASP3, CASP7, CREB1, EGF, EGFR, ELK1, GRM1, HRAS, NTRK2, MAPK1, MAP2K7, RAF1, RPS6KA5
WIKIPATHWAYS	ESC Pluripotency Pathways	ACVR1, AKT1, AKT2, APC, ARAF, BMP4, BMPR1A, BMPR1B, BMPR2, BRAF, CTNNB1, DVL1, DVL2, DVL3, EGF, EGFR, ELK1, FGF1, FGF2, FGF3, FGF4, FGF5, FGF6, FGF7, FGF8, FGF9, FGF10, FGF11, FGF12, FGF13, FGF14, FGFR1, FGFR3, FGFR2, FGFR4, FOS, MTOR, FZD2, GAB1, GRB2, GSK3B, HRAS, ERAS, IL6ST, JAK1, JUN, LIF, LIFR, LRP6, LRP5, SMAD1, SMAD4, SMAD5, SMAD6, SMAD7, SMAD9, MDM2, PDGFA, PDGFB, PDGFRA, PDGFRB, PIK3CD, PIK3R2, MAPK1, MAPK4, MAPK6, MAPK7, MAP2K1, MAP2K2, MAP2K3, MAP2K5, MAP2K6, PTEN, PTPN11, RAF1, MAPK12, SELENOP, SOS1, STAT3, HNF1A, WNT1, WNT2, WNT3, WNT5A, WNT6, WNT7A, WNT7B, WNT10B, WNT11, WNT2B, WNT9B, FZD5, FZD3, FGF23, AXIN1, FZD1, FZD4, FZD6, FZD7, FZD8, FZD9, FGF18, FGF17, FGF16, NOG, FGF19, AKT3, ACTR2, FGF20, FGF21, FGF22, WNT16, WNT4, WNT10A, WNT5B, WNT3A, MIR4683, LOC101929777, MIR6808
WIKIPATHWAYS	Estrogen metabolism	STS, ARSD, ARSE, COMT, CYP1A1, CYP1A2, CYP1B1, CYP3A4, NQO1, GSTA1, GSTM1, SULT1E1, SULT1A1, UGT2B7, UGT1A6, UGT1A9, UGT1A1, UGT1A3, MIR4761
WIKIPATHWAYS	Estrogen Receptor Pathway	ACOX1, CYP1A1, CYP1A2, CYP1B1, ESR1, JUN, PCK1, PDK4, PPARA, SP1, STAT3, NR0B2, GPAM

Predefined gene sets in Hepamine

Data source	Pathways	Symbols
WIKIPATHWAYS	Estrogen signaling pathway	AKT1, BCL2, BRAF, CHUK, CREB1, MAPK14, ELK1, ESR1, FOS, GNAS, GNB1, GNGT1, GPER1, IKBKB, JUN, NFKB1, PIK3CA, PRKACA, MAPK1, MAPK9, MAP2K1, SP1, IKBKG
WIKIPATHWAYS	Ethanol effects on histone modifications	ADH1A, ADH1B, ADH1C, AHCY, ALDH1A1, ALDH2, ALDH1A3, ATF2, CYP2E1, DHFR, HDAC1, HDAC2, MAT1A, MTHFR, MTR, SLC19A1, TYMS, HAT1, HDAC3, KAT2B, ALDH1A2, HDAC9, HDAC4, HDAC6, HDAC5, EHMT2, HDAC7, ELP3, HDAC8, ACSS2, HDAC10
WIKIPATHWAYS	Ethanol metabolism resulting in production of ROS by CYP2E1	CYP2E1, MAFG, NFE2L2, PRKCQ, MAPK8, MAP2K1, MAP2K2, SP1, MAFK, MAFF
WIKIPATHWAYS	Eukaryotic Transcription Initiation	POLR3D, CCNH, CDK7, ERCC2, ERCC3, GTF2A2, GTF2B, GTF2E1, GTF2E2, GTF2F2, GTF2H1, GTF2H2, GTF2H3, GTF2H4, ILK, MNAT1, POLR2A, POLR2B, POLR2C, POLR2E, POLR2F, POLR2G, POLR2H, POLR2I, POLR2J, POLR2K, TAF5, TAF6, TAF7, TAF9, TAF12, TAF13, TBP, POLR1A, POLR1D, POLR3K, POLR3B, POLR3E, POLR1E, POLR1B, POLR3H, GTF2H2C, LOC101060521
WIKIPATHWAYS	Evolocumab Mechanism	LDLR, PCSK9, MIR6886
WIKIPATHWAYS	EV release from cardiac cells and their functional effects	ERBB4, MYB, PRL, RGS16, CXCL12, TNF, KLF2, MIR126, MIR150
WIKIPATHWAYS	Exercise-induced Circadian Regulation	ARNTL, KLF9, BTG1, CEBPB, CRY1, CRY2, EIF4G2, ETV6, GFRA1, GSTM3, GSTP1, HLA-DMA, DNAJA1, HSPA8, IDI1, MYF6, PER1, PIGF, PPP1R3C, PPP2CB, PSMA4, PURA, SUMO3, CLDN5, SUMO1, UCP3, UGP2, NCOA4, HIST1H2BN, PER2, STBD1, VAPA, QKI, CLOCK, HERPUD1, DAZAP2, NR1D2, TOB1, TUBB3, NCKAP1, SF3A3, RBPMS, CBX3, TAB2, ERC2, G0S2, AZIN1, ZFR, MIR6883
WIKIPATHWAYS	Extracellular vesicle-mediated signaling in recipient cells	AKT1, APC, CTNNB1, EGFR, ERBB2, MTOR, HGF, HRAS, KRAS, SMAD2, SMAD3, SMAD4, MET, MFGE8, NRAS, RAF1, TGFA, TGFB1, TGFB2, TGFB3, TGFBR1, TGFBR2, TGFBR3, TSPAN8, WNT5A, AXIN1, PROM1, PIK3R5, DKK4, WNT3A

Predefined gene sets in Hepamine

Data source	Pathways	Symbols
WIKIPATHWAYS	Extracellular vesicles in the crosstalk of cardiac cells	BIRC5, CD63, CD81, EGF, EGFR, ETS2, GATA4, HSPB1, IGF1, IL6, KDR, MMP9, PTEN, SOD1, SPP1, STAT3, TLR4, SORBS2, PDLIM5, MIR17, MIR18A, MIR19A, MIR19B1, MIR20A, MIR320A, MIR92A1, MIR17HG, MIR455
WIKIPATHWAYS	Factors and pathways affecting insulin-like growth factor (IGF1)-Akt signaling	ACVR2B, AKT1, EIF4E, MTOR, MSTN, GSK3B, IGF1, IGF1R, IGFBP5, ILK, IRS1, ITGB1, SMAD2, SMAD3, NEB, NFKB1, PDK1, PIK3CG, PLD1, PRKAB1, PTEN, RPS6KB1, TNFRSF1A, TNFSF9, EIF2B2, WASL, PPARGC1A, JKAMP, MAP1LC3A, TRIM63, FBXO32
WIKIPATHWAYS	Farnesoid X Receptor Pathway	BAAT, CYP3A4, CYP7A1, CYP8B1, FKBP5, ABCB4, RXRA, SLC10A1, SULT2A1, UGT2B4, NR0B2, ABCB11, IRS2, FGF19, NR1H4, PPARGC1A, SLC27A5, SLCO2B1, IP6K3
WIKIPATHWAYS	Fas Ligand (FasL) pathway and Stress induction of Heat Shock Proteins (HSP) regulation	ACTA1, ACTB, ACTG1, PARP1, APAF1, FAS, FASLG, ARHGDIB, BCL2, CASP3, CASP6, CASP7, CASP8, CASP9, CASP10, DAXX, DFFA, DFFB, HSPB1, IL1A, JUN, LMNA, LMNB1, MAP3K1, NFX1, PAK1, PAK2, PRKDC, MAPK8, RB1, MAP2K4, SPTAN1, MAP3K7, TNF, MAPKAPK3, RIPK2, FADD, CFLAR, MAPKAPK2, FAF1, CYCS, LMNB2, MIR34C, MIR7108
WIKIPATHWAYS	Fatty Acid Beta Oxidation	ACADL, ACADM, ACADS, ACADVL, ACAT1, SLC25A20, CHKB, CPT1A, CPT1B, CPT2, CRAT, ECI1, DECR1, DLD, ECHS1, ACSL1, ACSL3, ACSL4, GCDH, GK, GK2, GPD2, HADHA, HADHB, HADH, LIPC, LIPE, LPL, TPI1, LIPF, ACSL6, ACSL5, ACSS2, PNPLA2
WIKIPATHWAYS	Fatty Acid Biosynthesis	ACACA, ACACB, ACLY, DECR1, ECH1, ECHS1, ACSL1, ACSL3, ACSL4, FASN, HADH, PC, SCD, ACAA2, ACSL6, MECR, ACSL5, ECHDC2, PECR, ECHDC1, ACSS2, ECHDC3
WIKIPATHWAYS	Fatty Acid Omega Oxidation	ADH1A, ADH1B, ADH1C, ADH4, ADH6, ADH7, ALDH1A1, ALDH2, CYP1A1, CYP1A2, CYP2A6, CYP2D6, CYP2E1, CYP3A4, CYP4A11
WIKIPATHWAYS	Felbamate Metabolism	CYP2E1, CYP3A4

Predefined gene sets in Hepamine

Data source	Pathways	Symbols
WIKIPATHWAYS	Ferroptosis	ALOX15, CP, CYBB, ACSL1, ACSL3, ACSL4, FTH1, FTL, GCLC, GCLM, GPX4, GSS, HMOX1, SLC11A2, PCBP1, PCBP2, PRNP, SAT1, SLC3A2, TF, TFRC, TP53, VDAC2, VDAC3, NCOA4, ATG5, LPCAT3, ATG7, ACSL6, SLC39A14, SLC7A11, SLC40A1, ACSL5, STEAP3, SLC39A8, MAP1LC3B, MAP1LC3A, FTMT, SAT2, MAP1LC3C
WIKIPATHWAYS	Fibrin Complement Receptor 3 Signaling Pathway	AKT1, RHOA, CBLB, CD14, CHUK, CXCL3, IFNB1, IKBKB, IL6, IL12B, CXCL10, IRAK1, IRAK2, IRF3, ITGAM, ITGB2, LBP, MYD88, NFKB1, NOS2, PLAT, PLG, CCL2, SRC, SYK, TLR3, TLR4, TNF, TRAF6, TYROBP, IKBKG, LY96, IRAK4, RASSF5, TIRAP, TICAM1, TICAM2, MIR718
WIKIPATHWAYS	Fluoropyrimidine Activity	CDA, CES1, CYP2A6, DHFR, DPYD, DPYS, TYMP, SLC29A1, ERCC2, FPGS, MTHFR, PPAT, RRM1, RRM2, TDG, TK1, TP53, TYMS, UCK2, UMPS, UPP1, XRCC3, ABCC3, CES2, GGH, ABCG2, ABCC5, ABCC4, SLC22A7, SMUG1, UPB1, UCK1, UPP2, MIR3658

Predefined gene sets in Hepamine

Data source	Pathways	Symbols
WIKIPATHWAYS	Focal Adhesion	ACTB, ACTG1, ACTN4, ACTN1, AKT1, AKT2, BIRC2, BIRC3, XIAP, RHOA, ARHGAP5, BAD, CCND1, BCL2, BLK, BRAF, CAPN2, CAV1, CAV2, CAV3, CCND2, CCND3, CDC42, CHAD, COL1A1, COL1A2, COL2A1, COL4A1, COL4A2, COL4A4, COL4A6, COL5A2, COL6A2, COMP, CRK, CRKL, CTNNB1, DIAPH1, DOCK1, EGF, EGFR, ELK1, ERBB2, FGR, VEGFD, FLNA, FLNB, FLNC, FLT1, FN1, FYN, GRB2, RAPGEF1, ARHGAP35, GSK3B, HCK, HGF, HRAS, TNC, IBSP, IGF1, IGF1R, ILK, ITGA6, ITGA1, ITGA2, ITGA2B, ITGA3, ITGA4, ITGA5, ITGA7, ITGA9, ITGAV, ITGB1, ITGB3, ITGB4, ITGB5, ITGB6, ITGB7, ITGB8, JUN, KDR, LAMA2, LAMA3, LAMA4, LAMA5, LAMB1, LAMB2, LAMB3, LAMC1, LAMC2, MET, MYL2, MYL5, MYLK, PPP1R12A, PPP1R12B, PAK1, PAK2, PAK3, PDGFA, PDGFB, PDGFRA, PDGFRB, PDPK1, PGF, PIK3CA, PIK3CB, PIK3CD, PIK3R1, PIK3R2, PPP1CA, PPP1CB, PPP1CC, PRKCA, PRKCB, PRKCG, MAPK1, MAPK3, MAPK8, MAPK9, MAPK10, MAP2K1, RELN, PTEN, PTK2, PTK6, PXN, RAC1, RAC2, RAC3, RAF1, RAP1A, RAP1B, RASGRF1, ROCK1, SHC1, SOS1, SPP1, SRC, SRMS, THBS1, THBS2, THBS3, THBS4, TLN1, TNR, TNXB, TXK, VASP, VAV1, VAV2, VCL, VEGFA, VEGFB, VEGFC, VTN, VWF, ZYX, PIK3R3, ITGA10, ITGA8, TNK1, ROCK2, BCAR1, AKT3, TNK2, PAK4, LAMC3, MYL9, TESK2, VAV3, MYL12A, ITGA11, PIP5K1C, SHC2, PARVB, MYLPF, COL5A3, SHC3, PPP1R12C, STYK1, PARVA, PDGFC, PAK6, MYL7, TNN, PARVG, PDGFD, TLN2, MYLK2, MYLK3, MYL10, MYL12B, LAMA1, MYLK4, SHC4, MIR4260, LINC02478, MIR4758, MIR6852, BUB1B-PAK6

Data source	Pathways	Symbols
WIKIPATHWAYS	Focal Adhesion-PI3K-Akt-mTOR-signaling pathway	ACACA, AKT1, AKT2, ANGPT1, ANGPT2, ATF4, BAD, CASP9, CDKN1A, CDKN1B, CHAD, CHRM1, CHRM2, COL1A1, COL1A2, COL2A1, COL3A1, COL4A1, COL4A2, COL4A4, COL4A6, COL5A1, COL5A2, COL6A2, COL11A1, COL11A2, COMP, CREB1, ATF2, ATF6B, CSF1, CSF1R, CSF3, CSF3R, CSH1, CSHL1, LPAR1, EFNA1, EFNA2, EFNA3, EFNA4, EFNA5, EGF, EGFR, EPHA2, EIF4B, EIF4E, EIF4EBP1, ELAVL1, EPAS1, EPO, EPOR, F2R, FGF1, FGF2, FGF3, FGF4, FGF6, FGF7, FGF8, FGF9, FGF10, FGF11, FGF12, FGF13, FGF14, FGFR1, FGFR3, FGFR2, FGFR4, VEGFD, FOXO1, FOXO3, FLT1, FLT4, FN1, MTOR, GHR, GNB1, GNB2, GNB3, GNG3, GNG4, GNG5, GNG7, GNG10, GNG11, GNGT1, GNGT2, LPAR4, GRB2, GSK3B, GYS1, GYS2, HGF, HIF1A, FOXA1, HRAS, HSP90AA1, HSP90AB1, TNC, IBSP, IFNA7, IFNAR1, IFNAR2, IFNB1, IGF1, IGF1R, IKBKB, IL2, IL2RA, IL2RB, IL2RG, IL3RA, IL4R, IL6R, IL7R, INS, INSR, ITGA6, IRS1, ITGA2, ITGA2B, ITGA3, ITGA4, ITGA5, ITGA7, ITGA9, ITGAD, ITGAE, ITGAL, ITGAV, ITGAX, ITGB1, ITGB2, ITGB3, ITGB4, ITGB5, ITGB6, ITGB7, ITGB8, JAK1, JAK2, JAK3, KDR, KIT, KRAS, LAMA2, LAMA3, LAMA4, LAMA5, LAMB1, LAMB2, LAMB3, LAMC1, LAMC2, LIPE, MDM2, RAB8A, MET, KITLG, NGF, NGFR, NOS1, NOS2, NOS3, NRAS, OSM, PDGFA, PDGFB, PDGFRA, PDGFRB, PDPK1, PFKFB1, PFKFB2, PFKFB3, PFKFB4, PGF, PIK3C2A, PIK3C2B, PIK3CA, PIK3CB, PIK3CD, PIK3CG, PIK3R1, PIK3R2, PPP2CA, PPP2CB, PPP2R1A, PPP2R1B, PPP2R2B, PPP2R2C, PPP2R3A, PPP2R5A, PPP2R5B, PPP2R5C, PPP2R5D, PPP2R5E, PRKAA1, PRKAA2, MAPK1, MAPK3, MAP2K1, MAP2K2, PRL, PRLR, RELN, PTEN, PTK2, RAB2A, RAF1, RHEB, RPS6, RPS6KB1, RPS6KB2, SLC2A1, SLC2A2, SLC2A3, SLC2A4, SOS1, SPP1, SREBF1, STK11, TEK, THBS1, THBS2, THBS3, THBS4, TNR, TNXB, HSP90B1, TSC1, TSC2, VEGFA, VEGFB, VEGFC, VTN, VWF, TCL1A, ULK1, IRS4, ITGA10, ITGA8, IKBKG, IRS2, FGF18, FGF17, FGF16, LPAR2, OSMR, RAB11B, EIF4E2, CREB5, TCL1B, FGF19, AKT3, LPAR6, LAMC3, CREB3, RAB10, PPARGC1A, CDC37, ITGA11, PHLPP2, TBC1D1, PHLPP1, PIK3R5, LPAR3, FGF20, FGF21, FGF22, PIK3R4, COL5A3, ANGPT4, RAB14, CAB39, GNG13, PELO, GNG2, DDIT4

Predefined gene sets in Hepamine

Data source	Pathways	Symbols
WIKIPATHWAYS	Folate-Alcohol and Cancer Pathway Hypotheses	ADH5, ALDH1A1, CBS, CEBPB, CREB1, CYP2E1, MTHFR, MTR, ALDH1L1
WIKIPATHWAYS	Folate Metabolism	SERPINA3, ABCA1, AHCY, ALB, APOA1, APOB, CAT, CBS, SCARB1, CRP, CSF1, CTH, DHFR, F2, F7, FOLR1, FOLR2, FOLR3, GART, GPX1, GPX2, GPX3, GPX4, HBA1, HBB, ICAM1, IFNG, IL1B, IL2, IL4, IL6, INS, INSR, LDLR, MAT1A, MPO, MTHFD1, MTHFR, MTR, MTRR, NFKB1, NFKB2, NOS1, SERPINE1, PLAT, PLG, RELA, SAA1, SAA2, SAA3P, SAA4, CCL2, SHMT1, SHMT2, SLC19A1, SOD1, SOD2, SOD3, TNF, TP53, MTHFS, MTHFD2, RFK, FLAD1, SLC46A1, GPX6, IZUMO1R, SOD2-OT1, MIR6886, MIR6778
WIKIPATHWAYS	Follicle Stimulating Hormone (FSH) signaling pathway	AKT1, AREG, BDNF, CGA, CREB1, MAPK14, CYP19A1, EIF4EBP1, FOXO1, MTOR, FSHB, FSHR, GRK6, PRKACA, PRKCA, MAPK1, MAPK3, RAF1, RHEB, RPS6, RPS6KB1, RPS6KB2, SGK1, SRC, TSC2, HIST3H3, APPL1
WIKIPATHWAYS	FTO Obesity Variant Mechanism	TBX1, UCP1, IRX5, PPARGC1A, PRDM16, FTO, IRX3, ARID5B
WIKIPATHWAYS	G13 Signaling Pathway	RHOA, ARHGDIB, ARHGDIG, CALM1, CALM2, CDC42, CFL1, CFL2, DIAPH1, LIMK1, MAP3K4, MYBPH, MYL1, PAK3, PFN1, PIK3CA, PIK3CB, PIK3CD, PIK3R2, PIP4K2A, PPP1CB, PKN1, MAPK10, RAC1, ROCK1, RPS6KB1, RTKN, WAS, IQGAP1, WASL, ARHGEF1, ROCK2, TNK2, GNA13, IQGAP2, CIT, CYFIP1, SH3RF1, RHPN2, MIR1178, LOC100505585
WIKIPATHWAYS	G1 to S cell cycle control	ATM, CCND1, CCNB1, CCND2, CCND3, CCNE1, CCNG2, CCNH, CDK1, CDC25A, CDK2, CDK4, CDK6, CDK7, CDKN1A, CDKN1B, CDKN1C, CDKN2A, CDKN2B, CDKN2C, CDKN2D, CREB1, ATF6B, GADD45A, E2F1, E2F2, E2F3, MCM2, MCM3, MCM4, MCM5, MCM6, MCM7, MDM2, MNAT1, MYC, MYT1, ORC1, ORC2, ORC4, ORC5, PCNA, POLE, POLE2, PRIM1, PRIM2, RB1, RPA1, RPA2, RPA3, TFDP1, TFDP2, TP53, WEE1, CDC45, CCNA1, CCNE2, CREB3, ORC6, ORC3, POLA2, CREB3L3, CREB3L1, CREB3L4

Predefined gene sets in Hepamine

Data source	Pathways	Symbols
WIKIPATHWAYS	GABA receptor Signaling	ABAT, AP2A1, AP2A2, AP2B1, ALDH9A1, AP2M1, AP2S1, GABBR1, GABRA1, GABRA2, GABRA3, GABRA4, GABRA5, GABRA6, GABRB1, GABRB2, GABRB3, GABRD, GABRE, GABRG1, GABRG2, GABRG3, GABRP, GAD1, GAD2, SLC6A1, SLC6A11, GABBR2, GPHN, GABRQ, SLC32A1, MIR224, MIR452
WIKIPATHWAYS	Ganglio Sphingolipid Metabolism	FUT1, B4GALNT1, ST3GAL1, ST3GAL2, ST8SIA1, B3GALT4, B3GALT1, ST3GAL5, SLC33A1, ST8SIA5, ST6GALNAC6, ST8SIA3, A3GALT2
WIKIPATHWAYS	Gastric acid production	CCK, GAST, CBLIF, GRP, MUC6, SCT, VIP
WIKIPATHWAYS	Gastric Cancer Network 1	ACTL6A, APC, CENPF, ECT2, H3F3A, MCM4, MYBL2, NOTCH1, S100P, AURKA, TOP2A, GATD3A, RUVBL1, CCNA1, KIF20B, UBE2C, ESM1, TPX2, RNF216, INO80D, CEP192, KIF15, NUP107, SMOC2, HIST4H4, E2F7, LIN9, CASTOR3, GATD3B
WIKIPATHWAYS	Gastric Cancer Network 2	CD48, COL9A1, COL9A3, CTNNB1, EGFR, LBR, MYC, RAD17, RFC3, RFC4, S100A6, TOP2A, TP53, SNURF, CEBPZ, UBE2C, AHCTF1, CACYBP, ATAD2, UBE2T, PLAC8, CHTF8, FANCI, BRIX1, OTUD5, CHTF18, DSCC1, LMNB2, MTDH, FAM91A1, RNF144B, MIRLET7E, MIR7108
WIKIPATHWAYS	Gene regulatory network modelling somitogenesis	EPHA4, FGF8, HES1, LFNG, NOTCH1, TBX6, DLL1, HES7, WNT3A, RIPPLY2, MESP2
WIKIPATHWAYS	Genes targeted by miRNAs in adipocytes	HCN2, ERG, GJA1, IGF1, KCNE1, KCNJ2, KCNQ1, SRF, TMSB4X, HAND2, HDAC4, HCN4, PTBP2, MIR1-1, MIR1-2, MIR133A1, MIR133A2, MIR133B, MIR133A1HG

Predefined gene sets in Hepamine

Data source	Pathways	Symbols
WIKIPATHWAYS	Genotoxicity pathway	ACTA2, TNFRSF17, CBLB, CDKN1A, CEBPD, CENPE, PLK3, DDB2, GADD45A, HIST1H1E, HIST1H2BB, ID2, ITPR1, SMAD5, MDM2, PCDH8, PRKAB1, PRKAB2, PTGER4, RPS27, SEL1L, SEMG2, BTG2, COIL, HIST1H2BG, HIST1H2BN, HIST1H2BM, HIST1H2BI, HIST1H2BC, HIST1H3D, PPM1D, LRRFIP2, EI24, TP53I3, RAPGEF2, CCP110, TOPORS, TRIM22, B3GNT2, DUSP14, DAAM1, PHLDA3, FBXO22, SERTAD1, RRM2B, TRIAP1, TM7SF3, TIGAR, AEN, NLRX1, E2F8, ITPKC, MEX3B, BRMS1L, ARRDC4, IKBIP, AMER1, E2F7, DCP1B, BLOC1S2, GXYLT1, LCE1E, RBM12B
WIKIPATHWAYS	Glial Cell Differentiation	CNP, GAP43, MAG, MBP, MSN, PLP1, TPPP, MIR206
WIKIPATHWAYS	Globo Sphingolipid Metabolism	ABO, FUT1, FUT2, GALNT2, GCNT1, ST6GAL1, ST3GAL1, ST3GAL2, ST8SIA1, B3GALNT1, B3GALT5, ST6GALNAC2, FUT9, GBGT1, ST6GALNAC4, ST6GALNAC6, A4GALT, ST6GALNAC1, ST6GALNAC5, ST6GAL2, A3GALT2, ST6GALNAC3
WIKIPATHWAYS	Glucocorticoid and Mineralcorticoid Metabolism	CYP11A1, CYP11B2, CYP17A1, CYP21A2, HSD3B1, HSD3B2, HSD11B1, HSD11B2
WIKIPATHWAYS	Glucose Homeostasis	INS
WIKIPATHWAYS	Glucuronidation	HK1, PGM1, PGM3, PGM5, UGDH, UGP2, UGT2B4, UGT2B7, UGT2B10, UGT2B15, UGT2B17, UGT2B11, UGT2A1, UGT2B28, UGT1A10, UGT1A8, UGT1A7, UGT1A6, UGT1A5, UGT1A9, UGT1A4, UGT1A1, UGT1A3, PGM2, UGT2A3, UGT2A2
WIKIPATHWAYS	Glutathione metabolism	ANPEP, G6PD, GGT1, GGT5, GCLC, GCLM, GPX1, GPX2, GPX3, GPX4, GSR, GSS, GSTA1, GSTM1, GSTM2, GSTT2, IDH1, OPLAH, GGTLC2, GGTLC1, GSTA5, GGT2, LOC102724197
WIKIPATHWAYS	Glycerophospholipid Biosynthetic Pathway	CHKB, GK, GPD1, PLA2G2A, PCYT2, PIP5K1A, GNPAT, AGPS, CDS2, PGS1, LPIN2, PTDSS1, CEPT1, PEMT, CDIPT, LPIN1, CRLS1, PI4K2A, CHPT1, PTPMT1, MOGAT3

Predefined gene sets in Hepamine

Data source	Pathways	Symbols
WIKIPATHWAYS	Glycogen Metabolism	AGL, CALM1, CALM2, CALM3, GBE1, GSK3A, GSK3B, GYG1, GYS1, GYS2, HK1, HK2, HK3, PGM1, PHKA1, PHKA2, PHKB, PHKG1, PHKG2, PPP2CA, PPP2CB, PPP2R1A, PPP2R1B, PPP2R2A, PPP2R2B, PPP2R2C, PPP2R3A, PTPA, PPP2R5A, PPP2R5B, PPP2R5C, PPP2R5D, PPP2R5E, PYGB, PYGL, PYGM, UGP2, GYG2, PPP2R3B, HKDC1
WIKIPATHWAYS	Glycolysis and Gluconeogenesis	ALDOA, ALDOB, ALDOC, DLAT, DLD, ENO1, ENO2, ENO3, FBP1, G6PC, GAPDH, GCK, GOT1, GOT2, GPI, HK1, HK2, HK3, LDHA, LDHB, LDHC, MDH1, MDH2, PC, PCK1, PDHA1, PFKL, PFKM, PFKP, PGAM1, PGAM2, PGK1, PGK2, PKLR, PKM, SLC2A1, SLC2A2, SLC2A3, SLC2A4, SLC2A5, TPI1, FBP2, MPC2, MPC1, LDHAL6B

Data source	Pathways	Symbols
WIKIPATHWAYS	GPCRs, Class A Rhodopsin-like	ADORA1, ADORA2A, ADORA2B, ADORA3, ADRA1D, ADRA1B, ADRA1A, ADRA2A, ADRA2B, ADRA2C, ADRB1, ADRB2, ADRB3, AGTR1, AGTR2, APLNR, AVPR1A, AVPR1B, AVPR2, OPN1SW, BDKRB1, BDKRB2, CXCR5, BRS3, C3AR1, CCKAR, CCKBR, CHRM1, CHRM2, CHRM3, CHRM4, CHRM5, CCR1, CCR3, CCR4, CCR5, CCR6, CCR7, CCR8, ACKR2, CMKLR1, LTB4R, CNR1, CNR2, CX3CR1, DRD1, DRD2, DRD3, DRD4, DRD5, EDNRA, EDNRB, F2R, F2RL1, F2RL2, FPR1, FPR2, FPR3, FSHR, GALR1, OPN1MW, GHSR, GPR1, CCR10, GPR3, GPR4, XCR1, GPR6, NPBWR1, NPBWR2, CXCR3, PRLHR, GPR12, GPR15, GPR17, GPR18, GPR19, GPR20, GPR21, GPR22, LPAR4, MCHR1, GPR25, GPR27, GPER1, GPR31, GPR32, GPR34, GPR35, GPR37, MLNR, GPR39, FFAR1, FFAR3, FFAR2, GRPR, HCRTR1, HCRTR2, HRH1, HRH2, HTR1A, HTR1B, HTR1D, HTR1E, HTR1F, HTR2A, HTR2B, HTR2C, HTR4, HTR5A, HTR6, HTR7, CXCR1, CXCR2, LHCGR, MAS1, MC1R, MC2R, MC3R, MC4R, MC5R, MTNR1A, MTNR1B, NMBR, NPY1R, NPY2R, NPY6R, NPY5R, NTSR1, OPRD1, OPRK1, OPRL1, OPRM1, OR1D2, OR1F1, OR2C1, OR3A1, OR3A2, OXTR, P2RY1, P2RY2, P2RY4, P2RY6, P2RY11, NPY4R, PTAFR, PTGDR, PTGER1, PTGER2, PTGER3, PTGER4, PTGFR, PTGIR, OPN1LW, RHO, SSTR1, SSTR2, SSTR3, SSTR4, SSTR5, TBXA2R, TRHR, CXCR4, OR2H2, GPR68, OR1A1, OR1D5, OR1E1, OR1E2, OR1G1, OR3A3, GPR65, GALR3, OR6A2, GALR2, HCAR3, F2RL3, CCRL2, GPR50, GPR37L1, GPR52, P2RY14, LPAR6, NMUR1, RRH, OR5I1, CYSLTR1, CCR9, NPFFR2, GPR83, GPR75, GPR45, PTGDR2, HRH3, GPR161, OPN3, NTSR2, RABGAP1, OR1C1, OR1A2, OR2F1, OR2B6, OR7A17, OR10J1, OR8B8, OR11A1, OR10H3, OR10H2, OR10H1, OR7C2, OR7A5, OR7C1, OR2W1, OR2T1, OR2J2, OR2H1, HCAR1, C5AR2, P2RY10, GPR171, ACKR4, P2RY13, GPR87, GPR173, GPR85, OR2S2, SUCNR1, NMUR2, ACKR3, CYSLTR2, LPAR5, NPFFR1, P2RY12, OR2A4, GPR63, OR5V1, OR2B2, OR12D3, GPR174, OPN4, MAS1L, OR2D2, OR1I1, OR6B1, OR2F2, OR10A5, OR2AG1, OR1Q1, RGL4, OR8D2, OR10A4, OR5F1, OR7A10, OR2B3, OR2J3, OR14J1, OR1D4, CCR2

Predefined gene sets in Hepamine

Data source	Pathways	Symbols
WIKIPATHWAYS	GPCRs, Class B Secretin-like	ADCYAP1R1, CALCR, ADGRE5, CRHR1, CRHR2, ADGRE1, GCGR, GHRHR, GIPR, GLP1R, PTH1R, PTH2R, SCTR, VIPR1, VIPR2, GLP2R, ADGRG2, CALCRL, ADGRL1, ADGRL2, ADGRL3, ADGRE2, ADGRL4, LINC02210-CRHR1
WIKIPATHWAYS	GPCRs, Class C Metabotropic glutamate, pheromone	CASR, GABBR1, GRM1, GRM2, GRM3, GRM4, GRM5, GRM6, GRM7, GRM8, GPRC5A, GABBR2, GPRC5B, GPRC5D, GPRC5C, MIR614
WIKIPATHWAYS	GPCRs, Other	ADORA2A, ADORA3, ADRA1D, ADRB2, CCKBR, CHRM2, CHRM3, CCR5, CNR1, DRD3, DRD4, GPR183, S1PR1, EDNRA, CELSR3, CELSR2, F2R, FSHR, ACKR1, GHRHR, GNRHR, CXCR3, UTS2R, GPR17, GPR18, GRM1, GRM8, GRPR, HTR1F, HTR2A, HTR7, CXCR1, CXCR2, NTSR1, GPR143, OR1F1, OR3A1, P2RY11, PTGFR, SMO, SSTR2, OR1E1, OR1G1, OR3A3, TAAR5, TAAR2, TAAR3P, ADGRG1, GPR55, CELSR1, GPR83, GPR176, ADGRL2, ADGRL3, OR2F1, OR2B6, OR2M4, OR7A17, OR8G1, OR7E24, OR2H1, C5AR2, GPR162, ALG6, GPR132, ADGRE2, RXFP3, P2RY13, GPR84, GPR88, LTB4R2, VN1R1, HRH4, RXFP1, LGR6, GPR135, OR2A4, OR5AC2, GPR61, ADGRV1, MCHR2, ADGRE3, GPR62, PROKR2, OR1N1, OR10A5, ADGRF5, OR6C3, ADGRD1, OR10A2, OR6C2, OR2A5, OR2A20P, MIR4793
WIKIPATHWAYS	GPR40 Pathway	GNA11, FFAR1, ITPR3, PKD1, PLCB2, PLCB3, PLCD1, PLCL1, PLCG1, PLCG2, PLCH1, PLCB1, PLCE1, PLCZ1, PLCD3, MIR6511B1

Predefined gene sets in Hepamine

Data source	Pathways	Symbols
WIKIPATHWAYS	G Protein Signaling Pathways	ADCY1, ADCY2, ADCY3, ADCY5, ADCY6, ADCY7, ADCY8, ADCY9, RHOA, CALM1, CALM2, GNA11, GNA12, GNA15, GNAI1, GNAI2, GNAI3, GNAL, GNAO1, GNAQ, GNAS, GNAZ, GNB1, GNB2, GNB3, GNG3, GNG4, GNG5, GNG7, GNG10, GNG11, GNGT1, GNGT2, HRAS, ITPR1, KCNJ3, KRAS, NRAS, PDE1A, PDE1C, PDE4A, PDE4B, PDE4C, PDE4D, PDE7A, PDE8A, PDE1B, PLCB3, PPP3CA, PPP3CC, PRKACA, PRKACB, PRKACG, PRKAR1A, PRKAR1B, PRKAR2A, PRKAR2B, PRKCA, PRKCB, PRKCD, PRKCE, PRKCG, PRKCH, PRKCI, PRKD1, PRKCQ, PRKCZ, RRAS, SLC9A1, AKAP1, PDE8B, AKAP4, ARHGEF1, AKAP7, AKAP6, AKAP5, AKAP12, GNA14, AKAP9, AKAP8, AKAP3, GNA13, GNB5, AKAP13, AKAP11, AKAP10, AKAP2, PRKD3, PDE7B, GNG13, GNG12, GNG8, ADCY4, MIR197, LOC729966, LOC100505585, MIR7706
WIKIPATHWAYS	Gut-Liver Indole Metabolism	CYP2E1
WIKIPATHWAYS	H19 action Rb-E2F1 signaling and CDK-Beta-catenin activity	JAG1, CCND1, CDH1, CDK4, CDK8, CSRP2, CTNNB1, E2F1, PMAIP1, MED1, RB1, SOX4, TULP3, H2AFY, H19, MIR675
WIKIPATHWAYS	Hair Follicle Development: Cytodifferentiation (Part 3 of 3)	BMP4, BMP6, BMPR1A, RUNX3, CD34, CCN2, CTNNB1, CUX1, DLX3, DSC2, DSG1, EGFR, EGR2, ELANE, FOXE1, FOS, FOSB, GAS1, GATA3, GLI2, NR3C1, HOXC12, IFNG, IGF1, IGFBP5, RBPJ, JUN, KRT15, KRT19, SMAD1, SMAD4, KITLG, CD200, MSX1, MSX2, NFATC1, NFKB1, NOTCH1, NOTCH2, KLK7, KLK6, S100A4, SFRP1, SOX2, SOX9, ADAM17, TCF4, TCF3, TGFB1, WNT5A, FZD1, FOXN1, LGR5, TP63, PROM1, LHX2, KLK4, FST, GJB6, SPINK5, WIF1, PHLDA1, DKK1, GTPBP4, CASP14, KLK5, SOSTDC1, LRIG1, KLK13, DKK4, DKK3, KLK14, LEF1, GPRC5D, HR, PERP, BCL11B, ADAMTS20, KRTAP3-3, KRTAP2-4, FOXQ1, DSG4, KRTAP15-1, GSDMA, KRTAP8-1, KRTAP6-1, SPINK6, MIR6510

Predefined gene sets in Hepamine

Data source	Pathways	Symbols
WIKIPATHWAYS	Heart Development	BMP2, BMP4, BMPR1A, BMPR2, NKX2-5, CTNNB1, ERBB3, FGF8, FGF10, FOXC1, FOXC2, GATA4, GATA6, FOXA2, ISL1, SMAD1, SMAD4, MEF2C, NFATC1, NFATC2, NFATC3, NFATC4, NOTCH1, PITX2, MAPK1, PTPN11, SHH, SRF, TBX1, TBX2, TBX5, VEGFA, VEGFB, VEGFC, BHLHE40, FOXH1, HAND1, HAND2, HEY1, HEY2, BMP10, IRX4, TBX20, SMYD1, MIR1-1, MIR145
WIKIPATHWAYS	Hedgehog Signaling Pathway	GRK2, ARNTL, GLI1, GLI2, GLI3, IHH, PTCH1, SHH, SMO, PTCH2, SAP18, SIN3A, STK36, DHH, SUFU, KIF7, GRK2, GRK3, ARRB1, ARRB2, CCND1, BCL2, CCND2, CSNK1A1, CSNK1D, CSNK1E, CSNK1G2, CSNK1G3, EVC, GAS1, GLI1, GLI2, GLI3, IHH, LRP2, PRKACA, PRKACB, PRKACG, PTCH1, SHH, SMO, SPOP, CUL3, PTCH2, KIF3A, GPR161, DHH, CDON, SUFU, CSNK1G1, SMURF1, HHIP, SMURF2, FBXL17, BOC, CSNK1A1L, EVC2, SPOPL, KIF7, TPTEP2-CSNK1E
WIKIPATHWAYS	Hematopoietic Stem Cell Differentiation	ABO, ACVR1B, RHOH, RUNX1, CBFA2T3, CD34, CSF1, CSF2, CSF3, EPO, FLI1, FOS, FOSB, GATA1, GATA2, GP9, GYPA, NCKAP1L, IL1A, IL1B, IL3, IL5, IL6, IRF5, ITGA2B, ITGB3, KCNH2, LMO2, LYL1, KITLG, CIITA, MUC1, MXI1, MYB, NFATC2, NFE2, NOTCH1, PIM1, SPI1, STAT5A, THRB, TNXB, TPO, VAV1, CXCR4, RIOK3, IKZF1, KLF1, SEC14L2, TRIM29, LEF1, HES6, HMGN5, TRAF3IP3, ZNF835, MIR128-1, MIR130A, MIR150, MIR16-1, MIR181A1, MIR221, MIR3074, LOC100506403
WIKIPATHWAYS	Hematopoietic Stem Cell Gene Regulation by GABP alpha/beta Complex	ATM, BCL2, BCL2L1, CREBBP, DNMT1, DNMT3A, DNMT3B, EP300, ETV6, FOXO3, FLT3, GABPA, GABPB1, GZMB, SMAD4, MCL1, PTEN, SMARCA4, TERF2, ZFX, GABPB1-IT1, MIR1281
WIKIPATHWAYS	Heme Biosynthesis	ALAD, ALAS1, ALAS2, CPOX, FECH, HMBS, PPOX, UROD, UROS

Predefined gene sets in Hepamine

Data source	Pathways	Symbols
WIKIPATHWAYS	Hepatitis C and Hepatocellular Carcinoma	AKT1, BIRC3, BIRC5, FASLG, CCND1, BCL2L1, BRCA1, CASP3, CASP7, CASP9, CD44, CDKN1A, COL4A2, MAPK14, E2F2, CTTN, FRZB, GRB2, HIF1A, IL6, IL6R, CXCL8, CXCR1, JAK1, JUN, SMAD3, SMAD4, MMP1, MYC, NFKB1, NOS2, PODXL, MAPK3, MAPK8, PTGS2, PTPN11, RAC1, RRM2, SOS1, STAT3, HNF1A, TGFB1, TGFBR1, TP53, UCHL1, VAV2, VEGFA, MYOF, LEF1, C9orf3, MIR24-1
WIKIPATHWAYS	Hereditary leiomyomatosis and renal cell carcinoma pathway	ACACA, ACACB, HIF1A, LDHA, NFE2L2, SLC11A2, PDHA1, PDHA2, PDHB, PDK1, PRKAA1, PRKAB1, PRKAG1, RPS6, SLC2A1, TP53, VEGFA, CUL3, KEAP1, EGLN1
WIKIPATHWAYS	Heroin metabolism	BCHE, CES1, CES2
WIKIPATHWAYS	Hfe effect on hepcidin production	BMP6, HFE, ID1, SMAD7, HAMP, HJV, TMPRSS6
WIKIPATHWAYS	HIF1A and PPARG regulation of glycolysis	GAPDH, GPD1, HIF1A, LDHA, PPARG, SLC2A1, TPI1, GPAT3
WIKIPATHWAYS	Histone Modifications	EZH1, EZH2, H3F3A, H3F3B, KMT2A, SET, SETMAR, SUV39H1, PRDM2, KMT2D, HIST1H3A, HIST1H3D, HIST1H3C, HIST1H3E, HIST1H3I, HIST1H3G, HIST1H3J, HIST1H3H, HIST1H3B, HIST1H4A, HIST1H4D, HIST1H4F, HIST1H4K, HIST1H4J, HIST1H4C, HIST1H4H, HIST1H4B, HIST1H4E, HIST1H4L, HIST1H4G, EED, HIST1H3F, SETD1A, KMT2B, SETDB1, SMYD5, EHMT2, SETD1B, SETBP1, SETD2, KMT5B, SETD4, SETD5, ASH1L, KMT2E, SMYD2, KMT2C, NSD1, SMYD3, SUV39H2, EHMT1, SETD6, SETD7, SETDB2, SETD3, DOT1L, KMT5C, SMYD4, HIST4H4, AEBP2, HIST2H3C, SETD9, SMYD1, HIST2H3A, KMT5A, HIST2H3D, MIR555, MIR4738, MIR6755
WIKIPATHWAYS	Homologous recombination	ATM, BRCA2, MRE11, NBN, POLD1, POLD2, RAD51, RAD52, RPA1, RAD50, POLD3, RAD54B, POLD4
WIKIPATHWAYS	Hormonal control of Pubertal Growth Spurt	GNRH1, SST

Predefined gene sets in Hepamine

**Data source**	**Pathways**	**Symbols**
WIKIPATHWAYS	Human Complement System	ADM, ALB, APCS, APOA1, ARRB2, CFB, C1QBP, SERPING1, C1R, C1S, C2, C3, C3AR1, C4A, C4BPA, C5, C5AR1, C6, C7, C8A, C9, CALR, CD19, CD40, CD59, CPN1, CR1, CR2, CRP, CSNK1A1, CD55, DCN, CFD, ELANE, F10, F11, F12, F13A1, FCER2, FCGR3A, FCN1, FCN2, FGA, FGB, FGG, FKBP2, FPR1, GNA15, GNAI2, GNAI3, CFH, CFHR2, IBSP, ICAM1, ICAM2, CFI, ITGA2, ITGA2B, ITGAX, ITGB2, ITGB3, KLKB1, LAMA5, LAMB1, LAMC1, LRP2, MBL2, CD46, CFP, PLAUR, PLG, PRKACA, PRKCA, PRNP, PROS1, MASP1, PTX3, RPS19, SELE, SELL, SELP, SELPLG, SPP1, THBS1, TLR2, TXN, WAS, ADIPOQ, CLEC4M, MASP2, CFHR4, VSIG4, CD93, C5AR2, MIR197, SFTPA1, SFTPA2, MIR4758
WIKIPATHWAYS	Human Thyroid Stimulating Hormone (TSH) signaling pathway	ADCY2, ADCY3, AKT1, APEX1, BRAF, CCND3, CCNE1, CDK2, CDK4, CDKN1B, CGA, CREB1, MAPK14, E2F1, EGR1, FOS, MTOR, GNA12, GNAI1, GNAI2, GNAI3, GNAO1, GNAQ, GNAS, GNB1, HRAS, IGF1R, JAK1, JAK2, JUN, MYC, PDE4D, PDPK1, PIK3CA, PIK3R1, PIK3R2, PLD1, MAPK1, MAPK3, MAP2K1, MAP2K3, MAP2K6, RAF1, RALGDS, RAP1A, RAP1B, RAP1GAP, RB1, RBL2, RPS6, RPS6KA1, RPS6KB1, SRC, STAT1, STAT3, TSHB, TSHR, TTF1, PAX8, TTF2, GNA13, PLCB1, SCRIB, KCNIP3, GNG2, MYL12B, MIR197, MIR937
WIKIPATHWAYS	Hypertrophy Model	ADAM10, ATF3, HBEGF, EIF4E, EIF4EBP1, MSTN, IFNG, IFRD1, CCN1, IL1A, IL1R1, IL18, JUND, MYOG, ZEB1, VEGFA, NR4A3, WDR1, DUSP14, ANKRD1
WIKIPATHWAYS	Hypothesized Pathways in Pathogenesis of Cardiovascular Disease	AGTR1, ANGPT2, RUNX2, MAPK14, CCN2, ENG, FBN1, FBN2, FLNA, LTBP1, LTBP2, SMAD2, SMAD3, SMAD4, SERPINE1, MAPK1, MAPK3, MAPK8, SHC1, TGFBR1, TGFBR2, TGFBR3, NR2C2, POSTN, FBN3
WIKIPATHWAYS	Hypothetical Craniofacial Development Pathway	RHOA, IRF6, TFAP2A, TGFB3, WNT1, TP63, ARHGAP29, FGD5P1
WIKIPATHWAYS	Hypoxia-mediated EMT and Stemness	ZEB1, DICER1

Predefined gene sets in Hepamine

Data source	Pathways	Symbols
WIKIPATHWAYS	ID signaling pathway	CCNE1, CDK2, ELK1, ELK3, ELK4, ID1, ID2, ID3, MYOD1, PAX2, PAX5, RB1, RBL1, RBL2, SREBF1, PAX8
WIKIPATHWAYS	IL17 signaling pathway	AKT1, CEBPB, CEBPD, GSK3B, IKBKB, IL17A, JAK1, JAK2, NFKB1, NFKBIB, MAPK1, MAPK3, RELA, SP1, STAT3, MAP3K7, TRAF3, TRAF6, IKBKG, MAP3K14, TRAF3IP2, IL17RA, IL17C, IL17B, IL17D, IL17RD, IL17RB, IL25, IL17RC, IL17F, IL17RE
WIKIPATHWAYS	IL1 and megakaryocytes in obesity	CCR3, HBEGF, F2, F2R, FCER1A, ICAM1, IFNG, IL1B, IL1R1, IL18, IRAK1, MMP9, MYD88, NFKB1, PIK3CA, S100A9, CCL2, TIMP1, TIMP2, TLR1, TLR2, PLA2G7, SELENBP1, NLRP3, MIR718
WIKIPATHWAYS	IL-1 signaling pathway	AKT1, CHUK, ATF2, MAPK14, HSPB2, IKBKB, IL1A, IL1B, IL1R1, IL1RAP, IRAK1, IRAK2, JUN, MAP3K1, MAP3K3, MYD88, NFKB1, NFKBIA, NFKBIB, PIK3R1, PIK3R2, PLCG1, PRKCZ, MAPK1, MAPK3, MAPK8, MAPK9, MAP2K1, MAP2K2, MAP2K3, MAP2K6, MAP2K7, PTPN11, REL, RELA, CCL2, MAP2K4, MAP3K7, TRAF6, UBE2N, UBE2V1, IKBKG, SQSTM1, MAP3K14, MAPKAPK2, TAB1, MAP3K2, IRAK3, TAB2, IRAK4, ECSIT, TOLLIP, PELI2, PELI1, TAB3, MIR718, MIR6502
WIKIPATHWAYS	IL-2 Signaling Pathway	AKT1, BCL2, CBL, CCND2, CISH, CRKL, PTK2B, FOXO3, FOS, FYN, GRB2, HRAS, IL2, IL2RA, IL2RB, IL2RG, JAK1, JAK3, JUN, LCK, MAPT, MYC, PIK3R1, MAPK1, MAPK3, MAP2K1, MAP2K2, PTPN11, RAF1, RPS6, RPS6KB1, RPS6KB2, SHC1, SOS1, STAT1, STAT3, STAT5A, STAT5B, SYK, SOCS3, NMI, GAB2
WIKIPATHWAYS	IL-3 Signaling Pathway	AKT1, BAD, BCL2, BCL2L1, CBL, CD86, CD69, CCR3, CRKL, CSF2RB, FOS, FYN, GRB2, RAPGEF1, HCK, HRAS, IL3, IL3RA, IL5RA, CXCL8, INPP5D, JAK1, JAK2, JUN, LYN, ENPP3, PIK3CD, PIK3R1, PIK3R2, PRKACA, MAPK1, MAPK3, MAPK8, MAP2K1, PTPN6, PTPN11, RAF1, SHC1, SOS1, SRC, STAT3, STAT5A, STAT5B, SYK, TGFB1, VAV1, YWHAB, GAB2, YWHAQ

Predefined gene sets in Hepamine

Data source	Pathways	Symbols
WIKIPATHWAYS	IL-4 Signaling Pathway	AKT1, BIRC5, BAD, CBL, CEBPA, CEBPB, CHUK, ATF2, MAPK14, ELK1, EP300, FES, FLNA, FOS, GATA3, GRB2, HRH1, IKBKB, IL2RG, IL4, IL4R, INPP5D, IRS1, JAK1, JAK2, JAK3, NFIL3, NFKB1, NFKBIA, PIK3CA, PIK3CD, PIK3R1, PIK3R2, MAPK1, MAPK3, MAPK11, PTPN6, PTPN11, RELA, RPS6KB1, SHC1, SOS1, STAT1, STAT3, STAT5A, STAT5B, STAT6, TYK2, SOCS1, IRS2, SOCS3, DOK2, SOCS5, GAB2, MIR1281
WIKIPATHWAYS	IL-5 Signaling Pathway	AKT1, BCL2, BTK, CSF2RB, ELK1, FOXO3, FOS, GRB2, GSK3A, GSK3B, IL2, IL5RA, JAK1, JAK2, JUN, KRAS, LYN, MAPT, MYC, PIK3CG, PIK3R1, PIK3R2, MAPK1, MAPK3, MAP2K1, MAP2K2, PTPN11, RAF1, RPS6, RPS6KA1, RPS6KB1, RPS6KB2, SHC1, SOS1, STAT1, STAT3, STAT5A, STAT5B, SYK, SPRED1
WIKIPATHWAYS	IL-6 signaling pathway	AGT, AKT1, BAD, BCL2L1, PRDM1, CREBBP, CRP, NR2F6, GAB1, GRB2, GSK3B, HCK, HDAC1, IL6, IL6R, IL6ST, IRF1, JAK1, JAK2, JUNB, PIK3R1, PIK3R2, PRKCD, MAPK1, MAPK3, MAP2K1, MAP2K2, PTPN11, RAC1, RPS6KB1, MAP2K4, SHC1, SOS1, STAT1, STAT3, MAP3K7, TIMP1, TYK2, VAV1, VIP, NCOA1, SOCS3, NLK
WIKIPATHWAYS	IL-7 Signaling Pathway	AKT1, BAD, CCND1, BCL2L1, CDKN1B, PTK2B, FYN, GSK3B, IL2RA, IL2RG, IL7, IL7R, JAK1, JAK3, MYC, PIK3R1, PIK3R2, MAPK1, MAPK3, MAP2K1, MAP2K2, STAT1, STAT3, STAT5A, STAT5B
WIKIPATHWAYS	IL-9 Signaling Pathway	CDK9, GRB2, IL2RG, IL9, IL9R, JAK1, JAK3, PIK3R1, PIK3R2, MAPK1, MAPK3, MAP2K1, MAP2K2, STAT1, STAT3, STAT5A, STAT5B, MIR2861, MIR3960
WIKIPATHWAYS	Imatinib and Chronic Myeloid Leukemia	ABL1, BCR, CDKN1B, CSF1R, GADD45A, FOXO3, FLT1, KIT, LYL1, MYC, NOP2, PDGFRA, PDGFRB, ABCB1, PIM1, SKP2, ABCG2, GAB2, PIM2, SPRED2, MIR1204

Predefined gene sets in Hepamine

Data source	Pathways	Symbols
WIKIPATHWAYS	Inflammatory Response Pathway	CD28, CD80, CD86, CD40, CD40LG, COL1A1, COL1A2, COL3A1, FN1, IFNG, IL2, IL2RA, IL2RB, IL2RG, IL4, IL4R, IL5, IL5RA, LAMA5, LAMB1, LAMB2, LAMC1, LAMC2, LCK, THBS1, THBS3, TNFRSF1A, TNFRSF1B, VTN, ZAP70, MIR3606, MIR4758, MIR7846
WIKIPATHWAYS	Influenza A virus infection	BCL2, MIR34C
WIKIPATHWAYS	Inhibition of exosome biogenesis and secretion by Manumycin A in CRPC cells	ARAF, BRAF, HNRNPH1, HRAS, KRAS, NRAS, MAPK1, MAPK3, RAB5A, RAB5B, RAB27A, RAB5C, RAF1, RRAS, HGS, PDCD6IP, RRAS2, MRAS
WIKIPATHWAYS	Initiation of transcription and translation elongation at the HIV-1 LTR	CCNT1, CDK9, CREBBP, EP300, HDAC1, HDAC2, NFATC1, NFATC2, NFATC3, NFATC4, NFKB1, NFKBIA, PPP3CA, PPP3CB, PPP3CC, PPP3R1, PPP3R2, RELA, SP1, SUPT4H1, SUPT5H, NELFA, NELFE, HDAC3, HDAC9, HDAC4, HDAC5, HEXIM1, NELFB, NELFCD, HDAC7, HDAC8, MIR943, MIR1281, MIR1236, MIR2861, MIR3960

Predefined gene sets in Hepamine

Data source	Pathways	Symbols
WIKIPATHWAYS	Insulin Signaling	AKT1, AKT2, ARF1, ARF6, CBL, CBLB, MAP3K8, CRK, MAPK14, EGR1, EIF4E, EIF4EBP1, ELK1, FOXO1, FOXO3, FLOT2, FOS, MTOR, GAB1, GRB2, GRB10, GRB14, RAPGEF1, GSK3A, GSK3B, GYG1, GYS1, GYS2, HRAS, IGF1R, IKBKB, INPP4A, INPPL1, INSR, IRS1, JUN, KIF5B, LIPE, MAP3K1, MAP3K3, MAP3K4, MAP3K5, MAP3K9, MAP3K10, MAP3K11, MYO1C, ENPP1, PDPK1, PFKL, PFKM, PIK3C2A, PIK3C2G, PIK3C3, PIK3CA, PIK3CB, PIK3CD, PIK3CG, PIK3R1, PIK3R2, PPP1R3A, PRKAA1, PRKAA2, PRKCA, PRKCB, PRKCD, PRKCH, PRKCI, PRKCQ, PRKCZ, MAPK1, MAPK3, MAPK4, MAPK6, MAPK7, MAPK8, MAPK11, MAPK9, MAPK10, MAPK13, MAP2K1, MAP2K2, MAP2K3, MAP2K5, MAP2K6, MAP2K7, PTEN, PTPN1, PTPN11, PTPRF, RAB4A, MAP4K2, RAC1, RAC2, RAF1, REG1A, RHEB, RPS6KA1, RPS6KA2, RPS6KA3, RPS6KB1, RPS6KB2, RRAD, MAPK12, MAP2K4, SGK1, SHC1, SLC2A1, SLC2A4, SNAP25, SOS1, SOS2, SRF, STX4, STXBP1, STXBP2, STXBP3, VAMP2, MAP3K7, TSC1, TSC2, XBP1, MAP3K12, IRS4, MAP4K3, PIK3R3, SOCS1, IRS2, SNAP23, RPS6KA4, MAP3K14, SOCS3, MAP3K6, MAP3K13, RPS6KA5, CYTH3, MAP4K4, TBC1D4, SGK2, FLOT1, CAP1, SORBS1, SH2B2, MAP3K2, EHD1, KIF3A, MAP4K5, MAP4K1, RHOQ, CBLC, SGK3, SHC2, RPS6KA6, EHD2, PIK3R4, MINK1, SHC3, RHOJ, TRIB3, ARHGAP33, STXBP4, MIR3620
WIKIPATHWAYS	Insulin signalling in human adipocytes (diabetic condition)	AKT2, MTOR, INSR, IRS1, RPS6, RPS6KB1, SLC2A4, TBC1D4
WIKIPATHWAYS	Insulin signalling in human adipocytes (normal condition)	AKT2, MTOR, INSR, IRS1, RPS6, RPS6KB1, SLC2A4, TBC1D4

Predefined gene sets in Hepamine

Data source	Pathways	Symbols
WIKIPATHWAYS	Integrated Breast Cancer Pathway	ABL1, AHR, AKT1, ANXA1, AR, ARAF, ATF1, ATM, ATR, BACH1, BAD, BAK1, BARD1, BAX, CCND1, BCL2, BID, BLM, BMPR1A, BMPR2, BRCA1, BRAF, BRCA2, CASP3, CASP8, CASP9, CDC25A, CDC25B, CDC42, CDH1, CDK2, CDK4, CDK7, CHEK1, CHUK, PLK3, CREB1, CSNK1D, CTNNB1, CYP19A1, DAG1, GADD45A, E2F1, EGFR, EP300, ESR1, FAU, FER, FOXO1, MTOR, GDI1, GRN, GSK3A, MSH6, HDAC1, HMGCR, IMPA1, IRS1, JAK1, JUN, KRAS, SMAD1, SMAD2, SMAD4, SMAD6, SMAD7, MAX, MDM2, MMP1, MRE11, MSH2, MYC, MYT1, NAB1, NF1, NFKB1, ODC1, PAK1, PHB, PIGR, PIK3R2, PLK1, PML, PKIA, MAPK1, PTEN, RAC1, RAD51, RALA, RAP1A, RB1, RHEB, RHO, RRAS, SMARCA4, SP1, STAT1, AURKA, STK11, TFPI, TGFBR1, TGFBR2, TP53, TPR, TSC1, TSC2, VEGFA, WEE1, XRCC3, FOSL1, NCOA3, RAD54L, PIAS1, TRADD, FADD, ALKBH1, MAP3K13, USP15, RAD50, TAB1, RPP38, USP16, NOXA1, EDAR, CHEK2, MYCBP2, SIRT1, ZMYND8, RASGRP3, ERAL1, USP21, FILIP1, HIPK2, LGALS13, DHTKD1, PPP4R3A, MAP3K7CL, ZMIZ1, PPP4R3B, CCNB1IP1, APOBEC3G, CERK, ZNF655, DCAKD, NUP85, ITPKC, USP38, UBE2F, JAKMIP1, RASGEF1A, RALGAPA1, MIR1281, GRIK1-AS2, MIR6125
WIKIPATHWAYS	Integrated Cancer Pathway	AKT1, ATF1, ATM, ATR, BACH1, BAD, BARD1, BAX, BCL2, BLM, BRCA1, CASP3, CASP8, CASP9, CDK1, CDC25A, CDC25B, CDK2, CDK4, CDKN1A, CDKN1B, CDKN2B, CHEK1, E2F1, MSH6, JAK1, SMAD2, SMAD3, MDM2, MAP3K5, MMP1, MRE11, MSH2, MYC, PLK1, POU2F1, PTEN, RB1, STAT1, TP53, RAD50, NOXA1, CHEK2, BBC3, GRIK1-AS2, MIR3191

Predefined gene sets in Hepamine

Data source	Pathways	Symbols
WIKIPATHWAYS	Integrin-mediated Cell Adhesion	AKT1, AKT2, ARAF, BRAF, CAPN5, CAPN1, CAPN2, CAPN3, CAPNS1, CAPN6, CAV1, CAV2, CAV3, CDC42, CRK, CSK, DOCK1, FYN, GRB2, RAPGEF1, HRAS, ILK, ITGA6, ITGA1, ITGA2, ITGA2B, ITGA3, ITGA4, ITGA5, ITGA7, ITGA9, ITGAD, ITGAE, ITGAL, ITGAM, ITGAV, ITGAX, ITGB1, ITGB2, ITGB3, ITGB4, ITGB5, ITGB6, ITGB7, ITGB8, PAK1, PAK2, PAK3, PDPK1, PIK3R2, MAPK1, MAPK4, MAPK6, MAPK7, MAPK10, MAP2K1, MAP2K2, MAP2K3, MAP2K5, MAP2K6, PTK2, PXN, RAC1, RAC2, RAC3, RAF1, RAP1A, RAP1B, RHO, ROCK1, MAPK12, SELENOP, SHC1, SOS1, SRC, TLN1, TNS1, VASP, VAV2, VCL, ZYX, ITGA10, ITGA8, ARHGEF7, ROCK2, BCAR1, GIT2, AKT3, PAK4, VAV3, SORBS1, CAPN9, CAPN11, CAPN10, ITGA11, CAPN7, SHC3, PAK6, MYPN, MYLK2, MIR34C, LINC02478, MIR6852, BUB1B-PAK6
WIKIPATHWAYS	Interactome of polycomb repressive complex 2 (PRC2)	EZH1, EZH2, JARID2, RBBP4, RBBP7, ELL, EED, BCLAF1, THRAP3, STK38, MTF2, SETX, TRIM35, SUZ12, MORC3, AEBP2, MIR6755
WIKIPATHWAYS	Interferon type I signaling pathways	CBL, CREB1, CRK, CRKL, MAPK14, EIF4A1, EIF4B, EIF4E, EIF4EBP1, MTOR, FYN, RAPGEF1, PRMT1, IFNAR1, IFNAR2, IRS1, JAK1, LCK, MAP3K1, PIK3CD, PIK3R1, PIK3R2, MAP2K3, MAP2K6, PTPN6, PTPN11, PTPRC, RAC1, RAP1A, REL, RPS6, RPS6KB1, STAT1, STAT2, STAT3, STAT4, STAT5A, TYK2, VAV1, ZAP70, PIAS1, SOCS1, IRS2, RPS6KA4, SOCS3, RPS6KA5, GAB2, IRF9, RACK1, PIAS3, PDCD4, RPTOR, MLST8, MAPKAP1, MIR4680
WIKIPATHWAYS	Interleukin-11 Signaling Pathway	AKT1, BIRC5, ATF1, BCL2, BGLAP, RUNX2, CHUK, CREB1, FES, FYN, GRB2, HRAS, IBSP, ICAM1, IKBKB, IL6ST, IL11, IL11RA, ITGA2, JAK1, JAK2, PDPK1, PIK3R1, PIK3R2, PTPA, MAPK1, MAPK3, MAP2K1, MAP2K2, PTPN11, RAF1, RELA, RPS6, RPS6KA1, RPS6KB1, SRC, STAT1, STAT3, TGFB1, TYK2, YES1, PIAS1, SOCS3, PIAS3
WIKIPATHWAYS	Interleukin-1 Induced Activation of NF-kappa-B	IL1A, IRAK1, PRKCZ, TRAF6, UBE2N, UBE2V1, SQSTM1, AJUBA, TIFA, MIR718

Predefined gene sets in Hepamine

Data source	Pathways	Symbols
WIKIPATHWAYS	Irinotecan Pathway	BCHE, CES1, ABCC2, CYP3A4, CYP3A5, ABCC1, NPC1, CES2, ABCG2, SLCO1B1, UGT1A10, UGT1A9, UGT1A1
WIKIPATHWAYS	Iron metabolism in placenta	ACO1, FTH1, IREB2, SLC11A2, TF, TFR2, TFRC, SLC40A1, STEAP3, MCOLN1, HAMP, HEPHL1
WIKIPATHWAYS	Kennedy pathway from Sphingolipids	CHKA, CHKB, PCYT1A, PCYT2, SGPL1, PCYT1B, PTDSS1, CEPT1, PEMT, ETNK2, ETNK1, CHPT1, PTDSS2
WIKIPATHWAYS	Kit receptor signaling pathway	AKT1, BAD, BCL2, BTK, CBL, CRK, CRKL, MAPK14, DOK1, EP300, FOXO3, FOS, FYN, GRB2, GRB7, GRB10, HRAS, INPP5D, JAK2, JUNB, KIT, LYN, MAPT, MATK, KITLG, MITF, PIK3R1, PIK3R2, PLCG1, PRKCA, PRKCB, MAPK1, MAPK3, MAPK8, MAP2K1, MAP2K2, PTPN6, PTPN11, RAF1, RPS6, RPS6KA1, RPS6KA3, RPS6KB1, SHC1, SNAI2, SNAI1, SOS1, SRC, STAT1, STAT3, STAT5A, STAT5B, TBX2, TEC, VAV1, SOCS1, SOCS6, GAB2, SH2B2, MIR1281
WIKIPATHWAYS	Lamin A-processing pathway	LMNA, ZMPSTE24, ICMT
WIKIPATHWAYS	Leptin and adiponectin	ACACA, CPT1A, LEP, LEPR, PRKAA1, PRKAB1, PRKAG1, ADIPOQ, ADIPOR1, ADIPOR2
WIKIPATHWAYS	Leptin Insulin Overlap	AKT1, INS, INSR, IRS1, JAK2, LEP, LEPR, PDPK1, PIK3CG, STAT3, IRS4, PIK3R3, DGKZ, SOCS1, IRS2, SOCS2, SOCS3
WIKIPATHWAYS	Leptin signaling pathway	ACACA, ACACB, AKT1, RHOA, BAD, BAX, CCND1, BCL2L1, CDC42, CFL2, CHUK, CISH, CREB1, MAPK14, EIF4E, EIF4EBP1, ELK1, ERBB2, ESR1, FOXO1, MTOR, FYN, GRB2, GSK3A, GSK3B, HRAS, IKBKB, IL1B, IL1RN, IRS1, JAK1, JAK2, KPNA4, LEP, LEPR, NFKB1, NOS3, PDE3B, PIK3R1, PIK3R2, PLCG1, PLCG2, PRKAA1, PRKAA2, MAPK1, MAPK3, MAPK8, MAP2K1, MAP2K2, PTEN, PTK2, PTPN1, PTPN11, RAC1, RAF1, REL, RELA, ROCK1, RPS6, RPS6KA1, RPS6KB1, SHC1, SOS1, SP1, SRC, STAT1, STAT3, STAT5B, IKBKG, NCOA1, SOCS2, SOCS3, ROCK2, KHDRBS1, SH2B1, SOCS7

Predefined gene sets in Hepamine

Data source	Pathways	Symbols
WIKIPATHWAYS	let-7 inhibition of ES cell reprogramming	EGR1, MYC, POU5F1, SOX2, KLF4, TRIM71, MIRLET7A1, MIRLET7A2, MIRLET7C, MIRLET7D, MIRLET7E, MIRLET7F1, MIRLET7F2, MIRLET7G, MIRLET7I
WIKIPATHWAYS	Lidocaine metabolism	CYP1A2, CYP3A4
WIKIPATHWAYS	Lipid Metabolism Pathway	ACACA, ACLY, AKT1, AKT2, BCKDHA, FASN, LIPE, PDHA1, PLIN1, PRKAA1, PRKAA2, PRKAB1, PRKAB2, PRKACA, PRKACB, PRKACG, PRKAG1, PRKAR1A, PRKAR1B, PRKAR2A, PRKAR2B, AKT3, ACSBG1, HILPDA, ABHD5, PRKAG2, PRKAG3, ACSS2, PNPLA2
WIKIPATHWAYS	Liver X Receptor Pathway	CYP2B6, CYP3A4, CYP7A1, FASN, RXRA, SCD, SREBF1, NR1H3, ABCG5, ABCG8
WIKIPATHWAYS	LncRNA involvement in canonical Wnt signaling and colorectal cancer	APC, ATF3, CCND1, CCND2, CCND3, CDK6, CDK8, CSNK1A1, CSNK1E, CSNK2A1, CSNK2A2, CSNK2B, CTBP1, CTBP2, CTNNB1, DVL1, DVL2, DVL3, EZH2, FZD2, GSK3B, HNRNPK, HNRNPU, JUN, LRP6, LRP5, MYC, ROR1, ROR2, SERPINF1, PLAU, RYK, SFRP1, SFRP2, SFRP4, SFRP5, MAP3K7, TCF3, TCF7, TCF7L2, TFAP2A, WNT1, WNT2, WNT3, WNT5A, WNT6, WNT7A, WNT7B, WNT10B, WNT11, WNT2B, FZD5, FZD3, FOSL1, AXIN1, AXIN2, FZD1, FZD6, FZD7, FZD8, FZD9, RUVBL1, CER1, FRAT1, APC2, WIF1, FZD10, DKK1, FRAT2, DKK4, DKK2, SOST, LEF1, WNT16, NLK, WNT4, H2AFY2, CTNNBIP1, CHD8, SENP2, SOX17, PORCN, CXXC4, WNT10A, WNT5B, TCF7L1, KREMEN1, NKD1, NKD2, WNT3A, CSNK1A1L, NOTUM, CSNK2A3, H19, MIR16-1, MIR16-2, MIR34A, MIR7-1, MIR675, MIR4683, LOC101929777, MIR6808, TPTEP2-CSNK1E, MIR34AHG
WIKIPATHWAYS	LncRNA-mediated mechanisms of therapeutic resistance	BCL2L1, CDKN1A, HIF1A, ABCB1, TP53, WNT6, MEG3, VLDLR-AS1, UCA1, HOTAIR, LINC-ROR, LINC00970

Predefined gene sets in Hepamine

Data source	Pathways	Symbols
WIKIPATHWAYS	Lung fibrosis	BMP7, CALCA, CEBPB, CMA1, CCR3, CSF2, CSF3, CCN2, DSP, EDN1, EGF, ELN, FGF1, FGF2, FGF7, CXCL2, HGF, HMOX1, IGF1, IL1B, IL4, IL5, IL6, CXCL8, IL12B, IL13, SMAD7, MECP2, MMP2, MMP9, MT2A, NFE2L2, PARN, PDGFA, PDGFB, SERPINA1, PLAU, PTX3, CCL2, CCL3, CCL4, CCL5, CCL11, SFTPC, SKIL, SPP1, TERT, TGFA, TGFB1, TIMP1, TNF, FAM13A, ATP11A, GREM1, RTEL1, CYSLTR2, STN1, DPP9, ELMOD2, SFTPA1, MUC5B, CCR2, SFTPA2
WIKIPATHWAYS	Macrophage markers	CD14, CD86, CD68, CD74, F3, LYZ, RAC2, CD83, CD163
WIKIPATHWAYS	MAPK and NFkB Signalling Pathways Inhibited by Yersinia YopJ	CHUK, IKBKB, MAP3K1, NFKB1, NFKBIA, MAPK1, MAP2K6, RAF1, RRAS, TRAF6, IKBKG, MAP3K14
WIKIPATHWAYS	MAPK Cascade	ARAF, BRAF, MAPK14, ELK1, HRAS, JUN, KRAS, MAP2, MBP, MAP3K1, MAP3K3, NRAS, PLCB3, MAPK1, MAPK3, MAPK10, MAP2K1, MAP2K2, MAP2K3, MAP2K6, MAP2K7, RAF1, RRAS, MAPK12, MAP2K4, SIPA1, MAP3K12, MAP3K2, RASA3

Predefined gene sets in Hepamine

Data source	Pathways	Symbols
WIKIPATHWAYS	MAPK Signaling Pathway	AKT1, AKT2, FAS, FASLG, ARRB1, ARRB2, ATF4, BDNF, BRAF, CACNA1A, CACNA1B, CACNA1C, CACNA1D, CACNA1E, CACNA1F, CACNA1S, CACNA2D1, CACNB1, CACNB2, CACNB3, CACNB4, CACNG1, CASP3, CD14, CDC25B, CDC42, CHUK, MAP3K8, ATF2, CRK, CRKL, MAPK14, DAXX, GADD45A, DDIT3, DUSP1, DUSP2, DUSP3, DUSP4, DUSP6, DUSP7, DUSP8, DUSP9, EGF, EGFR, ELK1, ELK4, FGF1, FGF2, FGF3, FGF4, FGF5, FGF6, FGF7, FGF8, FGF9, FGF10, FGF11, FGF12, FGF13, FGF14, FGFR1, FGFR3, FGFR2, FGFR4, FLNA, FLNB, FLNC, FOS, GNA12, MKNK2, GRB2, NR4A1, HRAS, HSPA1A, HSPA1B, HSPA1L, HSPA2, HSPA6, HSPA8, HSPB1, IKBKB, IL1A, IL1B, IL1R1, JUN, JUND, KRAS, STMN1, MAPT, MAX, MEF2C, MAP3K1, MAP3K4, MAP3K5, MAP3K11, MOS, MYC, NF1, NFATC1, NFATC3, NFKB1, NFKB2, NGF, NRAS, NTF3, NTF4, NTRK1, NTRK2, PAK1, PAK2, PDGFA, PDGFB, PDGFRB, PLA2G4A, PPM1A, PPM1B, PPP3CA, PPP3CB, PPP3CC, PPP3R1, PPP3R2, PPP5C, PRKACA, PRKACB, PRKACG, PRKCA, PRKCD, PRKCG, MAPK1, MAPK3, MAPK7, MAPK8, MAPK11, MAPK9, MAPK10, MAPK13, MAP2K1, MAP2K2, MAP2K3, MAP2K5, MAP2K6, MAP2K7, PTPN7, PTPRR, MAP4K2, RAC1, RAC2, RAC3, RAF1, RAP1A, RAP1B, RASA1, RASA2, RASGRF1, RASGRF2, RELA, RELB, RPS6KA3, RRAS, MAPK12, MAP2K4, SOS1, SOS2, SRF, STK3, STK4, MAP3K7, TGFB1, TGFB2, TGFB3, TGFBR1, TGFBR2, TNF, TNFRSF1A, TP53, TRAF2, TRAF6, MAP3K12, IL1R2, MAPKAPK3, FGF23, MAP4K3, IKBKG, MAPKAPK5, MKNK1, PLA2G4C, LAMTOR3, FGF18, FGF17, FGF16, CACNA1I, CACNA1H, CACNA1G, RPS6KA4, MAP3K14, MAP3K6, MAP3K13, RPS6KA5, CACNA2D2, MAPKAPK2, TAOK2, MAP4K4, MAPK8IP1, RAPGEF2, FGF19, AKT3, RASGRP1, RASGRP2, CACNG3, CACNG2, TAB1, MAP3K2, MAP4K1, DUSP10, RRAS2, MRAS, TAB2, MAPK8IP3, MAPK8IP2, RASGRP3, FGF20, FGF21, FGF22, CACNG5, CACNG4, ECSIT, TAOK3, NLK, MAP3K20, CACNA2D3, GNG12, TAOK1, CACNG8, CACNG7, CACNG6, DUSP16, PTPN5, CACNA2D4, RASGRP4, LRRK2, PLA2G4E, PLA2G4F, PLA2G4D, MIR935, PLA2G4B, MIR3917, PPP5D1, MIR4523

Predefined gene sets in Hepamine

**Data source**	**Pathways**	**Symbols**
WIKIPATHWAYS	Matrix Metalloproteinases	BSG, MMP1, MMP2, MMP3, MMP7, MMP8, MMP9, MMP10, MMP11, MMP12, MMP13, MMP14, MMP15, MMP16, MMP17, MMP19, TCF20, TIMP1, TIMP2, TIMP3, TIMP4, TNF, MMP23B, MMP20, MMP24, MMP26, MMP27, MMP25, MMP28, MMP21
WIKIPATHWAYS	MECP2 and Associated Rett Syndrome	AKT1, BCL6, BDNF, CAMK2A, CEBPD, CNP, CREB1, CSRP1, DLX5, DLX6, E2F1, EZH2, FGF2, FGF3, FGF4, FGF5, FKBP5, MTOR, FUT8, GABRR2, GAD1, GAMT, GRIA1, GRID1, GRIN1, HDAC1, HNRNPF, HNRNPH1, IGF2, MAG, MBP, MECP2, MEF2C, MPP1, MYT1, NF1, YBX1, OPRK1, POU3F2, POU4F1, REST, RPS6, SGK1, SP1, SP3, SST, TAF1, TAP1, UBE3A, SMC3, NREP, NCOR1, CTCF, SIN3A, ARHGEF26, CDON, RBFOX1, TET2, TET1, PRPF38A, GPRIN1, TET3
WIKIPATHWAYS	Melatonin metabolism and effects	AANAT, ACHE, ADRB1, APOE, ARNTL, ASMT, CALM1, CALM2, CAMK2A, CRY1, CRY2, CSNK1D, CSNK1E, CYP1A1, CYP1A2, CYP1B1, CYP2C19, CYP2D6, ECE1, EDN1, FOXO1, GSK3B, IRAK1, MAOA, MAP2, MTNR1A, MTNR1B, NFKB1, PER1, PRKCA, SULT1A1, TRAF6, PER3, PER2, CLOCK, SIRT1, MIR126, MIR146A, MIR718, MIR6883, TPTEP2-CSNK1E, MIR3142HG

Predefined gene sets in Hepamine

Data source	Pathways	Symbols
WIKIPATHWAYS	Mesodermal Commitment Pathway	ACACA, ACVR1, ACVR2A, ACVR2B, AMH, CCND1, BMP4, BMP7, BMPR1A, BMPR2, KLF5, C1QBP, CSRP2, CTBP2, DNMT3B, ELK4, EXT1, EXT2, FGF8, FGFR1, FOXC1, FOXC2, GATA3, GATA6, HTT, FOXA1, FOXA2, HNF4A, HPRT1, INHBA, JAK2, JARID2, SMAD1, SMAD2, SMAD3, SMAD4, SMAD6, MEIS1, NFE2L2, NODAL, PAX6, PBX1, PBX3, PITX2, POU5F1, PPP2CA, PRKACA, PRKAR1A, RARB, RARG, RGS10, RPL38, SNAI1, SOX2, SRF, TBX1, TBX6, TCF4, TBX3, TEAD1, LEFTY2, KDM6A, WNT3, ZIC2, ZIC3, FZD5, CCDC6, HMGA2, AXIN1, AXIN2, EOMES, FZD4, FZD8, CUL4B, TEAD2, BHLHE40, PIAS1, CHRD, ADAM19, FOXH1, LATS1, NOG, KLF4, HAND1, GDF3, TOX, ARL4A, TRIM28, YAP1, VAV3, LEFTY1, ASCC3, SOX21, WDHD1, CEP250, MTF2, DKK1, PLCH1, DIP2A, CRTC1, MACF1, ZNF281, DDAH1, ELP4, SESN1, AHDC1, TOX3, DLL1, SETD2, SCHIP1, LEF1, UBR5, NLK, MBTD1, NCAPG2, CCDC88A, TRERF1, EMSY, TWSG1, ATP8B2, EPB41L5, ZNF462, BCORL1, SOX17, NABP2, PARP8, ZFHX4, NANOG, GRHL2, WDCP, TET1, TCF7L1, MIXL1, ARID5B, PHF6, HES7, TRIM5, ZIC5, WNT3A, WDFY2, AEBP2, TRIM71, SLC2A12, C9orf72, MSGN1, C6orf201, MIR125B1, MIR141, MIR200A, MIR302C, MIR372, MIR373, MIR375, KIAA0754, MIR4321, MIR4683, LOC101929777
WIKIPATHWAYS	Metabolic reprogramming in colon cancer	ACLY, ACO2, ALDOB, DLST, ENO1, FASN, FH, G6PD, GAPDH, GART, GLS, GLUD1, GOT2, GPI, HK3, IDH2, IDH3A, LDHA, MDH2, PDHA1, PDHB, PFKL, PGAM1, PGD, PGK1, PKM, PPAT, PSPH, PYCR1, SDHB, SHMT2, SLC1A5, SLC2A1, TALDO1, TKT, SUCLG2, SLC16A3, PAICS, RPIA, PYCR2, PSAT1, TIGAR, MIR6787, MIR6741
WIKIPATHWAYS	Metabolism of Dichloroethylene by CYP450	CYP2E1
WIKIPATHWAYS	Metabolism of Spingolipids in ER and Golgi apparatus	B4GALNT1, GALNT1, B4GALT1, B3GALNT1, B3GALT1, SPHK1, SPHK2, GALNT16, ST6GALNAC3
WIKIPATHWAYS	Metabolism of Tetrahydrocannabinol (THC)	CYP2C9, CYP3A4

**Data source**	**Pathways**	**Symbols**
WIKIPATHWAYS	Metapathway biotransformation Phase I and II	NAT2, AKR1B1, BAAT, AKR1C4, COMT, CYP1A1, CYP1A2, CYP1B1, CYP2A6, CYP2A7, CYP3A7, CYP2A13, CYP2B6, CYP2C19, CYP2C8, CYP2C9, CYP2C18, CYP2D6, CYP2E1, CYP2F1, CYP2J2, CYP3A4, CYP3A5, CYP4B1, CYP7A1, CYP8B1, CYP11A1, CYP11B1, CYP11B2, CYP17A1, CYP19A1, CYP21A2, CYP24A1, CYP26A1, CYP27A1, CYP27B1, CYP51A1, AKR1C1, AKR1C2, EPHX1, EPHX2, FMO1, FMO2, FMO3, FMO4, FMO5, GPX2, GPX3, GPX4, GPX5, GSR, GSS, GSTA1, GSTA2, GSTA3, GSTA4, GSTM1, GSTM2, GSTM3, GSTM4, GSTM5, GSTP1, GSTT2, GSTZ1, HNMT, NDST1, CYP4F3, CHST6, MGST1, MGST2, MGST3, NNMT, AKR1D1, SULT1E1, SULT1A2, SULT1A1, SULT1C2, SULT2B1, SULT2A1, TPMT, UGT2B4, UGT2B7, UGT2B15, UGT2B17, KCNAB1, NDST2, KCNAB2, CYP4F2, CHST1, AKR7A2, AKR1C3, NAT8, KCNAB3, NDST3, HS6ST1, CYP7B1, CHST2, GSTO1, CHST3, CHST10, GAL3ST1, HS2ST1, HS3ST4, HS3ST3B1, HS3ST3A1, HS3ST2, HS3ST1, CHST4, GLYAT, AKR1A1, UGT2B11, CYP46A1, UGT2A1, INMT, CYP4F8, AKR7A3, CHST5, NAA80, SULT4A1, NAT9, SULT1C4, SULT1B1, CYP2S1, CHST11, NAA20, CYP39A1, UGT2B28, UGT1A10, UGT1A7, UGT1A6, UGT1A5, UGT1A9, UGT1A4, UGT1A1, UGT1A3, CYP2W1, NAT10, CHST12, CHST7, CYP26B1, AKR1B10, NAT14, CYP20A1, CYP4F11, GAL3ST2, CHST8, NDST4, HS3ST6, CYP3A43, CYP4F12, GAL3ST4, UGT2A3, GSTCD, NAA40, NAA50, CHST9, GAL3ST3, HS6ST2, GLYATL1, CHST14, CYP2U1, GSTO2, CYP2R1, NAA30, CYP4F22, CHST13, CYP4Z1, GLYATL2, GSTA5, HS3ST5, CYP4X1, HS6ST3, CYP4V2, CYP27C1, NAT8L, CYP26C1, GSTK1, SULT6B1, SULT1C3, SULT1A4, UGT2A2, GSTT2B, LOC100287413, SLX1A-SULT1A3, SLX1B-SULT1A4, MIR4761, LOC101927181, LOC105369243

Predefined gene sets in Hepamine

Data source	Pathways	Symbols
WIKIPATHWAYS	Metastatic brain tumor	CDC42, CDK6, E2F3, MYC, PIK3R1, TP53, MIRLET7BHG, MIRLET7A1, MIRLET7A2, MIRLET7A3, MIRLET7B, MIRLET7C, MIRLET7D, MIRLET7E, MIRLET7F1, MIRLET7F2, MIRLET7G, MIR101-1, MIR101-2, MIR148A, MIR29A, MIR29B1, MIR29B2, MIR29C, MIR34B, MIR34C, MIR148B, MIR4763
WIKIPATHWAYS	Methionine De Novo and Salvage Pathway	AHCY, AMD1, BHMT, MAT1A, MAT2A, MSRA, MTAP, MTR, ODC1, SMS, SRM, TAT, TXN, MSRB2, MAT2B, APIP, ADI1, CHDH, ENOPH1, MRI1, MSRB3, IL4I1
WIKIPATHWAYS	Methylation Pathways	COMT, HNMT, MAT1A, MAT2A, NNMT, PNMT, TPMT, INMT, MAT2B, MIR4761
WIKIPATHWAYS	MET in type 1 papillary renal cell carcinoma	AKT1, AKT2, ALK, ARAF, BAD, BRAF, CBL, CDC42, CDKN1A, CRK, CRKL, ETS1, GAB1, GRB2, RAPGEF1, HGF, HRAS, INSL3, JAK3, JUN, KRAS, MET, NRAS, PAK1, PAK2, PAK3, PIK3CA, PIK3CB, PIK3CD, PIK3R1, PIK3R2, PLCG1, PRCC, MAPK1, MAPK3, MAPK8, MAP2K1, MAP2K2, PTK2, PTPN11, RAC1, RAF1, RAP1A, RAP1B, RPL11, SOS1, SOS2, SRC, STAT3, STRN, ELOA, TFE3, PIK3R3, AKT3, PAK4, PAK6, PAK5, C8orf34, BUB1B-PAK6
WIKIPATHWAYS	Mevalonate arm of cholesterol biosynthesis pathway with inhibitors	HMGCR, MVD
WIKIPATHWAYS	Mevalonate pathway	ACAT2, FDPS, HMGCR, HMGCS1, MVD, MVK, PMVK
WIKIPATHWAYS	MFAP5-mediated ovarian cancer cell motility and invasiveness	CREB1, ITGAV, ITGB3, ITPR3, JUN, PLCG1, PRKCQ, MAPK1, MAPK3, PTK2, RYR3, TNNC1, MFAP5
WIKIPATHWAYS	Microglia Pathogen Phagocytosis Pathway	C1QA, C1QB, C1QC, CYBA, CYBB, FCER1G, FCGR1A, HCK, NCKAP1L, ITGAM, ITGB2, LYN, NCF2, NCF4, PIK3C2A, PIK3C3, PIK3CA, PIK3CB, PIK3CD, PIK3CG, PIK3R1, PIK3R2, PLCG2, PTPN6, RAC1, RAC2, RAC3, SYK, TYROBP, VAV1, VAV2, PIK3R3, ARPC1B, VAV3, SIGLEC7, LAT, TREM2, TREM1, PIK3R6, NCF1
WIKIPATHWAYS	MicroRNA for Targeting Cancer Growth and Vascularization in Glioblastoma	HIF1A, HES1, VEGFA, VEGFB, VEGFC, HEY1, HIF1AN, MIR148A, MIR31

Predefined gene sets in Hepamine

Data source	Pathways	Symbols
WIKIPATHWAYS	MicroRNAs in cardiomyocyte hypertrophy	AGT, AKT1, AKT2, RHOA, CALM1, CALM2, CAMK2D, CDK7, CDK9, CHUK, CISH, MAPK14, CTF1, CTNNB1, RCAN1, DVL1, EDN1, EGF, FGF2, FGFR2, MTOR, FZD2, GATA4, GSK3B, NRG1, IGF1, IGF1R, IKBKB, IL6ST, LIF, LRP6, LRP5, MYLK, NFATC4, NFKB1, NPPA, NPPB, PDPK1, PIK3CA, PIK3CB, PIK3CD, PIK3CG, PIK3R1, PIK3R2, PLA2G2A, PLCB2, PPP3CA, PPP3CB, PRKCB, PRKG1, MAPK1, MAPK3, MAPK4, MAPK7, MAPK8, MAP2K1, MAP2K2, MAP2K3, MAP2K5, MAP2K6, MAP2K7, RAC1, RAF1, ROCK1, MAP2K4, STAT3, TGFB1, TNF, WNT5A, FZD1, PIK3R3, IKBKG, EIF2B5, MAP3K14, ROCK2, IKBKE, HDAC9, HDAC4, HDAC5, TAB1, MYEF2, HDAC7, WNT3A, MYLK3, MIR103A1, MIR103A2, MIR125B1, MIR125B2, MIR130B, MIR140, MIR15B, MIR185, MIR199A1, MIR199A2, MIR208A, MIR23A, MIR27B, MIR30E, MIR133B, MIR2861, MIR3960, MIR6808
WIKIPATHWAYS	mir-124 predicted interactions with cell cycle and differentiation	PRKAA1, PTBP1, STK11, SIX4, STRADB, CTDSP1, MIR4745
WIKIPATHWAYS	miR-222 in Exercise-Induced Cardiac Growth	CDKN1B, HIPK2, HMBOX1, HIPK1
WIKIPATHWAYS	mir34a and TGIF2 in osteoclastogenesis	TGIF2, MIR34A, MIR34AHG
WIKIPATHWAYS	miR-509-3p alteration of YAP1/ECM axis	COL1A1, COL3A1, COL5A1, EDNRA, FN1, PBX3, SNAI2, SPARC, TEAD1, TEAD4, TEAD3, THBS2, TWIST1, TEAD2, BCAR1, GPC6, YAP1, MIR509-3, MIR3606
WIKIPATHWAYS	miR-517 relationship with ARCN1 and USP1	ARCN1, CDKN1A, ID1, ID2, USP1
WIKIPATHWAYS	miRNA Biogenesis	RAN, TARBP2, DICER1, DROSHA, DGCR8, XPO5, MIR1306, MIR3618

Predefined gene sets in Hepamine

Data source	Pathways	Symbols
WIKIPATHWAYS	miRNA Regulation of DNA Damage Response	ABL1, APAF1, FAS, ATM, ATR, BAX, CCND1, BID, BRCA1, CASP3, CASP8, CASP9, CCNB1, CCND2, CCND3, CCNE1, CCNG1, CDK1, CDC25A, CDC25C, CDK2, CDK4, CDK5, CDK6, CDKN1A, CDKN1B, CHEK1, CREB1, DDB2, GADD45A, E2F1, FANCD2, SFN, H2AFX, MCM7, MDM2, MRE11, MYC, GADD45B, NBN, PMAIP1, PML, PRKDC, RAD1, RAD9A, RAD17, RAD51, RAD52, RB1, RFC1, RPA2, TP53, SMC1A, TNFRSF10B, CCNB2, CCNE2, TLK1, RAD50, GADD45G, TLK2, CHEK2, BBC3, SESN1, RRM2B, CYCS, PIDD1, TP53AIP1, ATRIP, CCNB3, HUS1B, CDC20B, MIR106B, MIR16-1, MIR17, MIR18A, MIR19A, MIR19B1, MIR20A, MIR203A, MIR210, MIR181A1, MIR221, MIR222, MIR34B, MIR92A1, MIR17HG, MIR373, MIR449A, MIR421, MIR449B, MIR3191, MIR3074, MIR3591
WIKIPATHWAYS	miRNA regulation of p53 pathway in prostate cancer	APAF1, ATM, BAX, BID, CASP3, CASP8, CASP9, DDB2, MDM2, SERPINE1, PMAIP1, PTEN, SIAH1, TP53, TNFRSF10B, EI24, CHEK2, BBC3, SHISA5, TP53AIP1, PERP, ZMAT3, SESN3, CYS1, MIR182, MIR27B, MIR320A, MIR548B, MIR548C, MIR1305, MIR3191, MIR548Z, MIR4482, MIR4491
WIKIPATHWAYS	miRNA regulation of prostate cancer signaling pathways	KLK3, AR, BAD, CCND1, BCL2, CASP9, CDKN1A, CDKN1B, CREBBP, CTNNB1, FOXO1, MTOR, GRB2, GSK3B, KRAS, MDM2, NFKB1, NFKBIA, PDGFA, PDGFRB, PIK3CA, MAPK1, MAP2K1, MAP2K2, RAF1, SOS1, TCF7, TP53, IKBKG, AKT3, PLCL2, NFATC2IP, CREB3L1, MIRLET7BHG, MIRLET7A3, MIRLET7B, MIR106A, MIR19B2, MIR200A, MIR30D, MIR92A2, MIR337, MIR363, MIR20B, MIR18B, MIR589, MIR603, MIR1299, MIR3120, MIR4325, MIR4311, MIR3149, MIR466, MIR3664, MIR3682, MIR3714, MIR4763, MIR4664, MIR4517

Predefined gene sets in Hepamine

Data source	Pathways	Symbols
WIKIPATHWAYS	miRNAs in the signaling pathway of the immune response in sepsis	CHUK, MAPK14, ELANE, GZMB, ICAM1, IKBKB, IL1A, CXCL8, IRAK1, IRF1, IRF5, IRF7, LCN2, MYD88, NFKB1, NFKB2, NFKBIA, MAPK8, REL, RELA, RELB, CCL3, CCL4, MAP3K7, TLR4, TNF, TRAF3, TRAF6, VCAM1, IKBKG, TAB1, TAB2, IRAK4, TLR7, TLR8, MIR155HG, MIRLET7E, MIRLET7I, MIR126, MIR149, MIR155, MIR16-1, MIR16-2, MIR199A1, MIR199A2, MIR200B, MIR200C, MIR203A, MIR223, MIR9-1, MIR146B, MIR758, MIR718, MIR203B
WIKIPATHWAYS	miRNAs involved in DNA damage response	ABL1, ATM, CCND1, CCND3, CCNE1, CDC25A, CDK6, CDKN1A, CDKN1B, CREB1, E2F1, H2AFX, MYC, RAD52, TP53, MIRLET7A1, MIRLET7D, MIRLET7F1, MIR100, MIR106B, MIR145, MIR15B, MIR16-1, MIR181B1, MIR203A, MIR210, MIR181A1, MIR221, MIR222, MIR23A, MIR23B, MIR25, MIR27A, MIR27B, MIR34B, MIR34C, MIR93, MIR371A, MIR372, MIR373, MIR449A, MIR450A2, MIR503, MIR542, MIR421, MIR449B, MIR450B, MIR374B, MIR449C, MIR3074
WIKIPATHWAYS	miRNA targets in ECM and membrane receptors	COL1A2, COL3A1, COL4A1, COL4A2, COL5A1, COL5A2, COL6A1, COL6A2, COL6A3, FN1, ITGA1, ITGB5, ITGB6, LAMA4, LAMB2, LAMC1, SDC2, THBS1, THBS2, TNXB, ITGA11, COL5A3, MIR107, MIR141, MIR15B, MIR200A, MIR200B, MIR200C, MIR219A1, MIR25, MIR30B, MIR30C1, MIR30C2, MIR30D, MIR30E, MIR148B, MIR429, MIR532, MIR548D1, MIR589, MIR3606, MIR548AA1, LINC02478
WIKIPATHWAYS	miRs in Muscle Cell Differentiation	EZH2, ID2, MEF2A, MEF2C, MEF2D, MYF5, MYOD1, PAX7, PRKACA, PRKACB, PRKACG, PRKAR1A, PRKAR1B, PRKAR2A, PRKAR2B, PRKCA, PRKCB, PRKCD, PRKCE, PRKCG, PRKCH, PRKCI, PRKD1, PRKCQ, PRKCZ, SRF, PRKD3, ELSPBP1, MIR1-1, MIR206, MIR221, MIR222, MIR26A1, MIR26A2, MIR133B, MEF2B
WIKIPATHWAYS	Mismatch repair	MSH6, LIG1, MLH1, MSH2, PCNA, POLD1, RFC1, RPA1, EXO1

Predefined gene sets in Hepamine

Data source	Pathways	Symbols
WIKIPATHWAYS	Mitochondrial complex I assembly model OXPHOS system	ND1, ND2, ND4, ND4L, ND5, ND6, NDUFA1, NDUFA2, NDUFA3, NDUFA5, NDUFA6, NDUFA7, NDUFA8, NDUFA10, NDUFAB1, NDUFB1, NDUFB2, NDUFB3, NDUFB4, NDUFB5, NDUFB6, NDUFB7, NDUFB8, NDUFB9, NDUFB10, NDUFC1, NDUFC2, NDUFS1, NDUFS2, NDUFS3, NDUFV1, NDUFS4, NDUFS5, NDUFS6, NDUFV2, NDUFV3, TMEM186, NDUFAF3, ACAD9, NDUFAF4, NDUFA13, NDUFAF1, ECSIT, TIMMDC1, NDUFB11, TMEM70, DMAC2, NDUFAF7, FOXRED1, COA1, TMEM126B, NDUFA12, NUBPL, DMAC1, NDUFAF2, NDUFAF6
WIKIPATHWAYS	Mitochondrial Gene Expression	CAMK4, CREB1, ESRRA, GABPA, GABPB1, HCFC1, NRF1, POLRMT, PPP3CA, SP1, TFAM, MTERF1, PPARGC1A, PPRC1, MYEF2, MTERF3, TFB1M, GABPB1-IT1, TFB2M, PPARGC1B
WIKIPATHWAYS	Mitochondrial LC-Fatty Acid Beta-Oxidation	ACADL, ACADM, ACADS, ACADVL, SLC25A20, CPT1A, CPT2, ECI1, EHHADH, ACSL1, ACSL3, ACSL4, HADHA, HADH, SCP2, PECR, ACSF2
WIKIPATHWAYS	Monoamine GPCRs	ADRA1D, ADRA1B, ADRA1A, ADRA2A, ADRA2B, ADRA2C, ADRB1, ADRB2, ADRB3, CHRM1, CHRM2, CHRM3, CHRM4, CHRM5, DRD1, DRD2, DRD3, DRD4, DRD5, HRH1, HRH2, HTR1A, HTR1B, HTR1D, HTR1E, HTR1F, HTR2A, HTR2B, HTR2C, HTR4, HTR5A, HTR6, HTR7
WIKIPATHWAYS	Monoamine Transport	ACHE, ADORA2A, AGT, AMPH, CDC25C, MAPK14, DBH, IL1B, IL1R1, ITGB3, NOS1, TNFRSF11B, PPP2CB, NECTIN2, RBL2, SLC6A1, SLC6A2, SLC6A3, SLC6A4, STX1A, SYN1, TDO2, TGFB1I1, TH, TNF, TSC2, SCAMP2, UNC13B, HRH3, SLC5A7, FBXO32, TPH2

Predefined gene sets in Hepamine

Data source	Pathways	Symbols
WIKIPATHWAYS	mRNA Processing	CLK1, CLK2, CLK3, CSTF1, CSTF2, CSTF3, DDX1, DHX8, DHX9, DHX15, FUS, HNRNPA1, HNRNPA2B1, HNRNPAB, HNRNPC, HNRNPD, HNRNPH1, HNRNPH2, HNRNPK, HNRNPL, HNRNPU, PRMT2, PRMT1, HNRNPM, NCBP1, SNU13, NONO, YBX1, PCBP2, POLR2A, PPM1G, PSKH1, PTBP1, RNU2-1, SFPQ, SRSF1, SRSF2, SRSF3, SRSF4, SRSF5, SRSF6, SRSF7, SFSWAP, TRA2B, SNRNP70, SNRPA, SNRPA1, SNRPB, SNRPB2, SNRPD1, SNRPD2, SNRPD3, SNRPE, SNRPF, SNRPG, SNRPN, SRP54, SRPK1, SRPK2, SUPT5H, U2AF1, PABPN1, SF3A2, SMC1A, SPOP, DHX16, PRPF18, SRSF9, RNMT, RNGTT, PRPF4B, PRPF4, PRPF3, EFTUD2, SNRNP40, RBM39, DHX38, SUGP2, RBM5, HNRNPR, SRRM1, SF3B4, SF3A1, CD2BP2, NXF1, PRPF8, CELF1, CELF2, SRSF10, CPSF4, TXNL4A, PAPOLA, RNPS1, SF3A3, TMED10, CLP1, SF3B2, NUDT21, CLASRP, DDX20, U2AF2, XRN2, DNAJC8, NCBP2, CSTF2T, DICER1, SF3B3, SF3B1, PRPF6, RBMX, CPSF1, CDC40, LSM7, CPSF3, CPSF2, PRPF40A, METTL3, CELF4, CLK4, SUGP1, LSM2, PTBP2, SF3B5, PHF5A, RBM17, SREK1, MIR7-1, MIR636, MIR939, MIR1234, MIR4745, MIR6849, U2AF1L5
WIKIPATHWAYS	MTHFR deficiency	ASMT, ALDH7A1, BHMT, CASP3, CASP9, CHKA, COMT, DNMT1, DNMT3A, DNMT3B, GRIN1, GRIN2A, GRIN2D, HNMT, MARS, MTHFR, PCYT1A, PEMT, EHMT2, CYCS, CHDH, NDUFAF7, CHPT1, EHMT1, SGMS1, MIR4761, MIR6758

Predefined gene sets in Hepamine

Data source	Pathways	Symbols
WIKIPATHWAYS	Myometrial Relaxation and Contraction Pathways	ACTA1, ACTA2, ACTB, ACTC1, ACTG1, ADCY1, ADCY2, ADCY3, ADCY5, ADCY6, ADCY7, ADCY8, ADCY9, ADM, ARRB1, ARRB2, ATF1, ATF3, ATF4, ATP2A2, ATP2A3, CACNB3, CALCA, CALD1, CALM1, CALM2, CALM3, CAMK2A, CAMK2B, CAMK2D, CAMK2G, CNN1, CNN2, CREB1, ATF2, ATF6B, CRH, CRHR1, LPAR1, ETS2, FOS, GABPA, GABPB1, GJA1, GNAQ, GNAS, GNB1, GNB2, GNB3, GNG3, GNG4, GNG5, GNG7, GNG11, GNGT1, SFN, GRK4, GRK5, GRK6, GUCA2A, GUCA2B, GUCY1A1, IGFBP1, IGFBP2, IGFBP3, IGFBP4, IGFBP5, IGFBP6, IL1B, IL6, ITPR1, ITPR2, ITPR3, JUN, MYL2, MYL4, NFKB1, NOS1, NOS3, OXT, OXTR, PDE4B, PDE4D, PLCB3, PLCD1, PLCG1, PLCG2, PRKACA, PRKACB, PKIA, PKIB, PRKAR1A, PRKAR1B, PRKAR2A, PRKAR2B, PRKCA, PRKCB, PRKCD, PRKCE, PRKCG, PRKCH, PRKD1, PRKCQ, PRKCZ, RGS1, RGS2, RGS3, RGS4, RGS7, RGS10, RGS16, RLN1, RYR1, RYR2, RYR3, SLC8A1, SP1, YWHAB, YWHAE, YWHAG, YWHAH, YWHAZ, RGS5, DGKZ, RGS20, RGS11, RGS9, GSTO1, RGS6, RAMP2, RAMP1, RAMP3, RGS19, CREB3, RGS14, GNB5, CORIN, YWHAQ, PKIG, GPR182, ATF5, MAFF, RGS17, CRCP, GNG13, GNG2, GABPB1-IT1, GNG12, ACKR3, GNB4, RXFP1, RGS18, MYLK2, GNG8, RXFP2, ADCY4, MIR4751, LINC02210-CRHR1
WIKIPATHWAYS	NAD Biosynthesis II (from tryptophan)	TDO2, KMO, KYNU, QPRT, HAAO, NADSYN1, NMNAT1, AFMID
WIKIPATHWAYS	NAD+ biosynthetic pathways	PARP1, PARP4, BST1, CD38, IDO1, TDO2, TNKS, PARP2, NAMPT, SIRT2, SIRT5, SIRT4, SIRT3, SIRT1, QPRT, SIRT7, SIRT6, NADSYN1, NMNAT1, TNKS2, NAPRT, ACMSD
WIKIPATHWAYS	NAD+ metabolism	PARP1, CD38, NT5E, NAMPT, SIRT2, NMNAT2, SIRT5, SIRT4, SIRT3, SIRT1, SIRT7, SIRT6, NMNAT1, NADK, NRK, NMNAT3
WIKIPATHWAYS	NAD metabolism, sirtuins and aging	PARP1, FOXO1, FOXO3, HIF1A, NFKB1, PPARG, ROS1, TFAM, NAMPT, SIRT3, SIRT1

Predefined gene sets in Hepamine

Data source	Pathways	Symbols
WIKIPATHWAYS	Nanomaterial induced apoptosis	APAF1, FAS, FASLG, BAK1, BAX, BCL2, BID, CASP3, CASP6, CASP7, CASP8, CASP9, ENDOG, PRF1, FADD, CFLAR, AIFM1, HTRA2, CYCS, DIABLO
WIKIPATHWAYS	Nanoparticle-mediated activation of receptor signaling	AREG, COL1A1, MAPK14, EGFR, FN1, GRB2, HRAS, ITGA1, ITGB1, KRAS, NRAS, PIK3CD, MAPK1, MAPK8, MAPK11, MAPK9, MAPK10, MAPK13, MAP2K1, MAP2K2, PTK2, PXN, RAF1, MAPK12, SOS1, SRC, TLN1, AKT3, MIR6852
WIKIPATHWAYS	Nanoparticle triggered autophagic cell death	BCL2, INS, INSR, TSC1, TSC2, UVRAG, ULK1, BECN1, ATG12, ATG5, ULK2, CHAF1A, ATG7, ATG14, SH3GLB1, ATG16L1, AMBRA1, ATG3, VMP1, ATG10, MAP1LC3A, ATG4A, ATG9B, MIR21
WIKIPATHWAYS	Nanoparticle triggered regulated necrosis	PARP1, CASP8, FTL, PLA2G4A, MAPK8, TNFRSF1A, TRADD, RIPK1, FADD, RIPK3, TICAM1
WIKIPATHWAYS	ncRNAs involved in STAT3 signaling in hepatocellular carcinoma	IL6, IL6R, IL6ST, IL11, IL11RA, JAK1, JAK2, JAK3, NFKB1, RELA, SOX4, STAT3, ZEB1, MIR200A, MIR200B, MIR200C, UCA1
WIKIPATHWAYS	ncRNAs involved in Wnt signaling in hepatocellular carcinoma	APC, CCND1, CCND2, CCND3, CSNK1A1, CSNK1E, CSNK2A1, CSNK2A2, CSNK2B, CTBP1, CTBP2, CTNNB1, DVL1, DVL2, DVL3, ELAVL1, EZH2, FZD2, GSK3B, JUN, LRP6, LRP5, MYC, ROR1, ROR2, SERPINF1, PLAU, RYK, SFRP1, SFRP2, SFRP4, SFRP5, TCF7, TCF7L2, WNT1, WNT2, WNT3, WNT5A, WNT6, WNT7A, WNT7B, WNT10B, WNT11, WNT2B, FZD5, FZD3, FOSL1, AXIN1, FZD1, FZD6, FZD7, FZD8, FZD9, CER1, FRAT1, WIF1, FZD10, DKK1, FRAT2, DKK4, DKK2, SOST, LEF1, WNT16, NLK, WNT4, CTNNBIP1, DANCR, CHD8, SENP2, SOX17, PORCN, CXXC4, WNT10A, WNT5B, TCF7L1, SOX7, KREMEN1, NKD1, NKD2, WNT3A, CSNK1A1L, NOTUM, CSNK2A3, MIR4683, LOC101929777, MIR6808, TPTEP2-CSNK1E

Predefined gene sets in Hepamine

**Data source**	**Pathways**	**Symbols**
WIKIPATHWAYS	Neural Crest Differentiation	RHOB, ASCL1, BMP4, BMP7, CDH1, CDH2, CDH6, CDH7, COL2A1, COL11A2, CTBP2, CTNNB1, DCT, DLX5, DVL1, DVL2, DVL3, ETS1, FGF2, FGF8, FGFR1, FGFR3, FGFR2, GBX2, GFAP, GJB1, GSK3B, HDAC1, HDAC2, TLX2, HOXA1, HOXB1, HES1, ID1, RBPJ, ISL1, ITGB1, LHX1, SMAD1, MBP, MITF, MPZ, MSX1, MSX2, MYB, MYC, NEUROG1, NFKB1, NFKB2, NOTCH1, NOTCH2, NOTCH3, NOTCH4, PAX3, PAX7, PMP22, SNAI2, SNAI1, SOX5, SOX9, SOX10, TBX6, TCF4, TFAP2A, TFAP2B, TWIST1, WNT1, WNT8A, ZIC1, FZD3, MIA, AXIN1, AXIN2, HDAC3, PHOX2B, LHX2, HAND1, HDAC9, HDAC4, FGF19, HDAC6, HDAC5, OLIG2, DLL3, HEY2, FOXD3, DLL1, HDAC7, DLL4, HDAC8, LHX5, HDAC11, TCF7L1, HDAC10, ZIC5, WNT3A, OLIG1, DMBX1, OLIG3, PRTG, HES5, MIR6808
WIKIPATHWAYS	Neurotransmitter Disorders	DBH, DDC, MAOA, TH
WIKIPATHWAYS	Nicotine Activity on Chromaffin Cells	CACNA1C, CHRNA3, CHRNB4, CACNA1G
WIKIPATHWAYS	Nicotine Activity on Dopaminergic Neurons	ADCY2, CDK5, CHRNA3, CHRNA4, CHRNA5, CHRNB2, DDC, DRD2, DRD3, DRD4, GNAI1, GNB1, KCNK3, PPP1CA, PRKACA, SLC18A2, TH, CHRNA6, KCNK9, GNG2, PPP1R1B, LOC102723788
WIKIPATHWAYS	Nicotine Metabolism	AOX1, CYP2A6, CYP2B6, FMO3, UGT1A9, UGT1A4
WIKIPATHWAYS	NLR Proteins	CD40, CHUK, EPHB2, IKBKB, MAPK8, RELA, MAP3K7, IKBKG, ERBIN
WIKIPATHWAYS	NO/cGMP/PKG mediated Neuroprotection	ACTN2, XIAP, BAD, BCL2, TSPO, CALM1, CALM2, CAMK2A, CAMK2B, CAMK2D, CAMK2G, CASP9, CNGB1, CNGA1, CNGA2, CNGA3, CNGA4, CREB1, DLG4, GRIN1, GRIN2A, GRIN2B, GRIN2C, GRIN2D, GUCY1B2, GUCY1A2, GUCY1A1, GUCY1B1, IFNG, IL1B, NEFL, NFKB1, NFKBIA, NOS1, NOS2, NOS3, NPPA, NPPB, NPR1, PDE2A, PDE3A, PPID, PRKG2, RELA, TNF, AKAP9, CYCS, CNGB3

Predefined gene sets in Hepamine

Data source	Pathways	Symbols
WIKIPATHWAYS	Non-genomic actions of 1,25 dihydroxyvitamin D3	CAMK2A, CAMK2B, CAMK2D, CAMK2G, CAMP, CAV1, CD40, CD40LG, MAPK14, CYP24A1, CYP27B1, DEFB4A, ETS1, HRAS, IFNA2, IFNG, IKBKB, IL6, CXCL8, JAK1, JUN, KRAS, NFKB1, NFKB2, NRAS, OAS2, PLCB2, PLCB3, PLCB4, PLCD1, PLCG2, MED1, PRKCA, PRKCB, PRKCD, PRKCE, PRKCG, PRKCH, PRKCQ, PRKCZ, MAPK1, MAPK3, MAPK7, MAPK8, MAPK11, MAPK9, MAPK13, RELB, RXRA, RXRB, RXRG, MAPK12, CCL2, SP1, SP3, STAT1, STAT2, TLR2, TLR4, TNF, TNFRSF1A, TYK2, VDR, ISG15, IFI44L, PLCB1, PLCE1, TLR8, NOD2, IFI27L2, RSAD2
WIKIPATHWAYS	Non-homologous end joining	XRCC6, MRE11, PRKDC, XRCC4, XRCC5, RAD50, NHEJ1
WIKIPATHWAYS	Non-small cell lung cancer	AKT1, AKT2, ALK, ARAF, BAD, BAK1, BAX, CCND1, BRAF, CASP9, CDK4, CDK6, CDKN1A, CDKN2A, DDB2, GADD45A, E2F1, E2F2, E2F3, EGF, EGFR, ERBB2, FHIT, FOXO3, GRB2, HRAS, JAK3, KRAS, GADD45B, NRAS, PDK1, PIK3CA, PIK3CB, PIK3CD, PIK3R1, PIK3R2, PLCG1, PLCG2, PRKCA, PRKCB, PRKCG, MAPK1, MAPK3, MAP2K1, MAP2K2, RAF1, RARB, RB1, RXRA, RXRB, RXRG, SOS1, SOS2, STAT3, STAT5A, STAT5B, STK4, TGFA, TP53, PIK3R3, AKT3, GADD45G, RASSF1, EML4, POLK, RASSF5
WIKIPATHWAYS	NOTCH1 regulation of human endothelial cell calcification	JAG1, ALPL, CALU, LPAR1, FGFR3, GJA5, ITGA1, JAG2, MGP, NOTCH1, PLAT, SAT1, VEGFA, DLL3, DLL1, DLL4, SOX6, MIR6073

Predefined gene sets in Hepamine

Data source	Pathways	Symbols
WIKIPATHWAYS	Notch Signaling Pathway	JAG1, AKT1, CCND1, CDKN1A, DTX1, EP300, FHL1, GATA3, GSK3B, HDAC1, HDAC2, HIF1A, HES1, RBPJ, JAG2, JAK2, LCK, MAGEA1, MAPT, MYC, NFKB1, NOTCH1, NOTCH2, NOTCH3, NOTCH4, PIK3R1, PIK3R2, PSEN1, PSEN2, RING1, SKP1, SRC, STAT3, ADAM17, TLE1, CUL1, NUMB, SAP30, NUMBL, CIR1, NCOR1, NCOR2, MAML1, DLL3, SNW1, SPEN, NCSTN, HEY1, HEY2, DLL1, APH1A, DLL4, FBXW7, HES6, MAML3, PSENEN, APH1B, ITCH, MAML2, PTCRA, HES5, MIR1281, LOC101928143, JAG1, CREBBP, CTBP1, CTBP2, DTX1, DVL1, DVL2, DVL3, KAT2A, HDAC1, HDAC2, HES1, RBPJ, JAG2, KCNJ5, LFNG, MFNG, NOTCH1, NOTCH2, NOTCH3, NOTCH4, PSEN1, PSEN2, RFNG, ADAM17, NUMB, KAT2B, NUMBL, NCOR2, MAML1, DLL3, RBPJL, DTX4, NCSTN, DLL1, APH1A, INPP5K, DLL4, MAML3, APH1B, DTX2, DTX3L, PTCRA, DTX3, HES5, LOC101928143, MIR6808
WIKIPATHWAYS	NRF2-ARE regulation	CEBPB, NQO1, EPHB2, FYN, GCLC, GCLM, GSK3B, GSTA2, HMOX1, INSR, MAF, NFE2L2, PIK3CA, PRKCA, MAPK8, SRC, YES1, AIMP2, CUL3, KEAP1, RBX1, SLC7A11, PGAM5

Predefined gene sets in Hepamine

Data source	Pathways	Symbols
WIKIPATHWAYS	NRF2 pathway	ADH7, AGER, ALDH3A1, BLVRB, CBR1, CBR3, CES1, ABCC2, CYP2A6, CYP4A11, NQO1, HBEGF, EGR1, EPHA2, EPHA3, FGF13, FTH1, FTL, G6PD, GGT1, GCLC, GCLM, GPX2, GPX3, GSR, GSTA1, GSTA2, GSTA3, GSTA4, GSTM1, GSTM2, GSTM3, GSTM4, GSTM5, GSTP1, GSTT2, HGF, NRG1, HMOX1, HSPA1A, HSP90AA1, HSP90AB1, DNAJB1, MAFG, ME1, MGST2, MGST3, NFE2L2, PRDX1, PDGFB, PGD, SERPINA1, PPARD, RXRA, SLC2A1, SLC2A2, SLC2A3, SLC2A4, SLC2A5, SLC5A1, SLC5A2, SLC5A3, SLC5A4, SLC5A5, SLC6A1, SLC6A2, SLC6A3, SLC6A4, SLC6A6, SLC6A7, SLC6A8, SLC6A9, SLC6A11, SLC6A13, SOD3, TGFA, TGFB1, TGFB2, TGFBR2, TXN, TXNRD1, UGT2B7, SLC39A7, ABCC3, CES2, SQSTM1, SLC5A6, SLC6A5, PRDX6, KEAP1, ABCC5, ABCC4, SLC2A6, SLC6A14, PTGR1, CES3, SLC39A14, SLC7A11, MAFF, SLC39A6, SLC39A1, SLC6A16, SLC39A3, SLC39A2, SLC2A8, UGT1A7, UGT1A6, UGT1A9, UGT1A4, UGT1A1, UGT1A3, SLC6A20, SLC6A15, SLC39A9, SLC39A4, SLC2A9, SLC39A10, SLC5A7, SLC39A8, SLC2A11, SLC2A10, GGTLC2, SLC39A13, GGTLC1, TXNRD3, SLC2A13, SLC5A11, SLC5A10, SLC2A14, SLC2A12, SLC2A7, SLC5A12, SLC5A8, SLC5A9, SLC39A11, SLC39A12, CES5A, GSTA5, SLC39A5, CES4A, SLC6A19, SLC6A18, SLC6A17, GGT2, LOC102724197
WIKIPATHWAYS	Nuclear Receptors	NR0B1, AR, NR2F6, ESR1, ESR2, ESRRA, ESRRB, NR5A2, NR5A1, NR3C1, NR4A1, HNF4A, ROR1, NR4A2, PGR, PPARA, PPARD, PPARG, RARA, RARB, RARG, RORA, RORC, RXRA, RXRB, RXRG, NR2F1, NR2F2, THRA, THRB, NR2E1, NR2C2, NR1H2, VDR, NR1I2, NR1I3, NR1D2, NR1H3, MIR1469
WIKIPATHWAYS	Nuclear Receptors in Lipid Metabolism and Toxicity	ABCA1, ABCD2, ABCC2, CYP1A2, CYP2B6, CYP2C9, CYP2E1, CYP3A4, CYP4A11, CYP4B1, CYP7A1, CYP8B1, CYP24A1, CYP26A1, CYP27B1, ABCB1, ABCB4, PPARA, PPARD, PPARG, ABCD3, RARA, RARB, RARG, VDR, ABCB11, ABCC3, NR1I2, ABCG1, NR1I3, NR1H4, NR1H3, ABCG5, MIR33A, MIR33B

Data source	Pathways	Symbols
WIKIPATHWAYS	Nuclear Receptors Meta-Pathway	ACAA1, ACADM, ACOX1, ADH7, AGER, AHR, ALAS1, ALDH3A1, ALOX5AP, BIRC2, BIRC3, APOA1, APOA2, APOC3, ARNT, BAAT, BAX, CCND1, BLVRB, CBR1, CBR3, CDK1, CDK4, CDKN1B, CDKN1C, CES1, ABCC2, CPT1A, CPT2, CYP1A1, CYP1A2, CYP1B1, CYP2A6, CYP3A7, CYP2B6, CYP2C19, CYP2C9, CYP3A4, CYP3A5, CYP4A11, CYP7A1, CYP8B1, DBI, NQO1, TSC22D3, SLC26A2, HBEGF, EDN2, EGFR, EGR1, EHHADH, EPHA2, EP300, STOM, EPHA3, ESR1, FABP1, FASN, FGF13, FKBP5, FOXO1, FTH1, FTL, G6PD, GGT1, GCLC, GCLM, GPX2, GPX3, NR3C1, GSR, GSTA1, GSTA2, GSTA3, GSTA4, GSTM1, GSTM2, GSTM3, GSTM4, GSTM5, GSTP1, GSTT2, HGF, NRG1, HMOX1, HES1, HSPA1A, HSP90AA1, HSP90AB1, DNAJB1, IFNG, IGFBP1, IL1B, IL2, IL11, IL12A, IL12B, JUN, JUNB, JUND, KTN1, MAFG, ME1, MFGE8, MGST1, MGST2, MGST3, MYC, GADD45B, NFE2L2, NFKB2, PRDX1, SERPINB2, PCK1, PDE4B, PDGFB, PDK4, PGD, ABCB1, ABCB4, SERPINA1, SERPINB9, PLTP, PMP2, POU5F1, PPARA, PPARD, PTPA, SRGN, PSMC5, PTGS2, RGS2, RXRA, S100P, SCD, SCNN1A, SCP2, CCL2, CCL20, SEC14L1, SLC2A1, SLC2A2, SLC2A3, SLC2A4, SLC2A5, SLC5A1, SLC5A2, SLC5A3, SLC5A4, SLC5A5, SLC6A1, SLC6A2, SLC6A3, SLC6A4, SLC6A6, SLC6A7, SLC6A8, SLC6A9, SLC6A11, SLC6A13, SLC10A1, SNAI2, SMARCA1, SOD3, SP1, SRC, SREBF1, STAT3, SULT1A1, SULT2A1, TGFA, TGFB1, TGFB2, TGFBR2, TGFBR3, THBD, TNF, TNFAIP3, DNAJC7, TXN, TXNRD1, UGT2B4, UGT2B7, VDR, ZIC2, SLC39A7, SLC7A5, NCOA3, NRIP1, SMC1A, NR0B2, CAVIN2, CUL1, ENC1, BHLHE40, ABCB11, NCOA1, IRS2, ABCC3, CES2, KAT2B, NR1I2, SQSTM1, SLC5A6, AIP, SLC6A5, PRDX6, SERTAD2, KEAP1, FGF19, NR1I3, NR1H4, FGFBP1, ABCC5, NR1H3, SPRY1, ABCC4, CAP2, NCOA2, SLC19A2, SLCO1B1, CDC42EP3, PTGES3, PLK2, PPARGC1A, SLC27A5, ABHD2, CDC37, SLC2A6, AKAP13, SLC6A14, SLCO2B1, PTGR1, NCOA6, CES3, SLC39A14, SLC7A11, MAFF, SLC39A6, MYOF, ANKRD1, SLC39A1, IL17B, SRPX2, SLC6A16, DNAJC15, SLC39A3, SLC39A2, SLC2A8, ANGPTL4, POLK, EPB41L4B, UGT1A7, UGT1A6, UGT1A9, UGT1A4, UGT1A1, UGT1A3, SLC6A20, SLC6A15, ETNK2, SLC39A9

Predefined gene sets in Hepamine

Data source	Pathways	Symbols
WIKIPATHWAYS	Nucleotide-binding Oligomerization Domain (NOD) pathway	AAMP, XIAP, PRDM1, CASP1, CASP5, CASP7, CASP8, CASP9, CHUK, HSP90AA1, IKBKB, IL1B, IL18, MEFV, NAIP, NFKBIA, RAC1, RELA, IKBKG, RIPK2, AIM2, ACAP1, NOD1, SUGT1, COPS6, NLRP1, CARD8, PYCARD, DUOX2, NDUFA13, NLRP2, ERBIN, NLRC4, NOD2, CARD9, CARD6, NLRP12, NLRP3, NLRP4, NLRP7, NLRP10
WIKIPATHWAYS	Nucleotide GPCRs	ADORA1, ADORA2A, ADORA2B, ADORA3, LTB4R, LPAR4, P2RY1, P2RY2, P2RY4, P2RY6, LPAR6
WIKIPATHWAYS	Nucleotide Metabolism	ADSL, ADSS, DHFR, HPRT1, IMPDH1, NME2, OAZ1, POLA1, POLB, POLD1, POLG, PRPS1, PRPS2, RRM1, RRM2, SAT1, SRM, MTHFD2, RRM2B
WIKIPATHWAYS	Oligodendrocyte Specification and differentiation(including remyelination), leading to Myelin Components for CNS	ASCL1, BMP2, BMP4, CNP, CNTF, FGF2, GLI2, CXCL1, CXCL2, IGF1, IL1B, LIF, MAG, MBP, MOG, MYT1, NKX2-2, OMG, PDGFB, PLP1, SHH, SOX5, SOX9, SOX10, TNF, OLIG2, SOX8, SOX6, OLIG1, NKX2-6, MIR6073
WIKIPATHWAYS	Oncostatin M Signaling Pathway	AKT1, CASP3, CASP7, CDK2, CDKN1B, CEBPB, CREB1, MAPK14, EGR1, PTK2B, FOS, MTOR, GRB2, HIF1A, HRAS, CCN1, IL6ST, IRS1, JAK1, JAK2, JAK3, JUNB, JUND, KRAS, LDLR, LIFR, MMP1, MMP3, MMP13, NFKB1, NFKBIA, OSM, SERPINE1, PIK3R1, PRKCA, PRKCB, PRKCD, PRKCE, PRKCH, MAPK1, MAPK3, MAPK8, MAPK9, MAP2K1, MAP2K2, PTPN11, PXN, RAF1, RELA, RPS6, CCL2, SHC1, SOS1, SRC, STAT1, STAT3, STAT5B, TIMP3, TP53, TYK2, VEGFA, SOCS3, OSMR, PIAS3, RICTOR, MIR6886
WIKIPATHWAYS	One Carbon Metabolism	AHCY, AMT, ATIC, BHMT, DHFR, DNMT1, DNMT3A, DNMT3B, FOLH1, GART, GLRX, MAT1A, MTHFD1, MTHFR, MTR, MTRR, SHMT1, SHMT2, TCN2, TYMS, MTHFS, MTHFD2, ALDH1L1, FTCD, AHCYL2, MTHFD1L, MAT2B, MTFMT, FOLH1B, MIR6778

Predefined gene sets in Hepamine

Data source	Pathways	Symbols
WIKIPATHWAYS	One carbon metabolism and related pathways	BAAT, BCAT1, BCAT2, BHMT, CDO1, CHKA, CHKB, CTH, SARDH, DNM1, DNMT3A, GAD1, GAD2, GCLC, GCLM, GPX1, GPX2, GPX3, GPX4, GPX5, GPX7, GSR, GSS, MAT1A, MAT2A, MTHFR, MTR, PCYT1A, PLD1, PCYT2, SHMT1, SHMT2, SOD1, SOD2, SOD3, TYMS, PCYT1B, CEPT1, PEMT, AHCYL1, BHMT2, GNMT, DMGDH, CSAD, ETNK2, CHDH, ETNK1, CHPT1, AGXT2, DHFR2, GPX6, SOD2-OT1, MIR6778, CBSL
WIKIPATHWAYS	Osteoblast Signaling	BGLAP, COL1A1, IBSP, ITGAV, ITGB3, TNFRSF11B, PDGFB, PDGFRA, PDGFRB, PTH, PTH1R, FGF23, TNFSF11, SLC17A2
WIKIPATHWAYS	Osteoclast Signaling	ACP5, CTSK, IFNAR1, IFNB1, ITGB3, TNFRSF11B, PDGFB, MAPK8, SLC9A1, SPP1, AIMP2, GPR68, TNFSF11, TNFRSF11A, ATP6V1G1, TRPV5
WIKIPATHWAYS	Osteopontin Signaling	CHUK, IKBKB, ITGAV, ITGB3, MMP9, NFKB1, PLAU, MAPK1, MAPK3, MAP2K1, RELA, SPP1, MAP3K14
WIKIPATHWAYS	Ovarian Infertility Genes	ATM, BMPR1B, CCND2, CDK4, CDKN1B, CEBPB, CYP19A1, DAZL, EGR1, ESR2, FSHR, NR5A1, GDF9, GJA4, INHA, LHCGR, SMAD3, MLH1, MSH5, PGR, PRLR, PTGER2, SMPD1, TBP, VDR, ZP2, ZP3, NRIP1, NCOR1, DMC1, SYNE2, FIGLA
WIKIPATHWAYS	Overview of nanoparticle effects	BAX, BCL2, CCND3, CDH3, COL4A1, CRP, FN1, HMOX1, IL6, CXCL8, ITGAD, LAMA3, NFRKB, PIK3CD, PTGS1, PTGS2, PTK2, TNF, AKT3
WIKIPATHWAYS	Oxidation by Cytochrome P450	CYB5A, CYP1A1, CYP1A2, CYP1B1, CYP2A6, CYP2A7, CYP3A7, CYP2A13, CYP2B6, CYP2C19, CYP2C8, CYP2C9, CYP2C18, CYP2D6, CYP2E1, CYP2F1, CYP2J2, CYP3A4, CYP3A5, CYP4A11, CYP4B1, CYP7A1, CYP8B1, CYP11A1, CYP11B1, CYP11B2, CYP17A1, CYP19A1, CYP24A1, CYP26A1, CYP27A1, CYP27B1, CYP51A1, CYB5R3, CYP4F3, POR, CYP4F2, CYP7B1, CYP46A1, CYP4F8, CYP2G1P, CYP2S1, CYB5R4, CYP39A1, CYB5R2, CYB5R1, CYP2W1, CYP26B1, CYP20A1, CYP4F11, CYP3A43, CYP4F12, CYB5B, CYP2U1, CYP2R1, CYP4F22, CYP4Z1, CYP4X1, CYP4A22, CYP4V2, CYP27C1, CYP26C1, MIR4651

Predefined gene sets in Hepamine

Data source	Pathways	Symbols
WIKIPATHWAYS	Oxidative Damage	APAF1, BAD, BAK1, BCL2, C1QA, C1QB, C1QC, C1R, C1S, C2, C3AR1, C4B, C5, C5AR1, CASP3, CASP9, CDC42, CDKN1A, CDKN1B, CDKN1C, CR2, GADD45A, MAP3K1, MAP3K9, NFKB1, NFKBIE, PCNA, MAPK10, MAPK13, MAP2K4, TNF, TNFRSF1B, TRAF1, TRAF2, TRAF3, TRAF6, BAG4, TNK2, TDP2, CYCS, MIR7846
WIKIPATHWAYS	Oxidative phosphorylation	ATP5F1A, ATP5F1B, ATP5F1D, ATP5F1E, ATP5PB, ATP5MC1, ATP5MC2, ATP5MC3, ATP5ME, ATP5PF, ATP6AP1, ATP5PO, GZMB, ATP6, ND1, ND2, ND3, ND4, ND4L, ND5, ND6, NDUFA2, NDUFA3, NDUFA4, NDUFA5, NDUFA6, NDUFA7, NDUFA8, NDUFA9, NDUFA10, NDUFAB1, NDUFB1, NDUFB2, NDUFB4, NDUFB5, NDUFB6, NDUFB7, NDUFB8, NDUFB9, NDUFB10, NDUFC1, NDUFC2, NDUFS1, NDUFS2, NDUFS3, NDUFV1, NDUFS4, NDUFS5, NDUFS6, NDUFS8, NDUFV2, NDUFV3, ATP5MF, ATP6AP2, ATP5PD, ATP5MG, DMAC2L, NDUFA4L2, NDUFA11, NDUFS7, MIR4691, MIR7113
WIKIPATHWAYS	Oxidative Stress	CAT, MAPK14, CYP1A1, NQO1, FOS, GCLC, GPX1, GPX3, GSR, GSTT2, HMOX1, JUNB, MAOA, MGST1, MT1X, NFE2L2, NFIX, NFKB1, MAPK10, SOD1, SOD2, SOD3, SP1, TXNRD1, XDH, TXNRD2, TXN2, NOX4, UGT1A6, SOD2-OT1
WIKIPATHWAYS	Oxytocin signaling	CD38, GNAQ, OXTR, PLCD1
WIKIPATHWAYS	p38 MAPK Signaling Pathway	CDC42, CREB1, ATF2, MAPK14, DAXX, DDIT3, ELK1, GRB2, HMGN1, HRAS, HSPB1, MAX, MEF2D, MAP3K1, MAP3K5, MAP3K9, MYC, PLA2G4A, MAP2K6, RAC1, RASGRF1, MAP2K4, SHC1, STAT1, MAP3K7, TGFB2, TGFBR1, TRAF2, MAPKAPK5, MKNK1, TRADD, RIPK1, RPS6KA5, MAPKAPK2

Predefined gene sets in Hepamine

**Data source**	**Pathways**	**Symbols**
WIKIPATHWAYS	Pancreatic adenocarcinoma pathway	AKT1, AKT2, ARAF, RHOA, BAD, BAK1, BAX, CCND1, BCL2L1, BRAF, BRCA2, CASP9, CDC42, CDK4, CDK6, CDKN1A, CDKN2A, CHUK, DDB2, GADD45A, DUSP6, E2F1, E2F2, E2F3, EGF, EGFR, ERBB2, MTOR, IKBKB, JAK1, KRAS, SMAD2, SMAD3, SMAD4, GADD45B, NFKB1, PEBP1, PAK1, PAK2, PAK3, PIK3CA, PIK3CB, PIK3CD, PIK3R1, PIK3R2, PLD1, PRKCD, MAPK1, MAPK3, MAPK8, MAPK9, MAPK10, MAP2K1, MAP2K2, RAC1, RAC2, RAC3, RAD51, RAF1, RALA, RALB, RALGDS, RB1, RELA, RPS6KB1, RPS6KB2, STAT1, STAT3, TGFA, TGFB1, TGFB2, TGFB3, TGFBR1, TGFBR2, TIAM1, TP53, VEGFA, PIK3R3, IKBKG, ARHGEF6, AKT3, PAK4, GADD45G, RALBP1, POLK, RIPK4, PAK6, PAK5, BUB1B-PAK6
WIKIPATHWAYS	Parkinsons Disease Pathway	APAF1, CASP2, CASP3, CASP6, CASP7, CASP9, CCNE1, MAPK14, DDC, EPRS, GPR37, PRKN, SEPT5, MAPK11, MAPK13, MAPK12, ATXN2, SLC6A3, SNCA, TH, UBB, UBA1, UBA7, UBE2G1, UBE2G2, UBE2L3, UCHL1, CCNE2, UBE2L6, SNCAIP, PARK7, SYT11, HTRA2, UBE2J1, CYCS, PINK1, UBE2J2, LRRK2, MIRLET7G, MIR106A, MIR10A, MIR127, MIR128-1, MIR128-2, MIR132, MIR136, MIR16-2, MIR17, MIR18A, MIR195, MIR19A, MIR19B1, MIR19B2, MIR20A, MIR212, MIR26A1, MIR26A2, MIR26B, MIR30A, MIR30E, MIR34B, MIR34C, MIR92A1, MIR92A2, MIR17HG, MIR338, MIR370, MIR375, MIR363, MIR20B, MIR18B, MIR433, MIR431, MIR409, MIR485, MIR497, MIR503, MIR873, MIR1224, MIR1294, MIR497HG, MIR4448, MIR5193, MIR6084

Predefined gene sets in Hepamine

Data source	Pathways	Symbols
WIKIPATHWAYS	Parkin-Ubiquitin Proteasomal System pathway	CASP1, CASP8, CCNE1, GPR37, HSPA1A, HSPA1B, HSPA1L, HSPA2, HSPA4, HSPA5, HSPA6, HSPA8, HSPA9, PRKN, SEPT5, PSMC1, PSMC2, PSMC3, PSMC4, PSMC5, PSMC6, PSMD1, PSMD2, PSMD3, PSMD4, PSMD5, PSMD7, PSMD8, PSMD9, PSMD10, PSMD11, PSMD12, PSMD13, SIAH1, SIAH2, SNCA, TUBA4A, TUBA3C, TUBB2A, UBA1, UBE2G1, UBE2G2, UBE2L3, TUBA1A, CUL1, CASK, UBE2L6, SNCAIP, PSMD6, PSMD14, STUB1, TUBA1B, TUBB3, TUBB4A, TUBB4B, RNF19A, HSPA14, UBE2J1, TUBA8, FBXW7, TUBAL3, TUBB1, TUBB6, TUBA1C, TUBA3E, TUBA3D, UBE2J2, TUBB, TUBB8, TUBB2B
WIKIPATHWAYS	Pathogenic Escherichia coli infection	ABL1, ACTB, ACTG1, RHOA, CD14, CDC42, CDH1, CTNNB1, CTTN, FYN, HCLS1, ITGB1, KRT18, NCK1, NCL, PRKCA, ROCK1, TLR4, TLR5, TUBA4A, TUBA3C, TUBB2A, EZR, WAS, YWHAZ, TUBA1A, NCK2, WASL, CLDN1, ARHGEF2, ROCK2, ARPC5, ARPC4, ARPC3, ARPC1B, ARPC2, TUBA1B, TUBB3, TUBB4A, TUBB4B, ARPC1A, YWHAQ, LY96, TUBA8, TUBAL3, TUBB1, ARPC5L, TUBB6, TUBA1C, TUBA3E, TUBA3D, TUBB, TUBB8, TUBB2B, OCLN
WIKIPATHWAYS	Pathways Affected in Adenoid Cystic Carcinoma	AKT1, ATM, ATRX, BRCA1, CEBPA, CHEK1, CREBBP, CTBP1, EP300, ERBB2, FGFR4, FOXO3, HIST1H1E, HRAS, INSRR, MAX, MYB, MYBL1, MYC, MYCN, NFIB, NOTCH1, PIK3CA, PRKDC, MAP2K2, PTEN, RAF1, SMARCA2, SMARCE1, TP53, KDM6A, KAT6A, SMC1A, ARID1A, HIST1H2AL, FGF16, MAGI1, NCOR1, MAGI2, TLK1, SRCAP, MORF4L1, CHEK2, KDM6B, DTX4, MGA, BRD1, MYCBP, CNTN6, SETD2, UHRF1, ARID4B, IL17RD, BCOR, FBXW7, MAML3, CMTR2, ERBIN, KMT2C, BCORL1, NSD1, ARID5B, FOXP2, JMJD1C, KANSL1, MIR1281

Predefined gene sets in Hepamine

Data source	Pathways	Symbols
WIKIPATHWAYS	Pathways in clear cell renal cell carcinoma	ACACA, ACACB, ACLY, AKT1, ALDOA, ALDOB, ALDOC, ARNT, CDH13, CREBBP, EGFR, ENO1, ENO2, ENO3, EP300, FASN, FLT1, MTOR, GAPDH, GPI, GRB10, HIF1A, HK1, HK2, HK3, KCNJ2, KDR, LDHA, LDHB, LDHC, MDH1, ME1, PDGFB, PDGFRA, PDGFRB, ENPP3, PFKL, PFKM, PFKP, PGK1, PGK2, PGM1, PKLR, PKM, PLOD2, PSPH, PTEN, RHEB, SHMT1, SHMT2, SLC2A1, STAT3, ZEB1, TGFB1, TGFB2, TGFB3, TPI1, TSC1, TSC2, VEGFA, VHL, SSPN, KDM5C, BAP1, CAMK1, KSR1, SQSTM1, RAPGEF5, SDS, PHGDH, SETD2, PSAT1, PBRM1, PGM2, RPTOR, MLST8, DEPTOR, BHLHE41, PGBD5, CEP290, AKT1S1, TOX2, SDSL, EFCAB3, LDHD, MIR1281, MIR6778
WIKIPATHWAYS	PDGF Pathway	RHOA, CDC42, CHUK, ELK1, FOS, GRB2, HRAS, JAK1, JUN, MAP3K1, COX2, NFKBIA, PAK1, PDGFA, PDGFB, PDGFRB, PIK3R1, PLA2G4A, PLCG1, MAPK1, MAPK3, MAPK8, MAP2K1, PTPN11, RAC1, RAF1, RASA1, MAP2K4, SHC1, SOS1, SRC, SRF, STAT1, STAT3, TIAM1, VAV1, VAV2, WASL, ARFIP2
WIKIPATHWAYS	PDGFR-beta pathway	ELK1, FOS, GRB2, HRAS, JAK1, JAK2, JUN, MAP3K1, PDGFRB, PIK3CA, PIK3R1, PLCG1, PRKCA, PRKCB, MAPK3, MAPK8, MAP2K1, EIF2AK2, RAF1, RASA1, MAP2K4, SHC1, SOS1, SRF, STAT1, STAT3, STAT5A, STAT5B, STAT6
WIKIPATHWAYS	Pentose Phosphate Pathway	G6PD, PGD, RPE, TALDO1, TKT, RPIA, PGLS

Predefined gene sets in Hepamine

Data source	Pathways	Symbols
WIKIPATHWAYS	Peptide GPCRs	AGTR1, AGTR2, AVPR1A, AVPR1B, AVPR2, BDKRB1, BDKRB2, CXCR5, BRS3, C3AR1, C5AR1, CCKAR, CCKBR, CCR1, CCR3, CCR4, CCR5, CCR6, CCR7, CCR8, CX3CR1, EDNRA, EDNRB, FPR1, FPR2, FPR3, FSHR, ACKR1, GALR1, GHSR, GNRHR, CCR10, CXCR3, GRPR, HCRTR1, HCRTR2, CXCR1, CXCR2, LHCGR, MC1R, MC2R, MC3R, MC4R, MC5R, NMBR, NPY1R, NPY2R, NPY6R, NPY5R, NTSR1, OPRD1, OPRK1, OPRL1, OPRM1, OXTR, NPY4R, SSTR1, SSTR2, SSTR3, SSTR4, SSTR5, TACR2, TACR1, TACR3, TRHR, TSHR, CXCR4, GALR3, GALR2, ATP8A1, CXCR6, CCR9, NTSR2, TAC4, CCR2
WIKIPATHWAYS	Peroxisomal beta-oxidation of tetracosanoyl-CoA	ACAA1, ACOX1, HSD17B4, SCP2
WIKIPATHWAYS	Phase I biotransformations, non P450	CES1, ESD, LIPA, PON1, PON2, PON3, CES2, CES5A
WIKIPATHWAYS	Phosphodiesterases in neuronal function	ADCY1, ADCY2, ADCY3, ADCY5, ADCY6, ADCY7, ADCY8, ADCY9, ADORA2A, CHRNA7, CREB1, DRD1, DRD2, GRIA1, GRIN1, GRIN2A, GRIN2B, GRIN2C, GRIN2D, GUCY1B2, GUCY1A2, GUCY1A1, GUCY1B1, NOS1, PDE1A, PDE1C, PDE2A, PDE3A, PDE3B, PDE4A, PDE4B, PDE4C, PDE4D, PDE6A, PDE6C, PDE6D, PDE6G, PDE6H, PDE7A, PDE8A, PDE9A, PDE1B, PDE6B, PDE8B, PDE5A, PDE10A, PDE7B, PDE11A, ADCY10, PPP1R1B, CHRFAM7A, ADCY4, PDE12, LOC729966, LOC101929970
WIKIPATHWAYS	Photodynamic therapy-induced AP-1 survival signaling.	FAS, FASLG, BAK1, BAX, CCND1, BCL2, BCL2L1, BCL3, BID, CCNA2, CCNE1, CDKN1A, CDKN2A, ATF2, MAPK14, HBEGF, EGFR, ELK1, FGF7, FOS, HSP90AA1, IFNG, IL2, IL6, JUN, JUNB, MCL1, MAP3K5, MMP2, NFE2L2, PDGFRA, MAPK8, MAPK11, MAPK13, MAP2K3, MAP2K6, MAP2K7, RB1, MAPK12, MAP2K4, TNF, TNFRSF1A, TP53, TRAF2, TRAF5, TRAF6, TNFSF10, CFLAR, BCL2L11, BMF, MIR8085

Predefined gene sets in Hepamine

Data source	Pathways	Symbols
WIKIPATHWAYS	Photodynamic therapy-induced HIF-1 survival signaling	ANGPT1, ANGPT2, BIRC5, ARNT, BAK1, BAX, BCL2A1, BCL2L1, BID, BNIP3, BNIP3L, EDN1, EPO, HIF1A, HK1, IGFBP1, IGFBP2, IGFBP3, LDHA, MCL1, NOS2, SERPINE1, PDHA1, PFKL, PGK1, PKM, PMAIP1, PTGS2, SLC2A1, SLC2A3, SLC16A1, TGFA, TGFB3, TP53, VEGFA, EGLN1, HIF1AN
WIKIPATHWAYS	Photodynamic therapy-induced NFE2L2 (NRF2) survival signaling	ABCC6, CES1, ABCC2, MAPK14, NQO1, EPHX1, FOS, GCLC, GCLM, GSTP1, HMOX1, JUN, NFE2L2, NQO2, MAPK8, MAPK11, MAPK13, MAPK12, ABCC3, ABCG2, KEAP1, ABCC4, SRXN1, LOC105369239
WIKIPATHWAYS	Photodynamic therapy-induced NF-kB survival signaling	BIRC2, BIRC3, BIRC5, CCND1, BCL2A1, BCL2L2, CD40LG, CHUK, CSF2, CXCL2, ICAM1, IKBKB, IL1A, IL1B, IL2, IL6, CXCL8, MMP1, MMP2, MMP3, MMP9, NFKB1, NFKB2, PTGS2, REL, RELA, RELB, SELE, TNF, TNFRSF1A, TRAF6, VCAM1, VEGFA, CFLAR, EGLN2
WIKIPATHWAYS	Photodynamic therapy-induced unfolded protein response	ASNS, ATF3, ATF4, CALR, DDIT3, ERN1, HSPA5, DNAJB9, NARS, NFE2L2, DNAJC3, SULT1E1, HSP90B1, UBE2E1, WARS, XBP1, EIF2AK3, EDEM1, BCL2L11, PDIA6, ATF6, PPP1R15A, BBC3, DNAJB11, TRIB3, EIF2A, ERP27, MIR3191, MIR3652
WIKIPATHWAYS	Physiological and Pathological Hypertrophy of the Heart	AGT, RHOA, CALM1, CALM2, CAMK2D, MAPK14, CTF1, EDN1, FOS, GATA4, IL6ST, JUN, LIFR, NFATC4, PPP3CA, PPP3CB, PRKCB, PRKCE, MAPK1, MAPK8, MAPK11, MAP2K3, RAC1, STAT3, MYEF2
WIKIPATHWAYS	Phytochemical activity on NRF2 transcriptional activation	CEBPB, NQO1, EPHB2, GCLC, GCLM, GSTA2, HMOX1, MAF, NFE2L2, PIK3CA, PRKCA, MAPK8, AIMP2, KEAP1, SLC7A11
WIKIPATHWAYS	PI3K-AKT-mTOR signaling pathway and therapeutic opportunities	AKT1, BAD, CDKN1B, EIF4EBP1, FOXO1, FOXO3, MTOR, GRB10, GSK3B, HRAS, KRAS, FOXO4, NOS3, NRAS, PDK1, PIK3CA, PIK3CB, PIK3CG, PIK3R1, PIK3R2, PTEN, RHEB, TSC2, TFEB, ULK1, PIK3R3, ATG13, RB1CC1, RPTOR, RICTOR

Predefined gene sets in Hepamine

Data source	Pathways	Symbols
WIKIPATHWAYS	PI3K/AKT/mTOR - VitD3 Signalling	AKT1, CD80, CD86, CYP24A1, MTOR, GSK3B, HK3, HLA-DRA, IL10, IL12A, LDHA, MYC, PDHA1, PFKFB4, PIK3CA, PRKAA2, RELA, RXRA, SLC2A3, TSC1, TSC2, VDR

Predefined gene sets in Hepamine

Data source	Pathways	Symbols
WIKIPATHWAYS	PI3K-Akt Signaling Pathway	AKT1, AKT2, ANGPT1, ANGPT2, FASLG, ATF4, BAD, CCND1, BCL2, BCL2L1, BDNF, BRCA1, CASP9, CCND2, CCND3, CCNE1, CD19, CDK2, CDK4, CDK6, CDKN1A, CDKN1B, CHAD, CHRM1, CHRM2, CHUK, COL1A1, COL1A2, COL2A1, COL4A1, COL4A2, COL4A3, COL4A4, COL4A5, COL4A6, COL6A1, COL6A2, COL6A3, COL9A1, COL9A2, COL9A3, COMP, CREB1, ATF2, ATF6B, CSF1, CSF1R, CSF3, CSF3R, CSH1, CSH2, CSHL1, LPAR1, EFNA1, EFNA2, EFNA3, EFNA4, EFNA5, EGF, EGFR, EPHA2, EIF4B, EIF4E, EIF4EBP1, EPO, EPOR, F2R, FGF1, FGF2, FGF3, FGF4, FGF5, FGF6, FGF7, FGF8, FGF9, FGF10, FGF11, FGF12, FGF13, FGF14, FGFR1, FGFR3, FGFR2, FGFR4, VEGFD, FOXO3, FLT1, FLT3, FLT3LG, FLT4, FN1, MTOR, G6PC, GH1, GH2, GHR, GNB1, GNB2, GNB3, GNG3, GNG4, GNG5, GNG7, GNG10, GNG11, GNGT1, GNGT2, LPAR4, GRB2, GSK3B, GYS1, GYS2, HGF, HRAS, HSP90AA1, HSP90AB1, TNC, IBSP, IFNA1, IFNA2, IFNA4, IFNA5, IFNA6, IFNA7, IFNA8, IFNA10, IFNA13, IFNA14, IFNA16, IFNA17, IFNA21, IFNAR1, IFNAR2, IFNB1, IGF1, IGF1R, IGF2, IKBKB, IL2, IL2RA, IL2RB, IL2RG, IL3, IL3RA, IL4, IL4R, IL6, IL6R, IL7, IL7R, INS, INSR, ITGA6, IRS1, ITGA1, ITGA2, ITGA2B, ITGA3, ITGA4, ITGA5, ITGA7, ITGA9, ITGAV, ITGB1, ITGB3, ITGB4, ITGB5, ITGB6, ITGB7, ITGB8, JAK1, JAK2, JAK3, KDR, KIT, KRAS, LAMA2, LAMA3, LAMA4, LAMA5, LAMB1, LAMB2, LAMB3, LAMC1, LAMC2, MCL1, MDM2, MET, KITLG, MYB, MYC, NFKB1, NGF, NGFR, NOS3, NRAS, NTF3, NTF4, NTRK1, NTRK2, OSM, PCK1, PCK2, PDGFA, PDGFB, PDGFRA, PDGFRB, PDPK1, PGF, PIK3CA, PIK3CB, PIK3CD, PIK3CG, PIK3R1, PIK3R2, PPP2CA, PPP2CB, PPP2R1A, PPP2R1B, PPP2R2A, PPP2R2B, PPP2R2C, PPP2R3A, PPP2R5A, PPP2R5B, PPP2R5C, PPP2R5D, PPP2R5E, PRKAA1, PRKAA2, PRKCA, PKN1, PKN2, MAPK1, MAPK3, MAP2K1, MAP2K2, PRL, PRLR, RELN, PTEN, PTK2, RAC1, RAF1, RBL2, RELA, RHEB, RPS6, RPS6KB1, RPS6KB2, SGK1, SOS1, SOS2, SPP1, STK11, SYK, TEK, TGFA, THBS1, THBS2, THBS3, THBS4, TLR2, TLR4, TNR, TNXB, TP53, HSP90B1, TSC1, TSC2, VEGFA, VEGFB, VEGFC, VTN, VWF, FGF23, TCL1A, PIK3R3, ITGA10, ITGA8, IKBKG, FGF18, FGF17, CCNE2, LPAR2, OSMR, EIF4E2, CREB5, TCL1B, FGF19, AKT3

Predefined gene sets in Hepamine

**Data source**	**Pathways**	**Symbols**
WIKIPATHWAYS	Polyol Pathway	ALDOB, AKR1B1, KHK, SORD
WIKIPATHWAYS	PPAR Alpha Pathway	ACAA1, ACADM, APOA1, APOA2, APOC3, CCND1, CDK1, CDK4, CPT1A, CPT2, CYP4A11, CYP7A1, CYP8B1, DBI, EHHADH, FABP1, MYC, PLTP, PPARA, RXRA, SCP2, NR1H3, UGT1A9, KLK15, APOA5, SLC27A1
WIKIPATHWAYS	PPAR signaling pathway	ACAA1, ACADL, ACADM, ACOX1, APOA1, APOA2, APOC3, AQP7, CD36, CPT1A, CPT1B, CPT2, CYP4A11, CYP7A1, CYP8B1, CYP27A1, DBI, EHHADH, FABP4, FABP1, FABP2, FABP3, FABP5, FABP6, FABP7, ACSL1, ACSL3, ACSL4, GK2, HMGCS2, ILK, LPL, ME1, MMP1, OLR1, PCK1, PCK2, PDPK1, PLIN1, PLTP, PPARA, PPARD, PPARG, RXRA, RXRB, RXRG, SCD, SCP2, UCP1, ACOX2, ACOX3, ADIPOQ, FADS2, NR1H3, SORBS1, SLC27A5, SLC27A4, SLC27A2, ACSBG1, ACSL6, SLC27A6, ANGPTL4, ACSL5, ACSBG2, APOA5, CPT1C, SLC27A1
WIKIPATHWAYS	Prader-Willi and Angelman Syndrome	BBS4, CCND1, DLX5, E2F1, GABRA5, GABRB3, GABRD, GABRG1, GABRG2, GABRG3, GABRR1, GABRR2, HTR2C, MSX1, NDN, NGF, NHLH2, OCA2, PCM1, PCSK1, PRKCZ, SNRPN, UBE3A, MKRN3, HERC2, SNURF, FEZ2, FEZ1, CYFIP1, NPAP1, SLC45A2, MAGEL2, ATP10A, NIPA2, SNORD107, TUBGCP5, NIPA1, GABRR3, SNORD109A, SNORD109B, SNORD64, PWRN1
WIKIPATHWAYS	Pregnane X Receptor pathway	ABCC2, CYP2A6, CYP3A7, CYP2B6, CYP2C19, CYP2C9, CYP3A4, CYP3A5, FOXO1, GSTA2, HSP90AA1, ABCB1, PSMC5, RXRA, SRC, SULT2A1, DNAJC7, NCOA3, NRIP1, NCOA1, ABCC3, NR1I2, ABCC4, NCOA2, SLCO1B1, PPARGC1A, SRPX2, UGT1A6, UGT1A9, UGT1A4, UGT1A1, UGT1A3, CYP4F12

Predefined gene sets in Hepamine

Data source	Pathways	Symbols
WIKIPATHWAYS	Preimplantation Embryo	AQP3, AQP9, ATP1A1, ZFP36L2, CDH1, CDX2, DDIT3, DLX2, E2F5, EGR1, ELAVL1, ESRRA, FOXD1, FOSB, GATA2, GATA3, HMGA1, HNRNPAB, IRF4, MXD1, NR3C2, MOS, MYBL1, PBX1, POU5F1, SIX3, SMARCA4, SOX2, SOX11, TBX3, TEAD4, TFAP2B, NKX2-1, ZFP36, BARX2, KHSRP, KLF4, IRX5, CELF3, DNMT3L, SOX8, H2AFY2, BATF3, MTA3, NANOG, TCF7L1, FOXQ1, NLRP5, ZFP42, ZSCAN4, TPRX1, ZAR1, LEUTX, PADI6, DPPA3, NANOGNB, MIR302A, ARGFX, DPRX, MIR3940
WIKIPATHWAYS	Primary Focal Segmental Glomerulosclerosis FSGS	ACTN4, JAG1, AKT1, CAMK2B, CD80, SCARB2, CD151, CDH2, CDKN1A, CDKN1B, CDKN1C, COL4A3, COL4A4, COL4A5, CR1, CTNNB1, CTSL, DAG1, DNM1, FAT1, FYN, ILK, IRF6, ITGA3, ITGAV, ITGB1, ITGB3, ITGB4, KRT8, LAMA5, LAMB2, LIMS1, LMX1B, LRP6, LRP5, MKI67, MME, MYH9, MYO1E, NCK1, NOTCH1, NPHS1, PAX2, PCNA, PLAUR, PLCG1, PODXL, PTK2, PTPRO, TGFB1, TLN1, TLR4, TRPC6, UTRN, VCL, VIM, VTN, WNT1, WT1, NPHS2, CLDN1, YWHAQ, SYNPO, DKK1, CD2AP, SMARCAL1, PLCE1, PARVA, INF2, KIRREL2, KIRREL3, AGRN, MIR4758, MIR6852
WIKIPATHWAYS	Prion disease pathway	BCL2, CASP3, CHD2, CREB1, EBF1, ELK1, EP300, FGFR1, FYN, PDIA3, HSPA5, IRF4, MEF2C, NCAM1, NFKB1, PAX5, POU2F2, MAPK1, MAPK3, PRNP, PTK2, RAD21, RFX5, RXRA, SPI1, STAT3, TBP, HSP90B1, SMC3, BATF, CTCF, BCL11A, MIR1281, MIR3175, MIR3652, MIR3610, CASP12

Predefined gene sets in Hepamine

Data source	Pathways	Symbols
WIKIPATHWAYS	Prolactin Signaling Pathway	AKT1, CASP3, CBL, CISH, MAPK14, CTSD, EIF4EBP1, ELK1, ERBB2, FLNA, FOS, MTOR, FYN, GRB2, GSK3B, HRAS, IRF1, IRS1, ITGB1, JAK1, JAK2, JUN, MYC, NEK3, NFKB1, NFKBIA, NFKBIB, NOS2, PAK1, PIK3CA, PIK3CB, PIK3CG, PIK3R1, PIK3R2, PPIA, PPIB, MAPK1, MAPK3, MAPK8, MAPK9, MAP2K1, MAP2K2, PRL, PRLR, PTK2, PTPN1, PTPN6, PTPN11, PXN, RAC1, RAF1, RELA, RPS6, RPS6KA2, RPS6KB1, SHC1, SOS1, SRC, STAT1, STAT3, STAT5A, STAT5B, TEC, VAV1, VAV2, YWHAG, YWHAZ, ZAP70, SOCS1, IRS2, SOCS2, SOCS3, GAB2, PIAS3, AGAP2, SIRPA
WIKIPATHWAYS	Proprotein convertase subtilisin/kexin type 9 (PCSK9) mediated LDL receptor degradation	LDLR, PCSK9, MIR6886
WIKIPATHWAYS	Prostaglandin Synthesis and Regulation	AKR1B1, ANXA1, ANXA2, ANXA3, ANXA4, ANXA5, ANXA6, CBR1, CYP11A1, AKR1C1, AKR1C2, EDN1, EDNRA, EDNRB, HPGD, HSD11B1, HSD11B2, MITF, PLA2G4A, PPARG, PRL, PTGDR, PTGDS, PTGER1, PTGER2, PTGER3, PTGER4, PTGFR, PTGFRN, PTGIR, PTGIS, PTGS1, PTGS2, S100A6, S100A10, SOX9, TBXA2R, TBXAS1, SCGB1A1, AKR1C3, PTGES, ABCC4, PPARGC1A, HPGDS, PPARGC1B
WIKIPATHWAYS	Proteasome Degradation	H2AFX, H2AFZ, HLA-A, HLA-B, HLA-C, HLA-E, HLA-F, HLA-G, IFNG, NEDD4, PSMA1, PSMA2, PSMA3, PSMA4, PSMA5, PSMA6, PSMA7, PSMB1, PSMB2, PSMB3, PSMB4, PSMB5, PSMB6, PSMB7, PSMB8, PSMB9, PSMB10, PSMC1, PSMC2, PSMC3, PSMC4, PSMC5, PSMC6, PSMD1, PSMD2, PSMD3, PSMD4, PSMD5, PSMD7, PSMD8, PSMD9, PSMD10, PSMD11, PSMD12, PSMD13, PSME1, PSME2, RPN1, RPN2, UBB, UBC, UBA1, UBA7, UBE2B, UBE2D1, UBE2D2, UBE2D3, UCHL1, UCHL3, HIST1H2AB, PSMD6, PSME3, MIR5193, MIR7703
WIKIPATHWAYS	PTF1A related regulatory pathway	CTNNB1, FGF10, HES1, RBPJ, PDX1, NKX6-1, NOTCH1, PROX1, KAT2B, RBPJL, PTF1A

Predefined gene sets in Hepamine

Data source	Pathways	Symbols
WIKIPATHWAYS	Purine metabolism	ADA, ADSL, AMPD1, APRT, ATIC, DGUOK, GRM5, HPRT1, IMPDH1, PPAT, PRPS1, RRM2B
WIKIPATHWAYS	Pyrimidine metabolism	POLR3D, CAD, ENTPD1, ENTPD3, CDA, CTPS1, DCK, DCTD, DHODH, DPYD, DPYS, DTYMK, DUT, TYMP, NME1, NME2, NME3, NME4, ENPP1, ENPP3, POLA1, POLD1, POLD2, POLE, POLE2, POLR2A, POLR2B, POLR2C, POLR2D, POLR2E, POLR2G, POLR2H, POLR2I, POLR2J, POLR2K, POLR2L, PRIM1, PRIM2, RRM1, RRM2, TK1, TK2, TYMS, UCK2, UMPS, UPP1, POLR1C, NME6, POLR3F, POLR3G, POLR3C, POLD3, POLR3A, POLA2, POLR1A, NME7, NT5C, ZNRD1, RRM2B, POLR1D, CMPK1, POLR3K, UPB1, POLE3, UCKL1, POLR3B, POLR3E, CTPS2, POLE4, NT5M, POLD4, POLR1E, DCTPP1, UCK1, POLR1B, POLR3GL, PNPT1, CMPK2, UPRT, POLR3H, TWISTNB, POLR2J2, POLR2J3, NME1-NME2, MIR1914, MIR3658, LOC101060521
WIKIPATHWAYS	Pyrimidine metabolism and related diseases	DPYD, DPYS, TYMP, OTC, TYMS, NT5C, RRM2B, NT5C3A, UPB1
WIKIPATHWAYS	RAC1/PAK1/p38/MMP2 Pathway	AKT1, ANGPT1, ANGPT2, BIRC5, BAD, BAX, CASP7, CASP9, CHUK, CRK, MAPK14, CTNNB1, EGFR, EIF4EBP1, ERBB2, FOXO1, FN1, GRB2, GRB7, GRB14, HRAS, IKBKB, ITGB1, KRAS, STMN1, MMP2, MSH2, MYC, NCK1, NFKB1, NFKBIA, NOS1, NOS2, NRAS, PAK1, PIK3CA, PIK3R1, MAPK1, MAPK3, MAPK8, MAPK11, MAPK9, MAPK13, PTK2, PTPN11, PXN, RAC1, RAD51, RASA1, RELA, RS1, MAPK12, SOS1, SRC, STAT3, STAT5A, STAT5B, TEK, TIE1, TP53, IKBKG, DOK2, ANGPTL1, TNIP1, YAP1, ANGPT4, TNIP2, TNIP3, MIR3917
WIKIPATHWAYS	RalA downstream regulated genes	CDC42, HRAS, KRAS, NRAS, RAC1, RAC2, RAC3, RALA, YBX3, RALBP1, EXOC2, EXOC8

Predefined gene sets in Hepamine

Data source	Pathways	Symbols
WIKIPATHWAYS	RANKL/RANK (Receptor activator of NFKB (ligand)) Signaling Pathway	ACP5, AKT1, AKT2, CALCR, CBL, CDC42, CHUK, MAPK14, CTSK, FHL2, FOS, MTOR, ICAM1, IKBKB, JUN, LYN, MITF, NFATC1, NFKB1, NFKB2, NFKBIA, TNFRSF11B, PIK3R1, PIK3R2, PLCG1, MAPK1, MAPK3, MAPK8, MAPK9, MAP2K1, MAP2K6, MAP2K7, PTK2, RAC1, RELA, RELB, SPI1, SRC, STAT1, SYK, MAP3K7, TRAF1, TRAF2, TRAF3, TRAF5, TRAF6, VCAM1, IKBKG, TNFSF11, TNFRSF11A, SQSTM1, PAPSS2, GAB2, TAB1, TAB2
WIKIPATHWAYS	Ras Signaling	ABL1, ABL2, AKT1, AKT2, FASLG, ARF6, RHOA, BAD, BCL2L1, CALM1, CALM2, CALM3, CALML3, CDC42, CHUK, CSF1R, EGFR, EPHA2, ELK1, ETS1, ETS2, FGFR1, FGFR3, FGFR2, FGFR4, FLT1, FLT3, FLT4, GAB1, GNB1, GNB2, GNB3, GNG3, GNG4, GNG5, GNG7, GNG10, GNG11, GNGT1, GNGT2, GRB2, GRIN1, GRIN2A, GRIN2B, HRAS, HTR7, IGF1R, IKBKB, INSR, KDR, KIT, KRAS, MET, AFDN, FOXO4, NF1, NFKB1, NGFR, NRAS, NTRK1, NTRK2, PAK1, PAK2, PAK3, PDGFRA, PDGFRB, PIK3CA, PIK3CB, PIK3CD, PIK3R1, PIK3R2, PLA2G1B, PLA2G2A, PLA2G4A, PLA2G5, PLCG1, PLCG2, PLD1, PLD2, PRKACA, PRKACB, PRKACG, PRKCA, PRKCB, PRKCG, MAPK1, MAPK3, MAPK8, MAPK9, MAPK10, MAP2K1, MAP2K2, PTPN11, RGL2, RAB5A, RAB5B, RAB5C, RAC1, RAC2, RAC3, RAF1, RALA, RALB, RALGDS, RAP1A, RAP1B, RASA1, RASA2, RASGRF1, RASGRF2, REL, RELA, RRAS, SHC1, SOS1, SOS2, STK4, TEK, TIAM1, ZAP70, SHOC2, BRAP, PLA2G6, PLA2G10, RASAL1, PIK3R3, IKBKG, PLA2G4C, JMJD7-PLA2G4B, SYNGAP1, KSR1, RASAL2, RIN1, RAPGEF5, GAB2, AKT3, RASGRP1, RASA4, RASGRP2, PAK4, GNB5, RALBP1, PLA2G16, RASSF1, RRAS2, MRAS, RASA3, RGL1, SHC2, RASGRP3, PLA2G2D, LAT, PLA2G2E, PLA2G3, PLCE1, PLA1A, GNG13, CALML5, SHC3, GNG2, EXOC2, GNG12, PAK6, PAK5, GNB4, PLA2G2F, RASAL3, PLA2G12A, RASSF5, TTBK1, PLA2G12B, CALML4, GNG8, RASGRP4, PLA2G4E, CALML6, PLA2G4F, KSR2, PLA2G4D, PLA2G2C, SHC4, PLA2G4B, RASA4B, MIR5004, LOC102724229, BUB1B-PAK6

Predefined gene sets in Hepamine

Data source	Pathways	Symbols
WIKIPATHWAYS	Regulation of Actin Cytoskeleton	ACTB, ACTG1, ACTN1, APC, RHOA, BDKRB1, BDKRB2, BRAF, CD14, CDC42, CFL1, CFL2, CHRM1, CHRM2, CHRM3, CHRM4, CHRM5, CRK, CSK, DIAPH1, DOCK1, EGF, EGFR, F2, F2R, FGD1, FGF1, FGF2, FGF3, FGF4, FGF5, FGF6, FGF7, FGF8, FGF9, FGF10, FGF11, FGF12, FGF13, FGF14, FGFR1, FGFR3, FGFR2, FGFR4, FN1, GNA12, ARHGAP35, GSN, ITGA1, KRAS, LIMK1, MOS, MSN, MYH10, MYL1, MYL3, MYLK, PPP1R12A, NRAS, PAK1, PAK2, PAK3, PDGFA, PDGFB, PDGFRA, PDGFRB, PFN1, PIK3C2A, PIK3C2B, PIK3C2G, PIK3C3, PIK3CA, PIK3CB, PIK3CD, PIK3CG, PIK3R1, PIK3R2, PIP4K2A, MAPK1, MAPK3, MAPK4, MAPK6, MAP2K1, MAP2K2, PTK2, PXN, RAC1, RAC2, RAC3, RAF1, RDX, ROCK1, RRAS, SLC9A1, SOS1, SOS2, TMSB4X, VAV1, VCL, VIL1, EZR, WAS, RASSF7, FGF23, PIP5K1A, PIP5K1B, PIP4K2B, PIK3R3, FGF18, FGF17, FGF16, IQGAP1, ARHGEF7, WASF1, ARHGEF1, ARHGEF6, ROCK2, BCAR1, FGF19, ARPC5, ABI2, WASF2, APC2, PAK4, BAIAP2, GNA13, NCKAP1, RRAS2, MRAS, PIP5K1C, PIK3R5, FGF20, FGF21, CYFIP2, FGF22, GIT1, PIK3R4, ARHGEF4, SSH1, SSH3, ENAH, BRK1, GNG12, PAK6, PAK5, PIP4K2C, DIAPH3, SSH2, PIP5KL1, LOC100505585, BUB1B-PAK6
WIKIPATHWAYS	Regulation of Apoptosis by Parathyroid Hormone-related Protein	AKT1, BAK1, BAX, BCL2, BCL2A1, BCL2L1, BCL2L2, BID, BOK, GSK3A, GSK3B, ITGA6, ITGB4, MCL1, MYC, PIK3CG, PTHLH, BCL2L10, BCL2L13, BCL2L14, BCL2L12, BCL2L15
WIKIPATHWAYS	Regulation of Microtubule Cytoskeleton	ABL1, AKT1, APC, CAMK4, CDK1, CDC42, CFL2, DIAPH1, DPYSL2, DVL1, MARK2, EPHB2, F2RL2, GNAQ, GSK3B, STMN1, LIMK1, MAP1B, MAPT, MARK1, PAK1, PIK3CA, PRKACA, PRKCA, PTEN, PTPRA, RAC1, RHO, ROCK1, CLIP1, SRC, STAT3, TIAM1, TRIO, AURKB, MAPKAPK2, TESK2, KIF2C, TPPP, MAPRE1, CLASP1, PARD6A, TAOK1, WNT3A, PHLDB2, SPRED1, MIR3917, MIR4523, MIR6808

Predefined gene sets in Hepamine

Data source	Pathways	Symbols
WIKIPATHWAYS	Regulation of sister chromatid separation at the metaphase-anaphase transition	BUB1, BUB1B, CDC20, CENPE, MAD2L1, RAD21, SMC1A, MAD1L1, SMC3, BUB3, PTTG1, ESPL1, STAG1, ANAPC2, ANAPC11, MIR3610
WIKIPATHWAYS	Regulation of toll-like receptor signaling pathway	AKT1, AKT2, BTK, CASP8, CD14, CD80, CD86, CD40, CHUK, CISH, MAP3K8, MAPK14, CYLD, FOS, IFNA1, IFNA2, IFNA4, IFNA5, IFNA6, IFNA7, IFNA8, IFNA10, IFNA13, IFNA14, IFNA16, IFNA17, IFNA21, IFNAR1, IFNAR2, IFNB1, IKBKB, IL1B, IL6, CXCL8, IL12A, IL12B, CXCL10, IRAK1, IRAK2, IRF3, IRF5, IRF7, JUN, LBP, CD180, SMAD6, MBL2, CXCL9, MYD88, NFKB1, NFKB2, NFKBIA, PIK3CA, PIK3CB, PIK3CD, PIK3CG, PIK3R1, PIK3R2, PLK1, MAPK1, MAPK3, MAPK8, MAPK11, MAPK9, MAPK10, MAPK13, MAP2K1, MAP2K2, MAP2K3, MAP2K6, MAP2K7, PTPN6, RAC1, RELA, MAPK12, CCL3, CCL4, CCL5, CXCL11, MAP2K4, SFTPD, SPP1, STAT1, SYK, MAP3K7, TLR1, TLR2, TLR3, TLR4, TLR5, TNF, TNFAIP3, TRAF3, TRAF6, USP7, PIK3R3, IKBKG, SOCS1, CTNNAL1, RIPK1, FADD, SQSTM1, IKBKE, AKT3, RNF41, TLR6, TAB1, RBCK1, ZMYND11, TRAFD1, IRAK3, SARM1, TAB2, PIK3R5, LY96, TBK1, TMED7, IRAK4, TLR7, TLR8, TLR9, TREM1, FBXW5, TOLLIP, RNF216, RNF31, OTUD5, PELI2, PELI1, SIGIRR, MLST8, CUEDC2, TIFA, TIRAP, TICAM1, PELI3, TAB3, TICAM2, MIRLET7E, MIRLET7I, MIR105-1, MIR98, SFTPA2, MIR718, MIR6502
WIKIPATHWAYS	Regulation of Wnt/B-catenin Signaling by Small Molecule Compounds	APC, CSNK1A1, CTNNB1, DVL2, GSK3B, LRP1, SFRP4, TCF4, WNT1, AXIN1, FZD1, FZD7, FZD8, TNKS, DKK3, LEF1, MBOAT1, MIR4683

Predefined gene sets in Hepamine

Data source	Pathways	Symbols
WIKIPATHWAYS	Retinoblastoma Gene in Cancer	ABL1, BARD1, CCND1, CCNA2, CCNB1, CCND3, CCNE1, CDK1, CDC25A, CDC25B, CDK2, CDK4, CDK6, CDKN1A, CDKN1B, CHEK1, DCK, DHFR, DNMT1, E2F1, E2F2, E2F3, FANCG, MSH6, H2AFZ, HDAC1, HMGB1, HMGB2, PRMT2, STMN1, MCM3, MCM4, MCM6, MCM7, MDM2, MYC, NPAT, ORC1, PCNA, POLA1, POLE, POLE2, PRIM1, PRKDC, MAPK13, RABIF, RAF1, RB1, RBBP4, RBBP7, RBP1, RFC3, RFC4, RFC5, RPA1, RPA2, RPA3, RRM1, RRM2, SKP2, SMARCA2, HLTF, SUV39H1, TFDP1, TFDP2, TOP2A, TP53, TTK, TYMS, WEE1, CCDC6, SMC1A, CDC7, CDC45, SAP30, SMC3, CCNB2, CCNE2, SMC2, POLD3, PLK4, FAF1, KIF4A, SIN3A, ANLN, ZNF655, CDT1, MIR3917
WIKIPATHWAYS	Rett syndrome causing genes	BRAF, CHD4, CRK, FOXG1, GABRA3, GABRD, GNAO1, GPS2, GRIN2A, GRIN2B, HTT, HDAC1, HIVEP2, IMPDH2, KCNJ10, MECP2, MEF2C, SCN1A, SCN2A, SCN8A, SMARCA1, SMARCA2, SMARCA4, CDKL5, STXBP1, TBL1X, TCF4, SMC1A, TRRAP, SYNGAP1, EIF2B2, HAP1, TAF1B, GABBR2, NCOR1, NCOR2, HDAC5, KDM5B, RHOBTB2, SYNE2, SATB2, CECR2, ACTL6B, HDAC8, XAB2, TBL1XR1, SHANK3, SRRM3, MIR5004
WIKIPATHWAYS	RIG-I-like Receptor Signaling	CASP8, CASP10, CHUK, MAPK14, CYLD, DDX3X, IFNA1, IFNB1, IFNG, IKBKB, CXCL8, CXCL10, IRF3, IRF7, MAP3K1, NFKB1, NFKBIA, NFKBIB, PIN1, MAPK8, MAPK11, MAPK9, MAPK10, MAPK13, RELA, MAPK12, CXCL12, MAP3K7, TNF, TRAF2, TRAF3, TRAF6, TRIM25, IKBKG, DDX3Y, TRADD, RIPK1, FADD, ATG12, ATG5, ISG15, IKBKE, TBKBP1, TANK, DDX17, SNW1, DDX58, TKFC, TBK1, RNF125, OTUD5, IFNK, MAVS, IFIH1, AZI2, DHX58, NLRX1, SIKE1, IFNE, TMEM173, MIR3614
WIKIPATHWAYS	RNA interference	DICER1, DROSHA, DGCR8, XPO5, ERI1, MIR1306, MIR3618
WIKIPATHWAYS	Robo4 and VEGF Signaling Pathways Crosstalk	KDR, RAC1, SRC, VEGFA, SLIT2, ROBO4

Predefined gene sets in Hepamine

Data source	Pathways	Symbols
WIKIPATHWAYS	Role of Osx and miRNAs in tooth development	ALPL, BMP7, RUNX2, CTNNB1, DMP1, DSPP, NOTCH1, NOTCH2, NOTCH3, NOTCH4, HNF1A, KLF4, DKK1, SOST, SP7, MIRLET7A1, MIRLET7A2, MIRLET7C, MIRLET7D, MIRLET7E, MIRLET7F1, MIRLET7F2, MIRLET7G, MIRLET7I, MIR143, MIR145, MIR204, MIR211, MIR29A, MIR29B1, MIR32, MIR34A, MIR338, MIR586, MIR885, MIR34AHG
WIKIPATHWAYS	SCFA and skeletal muscle substrate metabolism	GCG, FFAR3, FFAR2, PPARD, PYY, SLC2A4
WIKIPATHWAYS	Secretion of Hydrochloric Acid in Parietal Cells	ATP4A, CCKBR, CHRM1, GAST, HRH2
WIKIPATHWAYS	Selenium Metabolism and Selenoproteins	CREM, CTH, DIO1, DIO2, DIO3, FABP1, FOS, GPX1, GPX2, GPX3, GPX4, JUN, NFE2L2, NFKB1, POU2F1, RELA, RPL30, SARS, SELENOP, SELENOW, SP1, SP3, TXNRD1, SELENBP1, TXNRD2, SEPHS2, SEPHS1, SEPSECS, SCLY, SELENOT, MSRB1, SARS2, TRNAU1AP, SELENOS, SELENON, SELENOK, EEFSEC, SECISBP2, SELENOO, SELENOI, TXNRD3, PSTK, SELENOM, GPX6, SELENOH, SELENOV
WIKIPATHWAYS	Selenium Micronutrient Network	SERPINA3, ABCA1, ALB, ALOX5, ALOX5AP, ALOX15B, APOA1, APOB, CAT, CBS, SCARB1, CRP, CTH, DIO1, DIO2, DIO3, F2, F7, GGT1, GPX1, GPX2, GPX3, GPX4, GSR, HBA1, HBB, ICAM1, IFNG, IL1B, IL6, INS, INSR, LDLR, MPO, MTHFR, MTR, NFKB1, NFKB2, PRDX1, SERPINE1, PLAT, PLG, PTGS1, PTGS2, RELA, SAA1, SAA2, SAA3P, SAA4, CCL2, SELENOP, SELENOW, SOD1, SOD2, SOD3, PRDX2, TNF, TXN, TXNRD1, XDH, KMO, KYNU, SELENOF, PRDX4, TXNRD2, PRDX3, SEPHS2, PRDX5, SELENOT, MSRB1, PNPO, RFK, SELENOS, SELENON, SELENOK, FLAD1, SELENOO, SELENOI, GGTLC2, GGTLC1, TXNRD3, SELENOM, GPX6, SELENOH, SELENOV, GGT2, SOD2-OT1, MIR6886, LOC102724197

Predefined gene sets in Hepamine

Data source	Pathways	Symbols
WIKIPATHWAYS	Senescence and Autophagy in Cancer	BCL2, BMI1, BMP2, BRAF, CD44, CDC25B, CDKN1A, CDKN1B, CDKN2A, CEBPB, COL1A1, COL3A1, COL10A1, MAPK14, E2F1, FN1, MTOR, CXCL1, GSK3B, GSN, HMGA1, HRAS, IFI16, IFNB1, IFNG, IGF1, IGF1R, IGFBP3, IGFBP5, IGFBP7, IL1A, IL1B, IL3, IL6, IL6R, IL6ST, CXCL8, ING1, ING2, INHBA, INS, IRF1, IRF5, IRF7, JUN, LAMP1, LAMP2, SMAD3, SMAD4, MDM2, KMT2A, MMP14, SERPINE1, SERPINB2, PCNA, PIK3C3, PLAT, PLAU, MAPK1, MAP2K1, MAP2K3, PTEN, RAF1, RB1, RNASEL, CCL3, SPARC, SRC, TGFB1, THBS1, TP53, UVRAG, VTN, ULK1, BECN1, CREG1, SQSTM1, ATG12, ATG5, CXCL14, ATG13, RB1CC1, TNFSF15, ATG7, IL24, GABARAP, GABARAPL2, ATG14, GABARAPL1, FKBP8, RSL1D1, SLC39A1, SLC39A3, SLC39A2, SH3GLB1, ATG16L1, AMBRA1, SLC39A4, MLST8, ATG3, MAP1LC3B, ATG10, AKT1S1, MAP1LC3A, MIR29B2, MIR29C, MAP1LC3C, MIR3606
WIKIPATHWAYS	Serotonin and anxiety	ADRA1A, CAMK2B, CRH, FMR1, FOS, GABRA1, GRM1, HTR1A, HTR2A, HTR2C, PLEK, POMC, PPP3CA, PRKCB, TRPV1, ARC, PLCD4
WIKIPATHWAYS	Serotonin and anxiety-related events	CRH, CRHR1, FOS, GRIN2D, HTR1A, HTR2A, HTR2C, PLEK, PPP3CA, PRKCB, NLGN1, ARC, PLCD4
WIKIPATHWAYS	Serotonin Receptor 2 and ELK-SRF/GATA4 signaling	ELK1, ELK4, GATA4, GNAQ, HRAS, HTR2A, HTR2B, HTR2C, ITPR1, KRAS, NRAS, MAPK1, MAPK3, MAP2K1, MAP2K2, RAF1, RASGRF1, SRF, MAPKAPK2, RASGRP1
WIKIPATHWAYS	Serotonin Receptor 2 and STAT3 Signaling	GNAQ, HTR2A, JAK2, STAT3
WIKIPATHWAYS	Serotonin Receptor 4/6/7 and NR3C Signaling	ATF1, BRAF, CREB1, EGR1, ELK1, ELK4, GNAS, NR3C1, HTR4, HTR6, HTR7, MAPK1, MAPK3, MAP2K1, MAP2K2, RAP1A, SRF, RPS6KA5, MAPKAPK2
WIKIPATHWAYS	Serotonin Transporter Activity	IL1B, IL1R1, ITGB3, MAOA, NOS1, PPP2CB, SLC6A4, STX1A, TGFB1I1, SCAMP2, TPH2

Predefined gene sets in Hepamine

Data source	Pathways	Symbols
WIKIPATHWAYS	Signaling of Hepatocyte Growth Factor Receptor	CRK, CRKL, DOCK1, ELK1, PTK2B, FOS, GAB1, GRB2, RAPGEF1, HGF, HRAS, ITGA1, ITGB1, JUN, MET, PAK1, PIK3CA, MAPK1, MAPK3, MAPK8, MAP2K1, MAP2K2, PTEN, PTK2, PTPN11, PXN, RAF1, RAP1A, RAP1B, RASA1, SOS1, SRC, STAT3, MAP4K1
WIKIPATHWAYS	Signaling Pathways in Glioblastoma	AKT1, AKT2, ARAF, ATM, CCND1, BRCA1, BRAF, BRCA2, CBL, CCND2, CCNE1, CDK2, CDK4, CDK6, CDKN1A, CDKN1B, CDKN2A, CDKN2B, CDKN2C, E2F1, EGFR, EP300, ERBB2, ERBB3, FGFR1, FGFR2, FOXO1, FOXO3, GAB1, GRB2, MSH6, HRAS, IGF1R, IRS1, KRAS, MDM2, MDM4, MET, FOXO4, NF1, NRAS, PDGFRA, PDGFRB, PDPK1, PIK3C2A, PIK3C2B, PIK3C2G, PIK3CA, PIK3CB, PIK3CD, PIK3CG, PIK3R1, PIK3R2, PLCG1, PLCG2, PRKCA, PRKCB, PRKCD, PRKCG, PRKCH, PRKCI, PRKCQ, PRKCZ, MAPK1, MAPK3, MAP2K1, MAP2K2, MAP2K3, MAP2K5, MAP2K6, MAP2K7, PTEN, RAF1, RB1, MAP2K4, SRC, TP53, TSC1, TSC2, AKT3, SPRY2, ERRFI1, MIR1281
WIKIPATHWAYS	Signal Transduction of S1P Receptor	AKT1, AKT2, ASAH1, S1PR1, S1PR3, GNAI1, GNAI2, GNAI3, PIK3C2B, PLCB2, PLCB3, MAPK1, MAPK3, MAPK4, MAPK6, MAPK7, MAPK12, SMPD2, SPHK1, S1PR2, AKT3, PLCB1, RACGAP1, S1PR5, SPHK2, MIR197
WIKIPATHWAYS	Simplified Depiction of MYD88 Distinct Input-Output Pathway	IL1A, IRAK1, JUN, MYD88, NFKB1, TLR1, TLR2, TLR4, TLR5, TRAF6, UBE2N, UBE2V1, TLR6, TLR7, TLR8, TLR9, TLR10, TIFA, MIR718
WIKIPATHWAYS	Simplified Interaction Map Between LOXL4 and Oxidative Stress Pathway	ANXA5, BMP2, DDR1, COL2A1, NQO1, FGF7, FN1, IGFBP7, NFE2L2, NRF1, PKD1, SUV39H1, TGFB1, CDC37, SIRT1, ECSIT, EXOC6, LOXL4, MIR1181, MIR4640, MIR6511B1
WIKIPATHWAYS	Sleep regulation	ADA, ADORA1, ADORA2A, AHCY, CHRNB2, CRH, CST3, DLAT, DRD1, DRD2, DRD3, SLC29A1, FOS, GHRH, UTS2R, GRIN2A, HCRTR1, HCRTR2, HTR2A, IL6, IL18, MTNR1B, NPAS2, NPY2R, OXT, OXTR, PTGDR, PTGDS, STAR, TH, PER3, CACNA1I, UTS2, NLGN1, GHRL, BTBD9, MRGPRX2, NPS

Predefined gene sets in Hepamine

Data source	Pathways	Symbols
WIKIPATHWAYS	Small Ligand GPCRs	CNR1, CNR2, S1PR1, LPAR1, S1PR3, MTNR1A, MTNR1B, PTAFR, PTGDR, PTGER1, PTGER2, PTGER3, PTGER4, PTGFR, PTGIR, TBXA2R, S1PR4, GPR50, S1PR2
WIKIPATHWAYS	Somatroph axis (GH) and its relationship to dietary restriction and aging	AKT1, FOXO1, MTOR, IGF1R, PTEN, SIRT1
WIKIPATHWAYS	Sphingolipid Metabolism	ASAH1, KDSR, GBA, PPP1CA, PPP2CA, PLPP1, SPHK1, SGPL1, B4GALT6, GAL3ST1, SPTLC2, SPTLC1, CERS2, SPTLC3, ASAH2, SPHK2, SERINC1, GBA2, CERK, CERS4, CERS5, SGPP2, SGMS2, CERS3, CERS6
WIKIPATHWAYS	Spinal Cord Injury	ACAN, AIF1, ANXA1, APEX1, AQP1, AQP4, ARG1, RHOA, RHOB, RHOC, CCND1, BDNF, C1QB, C5, CASP3, CCNG1, CD47, CDK1, CDC42, CDK2, CDK4, CDKN1B, LTB4R, COL2A1, COL4A1, VCAN, NCAN, CSPG4, GADD45A, E2F1, E2F5, EFNB2, EGFR, EGR1, EPHA4, FCGR2A, FKBP1A, FOXO3, FOS, GAP43, GDNF, GFAP, GJA1, GRIN1, CXCL1, CXCL2, NR4A1, ICAM1, IFNG, IL1A, IL1B, IL1R1, IL2, IL4, IL6, CXCL8, CXCL10, LEP, LGALS3, LTB, MAG, MBP, MIF, MMP9, MMP12, MYC, NGFR, NOS1, NOS2, OMG, PDYN, PLA2G2A, PLA2G5, PLXNA2, PPP3CA, PRB1, PRKCA, MAPK1, MAPK3, PTGS2, PTPRA, PTPRZ1, RAC1, RB1, ROS1, CCL2, SELP, SLIT1, SLIT3, SOX9, TACR1, TGFB1, TLR4, TNF, TP53, VIM, ZFP36, BTG2, PLA2G6, TNFSF13, FCGR2C, SLIT2, NTN1, ROCK2, LILRB2, TNFSF13B, LILRB3, KLK8, NOX4, CHST11, RGMA, RTN4, SEMA6A, BCAN, XYLT1, RTN4R, MIR23B, CCR2, MIR6869, LOC102725035
WIKIPATHWAYS	Splicing factor NOVA regulated synaptic proteins	ANK3, APLP2, ATP2B1, CAMK2G, CAV2, CDH2, CSN3, DAB1, EFNA5, EPB41, EPB41L1, EPB41L2, STX2, GABRG2, GRIK2, GRIN1, GRIN2B, KCNJ6, KCNMA1, KCNQ2, MAP4, NEO1, PLCB4, PRKCZ, MAPK4, MAPK9, RAP1GAP, STXBP2, CASK, GABBR2, GPHN, CHL1, NTNG1, CLSTN1, SNW1, EPB41L3, NCDN, CLASP1, CADM1, TERF2IP, CADM3, AGRN

Predefined gene sets in Hepamine

Data source	Pathways	Symbols
WIKIPATHWAYS	SREBF and miR33 in cholesterol and lipid homeostasis	ABCA1, FASN, MTOR, HMGCR, HMGCS1, LDLR, PPARA, PRKAA1, SCD, SREBF1, SREBF2, NR1H3, PPARGC1A, SIRT1, SIRT6, MED15, MIR33A, MIR33B, MIR6886
WIKIPATHWAYS	SRF and miRs in Smooth Muscle Differentiation and Proliferation	CAMK2D, CCND2, NKX2-5, ELK1, MEF2A, MEF2C, MEF2D, SRF, KLF4, MYOCD, MIR145, MIR199A1, MEF2B
WIKIPATHWAYS	Statin Pathway	ABCA1, APOA1, APOA2, APOA4, APOB, APOC1, APOC2, APOC3, APOE, SCARB1, CETP, CYP7A1, FDFT1, HMGCR, LCAT, LDLR, LIPC, LPL, LRP1, MTTP, PLTP, SOAT1, SQLE, DGAT1, ABCG5, ABCG8, PDIA2, ACSS1, APOA5, MIR33A, MIR33B, MIR6848, MIR6886
WIKIPATHWAYS	Steroid Biosynthesis	CPN1, CYP17A1, F13B, HSD3B1, HSD3B2, HSD17B1, HSD17B3, HSD17B2, HSD17B4, HSD17B7
WIKIPATHWAYS	Sterol Regulatory Element-Binding Proteins (SREBP) signalling	ACACA, ACLY, AKT1, AMFR, CAMP, SCARB1, CDK8, CREB1, CYP51A1, DBI, FASN, FDFT1, FDPS, MTOR, GSK3A, HMGCR, HMGCS1, IDI1, INS, INSIG1, KPNB1, LDLR, LPL, LSS, MDH1, MVD, NFYA, PIK3CA, PPARG, PRKAA1, PRKAA2, PRKAB1, PRKAB2, PRKACA, PRKAG1, RBP4, SCD, SEC13, SP1, SQLE, SREBF1, SREBF2, NR1H2, YY1, MBTPS1, SEC24C, SEC24D, SEC24B, SEC23B, SEC23A, SEC24A, RNF139, SEC31A, ATF6, SCAP, LPIN1, SIRT1, SEC31B, FGF21, SAR1B, INSIG2, MBTPS2, PRKAG2, MED15, PRKAG3, SAR1A, GPAM, ACSS1, PPARGC1B, MIR33A, MIR33B, MIR6886, MIR6764
WIKIPATHWAYS	Striated Muscle Contraction	ACTA1, ACTA2, ACTC1, ACTG1, ACTN4, ACTN2, ACTN3, DES, DMD, MYBPC1, MYBPC2, MYBPC3, MYH3, MYH6, MYH8, MYL1, MYL2, MYL3, MYL4, NEB, TMOD1, TNNC2, TNNC1, TNNI1, TNNI2, TNNI3, TNNT1, TNNT2, TNNT3, TPM1, TPM2, TPM3, TPM4, TTN, VIM, TCAP, MYOM1, MYL9

Predefined gene sets in Hepamine

Data source	Pathways	Symbols
WIKIPATHWAYS	Structural Pathway of Interleukin 1 (IL-1)	CHUK, MAP3K8, ATF2, MAPK14, EIF4E, ELK1, FOS, MKNK2, IKBKB, IL1A, IL1R1, IL1RAP, IRAK1, IRAK2, IRF7, MBP, MAP3K1, MAP3K3, MYC, MYD88, NFKB1, NFKBIA, NFKBIB, MAPK1, MAPK3, MAPK8, MAPK11, MAPK9, MAPK10, MAP2K1, MAP2K2, MAP2K3, MAP2K6, MAP2K7, RELA, SAFB, MAP2K4, MAP3K7, TRAF6, MKNK1, MAP3K14, RPS6KA5, MAPKAPK2, TANK, TAB1, TAB2, IRAK4, TOLLIP, TAB3, MIR718
WIKIPATHWAYS	Sudden Infant Death Syndrome (SIDS) Susceptibility Pathways	ACADM, ADCYAP1, ADCYAP1R1, ALDOA, SLC25A4, AQP4, AR, PHOX2A, ASCL1, ATP1A3, AVP, BDNF, C4A, C4B, CASP3, CAV3, RUNX3, CEBPB, CHAT, CHRM2, CHRNA4, CHRNA7, CHRNB2, CHRNB4, CPT1A, CREB1, CREBBP, CREM, CTNNB1, DDC, DLX2, ECE1, EGR1, EN1, EP300, ESR2, FOXM1, FMO3, G6PC, GABRA1, GAPDH, GATA2, GATA3, GCK, GJA1, GNB3, GRIN1, NR3C1, HADHA, HADHB, HDAC1, HIF1A, HES1, HSPD1, HTR1A, HTR2A, HTR3A, IL1A, IL1B, IL1RN, IL6, IL6R, CXCL8, IL10, IL13, JUN, KCNH2, KCNJ8, KCNQ1, LMX1B, MAOA, MAP2, MAZ, MBD1, MECP2, MEF2C, MYB, NEUROD1, NFKB1, NFKB2, NFYA, NGF, NKX2-2, NKX3-1, YBX1, NTRK2, PAH, PBX1, PKNOX1, PLP1, POU2F2, POU3F2, POU5F1, PRKACA, PRKACB, PRKAR1A, PRKAR1B, PRKAR2A, PRKAR2B, REST, RET, RORA, RYR2, SCN4B, SCN5A, SLC1A3, SLC6A4, SLC9A3, SNAP25, SNTA1, SOX2, SP1, SP3, SPTBN1, SST, SSTR1, SSTR2, VAMP2, TAC1, TACR1, TCF3, TF, TH, THRB, TNF, TP73, TPH1, HSP90B1, TSPYL1, VEGFA, VIPR1, VIPR2, YWHAB, YWHAE, YWHAG, YWHAH, YWHAZ, BHLHE40, PHOX2B, NOS1AP, HDAC9, DEAF1, CTCF, PPARGC1A, YWHAQ, TPPP, GPD1L, TLX3, FEV, CC2D1A, CDCA7L, SCN3B, NANOG, CHRFAM7A, TPH2, PPARGC1B, HES5, MIR130A, MIR16-1, MIR210, MIR1281, MIR3652, LOC101929970, LOC102723788

Predefined gene sets in Hepamine

Data source	Pathways	Symbols
WIKIPATHWAYS	Sulfation Biotransformation Reaction	G6PD, GSR, SULT1E1, SULT1A2, SULT1A1, SULT1C2, SULT2B1, SULT2A1, PAPSS2, PAPSS1, SULT4A1, SULT1C4, SULT1B1, SULT6B1, SULT1C3, SULT1A4, SLX1A-SULT1A3, SLX1B-SULT1A4, LOC105369243
WIKIPATHWAYS	Sulindac Metabolic Pathway	CYP1A2, CYP1B1, MSRA, MSRB2, MSRB3
WIKIPATHWAYS	Synaptic Vesicle Pathway	AP2A1, AP2A2, AP2B1, SLC25A4, ATP1A2, CACNA1A, CACNA1B, AP2M1, AP2S1, CLTA, CLTC, DNM1, DNM2, STX2, CLN8, NSF, RAB3A, SLC1A3, SLC6A4, SLC18A1, SLC18A2, SLC18A3, SLC22A3, SNAP25, STX1A, STX3, STXBP1, VAMP2, SYN1, SYN2, SYP, SYT1, CLTCL1, SYN3, NAPA, DNM1L, UNC13B, CPLX2, CPLX1, PARK7, RIMS1, UNC13A, DNM3, SLC17A7, SLC17A6, SLC38A1, STX1B, SLC32A1, SLC17A8, UNC13C, CPLX3, MIR3674
WIKIPATHWAYS	Synthesis and Degradation of Ketone Bodies	ACAT1, BDH1, HMGCL, HMGCS2, OXCT1
WIKIPATHWAYS	Tamoxifen metabolism	CYP1A1, CYP1A2, CYP1B1, CYP2A6, CYP2C19, CYP2C8, CYP2C9, CYP2D6, CYP2E1, CYP3A4, CYP3A5, FMO1, FMO3, SULT1E1, SULT1A1, SULT2A1, UGT2B7, UGT2B15, UGT1A10, UGT1A8, UGT1A4
WIKIPATHWAYS	Target Of Rapamycin (TOR) Signaling	AKT1, CDC42, EIF4EBP1, FKBP1A, MTOR, HMGCR, IDI1, PRKAA1, PRKAA2, PRKAB1, PRKAB2, PRKAG1, PRKCA, RAC1, RHEB, RPS6KB1, TSC1, TSC2, ULK1, ULK2, RRAGB, RRAGA, ULK3, PRKAG2, PRKAG3, DDIT4, PRR5, RPTOR, RRAGD, RRAGC, MLST8, MAPKAP1, PRR5L, AKT1S1, DDIT4L, RICTOR, MIR6869
WIKIPATHWAYS	TCA Cycle (aka Krebs or citric acid cycle)	ACO2, CS, DLD, DLST, FH, IDH2, IDH3A, IDH3B, IDH3G, MDH2, OGDH, SDHA, SDHB, SDHC, SDHD, SUCLG2, SUCLG1, SUCLA2
WIKIPATHWAYS	TCA Cycle and Deficiency of Pyruvate Dehydrogenase complex (PDHc)	ACLY, ACO1, CS, DLAT, DLD, DLST, FH, IDH1, IDH3A, MDH1, OGDH, PC, PCK1, PDHA1, SDHA, SUCLG2
WIKIPATHWAYS	TCA Cycle Nutrient Utilization and Invasiveness of Ovarian Cancer	EGFR, JAK1, MAPK1, MAPK3, STAT3

Predefined gene sets in Hepamine

Data source	Pathways	Symbols
WIKIPATHWAYS	T-Cell antigen Receptor (TCR) pathway during Staphylococcus aureus infection	AKT1, CALM1, CALM2, CBL, CD3D, CD4, CD8A, CD28, CD40LG, CDK4, CHUK, MAP3K8, CSF2, CTLA4, DLG1, FOS, FYN, GRB2, GSK3B, IFNG, IKBKB, IL2, IL4, IL5, IL10, ITK, JUN, LCK, LCP2, NCK1, NFATC2, NFKB1, NFKBIA, PAK1, PDCD1, PDK1, PIK3CA, PLCG1, PRKCQ, MAPK3, MAPK9, MAP2K1, MAP2K2, MAP2K7, PTPN6, PTPRC, RAF1, RRAS, SOS1, MAP3K7, TNF, ZAP70, IKBKG, BCL10, MAP3K14, GRAP2, AHSA1, MALT1, CHP1, LAT, ICOS, CARD11
WIKIPATHWAYS	T-Cell antigen Receptor (TCR) Signaling Pathway	AKT1, FAS, CBL, CBLB, CD3D, CD3E, CD3G, CD247, CD4, CD8A, CD28, CDC42, CHUK, CCR5, MAP3K8, CREB1, ATF2, CRK, CRKL, MAPK14, PTK2B, FOS, FYB1, FYN, GATA3, GRB2, HRAS, IKBKB, IL1A, IL1B, IL6, IL9, IL15RA, TNFRSF9, IL17A, IRF4, ITK, ITPR1, JUN, LCP2, LHB, NCK1, NFATC1, NFATC2, NFKB1, NFKBIA, OPRM1, PAK1, PDPK1, PIK3R1, PIK3R2, PLCG1, PRKCD, PRKCQ, MAPK1, MAPK3, MAPK8, MAPK9, MAP2K1, MAP2K2, PTPN11, RAF1, REL, RELA, SHC1, SOS1, MAP3K7, TGFB1, TRAF6, VAV1, VIM, WAS, ZAP70, IKBKG, SKAP1, RIPK2, BCL10, MAP3K14, PSTPIP1, CD83, GRAP2, GAB2, SH2B3, VAV3, MALT1, MAP4K1, LAT, DBNL, ICOS, CARD11, MIR6837
WIKIPATHWAYS	T-Cell Receptor and Co-stimulatory Signaling	AKT1, CALM1, CALM2, CD8A, CD8B, CD28, CSNK1A1, CTLA4, DYRK1A, FYN, GSK3A, GSK3B, IL2, ITK, LCK, NFATC2, NFKB1, NFKBIA, PDCD1, PDK1, PLCG1, PPP3CA, PRKCA, PTEN, PTPN6, RASA1, ZAP70, DYRK2, RASGRP1
WIKIPATHWAYS	TFs Regulate miRNAs related to cardiac hypertrophy	AKT1, AKT2, NFKB1, PPP3R1, RASGRF1, STAT3, TGFB1, MYEF2, MIR1-1, MIR125B1, MIR125B2, MIR133B

Predefined gene sets in Hepamine

Data source	Pathways	Symbols
WIKIPATHWAYS	TGF-beta Receptor Signaling	BMP4, RUNX2, RUNX3, CREBBP, CTNNB1, EGF, ENG, EP300, FKBP1A, FOS, HRAS, IFNG, INHBA, ITGB6, JAK1, JUN, LIF, LTBP1, SMAD1, SMAD2, SMAD3, SMAD4, SMAD5, SMAD6, SMAD7, SMAD9, NFKB1, SERPINE1, MAPK3, MAPK9, SKI, SKIL, SPP1, STAT1, STAT3, TFE3, TGFB1, LEFTY2, TGFBR1, TGFBR2, TGFBR3, TGIF1, THBS1, TNF, WNT1, FOXH1, NOG, ZFYVE9, ZEB2, FST, LEFTY1, ZNF423, BAMBI, LEF1, MIR302A, MIR1281, LINC02478, MIR6869
WIKIPATHWAYS	TGF-beta Signaling Pathway	AKT1, APP, RHOA, ATF3, CCND1, CAV1, RUNX2, CDK1, CDC42, CDKN1A, CDKN2B, COL1A2, KLF6, ATF2, CREBBP, MAPK14, DAB2, E2F4, E2F5, EP300, ETS1, FN1, FOS, FOSB, GRB2, HDAC1, TNC, ITGA2, ITGB1, ITGB3, ITGB4, JUN, JUNB, JUND, LIMK2, SMAD2, SMAD3, SMAD4, SMAD7, MEF2A, MEF2C, MET, MMP1, MMP12, CITED1, MYC, NEDD9, PAK2, PDK1, PIK3R1, PIK3R2, PML, PPM1A, PRKAR2A, MAPK1, MAPK4, MAPK8, MAPK9, MAP2K1, MAP2K2, MAP2K3, MAP2K6, PTK2, RAC1, RAF1, RBL1, RBL2, ROCK1, MAP2K4, SHC1, SKI, SKIL, SKP1, SOS1, SP1, SPTBN1, SRC, MAP3K7, ZEB1, TERT, TFDP1, TGFB1, TGFB1I1, TGFBR1, TGFBR2, TGFBR3, TGIF1, THBS1, KLF10, TP53, TRAF6, UBE2I, SUMO1, NUP214, AXIN1, CUL1, KLF11, PIAS1, FOXH1, BTRC, PIAS2, CCNB2, HGS, ZFYVE9, BCAR1, ZFYVE16, ZEB2, NUP153, RBX1, TRAP1, YAP1, TAB1, COPS5, WWP1, STRAP, MAP4K1, SNW1, NEDD4L, SIN3A, PARD6A, FOXP3, UCHL5, RNF111, DCP1A, SMURF1, STAMBPL1, PJA1, SMURF2, SNIP1, ITCH, SIK1, EID2, MIR1281
WIKIPATHWAYS	TGF-B Signaling in Thyroid Cells for Epithelial-Mesenchymal Transition	AKT1, RUNX2, CDH1, CDH2, CDH6, CDH16, FN1, TNC, ID1, SMAD2, SMAD3, SMAD4, MAPK1, MAPK3, SNAI2, SNAI1, TGFB1, VIM
WIKIPATHWAYS	Tgif disruption of Shh signaling	FGF8, FOXG1, GLI3, SMAD2, NODAL, SHH, TGIF1, NKX2-1, TGIF2

Predefined gene sets in Hepamine

Data source	Pathways	Symbols
WIKIPATHWAYS	The effect of progerin on the involved genes in Hutchinson-Gilford Progeria Syndrome	CHD3, CHD4, E2F1, H3F3A, H3F3B, HDAC1, HDAC2, RB1, RBBP4, RBBP7, SREBF1, SUV39H1, TP53, HIST1H3A, HIST1H3D, HIST1H3C, HIST1H3E, HIST1H3I, HIST1H3G, HIST1H3J, HIST1H3H, HIST1H3B, MBD2, HIST1H3F, MTA1, MTA2, CBX1, CBX3, KDM1A, CBX5, LEF1, INPP5K, MBD3, MTA3, HIST2H3C, HIST2H3A, HIST2H3D, MIR4738, MIR3198-2
WIKIPATHWAYS	The human immune response to tuberculosis	IFI35, IFIT1, IFIT3, IFNAR1, IFNAR2, IFNGR1, IFNGR2, IRF1, JAK1, JAK2, MX1, OAS1, PSMB8, PTPN2, STAT1, STAT2, TAP1, TYK2, IFITM1, PIAS1, SOCS1, IRF9
WIKIPATHWAYS	Thiamine metabolic pathways	BCKDHA, OGDH, PDHA1, TKT, BCKDK, SLC19A2, TPK1, SLC25A19, SLC19A3
WIKIPATHWAYS	Thymic Stromal LymphoPoietin (TSLP) Signaling Pathway	AKT1, BTK, CISH, MAPK14, EIF4EBP1, FES, MTOR, FYN, HCK, IL2RA, IL6, IL7R, CXCL8, JAK1, JAK2, LCK, LYN, MYC, NFKB1, NFKB2, NFKBIA, PIK3CA, MAPK1, MAPK3, MAPK8, MAPK9, MAP2K1, MAP2K2, PTPN11, RELA, RELB, RPS6, CCL11, SRC, STAT1, STAT3, STAT4, STAT5A, STAT5B, STAT6, TEC, TNFSF4, YES1, GAB2, PI4K2A, CRLF2, TSLP
WIKIPATHWAYS	Thyroxine (Thyroid Hormone) Production	CGA, SLC5A5, TG, TPO, TRH, TSHR
WIKIPATHWAYS	TLR4 Signaling and Tolerance	CHUK, IFNB1, IKBKB, IL6, CXCL8, INPP5D, IRAK1, IRF3, IRF7, MYD88, NFKB1, NFKBIA, MAP3K7, TLR4, TNF, TRAF3, TRAF6, IKBKG, RIPK1, IKBKE, TAB1, IRAK3, TAB2, TRAM1, TBK1, IRAK4, TIRAP, TICAM1, MIR718, MIR6502

Predefined gene sets in Hepamine

Data source	Pathways	Symbols
WIKIPATHWAYS	TNF alpha Signaling Pathway	AKT1, APAF1, BIRC2, BIRC3, BAD, BAX, BCL2L1, BID, CASP3, CASP7, CASP8, CASP9, CHUK, MAP3K8, CREBBP, CSNK2A1, CYBA, GLUL, GRB2, HRAS, HSP90AA1, IKBKB, IL6, JUN, KRAS, MAP3K1, MAP3K3, MAP3K5, NFKB1, NFKB2, NFKBIA, NFKBIB, NFKBIE, NRAS, PLK1, PPP2CA, PRKCZ, MAPK1, MAPK3, MAPK8, MAPK9, MAP2K3, MAP2K6, MAP2K7, PSMD2, PTPRCAP, PYGL, MAP4K2, RAC1, RAF1, REL, CCL2, SELE, MAP2K4, SKP1, SMPD2, SOS1, MAP3K7, TNF, TNFAIP3, TNFRSF1A, TNFRSF1B, TRAF1, TRAF2, TXN, NSMAF, CUL1, IKBKG, MADD, TRADD, RIPK1, FADD, CFLAR, KSR1, BTRC, MAP3K14, TANK, TRAP1, TAB1, RIPK3, CDC37, TAB2, FBXW11, NOX1, TBK1, RFK, DIABLO, OTUD7B, RFFL, NOXO1, TAB3, KSR2, MIR1181, MIR7846
WIKIPATHWAYS	TNF related weak inducer of apoptosis (TWEAK) Signaling Pathway	AKT1, AKT2, BIRC2, BIRC3, CASP3, CASP7, CASP8, CHUK, MAPK14, CTNNB1, GSK3B, HDAC1, IKBKB, IL6, JUN, MMP9, NFKB1, NFKB2, NFKBIA, NFKBIB, MAPK1, MAPK3, MAPK8, MAPK9, RAC1, RAF1, RELA, RELB, CCL2, CCL5, MAP3K7, TNF, TRAF1, TRAF2, TRAF3, TRAF5, RIPK1, TNFSF12, FADD, MAP3K14, TNFRSF12A, TRIM63
WIKIPATHWAYS	Toll-like Receptor Signaling	CHUK, IKBKB, IRAK1, IRF3, IRF7, MYD88, NFKB1, NFKB2, MAPK1, REL, RELA, RELB, TLR1, TLR2, TLR3, TLR4, TLR5, TRAF3, TRAF6, IKBKG, IKBKE, TLR6, TBK1, IRAK4, TLR7, TLR8, TLR9, TOLLIP, TIRAP, TICAM1, TICAM2, MIR718

Data source	Pathways	Symbols
WIKIPATHWAYS	Toll-like Receptor Signaling Pathway	AKT1, AKT2, CASP8, CD14, CD80, CD86, CD40, CHUK, MAP3K8, MAPK14, FOS, IFNA1, IFNA2, IFNA4, IFNA5, IFNA6, IFNA7, IFNA8, IFNA10, IFNA13, IFNA14, IFNA16, IFNA17, IFNA21, IFNAR1, IFNAR2, IFNB1, IKBKB, IL1B, IL6, CXCL8, IL12A, IL12B, CXCL10, IRAK1, IRF3, IRF5, IRF7, JUN, LBP, CXCL9, MYD88, NFKB1, NFKB2, NFKBIA, PIK3CA, PIK3CB, PIK3CD, PIK3CG, PIK3R1, PIK3R2, MAPK1, MAPK3, MAPK8, MAPK11, MAPK9, MAPK10, MAPK13, MAP2K1, MAP2K2, MAP2K3, MAP2K6, MAP2K7, RAC1, RELA, MAPK12, CCL3, CCL4, CCL5, CXCL11, MAP2K4, SPP1, STAT1, MAP3K7, TLR1, TLR2, TLR3, TLR4, TLR5, TNF, TRAF3, TRAF6, PIK3R3, IKBKG, RIPK1, FADD, IKBKE, AKT3, TLR6, TAB1, TAB2, PIK3R5, LY96, TBK1, IRAK4, TLR7, TLR8, TLR9, TOLLIP, TIRAP, TICAM1, TICAM2, MIR718
WIKIPATHWAYS	TP53 Network	ABL1, ATM, BAX, BCL2, BID, BOK, CDKN1A, CDKN2A, GADD45A, MDM2, MYC, OTX2, PMAIP1, TP53, TP73, SUMO1, TP63, TNFSF10, BBC3, MIR3191
WIKIPATHWAYS	Transcriptional cascade regulating adipogenesis	KLF5, CEBPA, CEBPB, CEBPD, CEBPG, DDIT3, EGR2, GATA2, GATA3, PPARG, SREBF1, KLF2, KLF15
WIKIPATHWAYS	Transcription factor regulation in adipogenesis	CEBPA, CEBPB, CEBPD, CREB1, FOXO1, NR3C1, IL6, INSR, IRS1, LEP, PCK2, PPARG, MAPK8, RXRA, SLC2A4, TNF, TWIST1, NRIP1, IRS2, ADIPOQ, PPARGC1A, LPIN1
WIKIPATHWAYS	Translation Factors	EEF1A1, EEF1A2, EEF1B2, EEF1D, EEF1G, EEF2, EIF1AX, EIF2S1, EIF2B1, EIF2S3, EIF4A1, EIF4A2, EIF4B, EIF4E, EIF4EBP1, EIF4EBP2, EIF4G1, EIF5, EIF5A, ETF1, EIF3E, EIF6, EIF2AK2, EIF4H, EIF4EBP3, EIF3A, EIF3B, EIF3C, EIF3D, EIF3F, EIF3G, EIF3H, EIF3I, EIF3J, EIF4G3, EIF2B4, EIF2B3, EIF2B2, EIF2B5, EIF2S2, EIF1AY, EIF2AK3, EIF5B, EIF1, PAIP1, CLUH, GSPT2, PABPC1, EIF2AK1, EEF2K, EIF3CL, MIR1248, LOC101930123, MIR7705

Predefined gene sets in Hepamine

Data source	Pathways	Symbols
WIKIPATHWAYS	Trans-sulfuration and one carbon metabolism	AHCY, AMT, BHMT, CBS, CTH, DHFR, DNMT1, DNMT3A, DNMT3B, GCLC, GCLM, GSS, MAT1A, MAT2A, MTHFD1, MTHFR, MTR, PSPH, SHMT1, SHMT2, TYMS, AHCYL1, MTHFD2, AHCYL2, MTHFD1L, PHGDH, MAT2B, DNMT3L, PSAT1, DHFR2, MTHFD2L, MIR6778
WIKIPATHWAYS	Trans-sulfuration pathway	AHCY, CBS, CTH, DNMT1, GCLM, GOT1, MPST, MTR, MAT2B, CSAD
WIKIPATHWAYS	Triacylglyceride Synthesis	GK, GK2, GPD1, LIPC, LIPE, LPL, GNPAT, LIPF, AGPS, PLPP1, PLPP2, PLPP3, DGAT1, AGPAT1, AGPAT2, AGPAT5, AGPAT3, AGPAT4, PNPLA2, GPAM, MOGAT2, DGAT2, MOGAT1, MOGAT3, MIR6848, MIR6721
WIKIPATHWAYS	Tryptophan catabolism leading to NAD+ production	IDO1, TDO2, KMO, KYNU, NAMPT, NMNAT2, QPRT, HAAO, NMRK2, AADAT, NMRK1, NADSYN1, NMNAT1, NAPRT, AFMID, NMNAT3
WIKIPATHWAYS	Tryptophan metabolism	AANAT, AOC1, ACAT1, ALDH1A1, ALDH2, ALDH9A1, ALDH3A2, AOX1, ASMT, CAT, CYP1A1, CYP1A2, CYP1B1, CYP2A13, CYP2C18, CYP2E1, CYP2F1, CYP2J2, CYP3A4, CYP19A1, DDC, DHCR24, ECHS1, GCDH, HSD17B10, HADH, PRMT1, IDO1, MAOB, MDM2, OGDH, TDO2, TPH1, UBE3A, WARS, ALDH1A2, KYNU, CYP7B1, INMT, HAAO, AADAT, UBR5, RNF25, CYP4F12, AFMID, ACMSD
WIKIPATHWAYS	Tumor suppressor activity of SMARCB1	ACTL6A, CDK4, CDK6, CDKN2A, EZH2, GLI1, GLI2, GLI3, GLI4, H3F3A, H3F3B, PTCH1, RB1, RBBP4, DPF2, SMARCA4, SMARCB1, SMARCC1, SMARCC2, SMARCD1, SMARCD2, SMARCD3, SMARCE1, SMO, DPF3, DPF1, ARID1A, EED, SUZ12, ACTL6B, ARID1B, MIR4738, MIR6755
WIKIPATHWAYS	Type 2 papillary renal cell carcinoma	ARNT, CDKN1A, CREBBP, CTSK, DIAPH1, DVL2, EP300, EPAS1, FH, HIF1A, PDGFB, PRCC, SFPQ, SLC2A1, ELOC, ELOB, TFE3, TGFA, TGFB1, TGFB2, TGFB3, VEGFA, VHL, TFEB, CUL2, ARNT2, RBX1, SETD2, EGLN1, BIRC7, COL21A1, EGLN2, EGLN3, CADM2, MIR1281, MIR5572

Predefined gene sets in Hepamine

Data source	Pathways	Symbols
WIKIPATHWAYS	Type II diabetes mellitus	CACNA1A, MTOR, GK, IKBKB, INSR, PDX1, IRS1, KCNJ11, PHKA2, PRKCD, PRKCZ, MAPK1, MAPK8, SLC2A2, SLC2A4, SURF1, TNF, ADIPOQ, PIK3R5, SOCS4, MAFA, INS-IGF2
WIKIPATHWAYS	Type III interferon signaling	IL10RB, JAK1, STAT1, STAT2, TYK2, IRF9, IFNLR1, IFNL2, IFNL3, IFNL1
WIKIPATHWAYS	Type II interferon signaling (IFNG)	CYBB, IFI6, GBP1, HLA-B, ICAM1, IRF8, IFIT2, IFNA2, IFNB1, IFNG, IFNGR1, IFNGR2, IL1B, CXCL10, IRF1, IRF2, IRF4, JAK1, JAK2, CIITA, CXCL9, NOS2, OAS1, PRKCD, EIF2AK2, PSMB9, PTPN11, REG1A, SPI1, STAT1, STAT2, TAP1, HIST2H4A, SOCS1, SOCS3, ISG15, IRF9
WIKIPATHWAYS	TYROBP Causal Network	ZFP36L2, C1QC, C3, CAPG, RUNX3, CD4, CD37, DPYD, ELF4, GPX1, HCLS1, NCKAP1L, HLX, IL10RA, IL13RA1, IL18, ITGAM, ITGAX, ITGB2, LYL1, MAF, NCF2, PLEK, RGS1, RNASE6, RPS6KA1, SLC1A5, SPP1, STAT5A, TGFBR1, TNFRSF1B, TYROBP, CD84, SLC7A7, KCNE3, SH2B3, LHFPL2, ABCC4, IGSF6, TCIRG1, NPC2, FKBP15, GIMAP2, PYCARD, BIN2, CYTL1, RBM47, APBB1IP, ADAP2, CXCL16, SAMSN1, CREB3L2, GAL3ST4, LOXL3, TMEM106A, PPP1R18, GAPT, LGALS9B, SFT2D2, NRROS, LGALS9C, MIR4709, MIR7846
WIKIPATHWAYS	Ultraconserved region 339 modulation of tumor suppressor microRNAs in cancer	TP53, CCNE2, MIR95, MIR339, MIR663B
WIKIPATHWAYS	Urea cycle and metabolism of amino groups	ACY1, ARG1, ARG2, ASL, ASS1, CKB, CKM, CPS1, GAMT, GATM, GLUD1, OAT, ODC1, OTC, PYCR1, ALDH18A1, SMS, SRM, PYCR3, NAGS
WIKIPATHWAYS	Valproic acid pathway	ABAT, ACADSB, CYP2A6, CYP2B6, CYP2C9, EHHADH, HSD17B10, HADHA, HADHB, HDAC1, IVD, UGT1A3, ACSM1

Data source	Pathways	Symbols
WIKIPATHWAYS	VEGFA-VEGFR2 Signaling Pathway	ACACA, ACACB, ACP1, JAG1, AKT1, BIN1, ANXA1, FAS, ARF6, RHOA, RHOC, ARRB2, ATF4, CCND1, BCL2, BCL2L1, CAPN2, CAV1, CBL, CDC42, CDH5, CFL1, CLTC, CREB1, ATF2, CRK, MAPK14, CSK, CCN2, CTNNA1, CTNNB1, CTNND1, CYP2C8, DOK1, RCAN1, HBEGF, EGR1, EGR3, EIF4E, ELK1, EPS15, ERG, ERN1, ETS1, F3, PTK2B, FGA, FGB, FGG, FHL2, FOXO1, FOXO3, FLNB, FLT1, MTOR, FYN, GAB1, GJA1, GPC1, GRB2, GRB10, RAPGEF1, GSK3B, HDAC1, NR4A1, HRAS, HSPA1A, HSPB1, HSP90AA1, ICAM1, IGFBP7, CXCL8, ITGAV, ITGB1, ITGB3, ITGB5, JUN, KCNC3, KDR, LIMK1, DNAJB9, MEF2C, MAP3K5, AFDN, FOXO4, MMP2, MMP10, MMP14, MYH9, MYL2, MYO6, NCF2, NCK1, NCL, NFATC1, NFATC2, NFKB1, NFKBIA, NOS3, NOTCH4, NR4A2, PAK1, PAK2, PDPK1, PFN1, PGF, PIK3CA, PIK3R1, PIK3R2, PLA2G4A, PLA2G5, PLAU, PLAUR, PLCB3, PLCG1, PPP3CA, PRKAA1, PRKAA2, PRKCA, PRKCB, PRKCD, PRKCE, PRKCI, PRKD1, PRKCZ, PRKG1, MAPK1, MAPK3, MAPK8, MAPK9, MAP2K1, MAP2K2, MAP2K3, MAP2K6, MAP2K7, PTGS2, PTK2, PTPN1, PTPN6, PTPN9, PTPN11, PTPRJ, PTPRZ1, PXN, RAB4A, RAB5A, RAC1, RAF1, RAP1A, RAP1B, RELA, ROCK1, RPS6, RPS6KB1, MAPK12, CCL2, SELE, MAP2K4, SHB, SHC1, SLC8A1, SMARCA2, SOD2, SRC, SRF, STAT1, STAT3, STAT6, ELOA, TEAD4, TNXB, TRPC1, TXN, VAV2, VCL, VEGFA, EZR, YWHAE, NR4A3, STAM, EEA1, MAPKAPK5, MKNK1, RAB11A, FADD, TNFRSF10C, INPP4B, IQGAP1, NRP2, WASF1, SH2D2A, HGS, RPS6KA5, MAPKAPK2, KL, EIF2AK3, ROCK2, ADAMTS1, BCAR1, HERPUD1, HDAC9, HDAC4, HDAC5, RCAN2, NDRG1, RACK1, TXNIP, CAMKK2, GIPC1, FRS2, FAF1, ARHGEF15, ATF6, DKK1, FBXW11, SHC2, PRKD2, RND1, BMP10, EPN1, HDAC7, DLL4, PBK, ACKR3, RHOJ, MLST8, MAPKAP1, MMRN2, ITCH, EIF2A, AKT1S1, AMOT, TPCN2, RICTOR, NCF1, SOD2-OT1, MIR4329, OCLN

Predefined gene sets in Hepamine

Data source	Pathways	Symbols
WIKIPATHWAYS	Viral Acute Myocarditis	ABL1, ABL2, ACTB, PARP1, AIF1, AKT1, BAX, CCND1, BCL2, BCL2L1, BID, BNIP2, CASP1, CASP2, CASP3, CASP6, CASP7, CASP8, CASP9, CAV1, CD4, CD80, CD40LG, CCR3, CCR5, CREB1, CD55, DAG1, DFFA, DFFB, DMD, EDN1, EIF4G1, EIF4G2, ENDOG, FYN, GSK3B, HLA-DMA, IFNG, IL2, IL6, IL10, IL12A, IL12B, ITGAL, ITGB2, JAK1, KRT8, LAMA2, MMP9, MYH6, NFKB2, NOS1, PIK3R1, MAPK1, MAPK3, RAC2, RAC3, RASA1, SGCA, SGCB, SGCD, SGCG, SOS1, SRC, STAT1, STAT3, TGFB1, TLR3, TLR4, TLR5, TNF, CXCR4, SOCS1, PABPC1, PYCARD, JKAMP, CHRAC1, CYCS, NOD2, CAAP1, TICAM1, PTCRA, MICA, MIR7705
WIKIPATHWAYS	Vitamin A and Carotenoid Metabolism	ADH1A, ADH4, ALDH1A1, ALDH1A3, CD36, SCARB1, CRABP1, CRABP2, CYP2E1, CYP26A1, LPL, MAPK1, RARA, RARB, RARG, RBP1, RBP2, RBP4, RDH5, RLBP1, RPE65, RXRA, RXRB, RXRG, SULT1A1, SULT2B1, DGAT1, ALDH1A2, LRAT, DHRS3, NPC1L1, RDH8, BCO1, RETSAT, CYP26B1, ABCG5, ABCG8, BCO2, RBP7, RDH12, RDH10, AWAT2, SDR16C5, MIR6848
WIKIPATHWAYS	Vitamin B12 Metabolism	SERPINA3, ABCA1, ALB, APOA1, APOB, APOE, CBS, SCARB1, CRP, CTH, F2, F7, CBLIF, HBA1, HBB, ICAM1, IFNG, IL1B, IL6, INS, INSR, LDLR, LRP2, MAT1A, MPO, MTHFR, MTR, MTRR, MMUT, NFKB1, NFKB2, SERPINE1, PLAT, PLG, RELA, SAA1, SAA2, SAA3P, SAA4, CCL2, CCL5, SHMT2, SOD1, SOD2, SOD3, TCN1, TCN2, TNF, CUBN, MCEE, MMAB, SOD2-OT1, MIR6886
WIKIPATHWAYS	Vitamin B6-dependent and responsive disorders	ALPI, ALDH7A1, AASS, PNPO, PIGV
WIKIPATHWAYS	Vitamin D Metabolism	CYP24A1, CYP27A1, CYP27B1, DHCR7, GC, PTH, RXRA, RXRB, VDR, CYP2R1

Predefined gene sets in Hepamine

**Data source**	**Pathways**	**Symbols**
WIKIPATHWAYS	Vitamin D Receptor Pathway	ADRA1B, ADRB2, ABCD1, ALOX5, ALPI, ALPG, STS, ATP2B1, CCND1, BCL6, BDKRB1, BGLAP, CEACAM1, PRDM1, BMP6, CA9, S100G, CAMP, CASP5, CBS, CCNC, CCNE1, CD9, CD14, CD40, ADGRE5, CDC34, CDK2, CDKN1A, CDKN1B, CDKN2A, CDKN2B, CDKN2C, CDKN2D, CDX2, CEBPA, COL13A1, CST1, CST6, CTLA4, CYP1A1, CYP2B6, CYP2C9, CYP2D6, CYP3A4, CYP3A5, CYP7A1, CYP24A1, CYP27B1, GADD45A, DEFB4A, EFNA5, SERPINB1, EPHB4, FOXO1, G6PD, HIF1A, HLA-DQA1, HLA-DQA2, HLA-DRB1, HSD17B2, IRF8, ID1, ID4, IGFBP1, IGFBP3, IGFBP5, IL12A, IRF4, IRF5, ITGAM, JUNB, KNG1, KRT13, KRT16, KRT34, LGALS9, LRP5, MXD1, CD200, MX2, MYC, MYO9B, NFATC2, NINJ1, TNFRSF11B, ORM1, ORM2, ABCB1, PNOC, PPARD, PRKCQ, KLK6, PTGER4, PTH, PTHLH, RXRA, S100A2, S100A4, S100A6, S100A8, S100A9, SATB1, SFRP1, SLC2A4, SLC8A1, SPP1, SPRR1B, SULT1C2, SULT2A1, HNF1A, TGFB1, TGFB2, THBD, TIMP2, TIMP3, TNFAIP3, TPM1, TNFSF4, VDR, SEMA3B, STAM, FGF23, NRIP1, TNFSF11, KRT38, ASAP2, CLDN2, IL1RL1, KLF4, KL, ATP2C2, LPGAT1, RASGRP1, SLC34A2, ADAMTS5, DUSP10, TRAK1, IGSF9B, CLEC16A, CASP14, SOSTDC1, LCE2B, NOX1, CYP2S1, HILPDA, G0S2, TREM1, CDKAL1, MED9, TRPV6, TRPV5, SALL4, IL25, COLEC11, MEG8, STEAP4, CLMN, ABCA11P, CLPTM1L, CRACR2A, KRTAP4-1, DNER, KRT71, ZNF257, LRRC25, BTLA, DACT2, CREG2, SLC37A2, CRACR2B, KRTAP8-1, LCE1D, LCE1F, KRTAP12-2, DND1, KRTAP10-4, KRTAP10-7, KRTAP10-9, KRTAP10-2, KRTAP5-1, KRTAP5-4, DEFB132, GXYLT2, MIR6872, LOC105369230
WIKIPATHWAYS	Vitamins A and D - action mechanisms	RARA, RXRA, VDR
WIKIPATHWAYS	White fat cell differentiation	KLF5, CEBPA, CEBPB, CEBPD, CREB1, CTNNA1, DDIT3, EBF1, EGR2, MECOM, FOXO1, GATA2, GATA3, NR3C1, INS, IRF3, IRF4, PPARG, RARA, RORA, SREBF1, STAT5A, STAT5B, NR2F2, TLE3, WNT10B, KLF4, NR1H3, KLF2, ZNF423, KLF15, TCF7L1, MIR1469

Predefined gene sets in Hepamine

Data source	Pathways	Symbols
WIKIPATHWAYS	Wnt/beta-catenin Signaling Pathway in Leukemia	AKT1, APC, CCND1, BCL9, RUNX1T1, CSNK1A1, CTNNB1, FLT3, GSK3B, JUP, LRP6, LRP5, MYC, PML, PPARD, RARA, TCF3, WNT1, ZBTB16, AXIN2, FZD6, WIF1, DKK1, PYGO1, LEF1, SALL4
WIKIPATHWAYS	Wnt Signaling in Kidney Disease	RHOA, CTNNB1, DVL1, DVL2, DVL3, FZD2, LRP6, LRP5, MAPK8, MAPK9, MAPK10, WNT1, WNT2, WNT3, WNT5A, WNT6, WNT7A, WNT7B, WNT10B, WNT11, WNT2B, WNT9B, FZD5, FZD3, FZD1, FZD4, FZD6, FZD7, FZD8, FZD9, INVS, WNT16, WNT4, WNT10A, WNT5B, WNT3A, MIR4683, LOC101929777, MIR6808
WIKIPATHWAYS	Wnt Signaling Pathway	APC, RHOA, CCND1, CAMK2A, CAMK2B, CAMK2D, CAMK2G, CCND2, CCND3, CSNK1A1, CSNK1E, CSNK2A1, CSNK2A2, CSNK2B, CTBP1, CTBP2, CTNNB1, DVL1, DVL2, DVL3, GPC4, FZD2, GSK3B, JUN, LRP6, LRP5, MYC, NFATC1, NFATC2, NFATC3, NFATC4, ROR1, ROR2, SERPINF1, PLAU, PLCB2, PLCB3, PLCB4, PPP3CA, PPP3CB, PPP3CC, PPP3R1, PPP3R2, PRKCA, PRKCB, PRKCG, MAPK8, MAPK9, RAC1, RYK, SFRP1, SFRP2, SFRP4, SFRP5, MAP3K7, TCF7, TCF7L2, WNT1, WNT2, WNT3, WNT5A, WNT6, WNT7A, WNT7B, WNT10B, WNT11, WNT2B, FZD5, FZD3, FOSL1, AXIN1, FZD1, FZD6, FZD7, FZD8, FZD9, CER1, ROCK2, FRAT1, WIF1, FZD10, DKK1, DAAM1, PLCB1, FRAT2, DAAM2, DKK4, DKK2, INVS, SOST, LEF1, WNT16, NLK, WNT4, CTNNBIP1, VANGL2, CHD8, SENP2, SOX17, PORCN, CXXC4, WNT10A, WNT5B, VANGL1, TCF7L1, KREMEN1, NKD1, NKD2, WNT3A, CSNK1A1L, PRICKLE1, NOTUM, PRICKLE2, CSNK2A3, MIR4683, LOC101929777, MIR6808, TPTEP2-CSNK1E, AKT1, APC, RHOA, ARRB2, CCND1, BCL9, CDK6, CSNK1A1, CSNK1D, CSNK1E, CTBP1, CTNNB1, DVL1, DVL2, DVL3, MTOR, GCKR, GJA1, GSK3A, GSK3B, LRP6, LRP5, MYC, NFATC2, ROR1, ROR2, PPARG, PRKCA, PRKCB, PRKCG, MAPK1, MAPK8, MAPK9, RAC1, RYK, SOX1, MAP3K7, TCF4, TCF3, TCF7L2, TEK, TSC1, TSC2, AXIN1, AXIN2, PIP5K1B, FRAT1, LEF1, NLK, CSNK1G1, PI4K2A, MIR6808, TPTEP2-CSNK1E

Predefined gene sets in Hepamine

Data source	Pathways	Symbols
WIKIPATHWAYS	Wnt Signaling Pathway and Pluripotency	APC, RHOA, CCND1, CCND2, CCND3, CD44, CREBBP, CSNK1E, CTBP1, CTBP2, CTNNB1, CTNND1, DVL1, DVL2, DVL3, EP300, ESRRB, FZD2, GSK3B, JUN, LDLR, LRP6, LRP5, MMP7, MYC, NFYA, PAFAH1B1, PLAU, POU5F1, PPARD, PPP2CA, PPP2CB, PPP2R1A, PPP2R1B, PPP2R2A, PPP2R2B, PPP2R2C, PPP2R3A, PTPA, PPP2R5C, PPP2R5E, PRKCA, PRKCB, PRKCD, PRKCE, PRKCG, PRKCH, PRKCI, PRKD1, PRKCQ, PRKCZ, MAPK9, MAPK10, MAP2K4, SOX2, MAP3K7, TCF7, TCF7L2, TP53, WNT1, WNT2, WNT3, WNT5A, WNT6, WNT7A, WNT7B, WNT10B, WNT11, WNT2B, WNT9B, FZD5, FZD3, FOSL1, AXIN1, AXIN2, FZD1, FZD4, FZD6, FZD7, FZD8, FZD9, ZBTB33, FRAT1, FZD10, FBXW2, FOXD3, PPP2R3B, RACGAP1, LEF1, WNT16, NLK, WNT4, NANOG, WNT10A, WNT5B, TCF7L1, NKD1, NKD2, WNT3A, LRRK2, PPM1J, MIR1281, MIR4683, LOC101929777, MIR6886, MIR6808, TPTEP2-CSNK1E
WIKIPATHWAYS	Zinc homeostasis	MT1A, MT1B, MT1E, MT1F, MT1G, MT1H, MT1M, MT1L, MT1X, MT2A, MT3, MTF1, SLC30A1, SLC30A2, SLC30A3, SLC30A4, SLC39A7, SLC30A9, SLC39A14, SLC39A6, SLC39A1, SLC39A3, SLC39A2, SLC39A9, SLC30A10, SLC39A4, SLC30A6, SLC39A10, SLC39A8, SLC30A5, MT4, SLC39A13, SLC30A7, SLC30A8, SLC39A11, SLC39A12, SLC39A5
